# ﻿Systematics and biogeography of Appalachian Anillini, and a taxonomic review of the species of South Carolina (Coleoptera, Carabidae, Trechinae, Anillini)

**DOI:** 10.3897/zookeys.1209.125897

**Published:** 2024-08-08

**Authors:** Curt W. Harden, Michael S. Caterino

**Affiliations:** 1 Department of Plant and Environmental Sciences, Clemson University, 277 Poole Agricultural Center, Clemson, South Carolina, 29634, USA Clemson University Clemson United States of America

**Keywords:** Evolution, eyeless, flightless, phylogenetics, predaceous, subterranean biology

## Abstract

In the eastern United States, 74 species of Anillini in two genera have been described, with most belonging to *Anillinus* Casey. Until now, no systematic framework has existed for this large genus, hampering integrative studies. Using DNA sequences from 101 Nearctic species, we present a well-resolved molecular phylogeny supporting a sound systematic framework. Sixteen species groups of Appalachian *Anillinus* are diagnosed, in part using newly recognized variation in the number of modified male protarsi and the state of the spermathecal duct. We present the first descriptions of Nearctic anilline larvae, which possess none of the synapomorphies of previously described anilline larvae. Within *Anillinus*, two major clades are mostly consistent with setation of the right paramere: a “hairy clade” with more than four setae, and a “quadrisetose clade.” Throughout the phylogeny, microhabitat use varies within each clade, and several endogean lineages are phylogenetically isolated. Our work increases the South Carolina fauna by nearly five-fold. Nine new species are described, *Serranillusmonadnock***sp. nov.**, *Anillinuscastaneus***sp. nov.**, *Anillinuschoestoea***sp. nov.**, *Anillinusdentatus***sp. nov.**, *Anillinusjancae***sp. nov.**, *Anillinusmica***sp. nov.**, *Anillinusmicamicus***sp. nov.**, *Anillinusseneca***sp. nov.**, and *Anillinussimplex***sp. nov.** Several species are newly reported from South Carolina, bringing the total to 20 described species representing seven species groups. Two endemic groups inhabit deep clay soils in the Piedmont and possess unique male sexual characters. The Anillini are a unique component of Nearctic biodiversity, with great potential as a model system for studies of biogeography, secondary male sexual modification, and endogean adaptations.

## ﻿﻿Introduction

Members of the tribe Anillini are small, flightless, predaceous ground beetles, ubiquitous in most temperate and tropical regions of the world, with the notable exception of eastern Asia (Jeannel 1963a; Andújar et al. 2016). More than 620 valid species have been described, all entirely eyeless, with the exceptions of the subtribe Nesamblyopina (Sokolov 2023) and the poorly known African genera *Microdipnodes* Basilewsky and *Cryptorites* Jeannel (Jeannel 1963a). Anillines inhabit a variety of dark, interstitial habitats and typically have very small ranges consistent with their limited dispersal capabilities.

**Figure 1.**
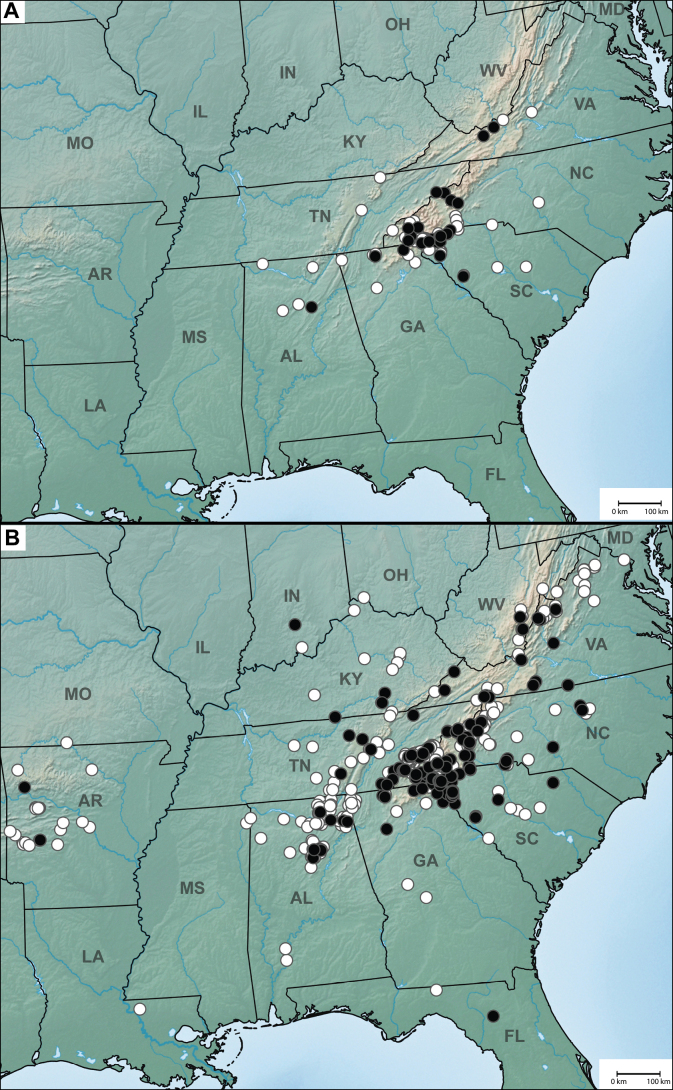
Distribution maps of eastern Nearctic Anillini**A***Serranillus***B***Anillinus*. White dots: all known occurrences. Black dots: locations of new DNA sequence vouchers from this study. Data from Harden (2024).

The United States east of the Mississippi River is home to 58 described anilline species in two genera, *Anillinus* Casey and *Serranillus* Barr. A third genus, *Stylulus* Schaufuss, has been reported from the region as well (Barr 1969; Cornell 1972, 1977a; Erwin and Sims 1984), but the identity of these records has not been confirmed. Members of *Anillinus* also occur west of the Mississippi River, in Arkansas (4 spp.), Missouri (1 spp.), Oklahoma (2 spp.), and Texas (9 spp.) (Sokolov 2022; Sokolov et al. 2004, 2014; Sokolov and Watrous 2008). *Serranillus* is restricted to the eastern United States, with a relatively small range (Fig. 1A) that is nested within that of *Anillinus* (Fig. 1B). In eastern North America, anillines have been collected from a wide variety of elevations and microhabitats, varying from deep sand near sea level in Florida (Sokolov and Schnepp 2021) to coniferous litter on the summit of Mount Mitchell in North Carolina, the highest point east of the Mississippi River (Carnegie Museum of Natural History, Pittsburgh data). The collective range of the tribe in the eastern United States has its northern limits in Maryland, southern Ohio, and the southern counties of Indiana (Dury 1902; Sokolov et al. 2004). Known occurrence data (Harden 2024) suggest that anillines may be expected to occur anywhere south of this line. Most records are from montane localities, but this likely is an artifact of collecting bias. At lower elevations, where conditions are warmer and drier, anillines are more difficult to collect by hand, except during exceptionally wet periods following heavy rains (Barr 1995; Carlton et al. 2005). In the “TAG” karst region at the corner of Tennessee, Alabama, and Georgia, *Anillinus* can be found in caves, including a small number of species that appear to be strict cave inhabitants or troglobites (Jeannel 1963b; Sokolov 2012, 2020). *Anillinus* have rarely been found in caves outside this region, and no apparent troglobites are known except in TAG caves. Most *Anillinus* found in caves are collected in very small numbers, and may actually be inhabitants of the smaller interstices in the “Milieu Souterrain Superficiel” or “Mesovoid Shallow Substratum” (MSS; Mammola et al. 2016) that enter caves only occasionally.

**Figure 2.**
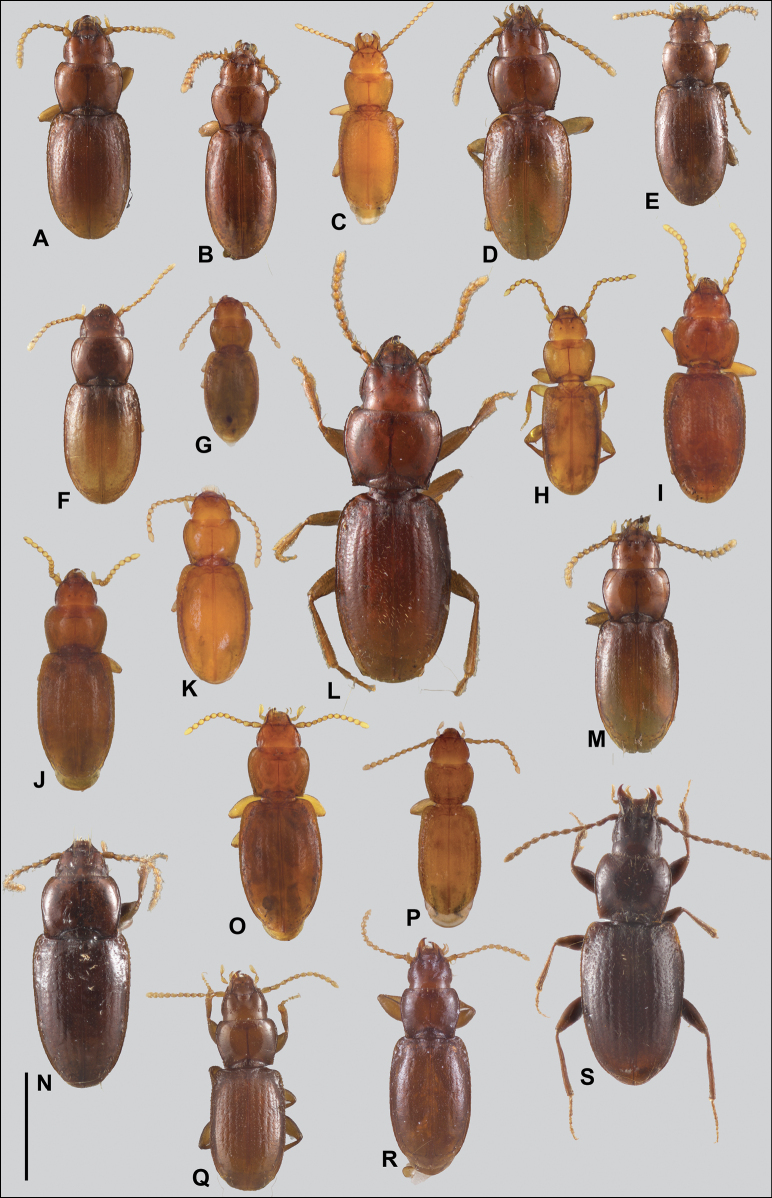
Habitus of eastern Nearctic Anillini. **A***Serranillusdunavani* (South Carolina, Oconee Co.) **B***Anillinusmoseleyae* (Tennessee, Sevier Co.) **C***Anillinusdentatus* (South Carolina, Abbeville Co.) **D***Serranillusjeanneli* (South Carolina, Oconee Co.) **E***Anillinusfolkertsioides* (Alabama, Blount Co.) **F***Anillinuspecki* (North Carolina, Avery Co.) **G***Anillinus* sp. “Georgia, Barnes Creek sp. 1” **H***Anillinusmontrex* (South Carolina, York Co.) **I***Anillinus* sp. “Tennessee, Kings Saltpeter Cave” **J***Anillinusvirginiae* (Virginia, Bath Co.) **K***Anillinuserwini* (Virginia, Grayson Co.) **L***Serranillus* sp. (North Carolina, Graham Co.) **M***Anillinus* sp. “South Carolina, Waldrop Stone” (South Carolina, Pickens Co.) **N***Anillinus* sp. “Alabama, Horseshoe Cave sp. 1” **O***Anillinusloweae* (North Carolina, Haywood Co.) **P***Anillinus* sp. “Kentucky, Hestand sp. 1” **Q***Anillinusindianae* (Indiana, Lawrence Co.) **R**Anillinuscf.barberi (West Virginia, Greenbrier Co.) **S***Anillinushirsutus* (Alabama, Madison Co.). Scale bar: 1 mm.

Anillines in eastern North America are diverse in body size and structure (Fig. 2). The smallest species known is an undescribed *Anillinus* species from northern Georgia (Fig. 2G) and the largest are some members of the genus *Serranillus* (Fig. 2L). Structural differences in convexity and overall habitus are associated with different microhabitats. For example, species readily collected from leaf litter are usually convex and ovoid in outline (Fig. 2A, F, G, K, O), whereas those inhabiting endogean (= deep soil) habitats are usually dorsoventrally flattened and more parallel-sided (Fig. 2B, C, E, H, P, Q). Species in rock interstices and caves are also typically flattened, but usually have relatively broad elytra with stronger humeral angles (Fig. 2I, N, S). However, the distinction between these three broad categories is often not clear, and some species are intermediate in both body structure and microhabitat use (Fig. 2D, J, L, M, R). While litter, soil and caves are convenient habitat “bins” for sorting species, the range of microhabitats experienced by animals less than 3 mm in length is probably more diverse.

Historically, taxonomic progress on Nearctic Anillini has been slow. One of the earliest descriptions of an anilline was the Appalachian Mountain endemic *Anillinusfortis* (Horn, 1868), but nearly a century would pass before any additional valid Appalachian species would be named (Jeannel 1963a). Barr (1969) published a brief but thorough overview of the anilline fauna of the United States. His point map of eastern anillines (fig. 9) has remained the most complete reference for their collective range in the region to date, including many geographically isolated species known to him in 1969, but that remain undescribed today. His lone taxonomic contribution to Anillini (Barr 1995) established the genus *Serranillus* and included valuable information about collecting techniques, but most of Barr’s extensive knowledge of the group has remained unpublished. The groundbreaking review of *Anillinus* by Sokolov et al. (2004) set in motion a modern period of taxonomic study that has steadily revealed the true diversity of Nearctic Anillini during the past two decades (Sokolov et al. 2007; Sokolov and Carlton 2008, 2010; Giachino 2011; Sokolov 2011, 2021; Sokolov and Schnepp 2021; LaBonte and Maddison 2023; Harden and Caterino 2024), including description of species based on Barr’s material, now at the Carnegie Museum of Natural History (Sokolov 2012, 2014, 2020). An early classification scheme of *Anillinus* based on presumed microhabitat use and patterns of dorsal microsculpture (Sokolov et al. 2004) has gradually been abandoned as additional new species and COI sequence data have challenged this classification (Sokolov and Carlton 2010; Sokolov 2020; Sokolov and Schnepp 2021). There is currently no systematic framework for this large genus, which includes at least 70 undescribed species (Harden 2024).

In this paper, we present an updated systematic framework of eastern Anillini based on a molecular phylogeny and newly recognized morphological variation, including novel male secondary modifications. The first larvae of Nearctic Anillini are described. We also present a taxonomic review of the surprisingly diverse and unique South Carolina anilline fauna, describing nine new species, redescribing four species, and designating a neotype for *Serranillusjeanneli* Barr. Combined with a distributional dataset for eastern anillines based upon all published occurrences and the examination of thousands of physical specimens (Harden 2024), our molecular phylogeny reveals the complex biogeographic history of eastern anillines.

## ﻿﻿Materials and methods

### ﻿﻿Field collecting

We used litter sifting, litter extraction, soil washing, turning embedded rocks, and soil trapping to collect anillines. Litter sifting involved placing a small amount of deep leaf litter into a ¼” or ½” mesh metal screen and shaking over a white plastic bin, which was then checked for beetles. Litter extraction involved sifting large amounts of litter through a metal screen into a cloth bag, and then placing the sifted material in Berlese funnels (with incandescent light bulbs as a heat source) or Winkler extractors and leaving it for several days. Soil washing involved digging up mineral soil with a large shovel, sifting it through a screen, and then slowly adding it to large buckets filled halfway with water; the mixture was then stirred vigorously and allowed to settle before skimming off the organic material floating on the surface; this material was dried in a cloth bag for one or more days and then processed in a Berlese funnel or placed on metal screens above containers set in direct sunlight (Maquet et al. 2018; Andújar and Grebennikov 2021). In some cases, the sifted soil itself was directly placed in Berlese funnels instead of mixing with water. Turning embedded rocks involved using a metal pry bar to loosen and dislodge rocks deeply embedded in the soil. The underside of the rock and the soil surface beneath it were carefully scanned while wearing a bright headlamp. Soil trapping was conducted using “pipe traps” modified from the design shown in LaBonte and Maddison (2023), with PVC pipes (5.08 cm outer diameter) cut to 20.32 cm lengths and cut with 1 cm wide latitudinal slots spaced along most of the length; a plastic container (Sarstedt #75.9922.421) was attached to one end of the pipe using duct tape. An initial hole was dug to a depth of 15–40 cm and a shaft was created at the bottom of this hole using an auger (Art’s Manufacturing and Supply, Inc. SKU: 400.48). A small amount of propylene glycol was added to the plastic container, and the pipe assembly was inserted in the shaft with a plastic sleeve inserted to keep soil out while refilling the soil around the pipe. A plastic lid with a piece of bright flagging tape attached with fishing line was placed on top and reburied, with the flagging marking the trap above ground. Traps were left in place for three months or longer. All of these collecting methods produced anilline specimens. For pipe traps and most hand collecting, geographical coordinates were taken using a Garmin GPSMAP 64 GPS receiver. For some hand collecting events and all literature records for which coordinates were not given, approximate coordinates were obtained retroactively using Google Earth; these have reduced digits to indicate less accuracy.

### ﻿﻿Morphology

Terminology of most structures follows that of Slipinski and Lawrence (2013). Mandibular tooth terminology follows Maddison (1993). Designations of “dorsal” and “ventral” faces of the median lobe of the aedeagus follow typical convention for carabids, and not their orientation in repose or when everted. Designation of “right” and “left” parameres of the aedeagus also follow typical convention (Sokolov 2022). The sclerotized structures in the internal sac of the median lobe have traditionally been referred to as “dorsal sclerites” and “ventral sclerites” in Nearctic anilline literature (e.g., Sokolov et al. 2004). We consider the “dorsal sclerites” to be equivalent with the flagellum, and refer to it as such; the ejaculatory duct connects to the base of this structure, and we consider it homologous across the taxa treated. Sokolov et al. (2007) used the schematic of the internal sac of *Lionepha* in Erwin and Kavanaugh (1981) to homologize their “dorsal sclerites”; an apparent flagellum is lacking in *Lionepha*, and homology between what we term the flagellum and any of the sclerotized structures shown in Erwin and Kavanaugh (1981) is uncertain. We refer to other sclerotized structures in the internal sac variously based on their shape and position within the sac; these structures are unlikely to be homologous across taxa, and so we avoid the use of a single term for them. Our interpretation of “lateral aspect” differs from that of Sokolov (e.g., 2012). We consider the lateral aspect to be that viewed when the basal lobes of the median lobe are parallel to the plane on which they rest. Most aedeagi of the species are strongly asymmetrical and twisted from this plane, and the aspect illustrated as “left lateral aspect” in previous papers on Appalachian Anillini is an aspect we consider a dorsal or dorsolateral aspect.

Measurements of the length of body regions were taken using calibrated images in Adobe Photoshop and are given using the following abbreviations in the descriptions and key.

**ABL** Apparent body length, measured from anterior edge of clypeus to apex of elytra

**EL** Elytral length, measured from posterior edge of scutellum to apex of elytra

**EW** Maximum elytral width

**HW** Maximum head width

**PbW** Pronotal basal width, measured at posterior angles and not including external denticles

**PL** Pronotal length, measured along midline

**PW** Maximum pronotal width

**RL** Ring sclerite length

External structures were examined using Amscope (SKU: SM-1BSL-V331), Leica M80, and Olympus SZX7 stereoscopes, at 7–100 × magnifications. Some mouthparts and legs were further examined by placing in glycerin and viewing with a Motic BA300 compound microscope. Dissection of male and female genitalia was performed using Dumont #5 forceps (Item nos. 11251-20 and 11252-20, www.finescience.com/) and bent #000 and minuten insect pins held in short pin vises. For males that had been through the DNA extraction process, removal of the ring sclerite and aedeagus was performed by tearing the tergites with minuten pins and pulling the structures out from the dorsal side. Specimens that were previously dry mounted or stored in ethanol were first relaxed in a warm water bath for 0.5 hr or more, then placed in a drop of weak ethanol or water on a depression slide; fine forceps were used to stabilize the specimen, with the tip of the index finger inserted between the forceps to allow better control and prevent crushing the specimen, and the ring sclerite and aedeagus were removed from the posterior abdominal opening using a bent minuten pin; such genitalia usually required further clearing by placing in 0.5 mL centrifuge vials containing 85% lactic acid or 10% potassium hydroxide and placing in a warm water bath for 15 min or longer to remove hardened musculature. In the case of specimens from vials from which the fluid had evaporated, labels were removed and a small amount of ammonia-based cleaner was sprayed into the vial and left for 0.5 hr or more to relax and clean the specimens; specimens treated in this way were usually pliable enough to be dissected without a warm water bath. To study female genitalia, the entire abdomen was removed and cleared (either by DNA extraction or using short treatments of warm 85% lactic acid or 10% potassium hydroxide) and the genital segment was carefully removed and studied in glycerin. For especially small specimens or single female specimens, the genital segment was left in the abdomen and the spermatheca was studied through the abdominal ventrites. Genitalic structures were studied in glycerin on depression slides using a Motic BA300 compound microscope and photographed using a Canon Powershot A2200 digital camera. Some median lobes were further cleared by placing in clove oil after rinsing in ethanol, and left to sit overnight before viewing. In most cases, genitalia were stored in glycerin in microvials pinned beneath the specimens. Male genitalia of *Anillinus* holotypes and some female genitalia were mounted in Euparal on plastic microslides pinned beneath the specimens. Both the aedeagus and the spermatheca are asymmetrical; mounting them permanently prevents studying their true structure, and for this reason was usually avoided.

Hand line drawings of male genitalia were first made with pen and ink, with proportions traced from printed photographs, and then scanned and digitized in Adobe Illustrator. Habitus photographs were taken with a Visionary Digital Passport II system with a Canon 6D SLR and 65-mm MP-E 1–5X macro lens, with focus stacking performed in Helicon Focus (www.heliconsoft.com). Scanning electron micrographs of uncoated specimens affixed to stubs with double sided tape were taken at 15.0kV in BSE and BSE3D modes using a Hitachi S-3400 Variable Pressure Scanning Electron Microscope (SEM) at the Clemson University Scanning Electron Microscopy Facility in Anderson, SC.

### ﻿﻿Material examined

Approximately 7,000 specimens of Anillini were examined. The dataset of Harden (2024) provides data for 6,438 of these. All specimens examined are deposited in the following collections.

**AMDc** Anthony M. Deczynski personal collection, Central, SC, USA

**ADGc** Augusto DeGiovanni personal collection, Bologna, Italy

**CMNH**Carnegie Museum of Natural History, Pittsburgh, PA, USA

**CMC** Cincinnati Museum Center, Cincinnati, OH, USA

**CNC**Canadian National Collection of Insects, Ottawa, Canada

**CUAC**Clemson University Arthropod Collection, Clemson, SC, USA

**CWHc** Curt Harden personal collection, Central, SC, USA

**FSCA**Florida State Collection of Arthropods, Gainesville, FL, USA

**GRSM** Great Smoky Mountains National Park collection, Gatlinburg, TN, USA

**KESc** Kyle E. Schnepp personal collection, Gainesville, FL, USA

**LSAM**Louisiana State Arthropod Museum, Baton Rouge, LA, USA

**NCSU**North Carolina State University, Raleigh, NC, USA

**NHMUK**The Natural History Museum, London, United Kingdom

**OSAC**Oregon State Arthropod Collection, Corvalis, OR, USA

**OSUC**Ohio State University Collection, Cleveland, OH, USA

**TLc** Todd Lawton personal collection, Winnipeg, MB, Canada

**UGCA**University of Georgia Collection of Arthropods, Athens, GA, USA

**USNM**United States National Museum of Natural History, Washington, D.C., USA

**VMNH**Virginia Museum of Natural History, Martinsville, VA, USA

The holotypes of four eastern Nearctic species, *Anillinuscampbelli* Giachino, *Anillinusdohrni* (Ehlers), *Anillinusfortis* (Horn), and *Anillinuspecki* Giachino, were studied only from digital photographs. All other eastern Nearctic anilline holotypes were physically studied, except for six that could not be located. *Anillinusclinei* Sokolov, *Anillinusfolkertsioides* Sokolov, *Anillinushildebrandti* Sokolov, and *Anillinushumicolus* Sokolov were deposited at the CMNH (Sokolov 2020) but could not be found there during a search in August 2023 by CWH and R. Davidson. They have since been located at the CMNH (R. Androw, pers. comm., April 2024) but have not yet been studied by us. The type specimens of *Anillinussteevesi* Barr and *Serranillusjeanneli* Barr were reportedly deposited in the CMNH as well (Barr 1995), but are not present in the type collection, general collection, or the unprocessed Barr material at the CMNH; we believe these types were either lost or were never actually designated by Barr. We designate a neotype for *S.jeanneli* because that species is treated in this paper.

We note that the holotype of *Anillinusrelictus* Sokolov, to be deposited at the CMNH, was at the USNM as of August 2023, and the holotype of *Anillinuscornelli* Sokolov & Carlton, to be deposited at the USNM, was at the NCSU as of March 2022.

### ﻿﻿Distribution maps

All of the coordinates used to create the dot maps in this paper are provided in the dataset of Harden (2024), available at https://doi.org/10.5281/zenodo.10983000. In addition to material examined, this file includes all additional, unique published occurrences of Anillini in the Appalachian, Ozark, and Ouachita regions (Jeannel 1963a; Cornell 1977a, 1977b; Barr 1995; Sokolov et al. 2004, 2007, 2017; Sokolov and Carlton 2008, 2010; Sokolov and Watrous 2008; Giachino 2011; Sokolov 2011, 2012, 2014, 2020, 2021; Sokolov and Schnepp 2021). Coordinates were converted to decimal degrees, and where no coordinates were given in the original citation, approximate coordinates were obtained using Google Earth.

### ﻿﻿Taxonomy

We follow the modified biological species concept of Coyne and Orr (2004), and therefore consider species to be independently evolving units of individuals that are reproductively isolated from other independently evolving units. Multiple instances of syntopy allowed testing of this concept in several cases. Several species described below are allopatric with respect to other members of their clades. In such cases, there are multiple males known and sufficient morphological differences in the median lobe shape and flagellum shape to provide strong support for their hypothetical species status. DNA sequence data are available from all species described as new except *Serranillusmonadnock* sp. nov., which is morphologically distinct from other members of the genus in external structure and male genitalia. All species formally described by us in this paper are monophyletic in our molecular phylogenies, in addition to being morphologically distinctive.

Our species-level taxonomic work in this paper is limited to species occurring in South Carolina. Several additional undescribed species are cited in this paper and in the supplementary checklist of all known eastern anillines (Suppl. material 3), but not described. We use informal placeholder names for these, formed by the state and locality from which specimens were first studied and recognized as new species. These include five species from South Carolina that are either known from insufficient material, or are allopatric to their most similar relatives and cannot be ruled out as one end of a grade.

Two abbreviations are used to indicate uncertainty in identifications: “cf.” stands for the Latin “confer” and is used for specimens that most closely fit our interpretation of the species name that follows, but for which we are not confident of this identification, due to either the sex of the individual or the uncertain identity of the type specimen; “aff.” stands for the Latin “affinis” and is used for a specimen that most closely resembles the species name that follows, but for which we have evidence to indicate the specimen does not belong to that species.

Larvae of *Serranillusdunavani* (Jeannel) and *Anillinusjancae* sp. nov. were associated with adults using DNA sequences.

### ﻿﻿Molecular phylogenetics

DNA sequences for our analyses came from 125 species, including 101 Nearctic Anillini species, representatives of 16 genera of Anillini from other regions, and seven far outgroups representing the other tribes of Trechitae. The 101 Nearctic Anillini included 42 previously described species, eight species described as new in this paper, and 51 additional undescribed species that will be described in future papers. Twenty-five previously described species have sequences published for the first time. Five previously described eastern Nearctic species are represented only by previously published COI sequences, because we did not collect fresh specimens of them.

DNA was extracted from 488 individuals using ThermoFisher’s GeneJet extraction kit (Vilnius, Lithuania) using the standard protocol of the manufacturer, except that in some specimens the elution volume was reduced to increase DNA concentration. For most specimens, DNA was extracted from the abdomen only. Some earlier vouchers were extracted from whole bodies.

We amplified fragments of two nuclear ribosomal genes (18S and 28S), one mitochondrial protein-coding gene (cytochrome oxidase I, 5’ [COIbc] and 3’ [COIjp] ends), and three nuclear protein-coding genes (carbamoyl phosphate synthetase domain of the rudimentary gene [CAD2 and CAD4], wingless [Wg], muscle-specific protein 300 [MSP]) using primers from Folmer et al. (1994), Simon et al. (1994), Wild and Maddison (2008), Moulton and Wiegmann (2004), Ward and Downie (2005), Maddison and Cooper (2014), Ober (2002), and Shull et al. (2001); primers, PCR programs, and PCR protocols are given in Tables 1–3. Cleaning and Sanger sequencing of PCR products was performed by Psomagen, Inc. (Maryland, USA).

**Table 1.** List of primers used in amplification (_a_) and Sanger sequencing (_b_).

Gene	Location	Primer	Direction	Sequence	Reference
COIbc	mitochondrial	LCO1490_a,b_	forward	GGTCAACAAATCATAAAGATATTGG	Folmer et al. (1994)
COIbc	mitochondrial	HCO2198 _a,b_	reverse	TAAACTTCAGGGTGACCAAAAAATCA	Folmer et al. (1994)
COIjp	mitochondrial	Jerry _a,b_	forward	CAACATTTATTTTGATTTTTTGG	Simon et al. (1994)
COIjp	mitochondrial	Pat _a,b_	reverse	TCCAATGCACTAATCTGCCATATTA	Simon et al. (1994)
CAD	nuclear	CD439F _a,b_	forward	TTCAGTGTACARTTYCAYCCHGARCAYAC	Wild and Maddison (2008)
CAD	nuclear	CD806F _a,b_	forward	GTNGTNAARATGCCNMGNTGGGA	Moulton and Wiegmann (2004)
CAD	nuclear	CD688R _a,b_	reverse	TGTATACCTAGAGGATCDACRTTYTCCATRTTRCA	Wild and Maddison (2008)
CAD	nuclear	CD1098R2 _a,b_	reverse	GCTATGTTGTTNGGNAGYTGDCCNCCCAT	Wild and Maddison (2008)
CAD	nuclear	CD1231R_a_	reverse	TCCACGTGTTCNGANACNGCCATRCA	Wild and Maddison (2008)
Wg	nuclear	wg550F_a_	forward	ATGCGTCAGGARTGYAARTGYCAYGGYATGTC	Wild and Maddison (2008)
Wg	nuclear	wg578F _a,b_	forward	TGCACNGTGAARACYTGCTGGATG	Ward and Downie (2005)
Wg	nuclear	wgAbR _a,b_	reverse	ACYTCGCAGCACCARTGGAA	Wild and Maddison (2008)
Wg	nuclear	wgAbRZ_a_	reverse	CACTTNACYTCRCARCACCARTG	Wild and Maddison (2008)
MSP	nuclear	MSP1F_a_	forward	CGAGAYGARGTYGATAARATGATGCA	Maddison and Cooper (2014)
MSP	nuclear	MSP2F _a,b_	forward	GCYGGACAAAAGGARATYAAYCARTGG	Maddison and Cooper (2014)
MSP	nuclear	MSP1R _a,b_	reverse	TCWACCAGATCCATCCACTTGACCAT	Maddison and Cooper (2014)
28S	nuclear	D1F _a,b_	forward	GGGAGGAAAAGAAACTAAC	Ober (2002)
28S	nuclear	D3R _a,b_	reverse	GCATAGTTCACCATCTTTC	Ober (2002)
18S	nuclear	18S5 _a,b_	forward	GACAACCTGGTTGATCCTGCCAGT	Shull et al. (2001)
18S	nuclear	18Sb5 _a,b_	reverse	TAACCGCAACAACTTTAAT	Shull et al. (2001)

**Table 2.** PCR programs. All began with an initial denaturing phase of 180 s at 94 °C, with each cycling phase including 30 s of denaturing at 94 °C and 30 s of annealing; from Maddison (2012).

Program	Denaturing temperature	Annealing temperature	Extension temperature	Extension time (s)	Final extension time (s)	Cycles
C1	94	52	72	45	180	35
C1alt	94	48	72	45	180	35
C2	94	50	72	150	150	35
C3	94	52	72	120	120	37
C4	94	54	72	150	150	35
C5	94	55	72	90	90	37
C7	94	60, 55	72	120	120	9, 30
C9	94	57, 52, 45	72	120	120	6, 6, 36

**Table 3.** PCR protocols for gene fragments. For nested and hemi-nested reactions, programs and primers for inner and outer reactions are indicated by ^1^ and ^2^, respectively.

Gene	Program	Primer 1	Primer 2
18S	C2	18SF5	18Sb5
28S	C1	D1F	D3R
COIbc	C1alt	LCO1490	HCO2198
COIjp	C1alt	Jerry	Pat
CAD2	C7^1^, C5^2^	CD439F^1,2^	CD688R^2^, CD1098R2^1^
CAD4	C7^1^, C5^2^	CD806F^1,2^	CD1098R2^2^, CD1231R^1^
Wg	C3^1^, C4^2^	wg550F^1^, wg578F^2^	wgAbRZ^1^, wgAbR^2^
MSP	C9^1,2^	MSP1F^1^, MSP2F^1^	MSP1R^1,2^

In total, 1,446 new sequences were generated for this study. Chromatogram assembly and base calls were made using Geneious (ver. 8.1.8; Auckland, NZ). Multiple peaks were scored with ambiguity codes. GenBank accession numbers for the new sequences are OR830242, OR837782–OR838296, OR839193–OR839823, and OR853105–OR853407. Names of taxa and molecular voucher codes associated with each are listed in Suppl. material 2.

The new sequences were supplemented by 191 sequences from previous studies (Sokolov et al. 2007; Sokolov and Watrous 2008; Sokolov and Carlton 2010; Hildebrandt and Maddison 2011; Maddison and Ober 2011; Maddison 2012; Andújar et al. 2016, 2017; Maddison et al. 2019; LaBonte and Maddison 2023; Harden et al. 2024).

Six of the eight species described as new have DNA sequences available from the holotypes: *A.dentatus* (OR853208, OR839248), *A.simplex* (OR853342, OR839331, OR839570, OR837888, OR838032, OR838222), *A.choestoea* (OR839239, OR839627, OR838052), *A.mica* (OR853287, OR839293, OR838076), *A.micamicus* (OR853291, OR839296, OR838075), *A.seneca* (OR853323, OR839466). DNA sequences from the holotypes of three previously described species are also newly published: *A.arenicollis* (OR853123, OR839200, OR838072), *A.montrex* (OR853113, OR853294, OR839300, OR839565, OR837884, OR838029, OR838218, OR838127), and *A.uwharrie* (OR853370, OR839348, OR839693, OR838093, OR838261). These holotype sequences are ‘genseq-1’ (sensu Chakrabarty et al. 2013). All eight new *Anillinus* species have DNA sequences from paratypes (‘genseq-2’), which are indicated in the descriptions. The previously described species *A.montrex* and *A.arenicollis* have newly published ‘genseq-2’ sequences (i.e., from secondary types) as well, which are indicated in the species treatments. Three previously described species have new ‘genseq-2’ sequences and are not otherwise treated in the taxonomic section: *Anillinusalbrittonorum* Sokolov & Schnepp (OR839194, OR839530, OR837859, OR838193, OR853109, OR853120, OR839195, OR839545, OR837868, OR838014, OR838202, OR838123), *Anillinuspittsylvanicus* (OR853207, OR839245, OR839412, OR837796, OR837957, OR838147), and *Anillinusuwharrie* (OR853371, OR839349, OR839694, OR839346, OR839695, OR853369, OR839347, OR839696). DNA sequences from three species redescribed in this paper are ‘genseq-3’, and are indicated in the redescriptions. Three previously redescribed species that are not otherwise treated in the taxonomic section have ‘genseq-3’ sequences published for the first time: *Anillinusdocwatsoni* Sokolov & Carlton (OR839709, OR853213, OR839253, OR839702, OR838096, OR853214, OR839254, OR839701, OR837928, OR838095, OR838263, OR853211, OR839251, OR839703, OR853212, OR839252), *Anillinuspecki* Giachino (OR839313, OR839560, OR837881, OR838214, OR853309, OR839732, OR853307, OR839733, OR853310, OR839314, OR839758, OR837942, OR838103, OR838281, OR839760, OR839762, OR839741, OR839740, OR853308, OR839768, OR837943, OR838104, OR853311, OR839315, OR839562, OR837882, OR838026, OR838215, OR853312, OR839563, OR838027, OR838216, OR853313, OR839316, OR839555, OR837878, OR838023, OR838211, OR838125, OR853314, OR839317, OR838085, OR838254), and *Anillinuselongatus* Jeannel (OR853218, OR839257, OR839688, OR853219, OR839258, OR839689, OR853216, OR839255, OR839690, OR838091, OR838260, OR853217, OR839256, OR839691). Only a photo voucher is available for the larva of *A.seneca* sp. nov., and so the 28S sequence from the specimen (OR853242) is ‘genseq-5’. All of the remaining new sequences are from specimens that are deposited in collections as vouchers, and are therefore ‘genseq-4’.

Alignment of sequences was performed in Mesquite (Maddison and Maddison 2023b). The ribosomal genes 18S and 28S included numerous insertions and deletions, and were aligned using the L-INS-I option in MAFFT version 7.490 (Katoh and Standley 2013). Sequences from the protein coding genes were mostly free of indels, and were aligned manually. However, several amino acid insertions and deletions were apparent among the outgroups in Wg, CAD2, and MSP. These were aligned by first translating the nucleotides to amino acids using Mesquite (Characters>Make New Matrix from>Translate DNA to Protein), aligning the amino acid matrix using the same MAFFT settings as for the ribosomal genes, and finally aligning the nucleotides to the amino acid alignment (Alter>Align DNA to Protein…).

The modified GBLOCKS algorithm in Mesquite (Talavera and Castresana 2007) was used to select and exclude ambiguously aligned regions of 18S and 28S, using the settings specified by Maddison et al (2019): minimum fraction of identical residues for conserved portions = 0.2, minimum fractions of identical residues for highly-conserved positions = 0.4, counting only fraction within taxa with non-gaps at that position, maximum length of non-conserved blocks = 4, minimum length of a block = 4, fraction of gaps allowed in a character = 0.5, and with sites selected in ambiguously aligned regions.

Two matrices were assembled for analyses. For the 6-gene concatenated analyses, a “core matrix” was used, containing individuals for which three or more of the six genes had been sequenced and individuals belonging to unique species for which only COI sequences were available, from a total of 269 individuals. A “complete matrix” was used for the remaining single-gene analyses, containing all of the newly generated sequences and all available Nearctic anilline sequences, from 551 individuals. Both matrices included the same outgroups: 17 anillines and seven far outgroups representing the other tribes of Trechitae.

Maximum likelihood analyses were performed in IQ-TREE using the Zephyr package (Maddison and Maddison 2023a) in Mesquite (Maddison and Maddison 2023b). For the six-gene concatenated analysis and the single-gene analyses of protein-coding genes, the TESTMERGE option was used to select the best model and partitioning scheme, starting with 18 parts, one each for 18S and 28S and one for each codon position of the protein coding genes. For single gene analyses of 18S and 28S, the MFP option was used, with the data unpartitioned. 100 search replicates were performed for the six-gene concatenated matrix and the single gene analyses. For 28S, two analyses were run, with and without site exclusion with GBLOCKS. Node support was calculated using standard bootstrapping in IQ-TREE, with 500 replicates for all analyses and the same options used in the Maximum Likelihood analyses. Node support for and against hypothetical clades was calculated using the “Frequency of clades in trees” feature in Mesquite, and are shown in Fig. 4.

## ﻿﻿Results

### ﻿﻿Molecular phylogenetics

**Figure 3.**
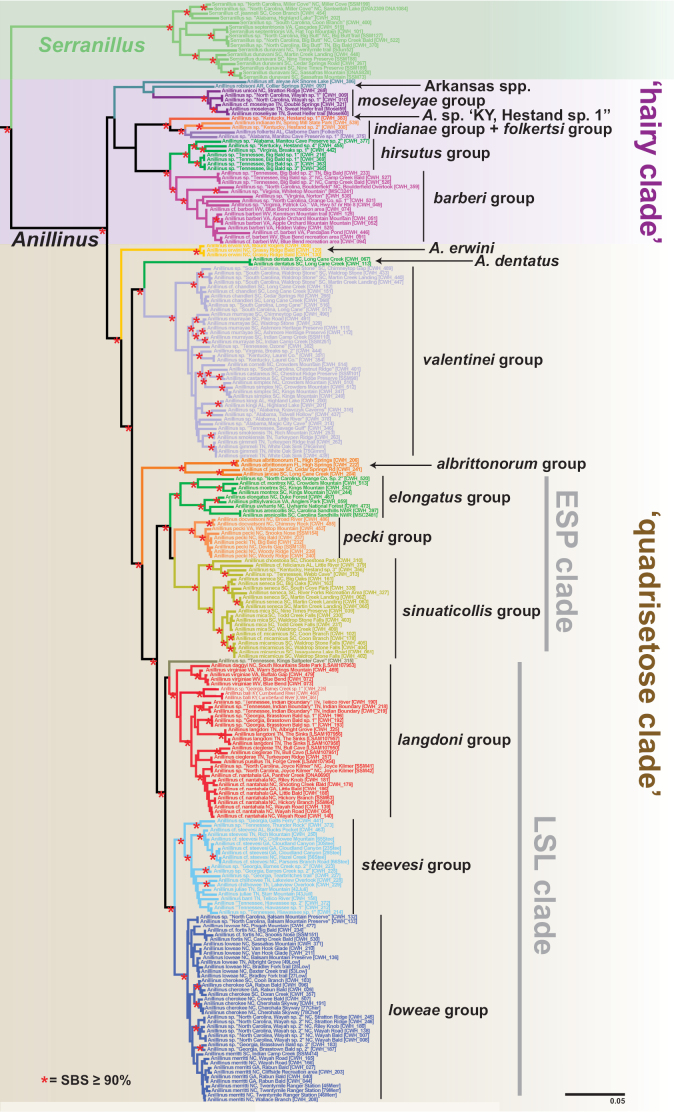
Maximum likelihood tree of 6-gene concatenated matrix. Outgroups cropped. Red asterisks = SBS 90% or greater.

**Figure 4.**
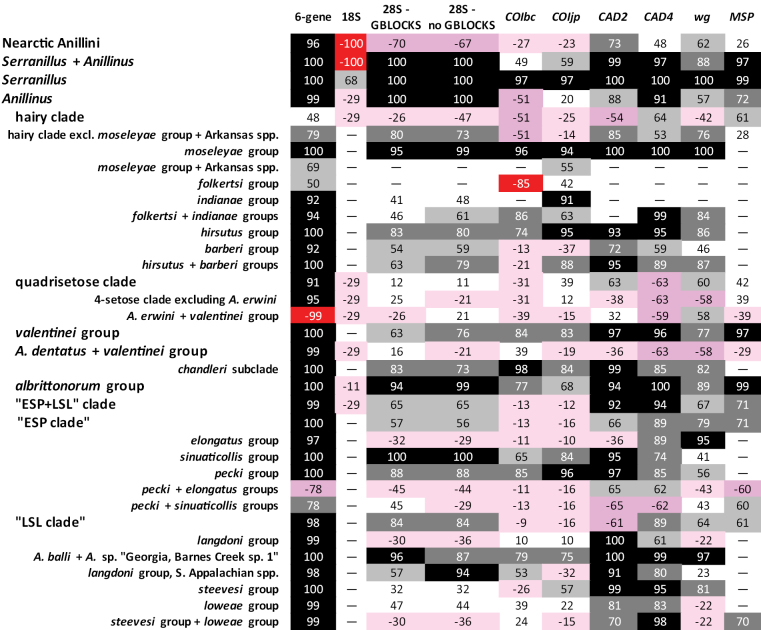
Support for and against clades of Anillini. Black = clade present in ML tree, SBS 90 or greater, Dark grey = clade present in ML tree, SBS between 70 and 89, light grey = clade present in ML tree, SBS between 50 and 69, white = clade present in ML tree, SBS less than 50. Red = clade absent in ML tree, highest SBS of contradictory clade greater than 80, dark pink = clade absent in ML tree, highest SBS of contradictory clade between 50 and 69, light pink = clade absent in ML tree, highest SBS of contradictory clade less than 50. “–” indicates insufficient data.

**Figure 5.**
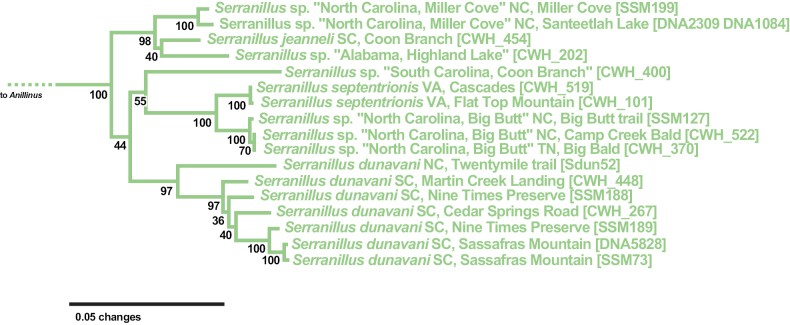
Maximum likelihood tree of *Serranillus*, from 6-gene concatenated core matrix. SBS values shown below nodes.

The maximum likelihood tree from the 6-gene concatenated core matrix (Fig. 3) found a well-supported Nearctic Anillini clade (standard bootstrap support [SBS] 96%; Fig. 4), in which *Anillinus* and *Serranillus* are well supported as sister to each other, and together as sister to the two western Nearctic genera sampled, *Anillodes* Jeannel and *Medusapyga* LaBonte & Maddison. Monophyly of *Serranillus* is supported by all genes sampled, with SBS values 97% or greater except in 18S (SBS 68%). All *Serranillus* sampled have several shared unique insertions in 28S, the longest of which is 11 (in *S.dunavani*) or 12 bp (in other species) in length. In our 6-gene tree, three strongly supported clades (SBS 97% or greater) within *Serranillus* are present (Fig. 5): *S.jeanneli* and two undescribed species from North Carolina and Alabama (SBS 98%), the northern *Serranillusseptentrionis* Sokolov & Carlton and an undescribed species from North Carolina and Tennessee (SBS 100%), and *S.dunavani* (SBS 97%). The lone female of *Serranillus* sp. “South Carolina, Coon Branch” is recovered as sister to the northern *S.septentrionis* clade in the 6-gene (SBS 55%), COIjp (SBS 53%), CAD4 (SBS 96%) and MSP (SBS 72%) trees. In the 6-gene tree, the widespread *S.dunavani* is sister to the clade containing *S.* sp. “South Carolina, Coon Branch” and the *S.septentrionis* clade, but with low support (SBS 44%).

**Figure 6.**
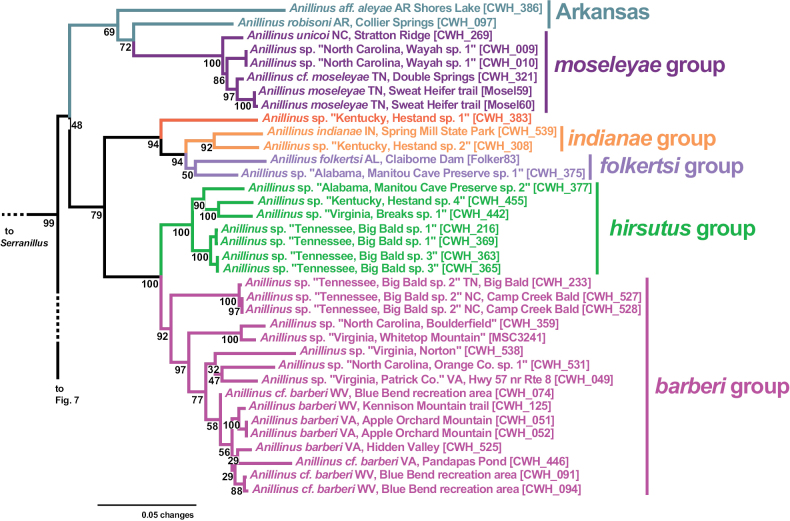
Maximum likelihood tree of the “hairy clade” of *Anillinus*, from 6-gene concatenated core matrix. SBS values shown below nodes.

*Anillinus* is recovered as sister to *Serranillus* in all genes sampled except 18S, and as monophyletic in all except 18S and COIbc. Support for the monophyly of *Anillinus* is strong in 28S (SBS 100% with and without GBLOCKS) and CAD4 (SBS 91%), moderately strong in CAD2 (SBS 88%), Wg (SBS 57%), and MSP (SBS 72%), with weaker support from COIjp (SBS 20%). In our 6-gene phylogeny, the sampled species of *Anillinus* are divided into two clades that are largely consistent with the setation of the right paramere: a “hairy clade” in which the apical setae form a dense brush and a “quadrisetose clade” in which four apical setae are present. Bootstrap support for the “hairy clade” in the 6-gene tree is low (SBS 48%), and the clade is present in only two single-gene trees, CAD4 (SBS 64%) and MSP (SBS 61%). In our 6-gene tree, the “hairy clade” species are split into two main clades that are better supported (Fig. 6), one with the Appalachian high elevation endemic *moseleyae* group and the Arkansas species (SBS 69%), and the other with the remaining species (SBS 79%). The latter clade contains two well-supported clades: one with the *indianae* group, *folkertsi* group, and the isolated species *Anillinus* sp. “Kentucky, Hestand sp. 1” (SBS 94%) and the other with the *hirsutus* group and *barberi* group (SBS 100%).

**Figure 7.**
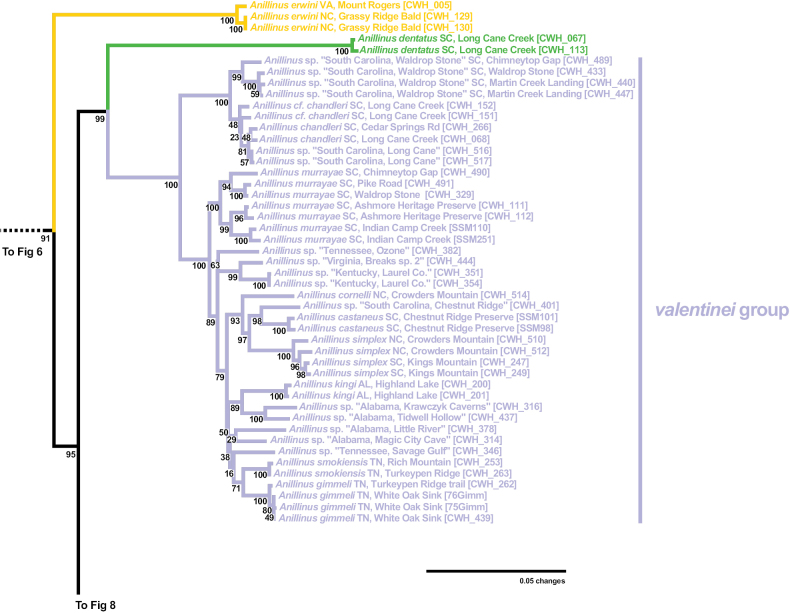
Maximum likelihood tree of portion of “quadrisetose clade” of *Anillinus*, from 6-gene concatenated core matrix. SBS values shown below nodes.

The “quadrisetose clade” of *Anillinus* is recovered in the 6-gene tree (SBS 91%), 28S with (SBS 12%) and without GBLOCKS (SBS 11%), COIjp (SBS 39%), CAD2 (SBS 63%), Wg (SBS 60%), and MSP (SBS 42%). The topology of the “quadrisetose clade” in the 6-gene tree (Fig. 3) is well-resolved, with eight strongly supported species groups (SBS 97% or greater) and three isolated individual taxa. The isolated species *Anillinuserwini* is recovered as sister to the remaining species (SBS 95%), a relationship also recovered in 28S with GBLOCKS (SBS 25%) and MSP (SBS 39%) trees. *Anillinuserwini* is sister to the *valentinei* group in 28S without GBLOCKS (SBS 21%), CAD2 (SBS 32%) and Wg (58%) trees. The remaining single gene trees recover unique placements of *A.erwini*: sister to the *A.dentatus*+*valentinei* group clade (COIbc), sister to *A.dentatus* alone (18S and COIjp), and sister to the “hairy clade” (CAD4), all with low support. The “quadrisetose clade” species besides *A.erwini* are divided into two well-supported clades in the 6-gene tree, one containing *A.dentatus* and the *valentinei* group (SBS 99%) and the other containing the *albrittonorum* group and the “ESP+LSL” clade (SBS 95%). The former clade recovers the isolated species *A.dentatus* as sister to the *valentinei* group (SBS 100%) (Fig. 7), a diverse and widespread lineage of mostly low-elevation species. The *valentinei* group is recovered as a clade in all of the genes sampled, with moderate to strong support in all (SBS 63% or greater). In contrast, the sister relationship between *A.dentatus* and the *valentinei* group is present only in 28S with GBLOCKS (SBS 16%) and COIbc trees (SBS 39%). Placement of *A.dentatus* varies between the single gene trees: sister to *A.erwini* (18S and COIjp), sister to the “ESP+LSL” clade (28S without GBLOCKS), sister to *A.erwini*+*valentinei* group (CAD2), sister to all other *Anillinus* species (CAD4), sister to the “quadrisetose clade” except *A.erwini* (MSP), and sister to the *albrittonorum* group (Wg). The *albrittonorum* group, consisting of two isolated species in South Carolina (*A.jancae*) and Florida (*A.albrittonorum*) is present in all gene trees except 18S, with SBS values of 68 or greater. The *albrittonorum* group is recovered as sister to the “ESP+LSL” clade in the 6-gene (SBS 95%), CAD2 (SBS 65%), CAD4 (SBS 82%), and MSP (SBS 22%) trees. Placement of the *albrittonorum* group varies in the remaining single gene trees: sister to “quadrisetose clade” except *A.erwini* (28S with GBLOCKS), sister to all “quadrisetose clade” *Anillinus* (28S without GBLOCKS), sister to the *moseleyae* group (COIbc), and sister to the *sinuaticollis* group (COIjp), all with low support.

**Figure 8.**
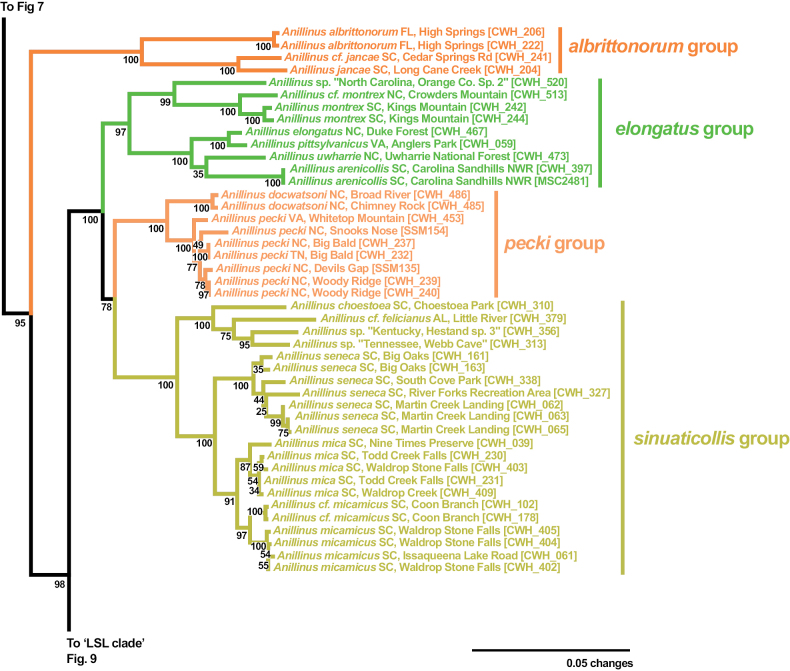
Maximum likelihood tree of *albrittonorum* group and “ESP clade” of *Anillinus*, from 6-gene concatenated core matrix. SBS values shown below nodes.

The “ESP” and “LSL” clades are sister to each other in the 6-gene tree (SBS 99%) and all single gene trees except 18S, COIbc, and COIjp, with moderate support in 28S (SBS 65% with and without GBLOCKS), Wg (SBS 67%), and MSP (SBS 71%), and strong support in CAD2 (SBS 92%), CAD4 (SBS 94%). The “ESP” clade (Fig. 8), consisting of the *elongatus* group, *sinuaticollis* group, and *pecki* group is present in all gene trees except COIbc and COIjp, and is strongly supported by the 6-gene tree (SBS 100%), and moderately supported by CAD2 (SBS 66%), CAD4 (SBS 89%), Wg (SBS 79%), and MSP (SBS 71%), with weaker support in 28S with (SBS 57%) and without (SBS 56%) GBLOCKS. The *sinuaticollis* group and *pecki* group are both recovered as clades in all genes sampled, with moderate to strong support from most. The *elongatus* group is strongly supported as a clade in the 6-gene tree (SBS 97%), but is present only in the CAD4 (SBS 89%) and Wg (SBS 95%) individual gene trees. There are two consistently recovered subclades in the *elongatus* group, one containing *A.montrex* and an undescribed North Carolina species, and the other containing the remaining species. These two clades are paraphyletic to each other in 28S (with and without GBLOCKS), COIbc, and CAD2 and polyphyletic to each other in COIjp. Only one individual of the *elongatus* group was sampled for 18S and MSP.

**Figure 9.**
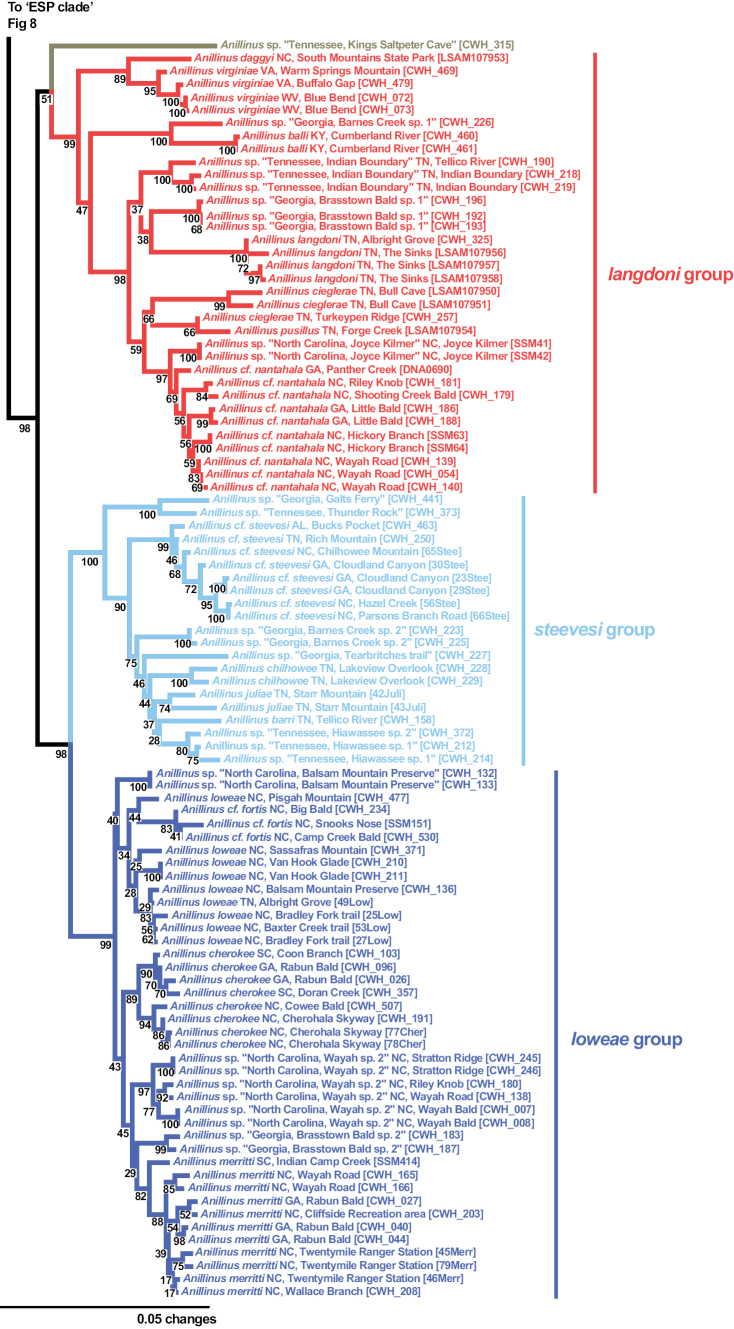
Maximum likelihood tree of “LSL clade” of *Anillinus*, from 6-gene concatenated core matrix. SBS values shown below nodes.

The “LSL” clade (Fig. 9), consisting of the *langdoni* group, *steevesi* group, and *loweae* group, is recovered in all genes except COIbc, COIjp, and CAD2, with strong support in the 6-gene (SBS 98%) and CAD4 (SBS 89%) trees, moderate support in 28S with and without GBLOCKS (SBS 84%), Wg (SBS 64%), and MSP (SBS 61%). The *langdoni* group is recovered in the 6-gene (SBS 99%), COIbc (SBS 10%), COIjp (SBS 10%), CAD2 (SBS 100%), and CAD4 (SBS 61%) trees. Three clades are consistently recovered in the *langdoni* group: the northern *Anillinusvirginiae* (and *A.daggyi* in COI trees), the Kentucky endemic *Anillinusballi* and an undescribed Georgia species, and the remaining southern Appalachian endemic species. The latter clade is absent from the COIjp tree, but otherwise the three clades are recovered in all genes, with moderate to strong support from most. The isolated species *Anillinus* sp. “Tennessee, Kings Saltpeter Cave” is recovered as sister to the *langdoni* group in the 6-gene (SBS 51%) and MSP (SBS 98%) trees, but its placement varies within the remaining genes. Its inclusion in the “LSL” clade is supported by the 6-gene (SBS 98%), CAD4 (SBS 89%), Wg (SBS 64%) and MSP (SBS 61%) trees. The *steevesi* group is present in 6-gene (SBS 100%), 28S with and without GBLOCKS (SBS 32%), COIjp (SBS 57%), CAD2 (SBS 99%), CAD4 (SBS 95%), and Wg (SBS 81%) trees. Only a single individual was included in 18S and MSP analyses. The *loweae* group is strongly supported in 6-gene (SBS 99%), CAD2 (SBS 81%) and CAD4 (SBS 83%), and weakly supported in 28S with (SBS 47%) and without GBLOCKS (SBS 44%), COIbc (SBS 39%), and COIjp (SBS 22%). In the Wg tree, the *loweae* group is polyphyletic, with some individuals of *A.merritti* and *A.* sp. “North Carolina Wayah sp. 2” sister to the Southern Appalachian *langdoni*-group species and the remaining individuals paraphyletic at the base of a clade including *A.balli* and its sister species and the *steevesi* group. The *steevesi* group and *loweae* group are strongly supported as sister clades in the 6-gene (SBS 98%) and CAD4 (SBS 98%) trees, moderately supported as sister in the MSP (SBS 70%) and CAD2 (SBS 70%) trees, and weakly supported as sister in the COIbc tree (SBS 24%). The species we include in the *steevesi* group were formerly included in the *loweae* group (Sokolov and Carlton 2010, Sokolov 2011), but we find the *steevesi* group to be monophyletic with respect to the *loweae* group in all genes except COIbc, in which two undescribed *steevesi*-group species from Tennessee and Georgia are recovered as sister to the *loweae* group.

Complete trees from all Maximum Likelihood and Standard Bootstrap analyses are shown in Suppl. material 1.

### ﻿﻿Systematics of Appalachian *Anillinus*

#### ﻿﻿Key to eastern genera of Anillini and most species groups of *Anillinus*

Precise identification of *Anillinus* and *Serranillus* specimens requires examination of the male genitalia, and genitalic characters could not be avoided in the key below. The key below will aid with initial sorting of an unknown specimen, but species-level identifications must be made by comparison of genitalia with descriptions or authoritatively identified specimens.

**Table:** 

1	Males; first protarsomere enlarged and with thick adhesive setae ventrally (Fig. 10), last abdominal ventrite with two fixed setae on posterior margin	**2**
–	Females; first protarsomere not enlarged and without thick adhesive setae ventrally, last abdominal ventrite with four fixed setae on posterior margin	**23**
2(1)	Last abdominal ventrite “serrate”, with three blunt lobes on posterior margin (Fig. 15B); medial setae of mentum on tooth (Fig. 11A), left mandible with retinacular tooth (Fig. 11C)	***Serranillus***
–	Last abdominal ventrite not “serrate”, without blunt lobes on posterior margin; medial setae of mentum set basad to tooth (Fig. 11B), left mandible without retinacular tooth (Fig. 11D) (*Anillinus*)	**3**
3(2)	Metafemur with angulate tooth or blunt spine on posterior margin (Fig. 12A, E, F)	**4**
–	Metafemur with posterior margin evenly rounded, at most with medial field of coarse microsculpture (Fig. 12B, C, D)	**12**
4(3)	Only first protarsomere with ventral adhesive setae, second unmodified (Fig. 10D, E, F)	**5**
–	Both first and second protarsomeres with ventral adhesive setae (Fig. 10G, H, I)	**10**
5(4)	Right paramere of aedeagus with more than eight apical setae	**6**
–	Right paramere of aedeagus nearly always with four apical setae, rarely with three or six	**7**
6(5)	Flagellum of internal sac long, filamentous; median lobe of aedeagus almost entirely membranous dorsally; range: central Tennessee, Kentucky, Indiana	***indianae* group**
–	Flagellum of internal sac short, thick; median lobe of aedeagus with sclerotized section apically, demarcated by a distinct notch; range: eastern Tennessee, North Carolina, Virginia, West Virginia	***barberi* group (in part)**
7(5)	Body strongly flattened dorsoventrally, with additional leg modifications, either profemora or mesotrochanters spinose	**8**
–	Body at least moderately convex, without additional leg modifications	**9**
8(7)	Profemora with prominent spine on posterior face, mesotrochanters simple; second abdominal ventrite with median keel; large, ABL > 2 mm	***albrittonorum* group (in part; *A.jancae* sp. nov.)**
–	Profemora simple, mesotrochanters spinose; second abdominal ventrite without median keel; smaller, ABL < 2 mm	***dentatus* group**
9(7)	Flagellum of aedeagus long and filamentous, often protruding beyond ostium; range: high elevations in mountains north of French Broad River	***erwini* group**
–	Flagellum of aedeagus short, not protruding beyond ostium; range: low elevations in Piedmont, south and west of Appalachian Mountains	***sinuaticollis* group (in part)**
10(4)	Body strongly flattened dorsoventrally, flagellum of aedeagus long and filamentous; range: Piedmont of Virginia, North Carolina, and South Carolina OR south-central Kentucky	**11**
–	Body moderately convex and ovoid, flagellum of aedeagus short and thick; range: mountains of northwestern Virginia and northern West Virginia	***langdoni* group (in part; *A.virginiae*)**
11(10)	Head with frontoclypeal horn reduced, nearly absent; median lobe of aedeagus with a saddle-like sclerite beneath flagellum; range: south-central Kentucky	***Anillinus* sp. “Kentucky, Hestand Sp. 1**”
–	Head with frontoclypeal horn well-developed and conspicuous; median lobe of aedeagus lacking saddle-like sclerite beneath flagellum; range: Piedmont of Virginia, North Carolina, and South Carolina	***elongatus* group**
12(3)	Only first protarsomere with ventral adhesive setae, second protarsomere unmodified	**13**
–	Both first and second protarsomeres with ventral adhesive setae	**17**
13(12)	Right paramere with more than eight apical setae	**14**
–	Right paramere with four apical setae (three in rare individuals)	**15**
14(13)	Dorsal margin of median lobe of aedeagus with prominent notch where membranous ostium meets apical dorsal sclerotized portion; femora not densely setose	***barberi* group (in part)**
–	Dorsal margin of median lobe of aedeagus without notch; femora densely setose or not	***hirsutus* group**
15(13)	Flagellum of median lobe short, not filamentous apically	***sinuaticollis* group (in part)**
–	Flagellum of median lobe long, filamentous apically in most species	**16**
16(15)	Metafemur swollen posteriorly; body narrower; range: high elevations in mountains northeast of French Broad River	***erwini* group**
–	Metafemur simple; body broader; range: low to mid elevations, western edge of Great Smoky and Unicoi mountains, west to northern Mississippi	***steevesi* group (in part)**
17(12)	Right paramere with dense brush of apical setae; left paramere with five or more poriferous canals on ventral margin	***folkertsi* group**
–	Right paramere with four apical setae; left paramere with four or less poriferous canals on ventral margin	**18**
18(17)	Walls of median lobe of aedeagus heavily sclerotized, left side with abruptly membranous section	***loweae* group**
–	Walls of median lobe less heavily sclerotized, left side without abruptly membranous section	**19**
19(18)	Internal sac of aedeagus with prominent sclerotized spines or scales	**20**
–	Internal sac of aedeagus without prominent sclerotized spines or scale	**21**
20(19)	Flagellum of internal sac well-sclerotized and elongate	***valentinei* group (in part)**
–	Flagellum of internal sac lightly sclerotized and short	***pecki* group (in part; *A.docwatsoni*)**
21(19)	Dorsal surface of head with microsculpture effaced in part, absent at least from sides of vertex	**22**
–	Dorsal surface of head entirely covered with microsculpture	***langdoni* group (in part)**
22(21)	Flagellum weakly sclerotized and short, appearing W-shaped in lateral aspect; range: mountains northeast of French Broad River	***pecki* group (in part; *A.pecki*)**
–	Flagellum well-sclerotized, shape various but never W-shaped in lateral aspect; range: lower elevations south and west of southern Appalachian Mountains southwest of French Broad River	**23**
23(22)	Apex of median lobe small and rounded	***valentinei* group (in part)**
–	Apex of median lobe modified, broadly rounded with ventral margin greatly expanded or with dorsal margin deeply excavated	***steevesi* group (in part)**
24(1)	Paramedial setae of mentum on mentum tooth (Fig. 11A); spermatheca short and curved, gradually enlarged apically (Fig. 21S, T)	***Serranillus***
–	Paramedial setae of mentum basad of tooth (Fig. 11B); spermatheca variable, if short and curved then abruptly enlarged apically (Fig. 21N) (*Anillinus*)	**25**
25(24)	Spermathecal duct coiled	**26**
–	Spermathecal duct not coiled	**31**
26(25)	Spermatheca shorter, not S- or 2-shaped	**27**
–	Spermatheca longer, with S- or 2 shape	**29**
27(26)	Spermatheca with smooth stem, abruptly enlarged apically	***albrittonorum* group (in part; *A.jancae* sp. nov.)**
–	Spermatheca with annulated stem, more gradually enlarged apically	**28**
28(27)	Body robust and broad, head relatively larger; range: southern Tennessee north to southern Indiana	***indianae* group**
–	Body narrower, head relatively smaller; range: central Tennessee south to southern Alabama	***folkertsi* group**
29(26)	Body strongly dorsoventrally flattened and parallel sided	**30**
–	Body at least moderately convex and ovoid	***steevesi* group OR *valentinei* group**
30(29)	Stem of spermatheca nearly straight, without basal bend; frontoclypeal horn absent	***dentatus* group**
–	Stem of spermatheca more sinuate, with basal bend; frontoclypeal horn present	***elongatus* group**
31(25)	Spermathecal duct greatly reduced, not apparent	***valentinei* group (in part)**
–	Spermathecal duct variable in length, but long enough to be apparent	**32**
32(31)	Stem of spermatheca rough, “ribbed” in part	**33**
–	Stem of spermatheca entirely smooth	**36**
33(32)	Base of spermatheca swollen at basal bend	**34**
–	Base of spermatheca not swollen	**35**
34(33)	Spermatheca long; range: high elevations in Southern Appalachians	***moseleyae* group**
–	Spermatheca short; range: south-central Kentucky	***Anillinus* sp. “Kentucky, Hestand Sp. 1**”
35(33)	Spermatheca with basal bend smoothly rounded	***loweae* group (in part)**
–	Spermatheca with basal bend angulate	***pecki* group**
36(32)	Pronotum entirely covered in microsculpture	**37**
–	Pronotum with microsculpture at least partially effaced on disc	**39**
37(36)	Body dorsoventrally flattened	**38**
–	Body convex, ovoid	***loweae* group (in part)**
38(37)	Spermathecal duct short, evenly curved; metafemora simple.	***barberi* group OR *hirsutus* group**
–	Spermathecal duct longer, not curved; metafemora with tooth on posterior margin	***Anillinus* sp. “South Carolina, Wateree**”
39(36)	Stem of spermatheca looped over itself proximally; endogean species south and west of southern Appalachians	***sinuaticollis* group**
–	Stem of spermatheca not looped over itself proximally	**40**
40(39)	Spermatheca short, stem shorter than enlarged apex; endogean species in South Carolina and Florida	***albrittonorum* group (in part)**
–	Spermatheca longer, stem longer than enlarged apex	**41**
41(40)	Elytra broadest at humeri; range: low elevations west of Appalachian Mountains in northeastern Tennessee	***Anillinus* sp. “Tennessee, Kings Saltpeter Cave**”
–	Elytra broadest approximately middle; range: high elevations in mountains northeast of French Broad River	***erwini* group**

### ﻿﻿Species groups of Appalachian *Anillinus*

Below, we list the species groups supported by both DNA sequence data and morphology, and provide diagnoses for each. We summarize available data on distribution and habitat use for each group.

Suppl. material 3 includes a checklist of all eastern Nearctic Anillini species, including those not sampled for our phylogeny, with their hypothetical placement within this new systematic arrangement. The dataset of Harden (2024) includes full locality and deposition data for all specimens studied, including undescribed species not treated in this work.

#### ﻿﻿‘*moseleyae* group’

“group VII endogean species” Sokolov et al. 2004: 228, in part.

**Diagnosis.** Dorsal microsculpture largely lacking from head and pronotum. Pronotum strongly constricted posteriorly. Elytra long and narrow (Fig. 2B). Male protarsomeres 1 and 2 both dilated and bearing ventral adhesive vestiture. Median lobe of aedeagus with left side bearing a patch of long setae, right paramere with dense brush of apical setae. Spermatheca long, with ribbed stem and short spermathecal duct. Humeri of females usually strongly sloped (not so in *A.unicoi*).

**Diversity.** Three described species (Sokolov 2011) and one undescribed (“North Carolina, Wayah sp. 1” in our phylogeny).

**Distribution and habitat.** All of the *moseleyae*-group species are restricted to elevations above 4,500ft in the mountains southwest of the French Broad River; specimens are known from the Great Smoky, Plott Balsam, Unicoi, Snowbird, and Nantahala Mountains (Fig. 42B). Their apparent absence from the Great Balsam Mountains is notable. In the Great Smoky Mountains, *Anillinuscarltoni* Sokolov, *Anillinusmoseleyae* Sokolov & Carlton, and *Anillinusunicoi* Sokolov have been collected in series by sifting leaf litter (Sokolov 2011; NCSU data). At lower elevations in the Unicoi, Snowbird, and Nantahala Mountains, specimens have been collected from endogean habitats using pipe traps or by searching under embedded rocks.

#### ﻿﻿*Anillinus* sp. “Kentucky, Hestand sp. 1”

**Diagnosis.** Members of this phylogenetically isolated species (Fig. 2P) possess the following unique set of character states: head entirely microsculptured, disc of pronotum lacking microsculpture, male protarsomeres 1 and 2 expanded and with ventral adhesive setae, male metafemora swollen with large triangular tooth on posterior margin, internal sac of aedeagus with large saddle-like sclerite beneath flagellum, flagellum long and filamentous, left side of median lobe channelized near apex, right paramere with four setae and two or three additional pores without setae, left paramere with seven pores on ventral margin, female spermatheca small and S-shaped, base thick, stem ribbed, duct not coiled.

**Distribution and habitat.** This species is known only from a single stream hollow in Hestand, Kentucky (Fig. 42B). Specimens have been collected using pipe traps, soil extraction, and hand collecting underneath deeply embedded rocks.

**Notes.** A description of this species is in progress, based on specimens in the CUAC and CWHc collections.

#### ﻿﻿‘*indianae* group’

“group I litter species” Sokolov et al. 2004: 228, in part.

**Diagnosis.** Dorsal microsculpture fully developed on head and pronotum. Body broad and dorsoventrally flattened (Fig. 2Q). Males with one or two protarsomeres with ventral adhesive setae. Male metafemora swollen and angularly produced posteriorly in middle, bearing a sharp tooth or not. Dorsal margin of median lobe membranous except basal 1/4, apex of median lobe large and concave on left side, not channelized. Saddle-like sclerite present in internal sac near sclerotized ostial plate on left side, flagellum long and filamentous. Right paramere with dense brush of apical setae. Left paramere with many pores on ventral margin but without setae. Spermatheca (examined only in *A.indianae*) small and weakly ribbed, gradually enlarged distally, with short proximal bend, spermathecal duct long and loosely coiled.

**Diversity.** This group includes the described species *Anillinusindianae* Jeannel and *Anillinuslongiceps* Jeannel, as well as two undescribed species from Kentucky.

**Distribution and habitat.** The collective range of the group extends from the vicinity of Sewanee, Tennessee north to Lawrence Co., Indiana (Fig. 42B). These species inhabit deep soils, MSS and caves.

#### ﻿﻿‘*folkertsi* group’

“group I litter species” Sokolov et al. 2004: 228, in part.

**Diagnosis.** Dorsal microsculpture fully developed on head and pronotum. Body dorsoventrally flattened, head relatively small (Fig. 2E). Males with protarsomeres 1 and 2 with ventral adhesive setae. Male metafemora unmodified. Median lobe with dorsal margin more extensively sclerotized than in *indianae* group, apex broad, left side channelized. Internal sac with long filamentous flagellum that is coiled apically and an ostial saddle-like sclerite on left side. Right paramere with apical setae forming one or two dense brushes. Left paramere with eight or more short setae on ventral margin. Spermatheca short and weakly ribbed, spermathecal duct long and coiled.

**Diversity.** Two described species, *Anillinusfolkertsi* Sokolov & Carlton and *Anillinusfolkertsioides* Sokolov, and two undescribed species from Alabama and Tennessee comprise this group.

**Distribution and habitat.** The range of this group extends from southern Alabama to central Tennessee (Fig. 42B). Specimens have been extracted from leaf litter on the surface and in caves (Sokolov et al. 2004; Sokolov 2021), and collected from underneath embedded rocks.

#### ﻿﻿‘*barberi* group’

“group I litter species” Sokolov et al. 2004: 228, in part.

“*virginiae* group” Sokolov et al. 2007: 4, in part.

**Diagnosis.** Dorsal microsculpture fully developed on head and pronotum in most species, sculpticels on pronotum relatively small and dense. Body dorsoventrally flattened (Fig. 2R). Males with protarsomere 1 expanded and bearing ventral adhesive setae, male protarsomere 2 unmodified. Male leg modifications variable but metafemora always modified, either greatly swollen and bowed anteriorly with straight posterior margin or evenly swollen with posterior margin slightly angulate medially. Median lobe of aedeagus almost entirely membranous dorsally, only base and apex sclerotized. Apical dorsal sclerotized plate of median lobe variable in form but always abruptly set off posteriorly from dorsal ostium by abrupt notch. Left side of median lobe not channelized in most species. Flagellum heavily sclerotized and short, rotated dorsally and curved, internal sac scaly or spined. Right paramere with dense brush of apical setae. Left paramere with many scattered pores on ventral margin, some bearing short setae. Spermatheca long, with stem smooth except short ribbed region proximally, spermathecal duct short and evenly curved.

**Diversity.** One described species, *Anillinusbarberi* Jeannel, and seven undescribed species comprise this group.

**Distribution and habitat.***Anillinusbarberi* occurs in Virginia, Maryland, and West Virginia. The undescribed species occur in Virginia, North Carolina, and Tennessee (Fig. 42C). Specimens of the *barberi* group have been primarily collected from endogean habitats using pipe traps or searching under embedded rocks. A small number of specimens have been collected from sifted leaf litter.

#### ﻿﻿‘*hirsutus* group’

“*kovariki* group” Sokolov 2012: 65, in part.

**Diagnosis.** Members of this group are large (Fig. 2S) and share most of the character states of the *barberi* group, differing in the following: male femora and trochanters of all legs densely setose in most species, dorsal margin of aedeagus less irregularly structured, with ostium not separated from apex by sclerotized apical portion, internal sac usually without scales or spines.

**Diversity.** Three described species (*Anillinusclinei* Sokolov, *Anillinushildebrandti* Sokolov, and *Anillinushirsutus* Sokolov) and 10 undescribed species comprise this group.

**Distribution and habitat.** Members of the *hirsutus* group range from the Cumberland Plateau in extreme southwest Virginia and central Kentucky south to northern Alabama and Georgia (Fig. 42C). The only high elevation occurrence of the group is on Big Bald on the North Carolina-Tennessee border in the Bald Mountains, where two undescribed species have been collected from leaf litter and underneath embedded rocks. All other species are known only from endogean or cave habitats.

**Notes.** The right paramere of *A.hirsutus* was described and illustrated as having only four setae (Sokolov 2012). Our examination of three male paratypes found that the right paramere actually bears a dense brush of apical setae. *Anillinuscavicola* Sokolov is similar in some characters to members of the *hirsutus* group, especially the thick and dorsally rotated flagellum of the aedeagus, but for now we place *A.cavicola* in its own species group along with a closely similar undescribed species (Suppl. material 3).

#### ﻿﻿‘*erwini* group’

“group VI litter species” Sokolov et al. 2004: 228.

**Diagnosis.** Dorsal microsculpture weakly impressed and difficult to trace on most of head and pronotum. Body relatively narrow and elongate, slightly depressed dorsoventrally (Fig. 2K). Males with protarsomere 1 enlarged and bearing ventral adhesive setae, male protarsomere 2 unmodified and without ventral setae. Male metafemora swollen and slightly angulate medially on posterior margin. Median lobe of aedeagus slightly twisted dorsally. Flagellum elongate and filamentous. Right paramere quadrisetose. Left paramere with three or four ventral setae. Spermatheca long and smooth, spermathecal duct long with a few loose coils medially.

**Diversity.** This group consists of only *Anillinuserwini* Sokolov & Carlton.

**Distribution and habitat.***Anillinuserwini* is a high elevation endemic that ranges from Mount Mitchell in the Black Mountains north to the Mount Rogers vicinity in southwestern Virginia, including Roan Mountain and Grandfather Mountain, but apparently absent from the Great Craggy and Bald Mountains (Fig. 43B). Specimens have been collected from leaf litter and underneath embedded rocks.

#### ﻿﻿‘*dentatus* group’

**Diagnosis**. Body small (ABL = 1.49–1.69), dorsoventrally flattened (Fig. 2C). Dorsal microsculpture fully developed on head and pronotum. Head with frontoclypeal horn inconspicuous. Males with protarsomere 1 expanded and with adhesive setae ventrally, second protarsomere unmodified and without ventral setae. Male mesotrochanters spinose ventrally. Male metafemora swollen and with a triangular tooth on posterior margin near middle. Median lobe of aedeagus nearly straight, with dorsal margin largely unsclerotized. Flagellum long and coiled over itself in a proximal loop in repose. Right paramere quadrisetose. Left paramere asetose with four ventral pores. Stem of spermatheca straight at proximal end, without abrupt angulation, spermathecal duct long and evenly coiled several times.

**Diversity.** Only *Anillinusdentatus* sp. nov. belongs to this group.

**Distribution and habitat.***Anillinusdentatus* is known only from two localities within a small area near Long Cane Creek in Sumter National Forest, Abbeville Co., SC (Fig. 43B). One specimen was collected by Berlese extraction of sifted dead wood, but all other specimens were collected from endogean habitats using pipe traps, soil washing, and searching under embedded rocks.

#### ﻿﻿‘*valentinei* group’ sensu novo

“group VIII litter species” Sokolov et al. 2004: 228, in part.

“a group of ovoid species with partially microsculptured head” Sokolov and Carlton 2008: 44, in part.

“*valentinei* group” Sokolov 2011: 12; Sokolov 2012: 69, in part.

“*barri* group” Sokolov 2012: 66, in part.

**Diagnosis.** Dorsal microsculpture effaced from most of head and pronotum in most species, always absent from sides of vertex. Males with protarsomeres 1 and 2 both with adhesive setae ventrally in most species. Median lobe of aedeagus often with a carinate channel on left side near base. Flagellum well-sclerotized, internal sac often with various other well sclerotized structures such as spines or ostial plates. Right paramere quadrisetose. Left paramere with four pores on ventral margin, bearing setae or not. Spermatheca variable, long or short, with smooth or ribbed stem, spermathecal duct long and coiled or short.

**Diversity.** This is the most speciose group of *Anillinus*, with eight previously described species (*Anillinuschandleri* Sokolov, *Anillinuscornelli* Sokolov & Carlton, *Anillinusgimmeli* Sokolov & Carlton, *Anillinushumicolus* Sokolov, *Anillinuskingi* Sokolov, *Anillinusmurrayae* Sokolov & Carlton, *Anillinussmokiensis* Sokolov, and *Anillinusvalentinei* (Jeannel)) and two species from South Carolina described as new below. More than 20 additional species are known and will be described in future papers.

**Distribution and habitat.** The range of the group extends from western North Carolina to northern Alabama, north to southeastern Kentucky and adjacent southwestern Virginia (Fig. 43B). The group is apparently absent from most of northern Georgia, and with the exception of *A.murrayae*, members of this group do not inhabit montane habitats in the Appalachians, and are restricted to lower elevations. Specimens have been collected from every microhabitat from which Appalachian anillines are known to occur: shallow leaf litter, deep soils, dead wood, and caves. *Anillinusvalentinei* (sensu Sokolov 2012) is the only species of *Anillinus* that has been repeatedly collected in large series from caves, and according to Barr (1969) the species “exhibits the ecology and behavior of a troglobite.”

**Note.**Sokolov (2012) placed *Anillinustombarri* Sokolov in this group, based on its large body size, presumably troglobitic habits (the lone specimen was collected in a cave), and the presence of spines in the internal sac. The type of *A.tombarri* has numerous character states that indicate it is not closely related to this clade: second protarsomere without ventral adhesive setae, right paramere with more than four apical setae, dorsal margin of median lobe almost entirely membranous, metafemora modified. The thick, strongly curved flagellum of *A.tombarri* is similar to that of all members of the *hirsutus* and *barberi* groups, but for now we leave it in its own distinct group (Suppl. material 3).

#### ﻿﻿‘*albrittonorum* group’

“group VII endogean species” Sokolov and Schnepp 2021: 40, in part.

**Diagnosis.** The two species known to belong to this lineage are quite different in external morphology and most male genitalic characters, but share the following: male protarsomere 1 expanded and with ventral adhesive setae, protarsomere 2 unmodified and without ventral setae, flagellum of median lobe of aedeagus lightly sclerotized and filamentous, spermatheca short and abruptly enlarged distally, with long spermathecal duct.

**Diversity.** This group consists of *Anillinusalbrittonorum* Sokolov & Schnepp and *Anillinusjancae* sp. nov., described below.

**Distribution and habitat.***Anillinusalbrittonorum* inhabits deep sand in northern Florida, where it has only been collected using passive traps (Sokolov and Schnepp 2021). *Anillinusjancae* sp. nov. lives in deep red clay soils in South Carolina, and has been collected using pipe traps and by turning deeply embedded rocks.

#### ﻿﻿‘*elongatus* group’ sensu Harden and Caterino (2024)

“group V endogean species” Sokolov et al. 2004: 228, in part.

“*sinuaticollis* group” Sokolov 2012: 62, in part.

**Diagnosis.** This group includes the flattest and narrowest species of *Anillinus* in the eastern U.S., with parallel-sided elytra and relatively large heads (Fig. 2H). Development of dorsal microsculpture varies from fully developed on head and pronotum to lacking on most of pronotum and sides of vertex. Males have protarsomeres 1 and 2 expanded laterally on inner margin and both bear adhesive vestiture ventrally. Metafemora of males are modified, swollen apically with posterior margin tuberculate and in some species bearing a prominent peg-like tooth. The median lobe of the aedeagus is slightly twisted dorsally. Flagellum lightly sclerotized and open laterally, long and filamentous distally. Right paramere quadrisetose. Left paramere with four preapical pores, bearing setae or not. Spermatheca elongate and moderately sinuate or straight, with surface either smooth or ribbed, spermathecal duct long and coiled.

**Diversity.** This group includes five described species (*Anillinusarenicollis*, *Anillinuselongatus* Jeannel, *Anillinusmontrex*, *Anillinuspittsylvanicus*, and *Anillinusuwharrie*), and two undescribed species (Harden and Caterino 2024).

**Distribution and habitat.** This group is distributed in the Piedmont ecoregion from Virginia, North Carolina, and northeastern South Carolina (Fig. 43D). Members of this group are endogean, and have been collected using pipe traps, soil washing, soil extraction, and underneath embedded rocks. One specimen of *A.arenicollis* was collected in a litter sample taken in February.

#### ﻿﻿‘*sinuaticollis* group’ sensu novo

“group V endogean species” Sokolov et al. 2004: 228, in part.

“*sinuaticollis*-group” Sokolov 2012: 62, in part.

**Diagnosis.** Relatively small in size (ABL less than 2 mm). Dorsal microsculpture usually present on entire dorsal surface of head, absent from disc of pronotum in most species. Males with protarsomere 1 expanded and bearing ventral adhesive setae, male protarsomere 2 not expanded and not bearing ventral adhesive setae. Flagellum of median lobe lightly sclerotized, open laterally. Internal sac with or without other sclerotized structures. Stem of spermatheca long and coiled proximally, spermathecal duct short and without coils.

**Diversity.** This group includes two previously described species (*Anillinussinuaticollis* Jeannel and *Anillinusfelicianus* Sokolov). Four new species from South Carolina are described below. Two undescribed species are known and will be described in future papers.

**Distribution and habitat.** This group occupies three disjunct regions: the Cumberland Plateau from Kentucky south to northern Alabama, southeastern Louisiana, and the inner Piedmont and Blue Ridge of South Carolina (Fig. 43D). The apparent absence of this group from Georgia is notable. Members of this group are endogean and have been collected using pipe traps, soil washing, searching under embedded rocks, and from caves.

#### ﻿﻿‘*pecki* group’ sensu Harden and Caterino (2024)

“group II litter species” Sokolov et al. 2004: 228, in part.

**Diagnosis.** Dorsal microsculpture variable, weakly impressed on head and pronotum in most specimens. Body moderately convex and ovoid (Fig. 2F). Male protarsomeres 1 and 2 expanded and bearing ventral adhesive setae. Male metafemora unmodified except for patch of coarse microsculpture on posterior margin. Median lobe twisted dorsally, flagellum of median lobe short and lightly sclerotized, open laterally. Left side of internal sac with small scales of variable sclerotization. Spermatheca long with stem strongly ribbed medially and angulate at base, spermathecal duct short and not coiled.

**Diversity.** This group consists of *Anillinuspecki* Giachino and *Anillinusdocwatsoni* Sokolov & Carlton.

**Distribution and habitat.***Anillinuspecki* is widespread in the southern Appalachians north of the French Broad River, ranging from the Black Mountains in western North Carolina to Whitetop Mountain in southwest Virginia, whereas *Anillinusdocwatsoni* is a micro-range endemic known only from the Hickory Nut Gorge in Rutherford and Henderson Counties, North Carolina (Fig. 43D). *Anillinuspecki* has been collected from leaf litter and underneath embedded rocks. Most specimens of *A.docwatsoni* have been collected under embedded rocks (Harden and Caterino 2024), but a small number have been collected from flood debris.

#### ﻿﻿*Anillinus* sp. “Tennessee, Kings Saltpeter Cave”

**Diagnosis.** The single specimen of this unique lineage, a female, has an ABL of 2.09 mm. Dorsal microsculpture effaced from most of pronotum and head. Three supraorbital setae present on each side of head. Elytra are widest at the base, where the humeri are strongly produced (Fig. 2I). Spermatheca long and S-shaped, with a short spermathecal duct. Males of the species are unknown.

**Distribution and habitat.** The specimen was collected in a cave in northeastern Tennessee (Fig. 43E).

#### ﻿﻿‘*langdoni* group’ sensu novo

“group I litter species” Sokolov et al. 2004: 228, in part.

“group II litter species” Sokolov et al. 2004: 228, in part.

“*virginiae* group” Sokolov et al. 2007: 4, in part.

“*langdoni* group” Sokolov et al. 2007: 4, in part.

**Diagnosis.** Dorsal microsculpture fully developed on head and pronotum in most specimens, sculpticels relatively large. Male protarsomeres 1 and 2 expanded and with ventral adhesive setae in most species. Male metafemora unmodified in all species except *A.virginiae*, in which they are swollen and bear a blunt angulate projection on posterior margin. Median lobe twisted dorsally. Flagellum thick, variable in length and degree of sclerotization. Both parameres quadrisetose. Spermatheca short, spermathecal duct variable in length, not coiled.

**Diversity.** In the broad sense that we adopt here, six described species are assigned to this group (*A.balli*, *Anillinuscieglerae* Sokolov & Carlton, *Anillinuslangdoni* Sokolov & Carlton, *Anillinusnantahala* Dajoz, *Anillinuspusillus* Sokolov & Carlton, and *Anillinusvirginiae* Jeannel). Four undescribed species are known, and will be described in future papers.

**Distribution and habitat.** Members of this group are found in four disjunct geographic regions: southern Appalachian Mountains (*A.cieglerae*, *A.langdoni*, *A.nantahala*, *A.pusillus*, and the four undescribed species), South Mountains of western North Carolina (*A.daggyi*), northwestern Virginia and adjacent West Virginia (*A.virginiae*), and eastern Cumberland Plateau of Kentucky (*A.balli*) (Fig. 43E). The southern montane species are readily collected from leaf litter, while most specimens of *A.virginiae* have been collected from endogean habitats using pipe traps and searching under embedded rocks.

**Notes.**Giachino (2011) described *Anillinuscampbelli* as a member of the *langdoni* group. An examination of photos of the holotype, including the genitalia, suggests that *A.campbelli* is conspecific with *A.cherokee* of the *loweae* group, which is abundant near the type locality of *A.campbelli* (Van Hook Glade, Macon Co., NC).

#### ﻿﻿‘*loweae* group’ sensu novo

“group II litter species” Sokolov et al. 2004: 228 in part.

“a group of ovoid species with partially microsculptured head” Sokolov and Carlton 2008: 44, in part.

“*loweae* group” Sokolov and Carlton 2010: 2, in part.

**Diagnosis.** In most members of this group the dorsal microsculpture is well developed on the forebody, absent only from a small area on either side of the head near the base. Males have adhesive vestiture present ventrally on both protarsomeres 1 and 2. Male profemora often strongly swollen. Median lobe twisted dorsally, with well-defined ostium of variable shape on left side. Internal sac with well-sclerotized flagellum of variable length, never filamentous. Right paramere quadrisetose. Left paramere with four preapical pores, bearing setae or not. Spermatheca long and well-sclerotized, gradually enlarged distally, stem smooth or ribbed, spermathecal duct short and uncoiled.

**Diversity.** This species group includes four described species (*Anillinuscherokee* Sokolov & Carlton, *Anillinusfortis* (Horn), *Anillinusloweae* Sokolov & Carlton, and *Anillinusmerritti* Sokolov & Carlton) and three undescribed species that will be treated in a future paper.

**Distribution and habitat.** The range of this group encompasses most of the southern Appalachians from the Bald Mountains on the Tennessee-North Carolina border south to northern South Carolina and Georgia (Fig. 43G). Members of this group are strictly montane and most species can be readily collected in leaf litter. *Anillinusmerritti* is primarily endogean, and most specimens have been collected under rocks and using pipe traps.

**Notes.** The type of *A.fortis* has not been critically studied by modern workers (Sokolov et al. 2004), and the concept of the late T.C. Barr encompassed three species (*A.erwini*, *A.fortis* sensu Sokolov and Carlton 2010, and *A.pecki*). Barr’s concept informed the concept of later workers (Sokolov et al. 2004, Sokolov and Carlton 2010). To indicate the uncertainty of the identity of the type of *A.fortis*, we refer to the species as “A.cf.fortis” in our phylogeny.

#### ﻿﻿‘*steevesi* group’

“group II litter species” Sokolov et al. 2004: 228, in part.

“group VIII litter species” Sokolov et al. 2004: 228, in part.

“a group of ovoid species with partially microsculptured head” Sokolov and Carlton 2008: 44, in part.

“*loweae* group” Sokolov and Carlton 2010: 2, in part.

“*barri* group” Sokolov 2012: 66, in part.

**Diagnosis.** Dorsal microsculpture effaced from most of head and pronotum in most species. Males of most species with only protarsomere 1 expanded and bearing adhesive setae ventrally. Median lobe twisted dorsally, left side without well-defined ostial opening. Flagellum well-sclerotized and variable in length, filamentous in some species. Right paramere quadrisetose. Left paramere with four preapical pores, bearing setae or not. Spermatheca similar to that of the *loweae* group, but differing in having the spermathecal duct long and heavily coiled (the female genitalia of only three species have been examined, however).

**Diversity.** Five described species belong to this group (*Anillinusbarri* Sokolov & Carlton, *Anillinuschilhowee* Sokolov, *Anillinusinexpectatus* Sokolov, *Anillinusjuliae* Sokolov & Carlton, and *Anillinussteevesi* Barr), and eight undescribed species are known.

**Distribution and habitat.** This group is a dominant component of the anilline fauna of northern Georgia and adjacent Tennessee, where numerous short-range undescribed species are known. *Anillinussteevesi* has the largest range of any *Anillinus*, ranging from far western North Carolina across northern Alabama and into northeastern Mississippi (Fig. 43F). *Anillinussteevesi* has been collected from leaf litter, under rocks, and in caves. The other species in the group have been collected in leaf litter and under rocks. Members of this group occur at lower elevations than the *loweae* group.

**Notes.** The holotype of *A.steevesi* was not found at CMNH, and has probably been lost or was never designated by Barr. To indicate the uncertainty of the type status, we refer to the species as “A.cf.steevesi” in our phylogeny.

### ﻿﻿Taxonomic review of Anillini of South Carolina

#### ﻿﻿Tribe Anillini

Taxon classificationAnimaliaColeopteraCarabidae

Jeannel, 1937

BBC14D0C-7264-55AF-897F-A0512B8BD60D

##### Adult diagnosis.

In the United States, adult specimens of Anillini are the only carabids that are both eyeless and possess subulate palpomeres.

##### Larval diagnosis.

Late-instar larvae of Anillini in South Carolina share the following characters: body pale and soft, largely unsclerotized (Fig. 13A, B); legs with single tarsal claw; stemmata absent; coronal suture absent; retinaculum large; penicillus present and consisting of several setae; urogomphus with seven large setae (Fig. 14A, E). Other known carabid larvae in SC of similar size with a single tarsal claw and potentially lacking stemmata are the genera *Trechus*, *Semiardistomis*, *Clivina*, and the tribe Tachyini. Larvae of *Trechus* are easily recognized by the possession of 4-segmented labial palps and 5-segmented maxillary palps (versus 2- and 4-segmented, respectively, in anillines). *Semiardistomis* and *Clivina* can be readily separated by the small size of the retinaculum and a penicillus consisting of a single, large seta. South Carolina anilline larvae are similar to larvae of the tribe Tachyini, but differ by having seven large setae on the urogomphus (Fig. 14E), versus six in tachyines.

#### ﻿﻿ Serranillus

Taxon classificationAnimaliaColeopteraCarabidae

Barr, 1995: 246

6CBBE87B-2041-575E-9A84-3108EE9842D6

##### Adult diagnosis.

Males of *Serranillus* are easily distinguished from *Anillinus* by the “serrate” modification of the last abdominal ventrite, which bears three protruding blunt lobes (Figs 15B, 18B, 19B), and by the greatly reduced and inconspicuous right paramere which lacks setae (Figs 15D, 19D). The last abdominal ventrite of male *Serranillus* also has large lateral extensions internally, visible when the abdomen is removed and cleared (Fig. 20C). The dorsal body surfaces of *Serranillus* are typically more setose than members of *Anillinus*, particularly the elytra (Sokolov et al. 2004), but members of some *Anillinus* species are similarly setose (Sokolov 2021). The pair of median setae on the mentum are on the mentum tooth itself in all specimens of *Serranillus* examined by us (Fig. 11A), while in *Anillinus* they are typically basad the tooth (Fig. 11B). Both mandibles in *Serranillus* possess a retinacular tooth near the base (Fig. 11C), while in *Anillinus* only the right mandible has a tooth (Fig. 11D). In addition to the reduced size of the right paramere, the right basal lobe of the median lobe of the aedeagus in *Serranillus* is reduced to a thin strip. The internal sac of the aedeagus in all species also contains a coiled sclerite on the left side, which is absent in *Anillinus*. The female spermathecae of *Serranillus* species are relatively small and have a stem that is nearly straight before the curved apex (Fig. 21S, T). The spermathecae are similar in shape to those of *A.albrittonorum* and *A jancae*, but the stem in those species is more slender and curved outward from the base (Fig. 21N).

##### Larval diagnosis.

Differing from the single known late-instar *Anillinus* larva by mandibles with terebrae lacking serrations (Fig. 14B) and stipes of maxilla with setae of group gMX unevenly placed (Fig. 14D).

##### Diversity.

Four previously described species, one species described as new below, and at least 10 undescribed species (Suppl. material 3).

##### Distribution.

Few occurrence records have been previously published for *Serranillus*. The type locality of *Serranillusdunavani* (Jeannel) is Sassafras Mountain, near Rocky Bottom in Pickens Co, South Carolina, and the species has also been reported from Great Smoky Mountains National Park (Sokolov and Carlton 2008, 2010). Barr (1995) described *Serranillusjeanneli* from Coweeta Hydrologic Laboratory in Macon County, North Carolina, and stated the range extended from the Great Balsam Mountains in western North Carolina to northern Georgia, but gave no further details. He also mentioned in passing the existence of additional species in Cloudland Canyon (Dade County, GA) and the Piedmont of North Carolina and South Carolina. The Cloudland Canyon species is currently interpreted as *Serranillusmagnus* (Zaballos & Mateu, 1997), described from material mislabeled as from Brazil (Sokolov and Carlton 2012). Sokolov and Carlton (2008) described *Serranillusseptentrionis* from southwestern Virginia and cite a record of *S.jeanneli* from White County, Georgia. Museum records and personal collecting provide a more complete view of the range of the genus (Fig. 1A). Collectively, the genus occupies most of the southern Appalachians from NC to northwest GA, with disjunct occurrences in southwestern VA, the Black and Bald Mountains of NC and TN, central and western Alabama, and the Cumberland Plateau of northern Tennessee. A pair of female *Serranillus* in the CMNH collection bearing Connecticut locality labels is mislabeled according to the collector (S. Peck, pers. comm., July 2019).

### ﻿﻿Partial key to the species of *Serranillus* of South Carolina and adjacent parts of North Carolina and Georgia

Note. Females of *Serranillus* are currently impossible to identify to species without associated males. Therefore, females are not separated in the key below. Confirmation of identifications should always be made by examining male genitalia.

**Table:** 

1	Posterior margin of last abdominal ventrite modified, with three blunt projections; first and second protarsomeres expanded and bearing ventral adhesive setae (males)	**2**
–	Posterior margin of last abdominal ventrite unmodified; first and second protarsomeres unmodified (females, not treated further)
2(1)	Smaller, ABL = 1.79–2.03 mm; dorsal and ventral margins of median lobe in right dorsolateral aspect straight and parallel-sided for most of length before abruptly enlarged apex (Fig. 15E)	***S.dunavani***
–	Larger, ABL = 1.99–2.87 mm; margins of median lobe in right dorsolateral aspect not parallel-sided or straight	**3**
3(2)	Lobes of last abdominal ventrite with outer pair narrower and more prominent than inner lobe (Fig. 18B); body smaller, ABL = 1.99–2.35 mm	**4**
–	Lobes of last abdominal ventrite subequal in width and prominence, or with inner lobe slightly more prominent than outer pair; body larger, ABL = 2.48–2.87 mm	**5**
4(3)	Ventral surface of median lobe of aedeagus with carinate shelf near apex, causing ventral margin to appear deeply notched by a semicircular channel (Fig. 18D); left side of internal sac with long, dark spine that is bluntly hooked apically where it protrudes beyond ostium	***S.jeanneli***
–	Ventral surface of median lobe without carinate shelf, ventral margin without deep semicircular channel; left side of internal sac with short, broad claw-shaped sclerite that is acutely pointed apically and ends well before ostium	***S.* sp. “North Carolina, Miller Cove**”
5(3)	Hind angles of pronotum strongly projecting posteriorly (Fig. 19A)	***S.monadnock* sp. nov.**
–	Hind angles of pronotum not strongly projecting posteriorly (as in Fig. 18A)	**6**
6(5)	Apex of median lobe produced ventrally, dorsal margin of median lobe channelized	***S.* sp. “Georgia, Rabun Bald sp. 1**”
–	Apex of median lobe not produced ventrally, dorsal margin of median lobe not channelized	**7**
7(6)	Ventral margin of median lobe with central tuft of long setae; ventral margin not notched	***S.* sp. “North Carolina, Riley Knob**”
–	Ventral margin of median lobe without central tuft of long setae; ventral margin notched	***S.* sp. “Georgia, Rabun Bald sp. 3**”

#### ﻿﻿ Serranillus dunavani

Taxon classificationAnimaliaColeopteraCarabidae

(Jeannel, 1963)

CAFD8E51-DABE-5C9F-AA34-247C19BEB571

Figs 2A , 10G , 11A, C , 13A , 14B, D , 15A–E , 16 , 17A , 21S , 25A–C

Anillinus dunavani Jeannel, 1963: 76; Barr 1995: 245.
Serranillus dunavani (Jeannel): Sokolov et al. 2004: 188.

##### Material examined.

***Holotype male* (USNM)**: point mounted, not dissected, labeled: “Rocky Bottom, Pickens Co. S.C., 25 Aug. 1932, D. Dunavan Co.” “Sassafras Mtn. 3,500 ft” “In leaf mold” “TYPE [red paper]” “Type No. 69542 USNM [red paper]” “Anillinusdunavani n.sp. R. Jeannel det. 19/”.

**Figure 10.**
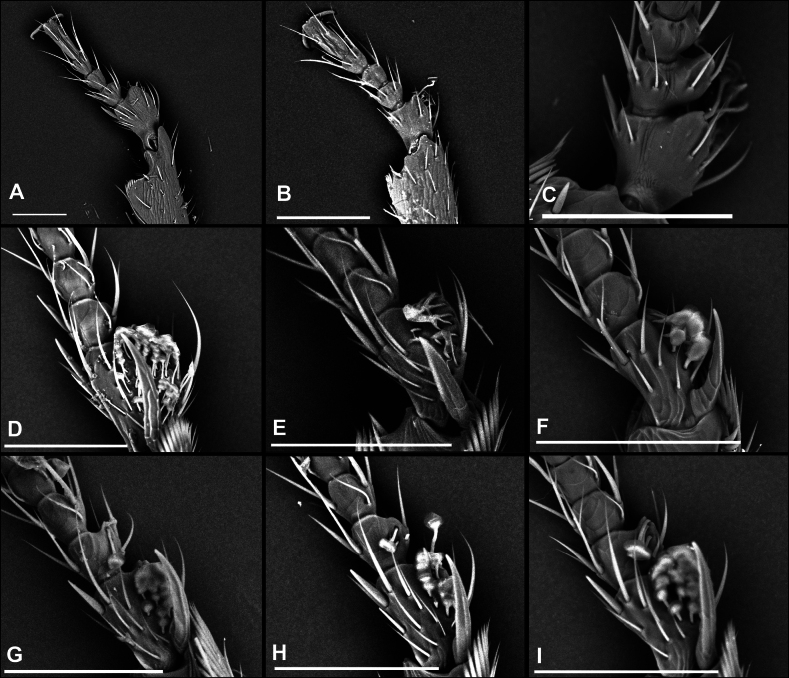
SEM micrographs of male protarsi **A***Anillinusjancae*, left protarsus, dorsal aspect **B***Anillinusmica*, left protarsus, dorsal aspect **C***Anillinusmontrex* left protarsus, dorsal aspect **D***A.jancae*, right protarsus, ventral aspect **E***Anillinuschoestoea*, right protarsus, ventral aspect **F***Anillinusdentatus*, right protarsus, ventral aspect **G***Serranillusdunavani* (Jeannel), right protarsus, ventral aspect **H***Anillinusmurrayae* Sokolov & Carlton, right protarsus, ventral aspect **I***A.montrex*, right protarsus, ventral aspect. Scale bars: 0.1 mm.

**Figure 11.**
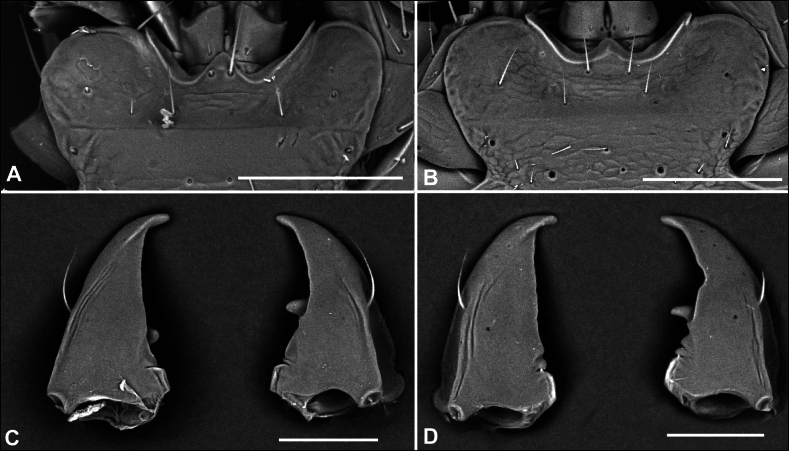
SEM micrographs of mouthparts of Appalachian Anillini genera **A** mentum of *Serranillusdunavani* (Jeannel) **B** mentum of *Anillinuschoestoea***C** mandibles of *S.dunavani*, dorsal aspect **D** mandibles of *A.choestoea*, dorsal aspect. Scale bars: 0.1 mm.

##### Other material

**(*n* = 177, CMNH, CUAC, CWHc, NCSU, OSUC, USNM). USA** • **North Carolina** • 1 ♂; **Clay Co**.; Nantahala National Forest, Shooting Creek Bald; 35.0679, -83.6466; 11 May 2020; C.W. Harden and M.S. Caterino leg.; CWH-381, CUAC000066851; • 1 ♂, 4 ♀; same data as previous; CWHc; • 1 ♂, 1 ♀; **Clay Co.**; Nantahala National Forest, Shooting Creek Bald; 35.0681, -83.6464; 11 May 2020; C.W. Harden leg.; CWHc; • 2 ♀; **Henderson Co.**; 0.3 miles southwest of Bat Cave; 35.4470, -82.2910; 22 July 1967; S. Peck and A. Fiske leg.; CMNH; • 2 ♂, 1 ♀; **Macon Co.**; 1 mile northwest of Highlands; 35.061, -83.217; 16 Aug. 1981; Q.D. Wheeler leg.; OSUC; • 1 ♂, 1 ♀; **Macon Co.**; Nantahala National Forest, Cowee Bald; 35.3269, -83.3350; 15 Sep. 2020; • 1 ♂; **Montgomery Co.**; Uwarrie National Forest, 2 miles south of Eldorado, Route 109; 35.4340, -80.0190; 27 Aug. 1990; W. Reeves leg.; LSAM0153884; NCSU; • 2 ♂, 1 ♀; **Polk Co.**; Green River Game Lands; 35.2940, -82.2610; 18 Mar. 2018; M.S. Caterino leg.; CUAC000107846, CUAC000107847, CUAC000107856; • 1 ♀; **Polk Co.**; Lower Bradley Falls Trail; 35.3580, -82.2878; 19 Mar. 2018; M.S. Caterino leg.; CUAC000107772; • 2 ♂, 2 ♀; **Polk Co.**; Melrose Falls; 35.2217, -82.2985; 10 Aug. 2021; M.S. Caterino leg.; • 1 unsexed; **Swain Co.**; Great Smoky Mountains National Park, Deep Creek area, loop trail at Sunkota Ridge trail; 35.4750, -83.4200; 25 Jul. 2002; C.E. Carlton leg.; NCSU_ENT00293747; NCSU; • 1 unsexed; **Swain Co.**; Great Smoky Mountains National Park, Lakeshore Trail; 35.4520, -83.5410; 18 Jul. 2018; A.K. Tishechkin leg.; NCSU_ENT00293744; NCSU; • 1 ♂, 1 ♀; **Transylvania Co.**; Sassafras Mountain; 35.0656, -82.7776; 11 Jun. 2020; CUAC; • 1 ♂, 2 ♀; **Transylvania Co.**; 1 mile south of Rosman, NC 178; 35.1290, -82.8230; [no date]; J.F. and S. Cornell leg.; NCSU; • **South Carolina**; • 1 ♂; **Abbeville Co.**; Sumter National Forest, Long Cane Creek; 34.1345, -82.3230; 4 Feb. 2022; M. Ferro leg.; CWHc; • 1 ♀; **Abbeville Co.**; Sumter National Forest, off Cedar Springs Road; 34.1086, -82.3390; 25 Sep. 2020; C.W. Harden leg.; CWH-267, CUAC000066830; • 1 ♂; **Greenville Co.**; Highway 97, River Falls Lodge; 35.1221, -82.5405; 18 Mar. 2017; M. Ferro leg.; CUAC000049982; • 1 ♂, 11 ♀; **Greenville Co.**; Ashmore Heritage Preserve; 35.0867, -82.5788; 14 Mar. 2020; C.W. Harden and L.M. Thompson leg.; CWHc; • 1 ♂; **Greenville Co.**; Ashmore Heritage Preserve; 35.0933, -82.5930; 29 Jun. 2015; S. Myers leg.; MSC-2463, CUAC000185783; • 1 ♂; **Greenville Co.**; Chestnut Ridge Heritage Preserve; 35.1506, -82.2779; 8 Apr. 2018; M. Caterino and L. Vasquez leg.; CUAC000108123; • 1 ♀; **Greenville Co.**; Chestnut Ridge Heritage Preserve; 35.1507, -82.2821; 20 Oct. 2021; C.W. Harden leg.; CWHc; • *n* = 11; **Kershaw Co.**; English Swamp, Wateree Floodland Memorial Forest; 34.0911, -80.6578; 27 Feb. 2010; J.F. Cornell, S. Cornell and B. Gregory leg.; NCSU; • 2 ♀; **Oconee Co.**; Coon Branch Natural Area; 35.0200, -83.0000; 21 Jun. 2018; D. Chandler leg.; CUAC000109930 and CUAC000109964; • 4 ♂, 2 ♀; **Oconee Co.**; Coon Branch Natural Area; 35.0170, -82.9970; 18 Oct. 2020; C.W. Harden leg.; CWHc; • 1 ♂; **Oconee Co.**; Devils Fork State Park; 34.9390, -82.8798; 26 Apr. 2015; S. Myers leg.; MSC-2454, CUAC000185791; • 1 ♂, 2 ♀; **Oconee Co.**; East Fork [Chattooga River]; 34.9843, -83.0981; 4 May 2015; M.S. Caterino and S. Myers leg.; CUAC000110650, CUAC000110651, CUAC000110653; • 1 ♀; **Oconee Co.**; East Fork trail, Ellicott Rock Wilderness; 34.9913, -83.0980; 29 Jun. 2015; S. Myers leg.; SSM250, CUAC000185792; • 1 unsexed; **Oconee Co.**; East Fork trail; 34.9838, -83.0979; 4 May. 2015; S. Myers leg.; SSM415, CUAC000185793; • 2 ♂, 1 ♀; **Oconee Co.**; Hill above parking lot near Stumphouse Tunnel; 34.8097, -83.1235; 5 Jul. 2019; C.W. Harden leg.; CWHc; • 1 ♂; **Oconee Co.**; Martin Creek Landing; 34.6389, -82.8663; 26 May 2022; C.W. Harden leg.; CWH-448, CUAC000066843; • 1 ♀; **Oconee Co.**; Oconee State Park, 6.7 miles north-northwest of Walhalla; 34.8690, -83.1050; 1 Jul. 1983; J. Pakaluk leg.; NCSU; • 2 ♂, 5 ♀; **Oconee Co.**; Oconee State Park; 34.8690, -83.1050; 21 Jul. 1967; S. Peck and A. Fiske leg.; CMNH; • 1 ♂, 1 ♀; **Oconee Co.**; Sumter National Forest, Yellow Branch Falls; 34.8067, -83.1283; 12 Oct. 2017; M.S. Caterino leg.; CUAC000107624 and CUAC000107625; • 3 ♂, 1 ♀; **Oconee Co.**; Sumter National Forest, Forest service road 725; 34.8395, -83.1677; 13 Sep. 2020; A. Deczynski leg.; CWHc; • 1 ♂; **Oconee Co.**; Sumter National Forest, Indian Camp Branch; 34.9898, -83.0724; 4 May 2015; M.S. Caterino and S. Myers leg.; CUAC000010470; • 2 ♂, 2 ♀; **Oconee Co.**; Sumter National Forest, Tater Hill trail; 34.9612, -83.0116; 24 Feb. 2018; M. Caterino leg.; CUAC; • 1 ♂; **Pickens Co.**; Chimneytop Gap; 35.0620, -82.8008; 10 Jun. 2015; S. Myers leg.; MSC-2457, CUAC000185780; • 1 ♂; **Pickens Co.**; Chimneytop Gap; 35.0632, -82.7964; 23 Mar. 2023; C.W. Harden leg.; CWHc; • 1 ♂, 1 ♀; **Pickens Co.**; Chimneytop Gap; 35.0630, -82.7968; 23 Mar. 2023; C.W. Harden leg.; CWHc; • 1 ♂; **Pickens Co.**; Clemson Experimental Forest; 34.7384, -82.8436; 20 Feb. 2023; E. Recuero leg.; CWHc; • 1 ♀; **Pickens Co.**; Clemson Experimental Forest, Waldrop Stone area; 34.7390, -82.8216; 2 Feb. 2021; C.W. Harden leg.; CWHc; • 1 ♂; **Pickens Co.**; Clemson Experimental Forest, Wildcat Creek; 34.7561, -82.8551; 20 Jun. 2018; C.W. Harden leg.; CWHc; • 3 ♂, 1 ♀; **Pickens Co.**; Clemson; 34.6820, -82.8330; 4 Oct. 1966; J.A. Payne leg.; CMNH; • 1 ♀; **Pickens Co.**; Clemson; 34.6820, -82.8330; 19 May 1962; J.A. Payne leg.; CMNH; • 4 ♂, 2 ♀; **Pickens Co.**; Clemson; 34.6820, -82.8330; 20 Jun. 1962; J.A. Payne leg.; CMNH; • 1 ♂; **Pickens Co.**; Eastatoe Creek Heritage Preserve; 35.1577, -82.4910; 30 Mar. 2015; S. Myers leg.; MSC-2455, CUAC000185778; • 1 ♂; **Pickens Co.**; Eastatoe Creek Heritage Preserve; [incorrect coordinates]; 30 Mar. 2015; S. Myers leg.; MSC-2461, CUAC000185781; • 1 ♀; **Pickens Co.**; Eastatoe Heritage Preserve; 35.0462, -82.8178; 6 Apr. 2014; M. Caterino and K. Caterino leg.; CUAC000002709; • 2 ♂, 1 ♀; **Pickens Co.**; near Chimneytop Gap; 35.0639, -82.7996; 11 Sep. 2019; M.S. Caterino leg.; CUAC; • 4 ♀; **Pickens Co.**; Nine Times Preserve, 34.9460, -82.8023; 31 Sep. 2015; S. Myers leg.; SSM184 to SSM187, CUAC000185770, CUAC000185773 to CUAC000185775; • 2 ♂, 1 ♀; **Pickens Co.**; Nine Times Preserve; 34.9465, -82.7992; 27 Mar. 2015; S. Myers leg.; SSM188 to SSM190; CUAC000185769, CUAC000185776, CUAC000185777; • 3 ♂, 1 ♀; **Pickens Co.**; Nine Times Preserve; 34.9464, -82.8027; 27 Mar. 2015; M.S. Caterino and S. Myers leg.; CUAC000173553, CUAC000173556, CUAC000173557, CUAC000173559; • 1 larva, 1 ♂; **Pickens Co.**; Sassafras Mountain; 35.0640, -82.7767; 20 Oct. 2020; F. Etzler and P. Wooden leg.; CWH-311 and CWH-312, CUAC000185899 and CUAC000066867; • 8 ♂, 9 ♀; same data as previous; CUAC; • 2 ♀; **Pickens Co.**; Sassafras Mountain; 35.0645, -82.7774; 10 Jun. 2015; S. Myers leg.; SSM67 and SSM68, CUAC000185771 and CUAC000185785; • 2 ♀; **Pickens Co.**; Sassafras Mountain; 35.0634, -82.7760; 10 Jun. 2015; S. Myers leg.; SSM69 and SSM70, CUAC000185784 and CUAC000185790; • 3 ♂, 1 ♀; **Pickens Co.**; Sassafras Mountain; 35.0579, -82.7705; 10 Jun. 2015; S. Myers leg.; SSM71 to SSM74, CUAC000185786 to CUAC000185789; • 3 ♂, 5 ♀; **Pickens Co.**; Sassafras Mountain; 35.0647, -82.7774; 11 Jun. 2020; F. Etzler leg.; CUAC; • 5 ♂, 4 ♀; **Pickens Co.**; Sassafras Mountain; 35.0650, -82.7750; 21 Jul. 1967; S. Peck and A. Fiske leg.; CMNH.

**Figure 12.**
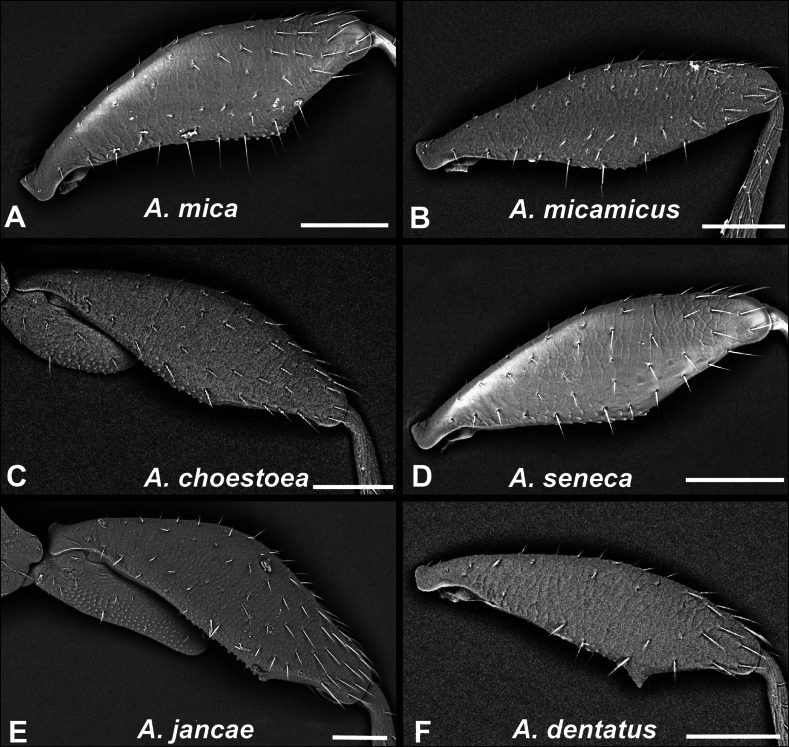
SEM micrographs of male left metafemora of *Anillinus* species. Scale bar: 0.1 mm.

**Figure 13.**
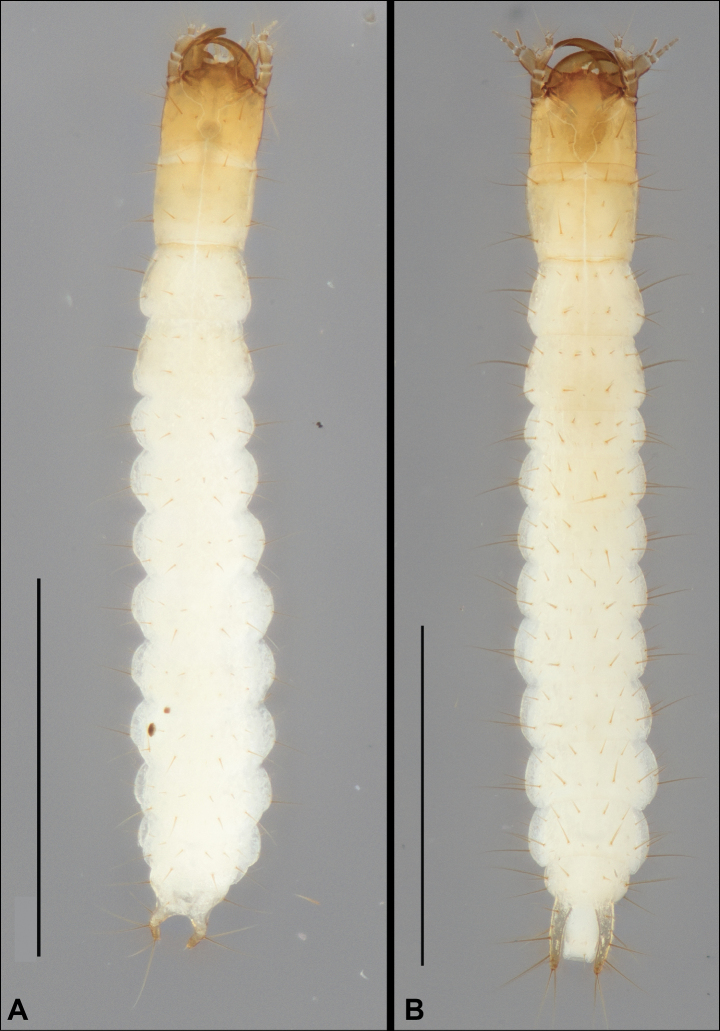
Dorsal habitus of late-instar larvae **A***Serranillusdunavani* (Jeannel) **B***Anillinusjancae*. Scale bars: 1 mm.

**Figure 14.**
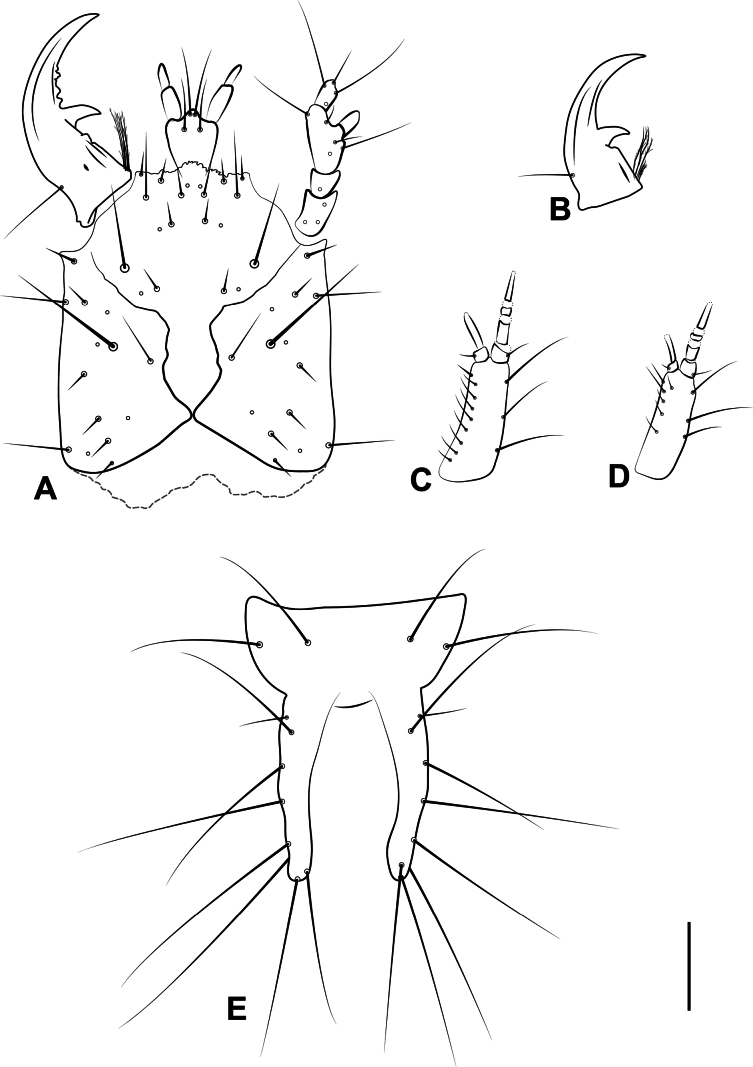
Details of late-instar larvae of Appalachian Anillini**A** head of *Anillinusjancae* sp. nov., dorsal aspect, left antenna and right mandible omitted **B** left mandible of *Serranillusdunavani* (Jeannel), dorsal aspect **C** right maxilla of *A.jancae*, dorsal aspect **D** right maxilla of *S.dunavani*, dorsal aspect **E** urogomphi of *A.jancae*, dorsal aspect. Scale bar: 0.1 mm.

GenBank accession numbers for specimens from Sassafras Mountain: OR839609, OR839610, OR839814, OR839815, OR839816, OR839817, OR839818, OR838294, OR853396, OR839363, OR839819, OR837946, OR838111, OR838295.

##### Literature records.

Besides the type locality, no specific records have been previously published for *S.dunavani*. Sokolov and Carlton (2010) cite a molecular voucher of *S.dunavani* from “GSMNP, Twentymile Trail.” Sokolov and Carlton (2008) state that they studied material of *S.dunavani* only from “GSMNP”. Their fig. 7 shows the range of *S.dunavani* covering over 30 counties in Tennessee, North Carolina, and South Carolina, citing “original data” as the source for the figure. However, these data are not apparently available and we have not seen any material of *S.dunavani* from Tennessee nor from most of the North Carolina counties that are shaded in that figure.

##### Diagnosis.

**Figure 15.**
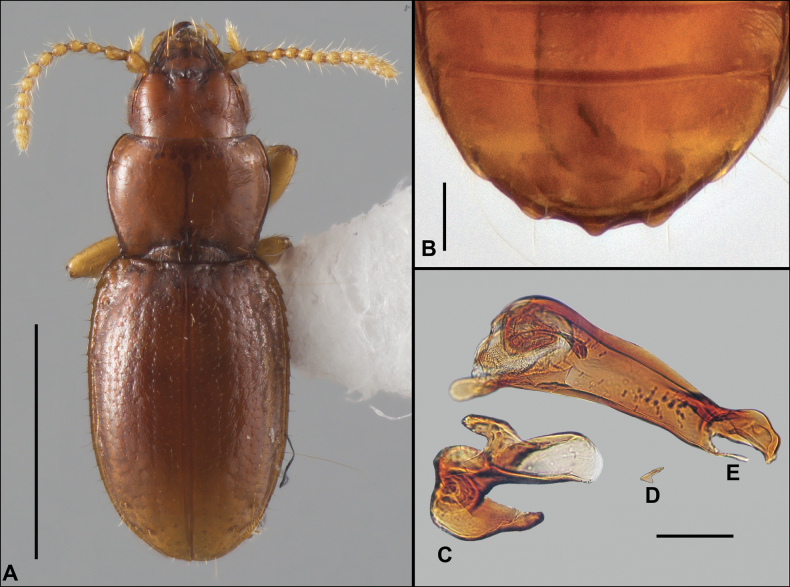
*Serranillusdunavani* (Jeannel) **A** habitus **B** last abdominal ventrite, ventral aspect **C** left paramere, left lateral aspect **D** right paramere, right lateral aspect **E** median lobe, right dorsolateral aspect. Scale bars: 1 mm (**A**); 0.1 mm (**B–E**).

This is the smallest *Serranillus* species, with most specimens having an ABL of 2 mm or less. The median lobe of the aedeagus is distinctive in being elongate with a recurved apex and lacking large, sclerotized spines in the internal sac (Fig. 15E).

##### Redescription.

***Habitus*** Small for the genus (ABL = 1.79–2.03 mm), convex and ovoid, robust (Fig. 15A). ***Integument*** Microsculpture indistinct on dorsal surfaces of head and pronotum, giving a shiny appearance. ***Head*** Relatively large (HW/PW = 0.73–0.75), antennomeres IV–X moniliform, slightly clavate. Frontoclypeal horn present, conspicuous. Ocular tubercles variable, present at least as a slight bulge, sometimes developed as a conspicuous hornlike projection below lateral carina. In some specimens, dorsad to lateral carina above tubercle on either side is a pale circular mark, more evident in teneral individuals. Two pairs of supraorbital setae present. Mentum with median pair of setae on tooth. ***Pronotum*** Relatively short (PL/ABL = 0.23–0.24) and broad (PW/EW = 0.83–0.84), moderately constricted posteriorly (PbW/PW = 0.72–0.78), sides evenly convergent to obtuse posterior angles. ***Elytra*** Moderately convex and ovoid, relatively broad (EW/ABL = 0.37–0.39), with weak traces of four striae. ***Legs*** Male protarsomeres 1 and 2 expanded and dentate on inner margin, with thick white adhesive setae ventrally (Fig. 10G). Male metatrochanters and posterior face of metafemora with coarse papillate microsculpture, metafemora slightly swollen. Female legs unmodified. ***Abdominal ventrites*** Last abdominal ventrite in males modified, apex protruding and bearing three blunt lobes, the inner slightly more prominent than the outer two (Fig. 15B). ***Male genitalia*.** Ring sclerite relatively large (RL/ABL = 0.30), narrow and subtriangular, with lightly-sclerotized expansions laterally; apex narrow and bent ventrally. Median lobe (Fig. 15E) complex, strongly asymmetrical (Fig. 17A), twisted dorsally from plane of basal lobes, heavily sclerotized on all faces. In strict right lateral aspect appearing as a blunt club-shaped organ without discernible apex. In dorsal aspect, nearly straight for basal 2/3, without basal bend, with ventral margin abruptly curved ventrally with apical sinuation. Left side of median lobe with a small, deep channel at base, with associated carina running obliquely for ~ 1/2 the length of median lobe. Apex variable: in topotype specimens, it is small, rounded, and deflected ventrally; in Clay Co., North Carolina, it is larger and more angularly produced ventrally. Small setae present on ventral surface of median lobe in some individuals. Basal lobes strongly asymmetrical, right lobe reduced to a narrow strap, left lobe large and cup-shaped. Flagellum long, thick at base and filamentous for most of its length beyond, evenly curved. Internal sac covered in small scales. Short scroll-like sclerite present at left side of ostium (behind flagellum in right lateral aspect), rolled into a tube; in lateral view appearing as a lightly sclerotized plate, rolled shape apparent in more posterior or anterior aspects. Right paramere minute and asetose, difficult to see in some aspects (Fig. 15D). Left paramere conchoidal, with enlarged base that is subequal in size to the rest of the paramere; ventral margin asetose (Fig. 15C). ***Female genitalia*.** Spermatheca small, curved, gradually enlarged apically, with swollen base (Fig. 21S). Spermathecal gland present, elongate. Spermathecal duct long and heavily coiled.

##### Distribution.

**Figure 16.**
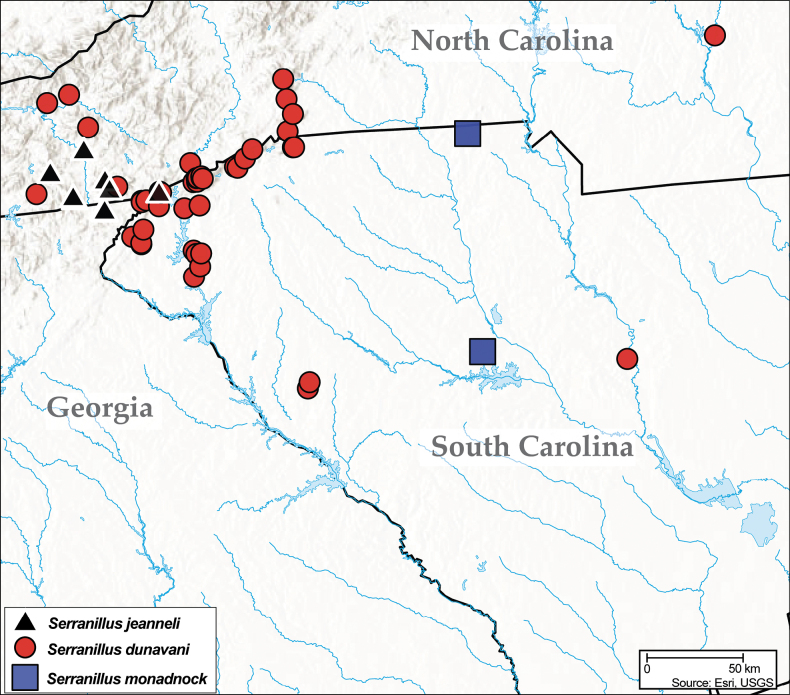
Distribution map of *Serranillus* species that occur in South Carolina. The locality for *Serranillus* sp. “South Carolina, Coon Branch” is the same as the single South Carolina *S.jeanneli* occurrence, and is not shown. Data are from Harden (2024).

This is the most widely distributed anilline in South Carolina (Fig. 16), with specimens known from Oconee Co. to Greenville Co. along the North Carolina border, south at least to Abbeville Co. and Kershaw Co. in the outer Piedmont and Sandhills physiographic regions, respectively. In North Carolina, the species has been found as far east as Montgomery Co., west to Clay Co., and north to Swain Co.

**Figure 17.**
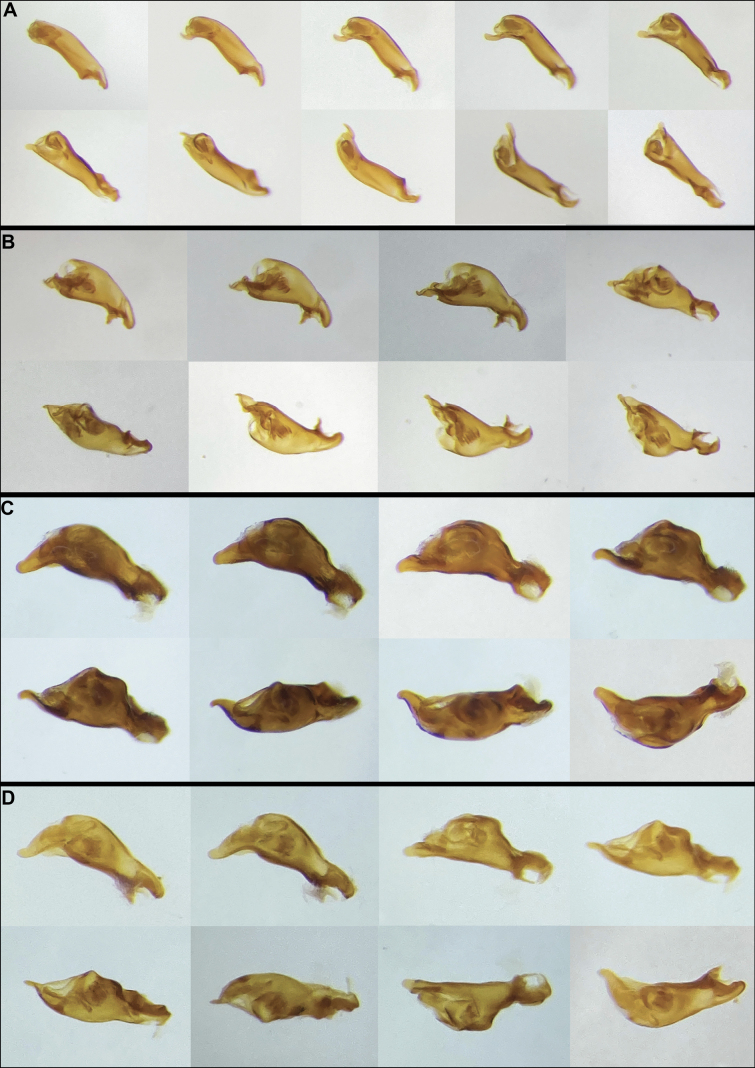
Multiple aspects of median lobes of South Carolina *Serranillus* species, progressively rotated dorsally from right lateral aspect (upper left) to left lateroventral aspect (lower right) **A***Serranillusdunavani***B***Serranillusjeanneli***C***Serranillusmonadnock*, holotype (Kings Mountain, SC) **D***Serranillusmonadnock*, paratype (Little Mountain, SC). Photographs not to same scale.

##### Sympatry.

In South Carolina, this species has been collected in association with the following eight anilline species: *A.castaneus* sp. nov. (Greenville Co.), *A.* sp. “South Carolina, Chestnut Ridge” (Greenville Co.), *A.cherokee* (Oconee Co.), *A.murrayae* (Greenville Co., Pickens Co., Oconee Co.), *A.dentatus* sp. nov. (Abbeville Co.), *Anillinus* sp. “South Carolina, Wateree”, *S.jeanneli*, and *S.* sp. “South Carolina, Coon Branch”.

##### Natural history.

Specimens have been obtained by Berlese extraction of sifted soil, litter, and coarse woody debris, and have been hand collected from under embedded rocks, bear dung, and pig carcasses. Two late instar larvae were collected in a sifted litter sample taken near the summit of Sassafras Mountain in October, in a mixed forest of pines and hardwoods. In a study of the soil and litter fauna of several South Carolina Piedmont forests, DuRant and Fox (1966) reported “*Anilluscarolinae* (Horn)” as the most abundant species of Coleoptera captured in a single litter sample; these specimens were probably *S.dunavani*.

#### ﻿﻿ Serranillus jeanneli

Taxon classificationAnimaliaColeopteraCarabidae

Barr, 1995

D7CA01C8-26CA-5B47-A913-8A3BE8FE53CB

**Figure 18.**
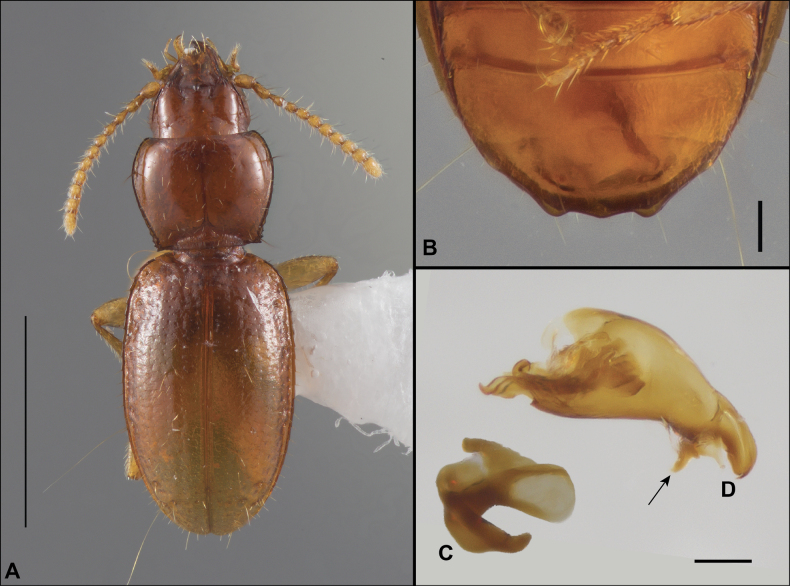
*Serranillusjeanneli* Barr. **A** dorsal habitus, abdomen removed for DNA extraction **B** last abdominal ventrite, ventral aspect **C** left paramere, left lateral aspect **D** median lobe (with right paramere attached, black arrow), right dorsolateral aspect. Scale bars: 1 mm (**A**); 0.1 mm (**B–D**).

Figs 2D , 16 , 17B , 18A–D , 21T , 25B

Serranillus jeanneli Barr 1995: 247.

##### Neotype male

**(CMNH)**, **here designated.** Dissected, with abdomen glued to point and genitalia in glycerin in plastic microvial pinned below labels, labeled: “NC: Macon Co. #62 Coweeta Exp. Sta. Ball Creek 3700’ 13Aug1969 T. Barr” “THOMAS C. BARR COLLECTION 2011 Acc. No. 38,014” “*Serranillusjeanneli* ♂ det. C.W. Harden 2024” “NEOTYPE *Serranillusjeanneli* Barr, 1995 des. Harden & Caterino 2024 [red cardstock]”

##### Material examined

**(*n* = 67). USA** • **Georgia** • 1 ♂; **Rabun Co.**; Chattahoochee National Forest, Rabun Cliffs; 34.913, -83.2978; 11 May 2021; M. Caterino and A. Haberski leg.; MSC-7026, CUAC000135496; • 1 ♂, 1 ♀; same data as previous; CUAC000172330 and CUAC000172322;• 2 ♀; **Rabun Co.**; Rabun Bald; 34.967, -83.299; 9 Jul. 2014; T. Lawton leg.; CWHc; • 1 ♀; **Rabun Co.**; Rabun Bald; 34.967, -83.299; 24 May 2014; T. Lawton leg.; CWHc; • 19 ♂, 15 ♀; **Rabun Co.**; Rabun Bald; 34.9708, -83.3032; 2 Jul. 2020; C.W. Harden leg.; CWHc; • 1 ♂; **Rabun Co.**; Chattahoochee National Forest, south of Beegum Gap; 34.9759, -83.3041; 5 Jun. 2023; C.W. Harden leg.; CWHc; • **North Carolina** • 4 ♂, 2 ♀; **Macon Co.**; Coweeta Hydrological Lab, ca. 13 miles west of Highlands; 35.045, -83.451; 23 May 1965; H.R. Steeves leg.; CMNH; • 1 ♀; **Macon Co.**; Coweeta Experimental Station [sic.], Ball Creek, #62, 3700’; 35.0339, -83.4505; 13 Aug. 1969; T.C. Barr leg.; CMNH; • 2 ♀; **Macon Co.**; Coweeta Experimental Station [sic.], Ball Creek #42, 3100’; 35.0432, -83.4535; 13 Aug. 1969; T.C. Barr leg.; CMNH; • 1 ♂; **Macon Co.**; Turtle Pond Creek, ca. 4 miles west-northwest of Highlands; 35.06, -83.26; 8 Aug. 1970; T.C. Barr leg.; CMNH; • 1 ♂, 1 ♀; **Macon Co.**; 0.6 miles northeast of Goldmine, California Ridge; 35.10, -83.28; 14 May 1971; T.C. Barr leg.; CMNH; • 5 ♂; **Macon Co.**; Nantahala National Forest, off Wayah Road ca. 10 km west from Route 64; 35.1554, -83.5584; 3 Aug. 2020; C.W. Harden leg.; CWHc; • 1 ♀; same data as previous; 4 Jun. 2021; • 1 ♀; **Macon Co.**; Nantahala National Forest, off Wayah Road ca. 10 km west from Route 64; 35.1557, -83.5583; 20 Oct. 2019; C.W. Harden leg.; CWHc; • 6 ♀; same data as previous; 3 Aug. 2020; CWHc; • 2 ♂; **Macon Co.**; four miles north of Franklin; 35.239, -83.374; 18 Mar. 1976; OSUC442503 and OSUC442504; OSUC; • **South Carolina** • 1 ♂; **Oconee Co.**; Coon Branch Natural Area, near Whitewater River; 35.023, -83.004; 23 Aug. 2022; C.W. Harden leg.; CWH-454, CUAC000185794.

GenBank: OR853398, OR839364, OR839681, OR837924, OR838086, OR838255.

##### Literature records.

Barr (1995) stated that the type locality was “along Ball Creek, elevation approximately 950 m, Coweeta Hydrologic Laboratory, U.S. Forest Service, Macon Co., North Carolina.” Field notes for the date of collection (13 August 1969) state that the site was below the first switchback of Ball Creek Road, approximate coordinates 35.0432, -83.4535. Barr also wrote that the species occurred in the Great Balsam Mountains in North Carolina and Towns Co., Georgia. Sokolov and Carlton (2008) state they studied specimens of *S.jeanneli* from “White County, Georgia” without further data. The *Serranillus* that we have studied from Towns Co. and White Co., Georgia are not *S.jeanneli* but two undescribed species.

##### Diagnosis.

From other species of *Serranillus*, *S.jeanneli* is best distinguished by the male median lobe of the aedeagus, which has a distinctive carinate shelf on the ventral surface, causing a preapical notch in the ventral margin in right lateral aspect (Fig. 18D). Externally, members of *S.jeanneli* are moderately sized for *Serranillus*, with male ABL = 2.13–2.35 mm and tentatively assigned females ABL = 2.07–2.51. The denticles on the last abdominal ventrite in males differ from other South Carolina species by having the inner denticle broader and less pronounced than the outer two (Fig. 18B).

##### Redescription.

***Habitus*** Robust and convex (Fig. 18A), moderately sized for genus, ABL = 2.07–2.51. ***Integument*** Dorsal microsculpture absent from most of head and pronotum, present at center of vertex. Small, irregular patches of weak microsculpture present on disc of pronotum in some individuals. ***Head*** Relatively narrow (male HW/PW = 0.70–0.71), frontoclypeal horn present and well developed. Ocular area behind antennal insertion with a dorsoventral linear tubercle at midpoint of carina. ***Pronotum*** Cordate, with sides broadly rounded and evenly converging posteriorly to constricted base, PbW/PW = 0.69–0.73. Approximately 1/4 body length (PL/ABL = 0.23–0.26). ***Elytra*** Ovoid and convex, with rounded humeri, weak traces of three striae present. ***Legs*** Male protarsomeres 1 and 2 expanded and bearing ventral adhesive setae. Male metatrochanters with coarse microsculpture, metafemora unmodified in either sex. ***Abdominal ventrites*** Males with last abdominal ventrite bearing three denticles on posterior margin, the outer two narrower and more prominent than the inner one. ***Male genitalia*** Ring sclerite long (RL/ABL = 0.32), similar in form to that of *S.dunavani*. Median lobe strongly asymmetrical (Fig. 17B), abruptly enlarged beyond base, outline a blunt-topped broad triangle in lateral aspects. Ventral face with a carinate shelf projecting below ventral margin; in right lateral aspect causing the ventral margin to appear notched (Fig. 18D). Apex of median lobe abruptly narrowed to a small curved hooklike apex visible in dorsal or ventral aspects. Internal sac of median lobe with two long sclerites. The right sclerite ribbonlike and corkscrewed along right side of internal sac, appearing as a dark curved shape in right lateral aspect (Fig. 18D), narrowing distally, where it protrudes from the ostium as a long evenly curved spine. The left sclerite is stouter, gradually narrowing along its sinuate length until it protrudes from the ostium beside the right sclerite as a bluntly hooked and curved spine. The left side of the ostium is dominated by a large rolled sclerite that curves dorsally from the left face of the median lobe into the internal sac, where it is rolled over itself twice; in right dorsolateral aspect, the scrolled sclerite appears to be a complex sclerotized structure resembling several stacked plates or a group of blunt spines (Fig. 18D); the true rolled shape is visible in posterior or anterior aspects. Flagellum not observed, possibly represented by a narrow, lightly sclerotized structure located in a similar position as the prominent flagellum in *S.dunavani* (cf. Fig. 15E). The parameres are as in *S.dunavani*, thus the right paramere is minute and asetose and the left paramere is large and conchoid, with a large base (Fig. 18C, D). The basal lobes of the median lobe are similarly asymmetrical, with the right lobe reduced to a thin strap and the left lobe larger and cup-shaped. ***Female genitalia*** Spermatheca small, less curved than in *S.dunavani* and with base less swollen (Fig. 21T). Spermathecal duct long and coiled.

##### Distribution.

Notwithstanding the comments of Barr (1995) and Sokolov and Carlton (2008), we have seen specimens only from a small area centered around the North Carolina-South Carolina-Georgia corner (Fig. 16).

##### Sympatry.

At Coon Branch in Oconee Co., South Carolina, this species co-occurs with *S.dunavani*, *S.* sp. “South Carolina, Coon Branch”, *Anillinusmurrayae*, *Anillinuscherokee*, and *A.* sp. “South Carolina, Coon Branch”.

##### Natural history.

Specimens have been collected from leaf litter, underneath embedded rocks, and using buried pipe traps.

##### Notes.

Barr’s concept of *S.jeanneli* involved at least three species: the one whose male genitalia he illustrated and which we consider *S.jeanneli*, a larger species whose median lobe has a ventral medial tuft of long curved setae (*Serranillus* sp. “North Carolina, Riley Knob”), and a closely related species that occurs in northern Georgia and the southern edge of the Great Smoky Mountains (*Serranillus* sp. “North Carolina, Miller Cove”). We have studied all of Barr’s anilline genitalia slide mounts, and the ones he identified as *S.jeanneli* were either those matching our concept or those belonging to *S.* sp. “North Carolina, Miller Cove.” The latter species does not occur at the type locality. Apparently, Barr never dissected *S.* sp. “North Carolina, Riley Knob”, the larger species that occurs at Coweeta, although his description implies that he considered it to be the same species whose genitalia he illustrated.

Barr (1995) designated a holotype and four paratypes for *S.jeanneli* and stated they were deposited at CMNH. However, no record exists of these specimens being deposited, and they could not be found in the type collection or the general collection (R. Androw, R. Davidson and A. Seago, pers. comm., January 2024). The specimens were also not found in any of the unprocessed material accessioned to CMNH after Barr’s death. One dried-out vial of undetermined specimens was found with label data matching Barr’s type series. In it were six anillines, including two female and one male *Serranillus*. However, the male was not the species that Barr illustrated as *S.jeanneli*, but the larger species we call *S.* sp. “North Carolina, Riley Knob.” To stabilize the name and clarify the identity of *S.jeanneli*, we have chosen as neotype a male of the correct species that was collected by Barr on the same day, along the same forest road approximately 1 km airline distance from the given type locality.

We noted that the previously published sequences from individuals identified as *S.jeanneli* (DNA1084 and DNA2309) were in a clade with *S.* sp. “North Carolina, Miller Cove” in our phylogeny, and the genitalia were confirmed to match that species rather than our concept of *S.jeanneli* (D. Maddison pers. comm., January 2024).

#### ﻿﻿ Serranillus monadnock sp. nov.

Taxon classificationAnimaliaColeopteraCarabidae

01B6AA75-3C23-5D27-BFF5-921091C3AAB0

https://zoobank.org/9968711C-1A42-426E-B295-23D834F53A73

**Figure 19.**
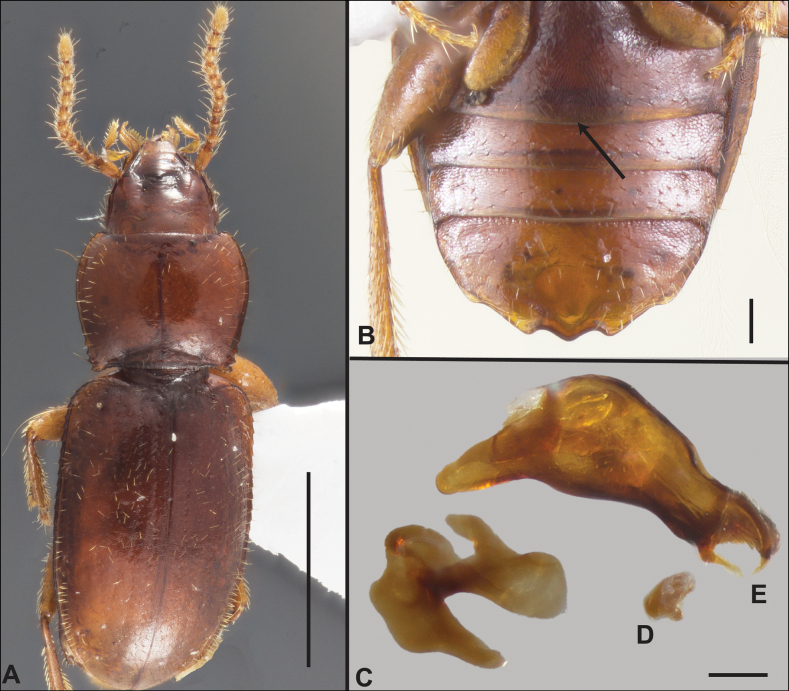
*Serranillusmonadnock* holotype **A** dorsal habitus **B** abdominal ventrites, ventral aspect (black arrow = posterior margin of medial area with stretched microsculpture) **C** left paramere **D** right paramere **E** median lobe. Scale bars: 1 mm (**A**); 0.1 mm (**B, C, D, E**).

**Figure 20.**
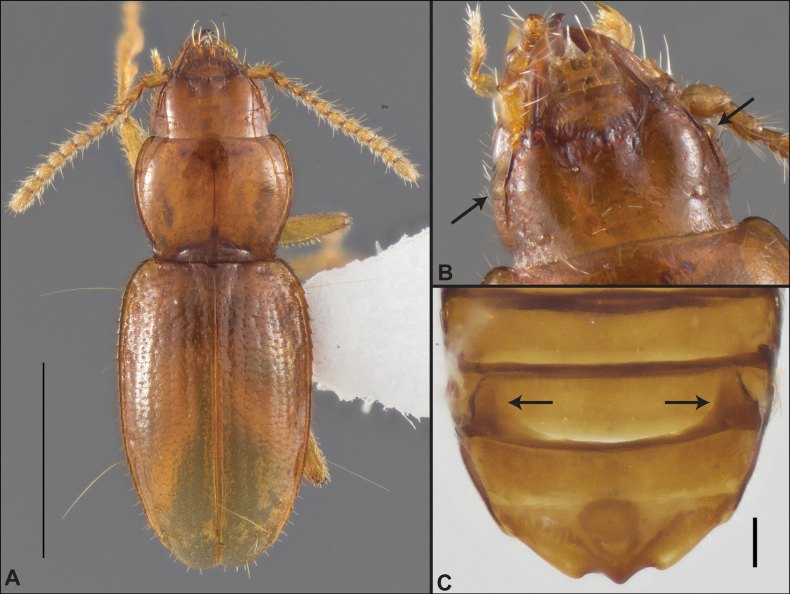
Morphological features of *Serranillus***A***Serranillus* sp. “South Carolina, Coon Branch” dorsal habitus **B** head of *Serranillusmonadnock*, dorsal aspect (black arrows = ocular tubercles) **C** dorsal aspect of abdominal ventrites of *Serranillusseptentrionis* (black arrows = lateral extensions of last abdominal tergite). Scale bars: 1 mm (**A**); 0.1 mm (**C**).

Figs 16 , 17C, D , 19A–E , 20B

##### Type material.

***Holotype male* (NCSU)**: point mounted, with genitalia in glycerin in plastic microvial pinned beneath labels. Original labels: “SCYorkCoKings MtStPklogslit Oct 28. 1989 JF&TADCornell” “Serranillus new species ♂ det. C.W. Harden 2021” “[QR Code] NCSU_ENT 00327997” “HOLOTYPE *Serranillusmonadnock* Harden & Caterino 2024” [red cardstock].

***Paratypes*** (*n* = 2, NCSU). **USA** • **South Carolina** • 1 ♂, 1 ♀; **Newberry Co.**; Little Mountain; 34.188, -81.408; 8 Dec. 2007; J. and S. Cornell leg.; sift and berlese litter 20”D Pine Stump Hole #5; NCSU_ENT00327998 and NCSU_ENT00327999; NCSU.

##### Diagnosis.

This is the only *Serranillus* species in which males have a flattened medial section of the second abdominal ventrite with longitudinally stretched microsculpture (Fig. 19B). The body is large, ABL = 2.82 mm (holotype), 2.79–2.87 mm (paratypes), the elytra and pronotum are broad, the head is relatively small (HW/PW = 0.65–0.70), the pronotum has the posterior angles produced posteriorly (Fig. 19A), and the median lobe of the aedeagus has a sinuate ventral surface, without ventral carinal shelf, with a large, blunt apex (Fig. 19E).

##### Description.

**Figure 21.**
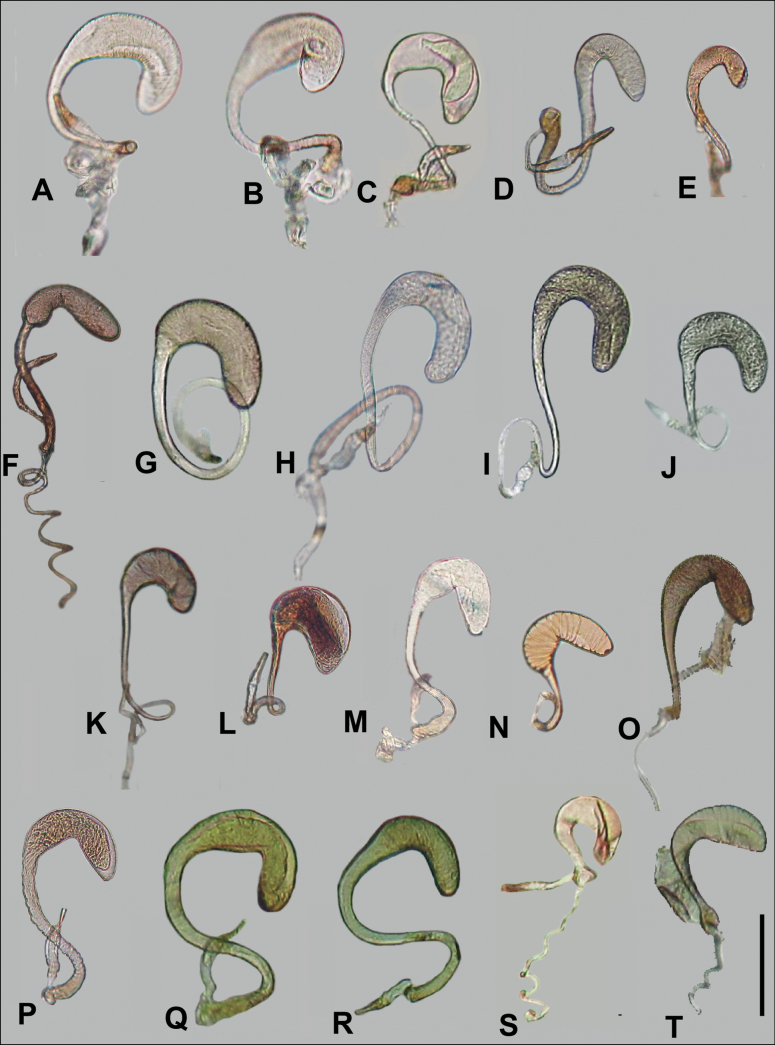
Spermathecae of Anillini species **A, B***Anillinuschandleri***C***Anillinuscastaneus***D***Anillinusmurrayae***E***Anillinussimplex***F***Anillinusdentatus***G***Anillinus* sp. “South Carolina, Coon Branch” **H***Anillinusmicamicus***I***Anillinusmica***J***Anillinuschoestoea***K***Anillinusseneca***L***Anillinusmontrex***M***Anillinusarenicollis***N***Anillinusjancae***O**Anillinuscf.nantahala**P***Anillinuscherokee***Q***Anillinusloweae***R***Anillinusmerritti***S***Serranillusdunavani***T***Serranillusjeanneli*. Scale bar: 0.1 mm.

***Habitus*** Large (ABL = 2.79–2.87) and robust (PW/EW = 0.84–0.88, EW/ABL = 0.36–0.38), with relatively small head (HW/PW = 0.65–0.70). ***Integument*** Dorsal microsculpture largely absent from surfaces of head, present across entire pronotum. ***Head*** Frontoclypeal horn well-developed and prominent. Ocular tubercle present on each side in the form of a short, rounded ridge running from lateral dorsal carina down a short distance laterally (Fig. 20B). Antennae short, not reaching posterior margin of pronotum when bent backward. ***Pronotum*** Broad (PW/EW = 0.84–0.88), margins not sinuate before posterior angles, which are slightly constricted (PbW/PW = 0.69–0.71) and protrude beyond posterior pronotal margin. ***Elytra*** Broad (EW/ABL = 0.36–0.38), disc flattened, with traces of five striae. ***Legs*** Male protarsomeres 1 and 2 expanded and bearing ventral adhesive setae. Male metatrochanters and posterior face of metafemora with coarse papillate microsculpture, metafemora swollen. Female legs unmodified. ***Abdominal ventrites*** Males with second abdominal ventrite with a flattened medial region where the microsculpture cells are stretched longitudinally (irregularly isodiametric elsewhere on abdomen). Males with last abdominal ventrite bearing three denticles on posterior margin, the inner one slightly more prominent than the outer two. ***Male genitalia*** Median lobe strongly asymmetrical and slightly twisted dorsally from plane of basal lobes (Fig. 17C, D). In right lateral aspect, the ventral margin undulating, with deep subapical sinuation before the blunt, rounded apex which is deflected ventrally. Row of short, stout setae present on ventral margin near and within subapical sinuation, visible at 100× or greater. Left side at base with broad, carinate channel that is interrupted medially by prominent dorsolateral region with two raised lumps; narrowed channel continuing across ventral surface, ending at subapical sinuation. Apex of median lobe in holotype curved to right side, appearing sinuous in dorsal or ventral aspects; apex in paratype straight and bladelike, possibly due to teneral condition of the specimen. Internal sac with thin, curved flagellum visible in right lateral aspect near dorsal margin. Large rolled sclerite present on left side of internal sac, making two coils over itself from left lateral wall of median lobe. Lightly sclerotized, blunt paddle-shaped sclerite extending apically from rolled sclerite, meeting left side before apex. Right paramere minute, bluntly rounded, asetose. Left paramere large, conchoidal, with thickened base. ***Female genitalia*** Spermatheca with enlarged base, otherwise similar to that of *S.jeanneli* (Fig. 21T). Spermathecal duct long and coiled.

##### Distribution.

Known from two localities in York and Newberry Counties (Fig. 16), both isolated monadnocks in the Piedmont ecoregion, Kings Mountain and Little Mountain.

##### Sympatry.

Three species of *Anillinus* occur at Kings Mountain State Park, and may co-occur with this species there. Three female *Anillinus* were collected from the same sample at Little Mountain, and are either unusually small individuals of *Anillinuschandleri* Sokolov or an undescribed species. A male *A.chandleri* was taken from a separate litter sample at the same locality.

##### Natural history.

The Kings Mountain specimen was presumably collected from extraction of sifted litter associated with logs, and the two Little Mountain specimens were collected by extraction of sifted litter from within a pine stump hole.

##### Species status justification.

Members of this species differ from those of the four previously described species in several external characters: pronotum with posterior angles produced posteriorly, males with flattened medial area with stretched microsculpture on second abdominal ventrite. The male genitalia are also unique, especially the relatively large, blunt apex of the median lobe.

##### Derivation of species name.

Noun in apposition, from “monadnock”, a word in the Abenaki language meaning an isolated, abruptly rising mountain or hill, in reference to the two known localities of this species on such features.

##### Notes.

The paratype male from Little Mountain is teneral, paler than both the female from the same collecting event at Little Mountain and the male holotype from Kings Mountain. The shape of the median lobe of the teneral paratype differs slightly from that of the holotype (Fig. 17C, D). In the paratype, the supabical sinuation on the ventral margin is deeper, and the apex of the median lobe is thinner and not curved to the right. Otherwise, the characters of the median lobe agree in both specimens. The unique medial microsculpture of the second abdominal ventrite is also the same in both males. The differences in the median lobe are either due to the teneral condition of the paratype, or may reflect slight geographic variation.

#### Serranillus sp. “South Carolina, Coon Branch”

Taxon classificationAnimaliaColeopteraCarabidae

624B67AE-4C7D-5BBB-B720-8674D32C1468

Figs 16 , 20A , 25B

##### Material examined.

**USA** • **South Carolina** • 1 ♀; **Oconee Co.**; Coon Branch Natural Area; 35.0251, -83.0053; 2 Oct. 2021; C.W. Harden leg.; On underside of embedded rock, steep north-facing slope; CUAC000169317, CWH-400; CUAC.

GenBank: OR853116, OR853403, OR839367, OR839665, OR837916, OR838073, OR838250, OR838133.

##### Diagnosis.

In addition to being larger (ABL = 2.70 mm), this female specimen differs from the widespread *S.dunavani* by the presence of faint microsculpture on the disc of the pronotum and the less ovoid shape of the elytra (Fig. 20A). The phylogeny based on the genes we sampled places the specimen in a well-supported clade with *S.septentrionis* Sokolov & Carlton and another potentially undescribed species from the Black Mountains in North Carolina.

##### Distribution.

Coon Branch Natural Area, near the Whitewater River in Oconee Co.

##### Sympatry.

The specimen was collected with adults of *Anillinusmurrayae* Sokolov & Carlton and two individuals of *Anillinus* that belong to the *sinuaticollis* group, discussed below as *Anillinus* sp. “South Carolina, Coon Branch”.

##### Natural history.

The specimen was collected in October from the underside of a large embedded rock in fine soil on a steep forested slope above the Whitewater River. Litter samples collected in June 2018, October 2020, and August 2022 did not collect this species, nor did a soilwash sample taken in June 2018.

##### Notes.

Without associated males, the identity of this species is uncertain. This female from Coon Branch possibly belongs to one of the undescribed species known from adjacent parts of Georgia and North Carolina that we lack DNA sequence data for. Males of both *S.* sp. “Georgia, Rabun Bald sp. 1” and *S.* sp. “North Carolina, Riley Knob” possess genitalia similar to *S.septentrionis*, suggesting they likely belong to the same clade as this Coon Branch female.

#### ﻿﻿Genus Anillinus

Taxon classificationAnimaliaColeopteraCarabidae

Casey, 1918

A040773F-8CC3-52B4-AEF2-65E4613DAFEC

Anillinus Casey 1918: 167. Type species: Anillus (Anillinus) carolinae Casey, 1918, by original designation.
Micranillodes Jeannel 1963a: 57. Synonymy established by Bousquet (2012: 699). Type species: Micranillodesdepressus Jeannel, 1963a, by original designation.
Troglanillus Jeannel 1963b: 147. Synonymy established by Barr (1995: 240). Type species: Troglanillusvalentinei Jeannel, 1963b, by original designation.

##### Adult diagnosis.

From *Serranillus*, members of *Anillinus* can be recognized by the position of the medial setae of the mentum not on the tooth (Fig. 11B), the last abdominal ventrite of males without dentate projections or lateral internal extensions, left mandible without a retinacular tooth (Fig. 11D), and right paramere well developed and bearing at least four setae. Most *Anillinus* also have shorter background pubescence on the elytral disc and a less robust habitus than *Serranillus*.

##### Larval diagnosis.

The single late-instar larval specimen of *Anillinus* that is known (Fig. 13A) differs from that of *S.dunavani* in possessing a serrate terebra (Fig. 14A) and stipes with gMX setae arranged in an even row (Fig. 14C). Early instar *Anillinus* do not seem to differ from early instar *Serranillus*.

##### Diversity.

The eight species described below bring the total number of described species of *Anillinus* to 78, making it the most speciose genus of Anillini. Including undescribed species that we have studied (Suppl. material 3), the total known diversity of *Anillinus* is at least 149 species.

##### Distribution.

West of the Mississippi, *Anillinus* are known from the Ozark Plateau in southern Missouri and Northern Arkansas, the Ouachita Mountains in Oklahoma and Arkansas, and the Balcones Escarpment in central Texas. In the east, *Anillinus* are known from Washington, D.C., Pendleton Co., WV, Cincinnati, OH (Dury 1902) and Lawrence Co., IN south to northern Florida, southern Alabama, and southeastern Louisiana.

#### ﻿﻿‘*dentatus* group’

##### ﻿﻿ Anillinus dentatus sp. nov.

Taxon classificationAnimaliaColeopteraCarabidae

E4679E5E-1988-5CCB-ABA7-53D0DD442D40

https://zoobank.org/250222AD-2A01-4DC5-90C2-58409C909833

Figs 2C , 12F , 21F , 22A–C , 23A–C , 24F , 25C , 43B

###### Type material.

***Holotype male* (USNM)**: point mounted, with abdominal ventrites and genitalia in Euparal on microslide pinned beneath specimen. Original labels: “USA: SC, Abbeville Co. Sumter NF Long Cane Crk at end of FS rd 530. 34.1133, -82.3300. 5.ii.2022. CW Harden & K Ivanov. Beneath embedded mossy rock in waterlogged soil.” “[QR Code] CLEMSON-ENT CUAC000163530” “Harden DNA Voucher CWH-420 *Anillinus* ‘dentate’ M Ext. 6-February-2022[green-bordered cardstock]” “HOLOTYPE *Anillinusdentatus* Harden & Caterino [orange cardstock]”

GenBank: OR853208, OR839248.

***Paratypes*** (*n* = 21; CMNH, NCSU, VMNH, OSAC, CUAC). **USA** • **South Carolina** • 1 ♂; same data as holotype; CUAC000163531, CWH-421; CUAC; • 5 ♀; same data as holotype; CUAC000163539 to CUAC000163541, CWH-422 to CWH-428; CUAC; • 1 ♀; **Abbeville Co.**; Sumter National Forest, near Secession St bridge; 34.134, -82.324; 25 Jan. 2020; C.W. Harden leg.; Underside of embedded rock; CUAC000163526, CWH-113; CUAC; • 1 ♂; **Abbeville Co.**; Sumter National Forest; 34.1345, -82.3230; 5 Feb. 2022; M. Ferro leg.; Sift subcort CWD; CUAC000168286; • 2 ♂; **Abbeville Co.**; Sumter National Forest, near Secession Street bridge; 34.1366, -82.3252; 5.February.2022; CW Harden leg.; beneath embedded mossy rock CUAC000163524 and CUAC000163529, CWH-417 and CWH-418; CUAC; • 1 ♀; same data as previous; CUAC000163542, CWH-419; CUAC; • 1 ♂; **Abbeville Co.**; Sumter National Forest, Long Cane Creek; 34.13519, -82.32503; 12 Jan. 2020; C.W. Harden & L.M. Thompson leg.; Soilwash flotation Berlese, clay-rich soil from ferny hill above floodplain; CUAC000163525, CWH-067; CUAC; • 2 ♂; **Abbeville Co.**; Sumter National Forest, Long Cane Creek; 34.1350, -82.3239; 15 Mar.–16 Jul. 2020; C.W. Harden leg.; Buried pipe trap baited with cheese, deep clay soil, LCC-05-0716; CUAC000163527, CUAC000163528; • 7 ♀; same data as for proceeding; CUAC000163532 to CUAC000163536; • 3 ♂, 3 ♀; **Abbeville Co.**; Sumter National Forest, Long Cane Creek area; 34.1370, -82.3226; 5 May 2023; C.W. Harden leg.; Under small rocks in ditch.; OSAC, VMNH, ADGc.

GenBank accession numbers for paratypes: OR853105, OR853210, OR839250, OR839420, OR837801, OR837962, OR838150, OR838114, OR853209, OR839249, OR839458, OR839458, OR837972, OR839247, OR839246.

###### Other material

**(*n* = 5). USA** • **South Carolina** • 2 ♂, 1 ♀; **Abbeville Co.**; Sumter National Forest, Long Cane Creek area; 34.1370, -82.3226; 5 May 2023; C.W. Harden leg.; Under small rocks in ditch.; CWHc; • 1 ♂; **Abbeville Co.**; Sumter National Forest, Long Cane Cr near Secession St bridge; 34.134, -82.324; 25 Jan. 2020; C.W. Harden leg.; Underside of embedded rock; CWHc; • 1 ♀; **Abbeville Co.**; Sumter National Forest, Long Cane Creek; 34.1349, -82.3241; 21 Mar. 2021; C.W. Harden and L.M. Thompson leg.; On underside of rock; CWHc.

###### Diagnosis.

Males of this species are easily recognized by the dentate mesotrochanters, a unique character in the genus. Females are most likely to be confused with *A.jancae* sp. nov., described below. Females of *A.jancae* are larger (ABL = 1.88 mm or greater), and the spermatheca is smaller (Fig. 21N).

###### Description.

**Figure 22.**
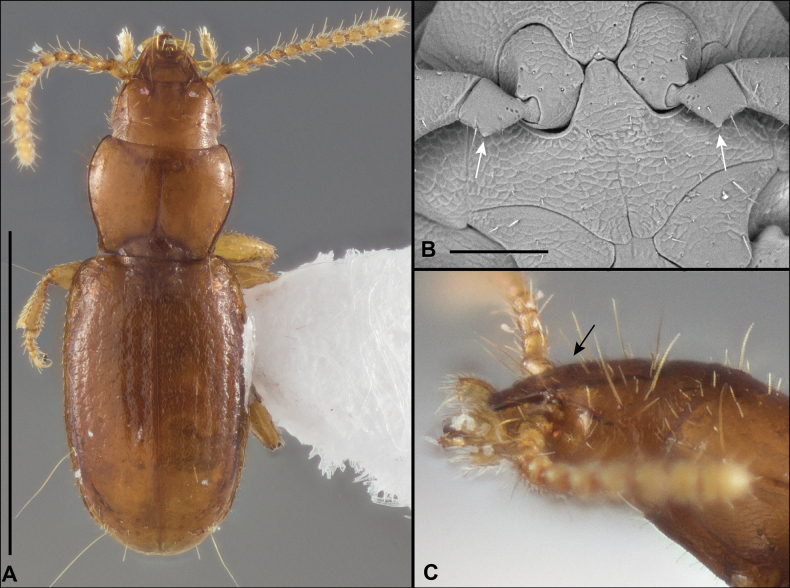
*Anillinusdentatus***A** dorsal habitus **B** SEM micrograph of metaventrite, ventral aspect (white arrows = mesotrochanter spines) **C** head, left lateral aspect (black arrow = absence of frontoclypeal horn). Scale bars: 1 mm (**A**); 0.1 mm (**B**).

**Figure 23.**
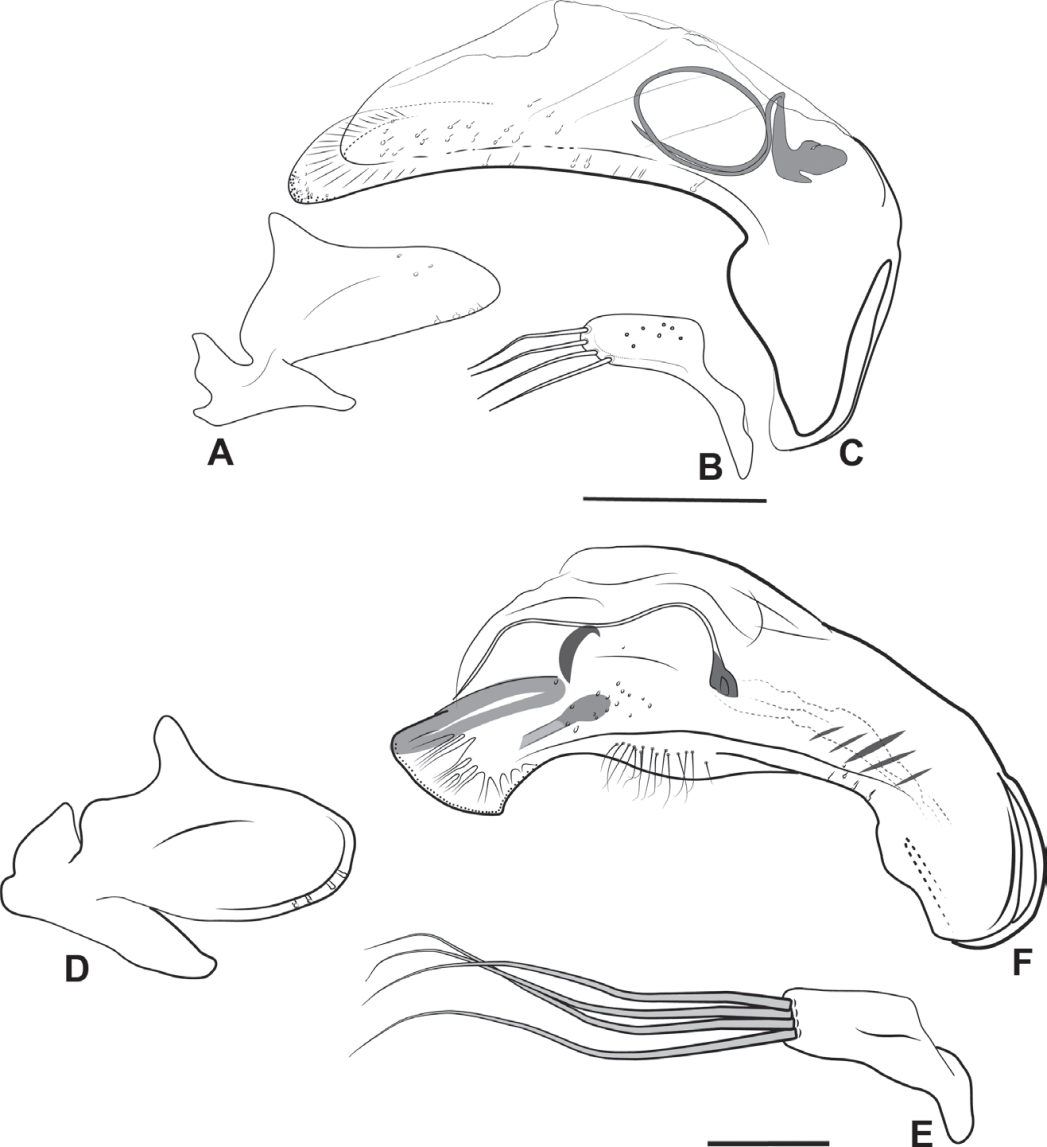
Male genitalia of *Anillinusdentatus* (**A–C**) and *Anillinusjancae* (**D–F**). Median lobes in right lateral (**C**) and right dorsolateral (**F**) aspects. Left parameres (**A, D**) and right parameres (**B, E**) in left and right lateral aspects, respectively. Scale bars: 0.1 mm.

**Figure 24.**
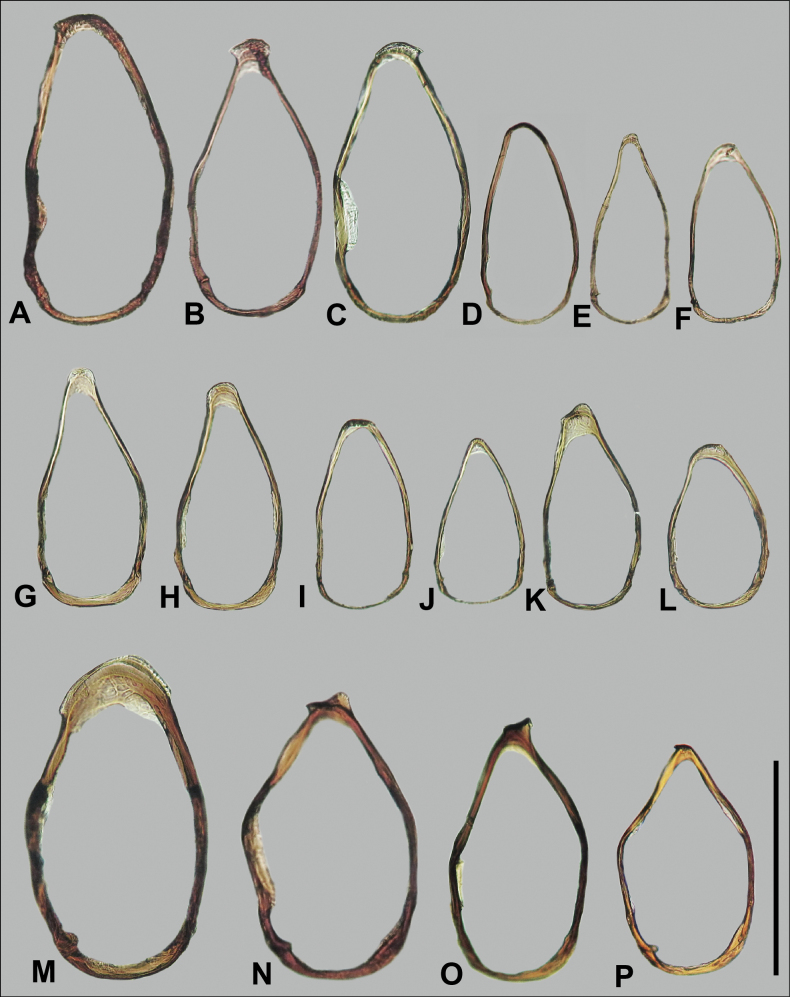
Male ring sclerite, ventral aspect. **A***Anillinuschandleri***B***Anillinuscastaneus***C***Anillinus* sp. “South Carolina, Chestnut Ridge” **D***Anillinusmurrayae***E***Anillinussimplex***F***Anillinusdentatus***G***Anillinusmica***H***Anillinusmicamicus***I***Anillinuschoestoea***J***Anillinusseneca***K***Anillinusarenicollis***L***Anillinusmontrex***M***Anillinusjancae***N***Anillinusloweae***O***Anillinuscherokee***P***Anillinusmerritti*. Scale bar: 0.5 mm.

***Habitus*** Body small (ABL = 1.49–1.69 mm, average = 1.59 ± 0.05 mm), flattened dorsoventrally and relatively narrow (avg. EW/ABL = 0.35) (Fig. 22A). Average ABL of males (1.63 mm, *n* = 5) greater than females (1.57 mm, *n* = 11). ***Integument*** Dorsal surfaces of forebody completely microsculptured with coarse mesh of irregular isodiametric cells. ***Head***HW/PW = 0.73–0.83. Antennomeres IV–X moniliform, slightly clavate. Frontoclypeal horn absent or barely suggested, inconspicuous in lateral view (Fig. 22C). Three pairs of supraorbital setae present. Mentum with median pair of setae posterior to the bead of the subtriangular mentum tooth, which is relatively large and blunt. ***Pronotum*** Relatively short (PL/ABL = 0.22–0.23), width variable (PW/EW 0.78–0.85); subcordate, strongly to moderately narrowed basally (bPW/PW = 0.70–0.80), anterior angles slightly prominent; sides typically slightly sinuate or evenly convergent towards obtuse hind angles; rarely, the sides are abruptly parallel sided just anterior to nearly right hind angles; pronotal sides with 3 or 4 basal serrulations. ***Elytra*** Parallel sided and flat, relative length variable (EL/ABL = 0.53–0.58); margins strongly serrulate; traces of five striae evident on disc of each elytron (Fig. 22A); without prominent subapical plica; fused inner margins slightly carinate at apex. ***Legs*** Profemora of males unmodified; protarsomere 1 of males moderately expanded, with inner margin spinose, bearing adhesive setae ventrally; protarsomere 2 not expanded, without adhesive setae ventrally (Fig. 10F). Mesotrochanters of males with spinose projection ventrally (Fig. 22B); mesotrochanters of females either evenly rounded or with blunt projection ventrally. Metafemora of males with prominent triangular toothlike projection on posterior margin (Fig. 12F). Tarsomeres of middle and hind legs of both sexes short and broad. ***Abdominal ventrites*** Unmodified in either sex. ***Male genitalia*** Ring sclerite (Fig. 24F) ~ 1/4 the total body length (RL/ABL = 0.25), sides asymmetric, margin of narrowed end slightly thickened but not deflexed in lateral view; sides without flattened lateral expansions medially. Median lobe of aedeagus (Fig. 23C) narrow and moderately curved, not twisted from plane of basal lobes; dorsal margin largely unsclerotized; ventral margin bladelike toward apex; apex rounded and slightly bent ventrally. Internal sac with flagellum elongate and filamentous distally, in repose coiled around itself in a circle and situated in basal 1/3 of median lobe. Right paramere (Fig. 23B) small and narrow, bearing four long setae on apex. Left paramere (Fig. 23A) with three or four small pores on ventral margin near apex, without setae. ***Female genitalia*** Spermatheca elongate, abruptly expanded distally; stem slightly sinuate, straight at proximal juncture with duct; duct long, tightly coiled in corkscrew pattern *in situ* (shown partially distended in Fig. 21F).

###### Distribution.

**Figure 25.**
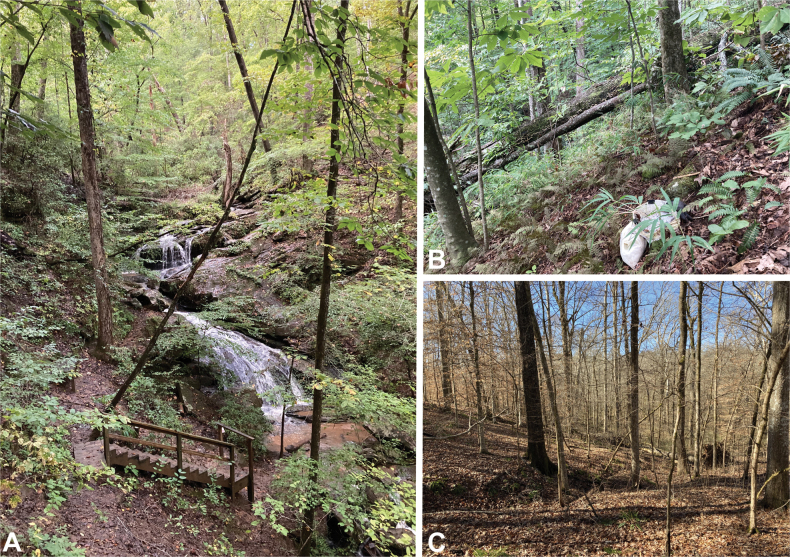
Anillini habitat in South Carolina **A** Waldrop Stone Falls, Pickens Co. (*Anillinusmica*, *Anillinusmicamicus*, *Anillinusmurrayae*, *Serranillusdunavani*) **B** Lower Whitewater River gorge, Oconee Co. (*Anillinuscherokee*, *A.murrayae*, *Anillinus* sp. “South Carolina, Coon Branch”, *S.dunavani*, *Serranillusjeanneli*, *Serranillus* sp. “South Carolina, Coon Branch” **C** Long Cane Creek, Abbeville Co. (*Anillinuschandleri*, *Anillinusdentatus*, *Anillinusjancae*, *S.dunavani*). The holotypes of *A.mica* and *A.micamicus* were collected under embedded rocks uphill of the wooden staircase in **A**. The holotype of *A.jancae* was collected in a pipe trap set on a hill just out of view in **C**.

Known from two localities within a small area of Sumter National Forest in Abbeville Co., SC along Long Cane Creek (Figs 25C, 43B)

###### Sympatry.

Members of this species have been collected under rocks in association with adults of *A.chandleri* and a larva of *A.jancae* sp. nov., and with *S.dunavani* in a sample of sifted coarse woody debris. Adults of *A.jancae* sp. nov. have been collected nearby.

###### Natural history.

Members of this species are endogean, inhabiting deep clay soils in mesic deciduous woods. Specimens have been found on the underside of embedded rocks in January, February, March, and May, and were collected in buried pipe traps operating from March to July but not January to March. One male specimen was collected in early February from Berlese extraction of sifted coarse woody debris. The sifted material was primarily subcortical, but some may have possibly come from the underside of logs that were on the soil surface (M. Ferro, pers. comm., February 2022). This material was collected near Long Cane Creek following a period of heavy rainfall, when the water level was above normal. Most of the specimens examined have had Laboulbeniales fungi present on the dorsum of the elytra, usually near the apex.

###### Species status justification.

The combination of morphological characters is unique within the genus, and DNA sequence data indicate that *A.dentatus* is distantly related to all other *Anillinus*. No known species of *Anillinus* possess characters suggesting a close relationship to *A.dentatus*.

###### Derivation of species name.

A male adjective referring to the triangular projections on the male metafemora, from the Latin for “toothed.”

###### Notes.

The long, coiled form of the flagellum in *A.dentatus* is also seen in *Anillinusbarri* Sokolov & Carlton, *Anillinuserwini* Sokolov & Carlton, *Anillinusfolkertsioides* Sokolov, and *Anillinusinexpectatus* Sokolov. However, with the exception of *A.folkertsioides*, all of these species differ from *A.dentatus* in most external characters. Members of *A.folkertsioides* and a possibly undescribed sister species from Fort Payne, Alabama are similar to *A.dentatus* in having fully developed microsculpture on the forebody and a depressed habitus, but differ from *A.dentatus* in having numerous long hairlike setae on the right paramere and male legs without modifications. Two character-states seen in members of *A.dentatus* are unique among known members of the genus: the nearly straight stem of the spermatheca and the spinose mesotrochanters of males.

#### ﻿﻿‘*albrittonorum* group’

##### ﻿﻿ Anillinus jancae sp. nov.

Taxon classificationAnimaliaColeopteraCarabidae

9E3BC079-AFD6-5D87-B7E0-BDFC595402C7

https://zoobank.org/0538E4CD-4117-4820-914F-399A48F0BFE2

Figs 10A, D , 12E , 13B , 14A, C, E , 21N , 23D–F , 24M , 25C , 26A–C , 27A , 43C

###### Type material.

***Holotype male* (USNM)**: point mounted, with genitalia in Euparal on microslide pinned beneath specimen. Original label: “USA: SC, Abbeville Co. Sumter NF Long Cane Creek. 34.1350, -82.3239. 15.March-16.July.2020. C.W Harden. Buried pipe trap baited with cheese. Deep clay soil. Oak, hickory, beech. LCC-01-0716.” “CLEMSON-ENT CUAC000168362” “HOLOTYPE *Anillinusjancae* Harden & Caterino [orange cardstock]”

***Paratypes*** (*n* = 7, CUAC). **USA** • **South Carolina** • **Abbeville Co.** • Sumter National Forest, Long Cane Creek; • 1 ♂; same data as holotype; CUAC000168363, CWH-204; • 1 ♀; same data as holotype; CUAC000168364, CWH-205; • 5 ♀; same data as holotype; CUAC000168365 to CUAC000168369.

GenBank accession numbers for paratypes: OR853233, OR839266, OR838005.

###### Other material

**(*n* = 2). USA** • **South Carolina** • **Abbeville Co.** • Sumter National Forest, Long Cane Creek • 1 ♀; 34.1362, -82.3235; 21 March 2021; C.W. Harden leg.; Underside of rock. CWHc; • 1 ♀; Near Cedar springs Rd.; 34.10914, -82.33948; 25 September 2020; C. W. Harden leg.; Underside of large rock; CUAC, CUAC000168370, CWH-241.

###### Diagnosis.

**Figure 26.**
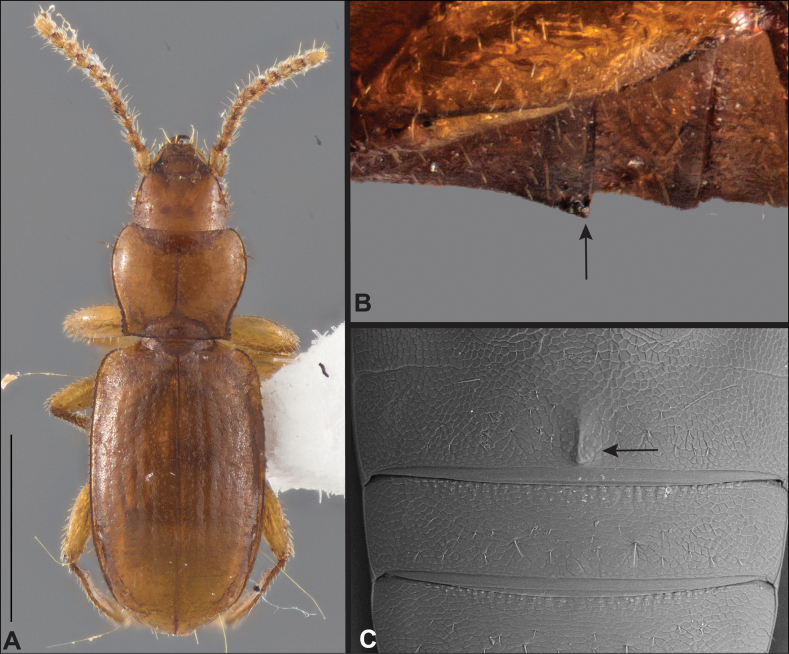
*Anillinusjancae***A** habitus **B** abdominal ventrites, left lateral aspect **C** SEM micrograph of abdominal ventrites, ventral aspect. Black arrows point to abdominal keel. Scale bar: 1 mm.

**Figure 27.**
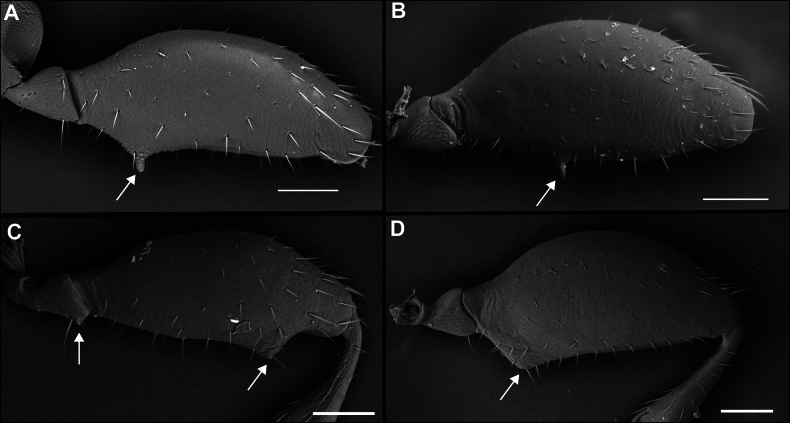
Modified profemora of *Anillinus* species. **A***Anillinusjancae***B***Anillinus* sp. “North Carolina, Orange Co. sp. 2” **C***Anillinuslescheni***D***Anillinus* sp. “Alabama, Aladdin Cave sp. 1”. White arrows point to protrusions on profemur and/or protrochanter. Scale bars: 0.1 mm.

Largest species of *Anillinus* in SC, with distinctive habitus (Fig. 26A), dorsoventrally flattened and not as compact as typical *Anillinus*; head relatively small (HW/PW 0.71–0.74); pronotum cordate, with sides subparallel at base, hind angles of pronotum prominent and rectangular or acute and extending posteriorly past hind margin. Protibiae of both sexes with a deep semicircular outer notch apically (Fig. 10A). Males with unique combination of secondary sexual characters: profemora with ventral spine (Fig. 27A), hind femora swollen and tuberculate, hind tibiae bowed inward and with inner surface scalloped, first abdominal ventrite bearing a short fin-like carina medially on posterior margin (Fig. 26B, C). Females with short spermatheca (Fig. 21N).

###### Description.

***Habitus*** Body dorsoventrally flattened and large (ABL = 2.32–2.64 mm). Males (2.63–2.64 mm) larger than females (2.32–2.36 mm). Less compact than most *Anillinus*, with relatively narrow forebody (HW/PW 0.71–0.74, PW/ EW = 0.76–0.83]). Pronotum with sinuate sides and long, parallel-sided posterior angles. ***Integument*** Dorsal surfaces of forebody mostly covered with irregularly isodiametric mesh of microsculpture, microsculpture absent from at least a small area on pronotal disc on each side of midline and from center of vertex, females more extensively microsculptured than males. Elytra with coarse mesh of isodiametric microsculpture, sculpticels small. ***Head***HW/PW = 0.71–0.74. Antennomeres I–IV[♂] or I–III[♀] longer than wide, V–X[♂] or VI–X[♀] moniliform. Frontoclypeal horn small, nearly absent in some specimens. Two pairs of supraorbital setae present. Mentum with median pair of setae posterior to bead of mentum tooth, which is relatively small and obtusely triangular, blunt. ***Pronotum*** Cordate, base strongly constricted in females (average Pbw/PW = 0.71, *n* = 3), moderately so in males (average Pbw/PW = 0.75, *n* = 2). Average PW/EW 0.81 (females), 0.77 (males). Relatively short (PL/ABL = 0.22 in all specimens measured, *n* = 5). Sides sinuate before long hind angles which are parallel sided and rectangular or slightly acute and projected posteriorly. ***Elytra*** Parallel sided and flat, relatively long (EL/ABL = 0.54–0.57); each with five striae, inner two or three more strongly impressed than outer two; small subapical plica evident; fused inner margins carinate at apex. ***Legs*** Profemora of males modified, swollen, and bearing a peg-like spine on posterior margin at proximal 1/3 (Fig. 27A). Protibiae of both sexes with deep semicircular notch on outer edge of apex; males with inner margin of protibiae extended as a bifid spine (Fig. 10A). Protarsi of males with first protarsomere greatly enlarged and produced on inner surface as a blunt lobe, with adhesive setae ventrally, second protarsomere unmodified and without ventral adhesive setae (Fig. 10D). Mesotrochanters of males unmodified. Male metatrochanters flattened, elongate, and covered in small tubercles; metafemora of males swollen and bearing several tubercles along posterior margin and a blunt angulate protrusion in distal 1/3 (Fig. 12E). Metatibiae of males concave along inner margin. Tarsomeres of middle and hind legs relatively short in both sexes. ***Abdominal ventrites*** Males with keel-like median carina at posterior margin of second visible ventrite (Fig. 26C); females without modifications. ***Male genitalia*** Ring sclerite large (RL/ABL = 0.28), oval; margins thickened; narrowed end with margin slightly deflexed; sides with small flattened lateral expansions medially. Median lobe (Fig. 23F) heavily sclerotized, with several parallel, diagonal sulci across surface in proximal 1/3 on left side, appearing as dark lines; strongly bicarinate ventrally, carinae forming a channel along most of ventral surface; band of many hairlike setae present across ventral surface and left side medially; apex expanded, with sides strongly curved ventrally, appearing bill-like in head-on view; in right lateral view, apex appears hatchet shaped, and thickened portions of curved cuticle appear as dark linear structures. Internal sac with long flagellum abruptly narrowed and filamentous past bulb-like base; small sclerotized straplike structure present ventrally at ostial opening. Right paramere (Fig. 23E) relatively short, with blunt apical margin bearing four or six long, thick setae that surpass the apex of the median lobe when paramere is attached. Left paramere (Fig. 23D) bluntly rounded at apex, with four poriferous canals and no setae on apical margin. ***Female genitalia*** Spermatheca short and small, abruptly enlarged distally, with short curved stem (Fig. 21N). Spermathecal duct longer than spermatheca, with a few loose coils.

###### Distribution.

Known for sure only from a single hillside on the southeast side of Long Cane Creek in Abbeville Co, within Sumter National Forest (Figs 25C, 43C).

###### Sympatry.

*Anillinuschandleri* and *A.dentatus* have been collected with this species. *Serranillusdunavani* is known from nearby.

###### Natural history.

Members of this species are endogean in habit. Most of the type series was collected from a buried pipe trap operated from March to July. The trap was set deeply in a layer of pure red clay, well below the shallow organic soil horizon. A late-instar larva was found underneath a deeply embedded rock in early February, indicating either overwintering of the egg or larval stage, or winter breeding and oviposition. A female adult was collected underneath a large, embedded stone. This female was kept alive for several months in a container with soil from the same locality packed into the bottom. The female quickly found a way around and underneath the packed clay soil on the bottom of the container, and apparently spent the remainder of its life in an inverted position on the underside of the clay. Given this behavior, and the microhabitat in which the trap that collected most of the type series was set, the habitat of this species is probably the series of crevices formed naturally in clay soils. Although clay is typically thought to be impervious, it forms naturally into aggregates called “peds” (Sherwood and Garst 2016), creating a series of crevices through which air and water (and small invertebrates) can pass. This habitat is widely distributed in the southeastern U.S., and further targeted sampling of it using buried traps is likely to discover many more species of anillines and other subterranean arthropods.

###### Species status justification.

The overall habitus, male secondary sexual characters, great length of setae on the apex of the right paramere, and characters of the median lobe are all unique within the genus. The consistent placement of this species in a clade with the geographically distant *A.albrittonorum* support its distinction from other *Anillinus* species.

###### Derivation of species name.

This remarkable species is named in honor of Janet C. Ciegler, in recognition of her contributions to the study of Coleoptera in South Carolina and the southeastern United States. Her many identification guides have made the study of beetles more accessible to amateur and professional entomologists alike. The specific name is a genitive noun derived from the shortened first name (“Jan”) and first letter of the surname.

###### Notes.

The female from the Cedar Springs Road site (CUAC000168370) is from the opposite side of Long Cane Creek, and differs from the type series in several respects. The specimen is smaller (ABL = 1.88 mm) and more compact, resembling *A.dentatus* in habitus. The head also has three supraorbital setae on each side, versus two in the type series. The spermatheca agrees with the females of the type series. DNA sequence data indicate that this female could represent a different species. The uncorrected p-distance of the barcoding region of COI of this specimen is 3.7% and 3.8% divergent from the two paratypes with sequenced barcodes. Its 28S sequence differs from that of the paratype male at only two sites – one substitution and a 2-bp insertion. CAD4 sequences of these same two specimens differ in 15 nucleotides. Male specimens from the Cedar Springs Road site would help resolve the situation. We note that members of *A.dentatus* have been collected from the same two sites, and show no differences in COI or 28S, so Long Cane Creek is likely not a barrier to dispersal in that species.

The male holotype of *A.jancae* is the only specimen in the “quadrisetose clade” to have more than four apical setae on the right paramere. Considering that the right paramere of the other known male of *A.jancae* is quadrisetose, we interpret the extra setae on the right paramere of the holotype to represent an unusual variant.

Our description of *Anillinusjancae* represents the first documented example of modified profemora in the genus. However, we found that the male profemora of the previously described *Anillinuslescheni* are also modified, though quite different in form, having a large, triangular tooth distally (Fig. 27C). Two undescribed species with modified male profemora are known, from North Carolina (Fig. 27B) and Alabama (Fig. 27D). The profemora of these two undescribed species are similar in form to *A.jancae* and *A.lescheni*, respectively. In the case of the North Carolina species, from which we have DNA sequence data, the similarities are the result of convergence.

#### ﻿﻿‘*valentinei* group’

##### ﻿﻿ Anillinus chandleri

Taxon classificationAnimaliaColeopteraCarabidae

Sokolov, 2011

EB7B962C-BC32-581C-A055-CE3327CCEA89

**Figure 28.**
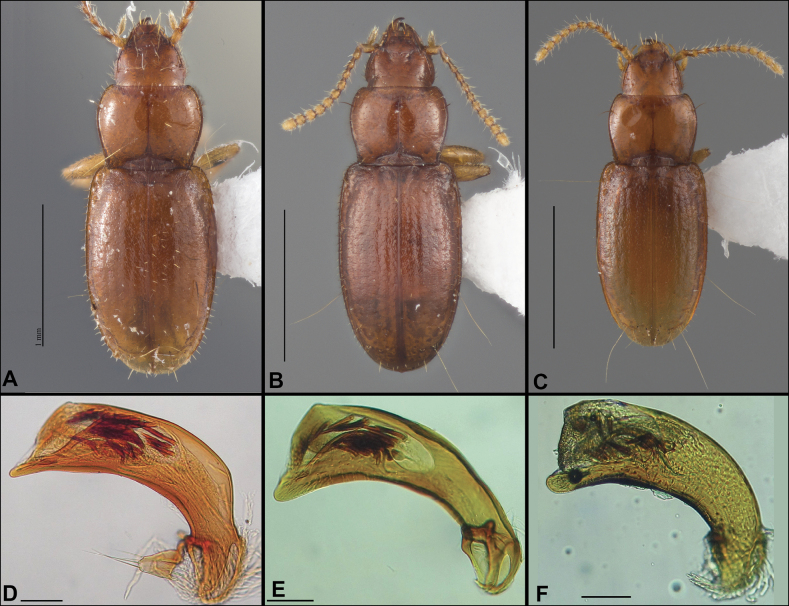
Dorsal habitus and dorsolateral aspect of median lobe of aedeagus of *Anillinuschandleri* and related species **A, D***Anillinus* sp. “South Carolina, Waldrop Stone” **B, E***Anillinuschandleri* Sokolov **C, F***Anillinus* sp. “South Carolina, Long Cane”. Scale bars: 1 mm (**A, B, C**); 0.1 mm (**D, E, F**).

**Figure 29.**
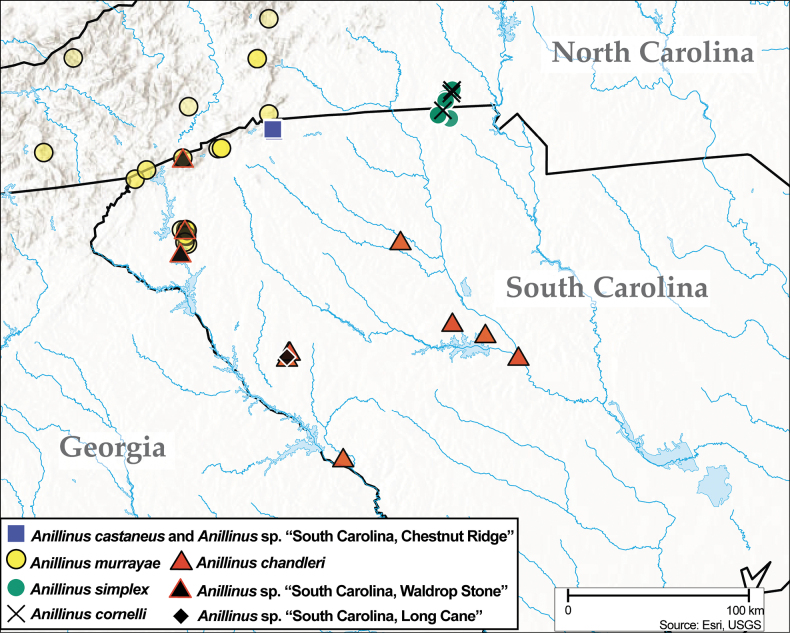
Distribution map of *valentinei*-group species in South Carolina and adjacent states. Data are from Harden (2024).

Figs 21A, B , 24A , 25C , 28B, E , 29

Anillinus chandleri Sokolov, 2011: 11.

###### Material examined.

***Holotype male* (USNM)**, glued to card, genitalia mounted on clear plastic slide pinned beneath specimen, labeled: “USA: SC: Edge. Co. Ft. Sumter Nat. For. Jct. Rds. 235 & 199” “VII-8-1987 RMReeves, sift forest litter” “Anillinus sp. det Bell” “HOLOTYPE *Anillinuschandleri* sp. n. Sokolov des. 2009”

###### Other material

**(*n* = 20, CUAC, CWHc, NCSU, UGCA). USA** • **South Carolina** • 1 ♂; **Abbeville Co.**; Sumter National Forest, Long Cane Creek; 34.1352, -82.3250; 12 Jan. 2020; C.W. Harden leg.; CWH-068, CUAC000168267; • 2 ♀; **Abbeville Co.**; Sumter National Forest, Long Cane Creek; 34.1356, -82.3238; 15 Mar. 2020; C.W. Harden leg.; CWH-151 and CWH-152, CUAC000153578 and CUAC000153579; • 1 ♂; same data as previous; CWHc; • 4 ♂, 4 ♀; **Abbeville Co.**; Sumter National Forest, Long Cane Creek; 34.1350, -82.3239; 15 Mar. to 16 Jul. 2020; C.W. Harden leg.; CWHc; • 1 ♂; **Abbeville Co.**; Sumter National Forest, off Cedar Springs Road; 34.1086, -82.3390; 25 Sep. 2020; C.W. Harden leg.; CWH-266, CUAC000168266; • 2 ♀; **Lexington Co.**; West Columbia; 34.001, -81.064; 10 Mar. 1991; J.C. Ciegler leg.; CUAC000153575 and CUAC000153576; • 1 ♂, 2 ♀; **Newberry Co.**; Little Mountain; 34.188, -81.408; 8 Dec. 2007; J.F. and S. Cornell leg.; NCSU; • 1 ♀; **Richland Co.**; Ballentine; 34.120, -81.236; 5 Nov. 2006; J.C. Ciegler leg.; CUAC000153574; • 1 ♂; **Union Co.**; Calhoun Critical Zone Observatory; 34.5866, -81.6472; 26 Sep. 2016; R. Carrera-Martinez leg.; UGCA.

###### Literature records.

The species was described from the male holotype only and has not been subsequently reported.

###### Diagnosis.

This is one of the largest species of *Anillinus* in South Carolina (Fig. 28B), and that combined with the effaced microsculpture from the forebody distinguishes it from nearly all other known species in the state. There are two undescribed *valentinei*-group species that approach the size of *A.chandleri*, but both differ in the form of male genitalia. The median lobe of *A.chandleri* is distinctive: long and strongly twisted dorsally from the plane of basal lobes, strongly constricted proximally, and expanded distally, internal sac with a dense group of large dark spines surrounding the long flagellum in repose (Fig. 28E).

###### Variation noted.

ABL = 1.89–2.26 mm, average = 2.11 ± 0.11 mm. Males (2.11–2.26 mm) larger than females (1.89–2.17 mm). The shape of the pronotum is variable, especially the relative width (PW/EW = 0.78–0.84). The proximal constriction of the median lobe is wider in the specimen from Union Co. than in other specimens studied. The number of spines in the internal sac is also variable, but the size and shape of the flagellum is constant. The ring sclerite (Fig. 24A), not described previously, is relatively large (RL/ABL = 0.34) and oval, asymmetrically narrowed anteriorly.

###### Description of female genitalia.

Spermatheca (Fig. 21A, B) broadly curved, gradually enlarged distally, proximally with abrupt perpendicular angulation. Spermathecal duct long and tightly coiled. Bursa with conspicuously sclerotized folds.

###### Distribution.

Although known only from South Carolina, this species has a larger range than most members of the *valentinei* group, from Abbeville and Edgefield Cos. east to Columbia and north to Union Co. (Fig. 29).

###### Sympatry.

At Long Cane Creek in Abbeville Co., SC, *A.chandleri* has been collected with *A.dentatus*, *A.jancae* and *S.dunavani*. *Anillinus* sp. “South Carolina, Long Cane”, an undescribed and apparently recently diverged member of the *valentinei* group, also occurs at Long Cane Creek. At Little Mountain, *A.chandleri* has been collected with *S.monadnock*.

###### Natural history.

Most of the material we have seen was collected from deep soil habitats. Several large litter samples collected from Long Cane Creek at various seasons (January, February, March, July, September) failed to produce specimens. Several specimens collected by J. Ciegler were sifted from litter, as were the holotype and the specimen from Union Co. The Union Co. site, the Calhoun Critical Zone Observatory, contains habitats that are an extreme example of highly eroded Piedmont forests, due to poor historical agricultural practices. The apparent persistence of a blind, flightless beetle such as *A.chandleri* at such a site is notable.

###### Notes.

Two undescribed species related to *A.chandleri* are discussed below.

##### ﻿﻿ Anillinus sp. “South Carolina, Long Cane”

Taxon classificationAnimaliaColeopteraCarabidae

BF9E0153-D2CE-5BF0-B9B4-92F0E1C00C20

Figs 28C, F , 29

###### Material examined

**(*n* = 2, CUAC). USA** • **South Carolina** • 1 ♂, 1 ♀; **Abbeville Co.**; Sumter National Forest, Long Cane Creek; 34.1095, -82.3397; 5 May 2023; C.W. Harden leg.; CWH-516 and CWH-517, CUAC000182318 and CUAC000182319.

GenBank: OR853246, OR839276, OR839722, OR837938, OR838268, OR853245, OR839275, OR839723, OR837939.

###### Diagnosis

Large (ABL of male = 2.13 mm), dorsoventrally flattened and parallel-sided (Fig. 28C). The median lobe of the aedeagus is similar to *A.chandleri* except it is not constricted basally, the ventral margin is less curved, the flagellum is shorter, and the spines in the internal sac are fewer and smaller (Fig. 28F).

###### Notes.

The two specimens were collected together underneath the same large rock. Several previous collecting visits to the same locality had produced specimens of *A.chandleri*, *S.dunavani*, and A.cf.jancae. The DNA sequences from the two specimens of *A.* “South Carolina, Long Cane” are nearly identical to those of *A.chandleri*, despite the apparent sympatry (Fig. 29) and morphological distinctiveness of the male genitalia.

##### ﻿﻿ Anillinus monadnock sp. “South Carolina, Waldrop Stone”

Taxon classificationAnimaliaColeopteraCarabidae

0CF12D7F-8EEA-59F8-8DAE-48EB37D16388

Figs 2M , 28A, D , 29

###### Material examined

(*n* = 4, CUAC). **USA** • **South Carolina** • 1 ♀; **Oconee Co.**; Martin Creek landing; 34.638, -82.866; 12 Apr. 2022; C.W. Harden leg.; Under large rock; CUAC000170070, CWH-440; • 1 ♀; **Oconee Co.**; Martin Creek landing; 34.6389, -82.8663; 26 May 2022; C.W. Harden leg.; Underside of large rock after heavy rain; CUAC000170071, CWH-447; • 1 ♂; **Pickens Co.**; Clemson Forest, Waldrop Stone Falls. 34.7385, -82.8252; 4 Aug. 2018; M. Caterino leg.; CUAC000109295, CWH-433; • 1 ♀; **Pickens Co.**; Chimneytop Gap; 35.0630, -82.7969; 21 Mar. 2023; C.W. Harden leg.; CUAC000066900, CWH-489.

GenBank: OR853159, OR839217, OR839704, OR837929, OR838097, OR838264, OR853160, OR839218, OR839673, OR837920, OR838081, OR839219, OR839353.

###### Diagnosis.

A large (ABL = 2.10–2.53 mm) and unusually setose member of the *valentinei* group (Fig. 28A). The armature of the internal sac as well as the shape of the flagellum are similar to those of *A.chandleri*, but the median lobe lacks the pronounced proximal constriction characteristic of that species (Fig. 28D).

###### Notes.

Repeated attempts to collect additional specimens of this species at Waldrop Stone Falls have been unsuccessful. The individuals from Martin Creek Landing and Chimneytop Gap are all female, so their association is tentative and mostly based on DNA sequence data. The individual from Chimneytop Gap (Fig. 2M) is smaller (2.10 mm) than the specimens collected near Clemson (2.47 and 2.53 mm), is divergent in DNA sequences from those specimens, and could represent a different species. With scattered material, only one male, and the allopatric distribution with respect to *A.chandleri* (Fig. 29), the support for our hypothetical distinctiveness of this species is not strong. Further sampling and collection of additional males, and perhaps more extensive molecular data, are required to clarify the status of this species and *A.* sp. “South Carolina, Long Cane.”

##### ﻿﻿ Anillinus murrayae

Taxon classificationAnimaliaColeopteraCarabidae

Sokolov & Carlton, 2004

19BC4D64-1F67-5A5B-9B4F-22489B3563A5

**Figure 30.**
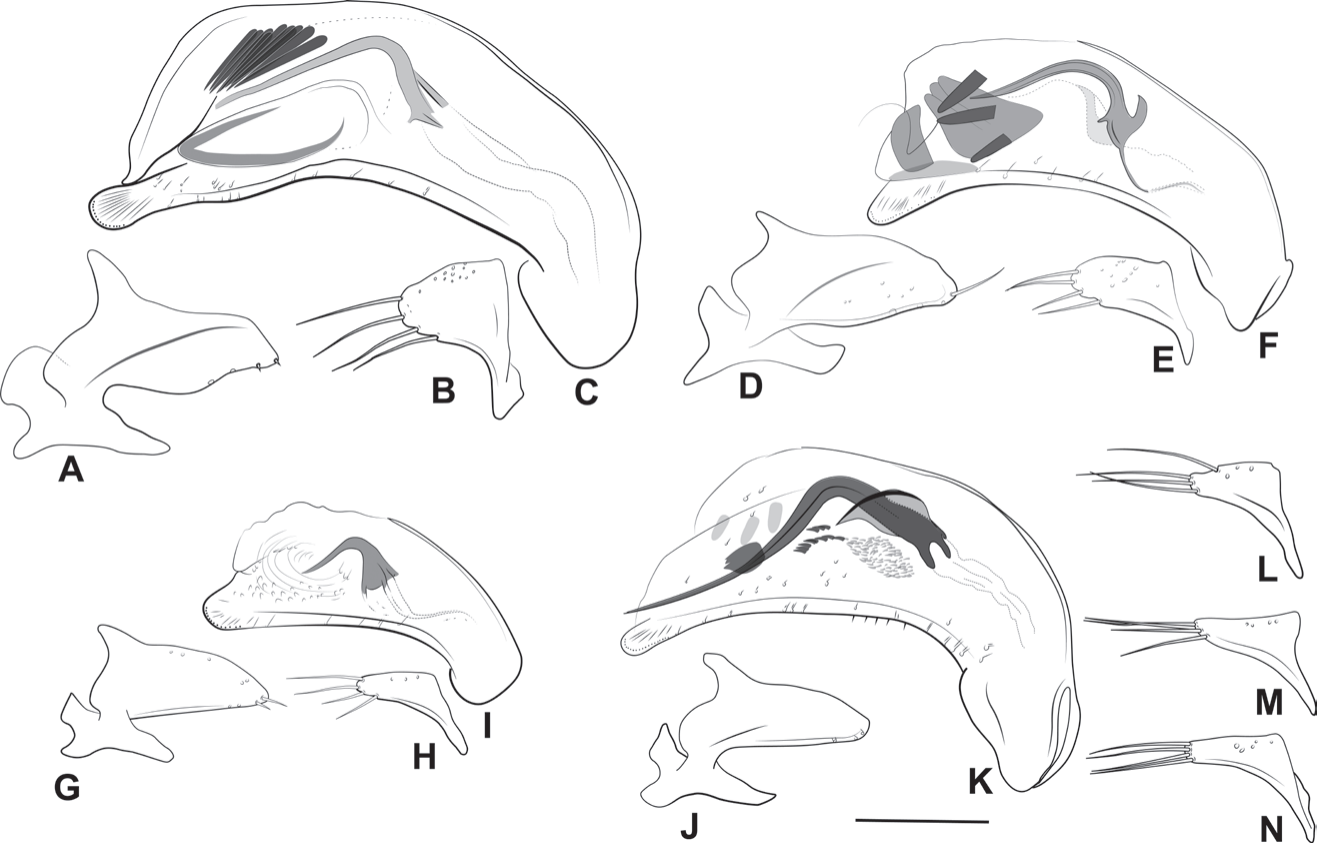
Aedeagi of *valentinei* group *Anillinus* species **A–C***Anillinuscastaneus* sp. nov. **D–F***Anillinuscornelli***G–I***Anillinussimplex* sp. nov. **J–N***Anillinusmurrayae*. Median lobes **C, F, I** in right dorsolateral aspect, median lobe **K** in right lateral aspect. Left **A, D, G, J** and right **B, E, H, L–N** parameres in left and right lateral aspects, respectively. Scale bar: 0.1 mm.

**Figure 31.**
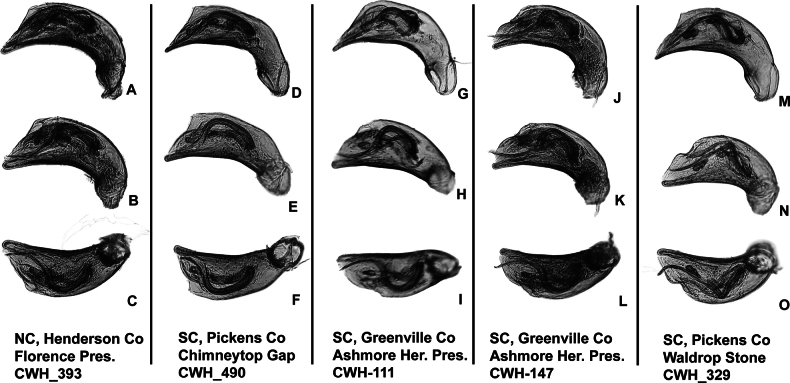
Median lobe of the aedeagus of *Anillinusmurrayae***A, D, G, J, M** right lateral aspect **B, E, H, K, N** right dorsolateral aspect **C, F, I, L, O** ventral aspect.

Figs 10H , 21D , 24D , 25A, B , 29 , 30J–N , 31A–O

Anillinus murrayae Sokolov & Carlton, 2004: 222.

###### Material examined.

***Holotype male*** (**USNM**), point-mounted backwards (on left side), dissected with genitalia in dried-out glycerin cup pinned beneath specimen, labeled: “USA: NC, Swain Co., GSMNP Collins Picnic Area, Quiet walk UTM 287857 E 3938299 N, C. Carlton 20 July 2002”

###### Other material

**(*n* = 64, CMNH, CUAC, CWHc, NCSU, OSUC, USNM). USA** • **North Carolina** • 1 ♂; **Buncombe Co.**; Round Knob; 35.6510, -82.2430; [no date]; OSUC442492; OSUC; • 1 ♂; **Henderson Co.**; Florence Nature Preserve; 35.4750, -82.3310; 13 Sep. 2020; C.W. Harden leg.; CWH-393, CUAC000168278; • 4 ♂, 4 ♀; same data as previous; CWHc; • 2 ♂, 4 ♀; **Jackson Co.**; Tennessee Mt [*sic*], Tom Beautell’s land; 18 May 1972; J. Hunter leg.; NCSU_ENT00293709 to NCSU_ENT00293714; NCSU; • 2 ♂; **Macon Co.**; Nantahala National Forest, west of Franklin; 35.1554, -83.5584; 4 June 2021; C.W. Harden leg.; CWHc; • 2 ♀; **Polk Co.**; Melrose Falls; 35.2217, -82.2985; 10 Aug. 2021; M.S. Caterino leg.; CWH-394 and CWH-395; CUAC000168276 and CUAC000168277; • 2 ♀; **Transylvania Co.**; White Pines group campground on Avery Creek; 35.2909, -82.7371; 24 Jul. 2009; J.F. and S. Cornell leg.; NCSU; • **South Carolina (new state record)** • 1 ♂, 1 ♀; **Greenville Co.**; Ashmore Heritage Preserve; 35.0878, -82.5800; 14 Apr. 2018; M. Caterino, M. Ferro, G. Powell leg.; CWH-111 and CWH-112, CUAC000168273 and CUAC000168274; • 1 ♀; **Greenville Co.**; Ashmore Heritage Preserve; 35.0888, -82.5979; 29 Jun. 2015; S. and C. Myers leg.; • 1 ♂, 2 ♀; **Greenville Co.**; Ashmore Heritage Preserve; 35.0867, -82.5788; 14 Mar. 2020; C.W. Harden and L.M. Thompson leg.; CWH-143 to CWH-145, CUAC000168263, CUAC000168265, CUAC000168268; • 2 ♂, 3 ♀; **Greenville Co.**; Ashmore Heritage Preserve; 35.0874, -82.5790; 14 Mar. 2020; C.W. Harden and L.M. Thompson leg.; CWH-146 to CWH-150, CUAC000168264, CUAC0001689269, CUAC000168270 to CUAC000168272; • 2 ♂; same data as previous; CWHc; • 1 ♀; **Oconee Co.**; Indian Camp Creek; 34.9899, -83.0724; 4 May 2015; M.S. Caterino and S. Myers leg.; SSM110, CUAC000169288; • 1 ♂, 1 ♀; **Oconee Co.**; Indian Camp Creek; 34.9903, -83.0723; 4 May 2015; M.S. Caterino and S. Myers leg.; SSM251 and SSM252, CUAC000169289 and CUAC000169290; • 1 ♂, 2 ♀; **Oconee Co.**; Coon Branch Natural Area; 35.0256, -83.0050; 2 Oct. 2021; C.W. Harden leg.; CWHc; • 1 ♂, 1 ♀; **Pickens Co.**; South Carolina Botanical Garden; 34.6718, -82.8234; 23 Apr. 2023; P. Rodrigues Flores and E. Recuero leg.; CWH-497 and CWH-498, CUAC000182300 and CUAC000182301; • 1 ♀; **Pickens Co.**; Clemson; 34.6820, -82.8330; 4 Oct. 1966; J.A. Payne leg.; CMNH; • 1 ♂; **Pickens Co.**; Clemson Experimental Forest, Pike Road inlet; 34.7109, -82.8238; 11 Mar. 2023; C.W. Harden and L.M. Thompson leg.; CWH-491, CUAC000066916; • 1 ♂; same data as previous; CWHc; • 1 ♀; **Pickens Co.**; Clemson Experimental Forest, Pike Road inlet; 34.7116, -82.8280; 26 Apr. 2023; C.W. Harden leg.; CWH-509, CUAC000182311; • 3 ♂, 2 ♀; same data as previous; CWHc; • 2 ♂, 2 ♀; **Pickens Co.**; Clemson Experimental Forest, Waldrop Stone area; 34.7358, -82.8187; 12 Nov. 2020; C.W. Harden leg.; CWH-329 to CWH-332, CUAC000168279 to CUAC000168282; • 1 ♂; same data as previous; CWHc; • 1 ♂, 2 ♀; **Pickens Co.**; Clemson Experimental Forest, Waldrop Stone Falls; 34.7393, -82.8205; 8 Oct. 2021; C.W. Harden leg.; CWH-406 to CWH-408, CUAC000168283 to CUAC000168285; • 1 ♀; **Pickens Co.**; Clemson Experimental Forest; 34.7396, -82.8479; 20 Feb. 2023; E. Recuero leg.; CWHc; • 1 ♀; **Pickens Co.**; Chimneytop Gap; 35.0628, -82.7977; 21 Mar. 2023; C.W. Harden leg.; CWH-508, CUAC000182310; • 1 ♂; **Pickens Co.**; Chimneytop Gap; 35.0631, -82.7960; 23 Mar. 2023; C.W. Harden leg.; CWH-490, CUAC000066882; • 1 ♂; **Pickens Co.**; Chimneytop Gap; 35.0632, -82.7964; 1 Jun. 2023; C.W. Harden leg.; CWHc.

###### Literature records.

There are no additional previously reported localities.

**Diagnosis.** Medium to large typical members of the *valentinei* group (ABL = 1.63–1.98 mm), with distinctive male and female genitalia, described below.

###### Redescription of male genitalia.

The median lobe (Figs 30K, 31A–O) has a narrow ventral margin and a small, rounded apex. In some individuals, there are several short setae present on the ventral margin. The flagellum is large and heavily sclerotized, rotated dorsally so that in right lateral aspect it is viewed through its base, falsely appearing to be evenly curved basally and straight beyond sinuation (Figs 30K, 31A, D, G, J, M). In dorsolateral aspect, the flagellum appears as illustrated in Sokolov et al. (2004), with a large basal piece and a long bisinuate shape that slightly surpasses the ostium (Fig. 31E, H, K, N). Several small blunt sclerites are present in the internal sac, as well as a small field of acute spines. Right paramere small and narrow, with four long apical setae that vary in their placement (Fig. 30L, M). The left paramere is narrowly conchoid and asetose (Fig. 30J).

###### Description of female genitalia.

Spermatheca long, deeply bisinuate, gradually enlarged distally (Fig. 21D). Spermathecal duct apparently absent. Bursa without sclerotization.

###### Variation noted.

In specimens from the vicinity of Sassafras Mountain in northern Pickens Co, SC, the flagellum of the internal sac of the aedeagus has a longer and broader basal curve and shorter apical curve (Fig. 31D–F). In specimens from southern localities in Pickens Co., SC, the internal sac of the median lobe lacks the blunt teeth present in other populations, the apical sclerite is saddle-shaped and lacks sharp projections, and the flagellum appears quite different from other populations in right lateral aspect (Fig. 31M) and dorsal aspect (Fig. 31N), because it is rotated differently within the median lobe in repose. Similarly, the spermatheca is the same size, but is oriented in a C-shape rather than an S. Further studies might support splitting *A.murrayae* into two or more species, but our sampling is too limited to support such an action.

###### Distribution.

This species is widely distributed, from Macon Co., NC east to Henderson Co., NC, north to the type locality in GSMNP and Round Knob in Buncombe Co. (Fig. 29). The population at the South Carolina Botanical Garden is the southernmost known locality.

###### Sympatry.

Specimens of *A.murrayae* have been collected in association with *A.mica* and *A.micamicus* at Waldrop Stone Falls, SC; with *A.merritti* at Indian Camp Creek, SC; with *A.cherokee* and *A.* sp. “South Carolina, Coon Branch” at Coon Branch, SC; with *A.merritti* and *A.langdoni*-group sp. near Wayah Bald, NC; and the paratype series from Jackson Co., NC is a mixture of *A.murrayae* and *A.loweae* (see Notes below). *Serranillusdunavani* co-occurs with *A.murrayae* at most of the known localities.

###### Natural history.

A mating pair was observed on the underside of a rock on 14 March 2020 at Ashmore Heritage Preserve in Greenville Co., SC. Specimens have been collected from sifted leaf litter, under embedded rocks, and using modified buried pitfall traps.

###### Notes.

Three of the male paratypes of *A.murrayae* in the NCSU collection are actually members of *A.loweae*, and have been labeled as such.

##### ﻿﻿ Anillinus cornelli

Taxon classificationAnimaliaColeopteraCarabidae

Sokolov & Carlton, 2004

FA190A38-3841-56E0-9B47-BD3F4423DD41

**Figure 32.**
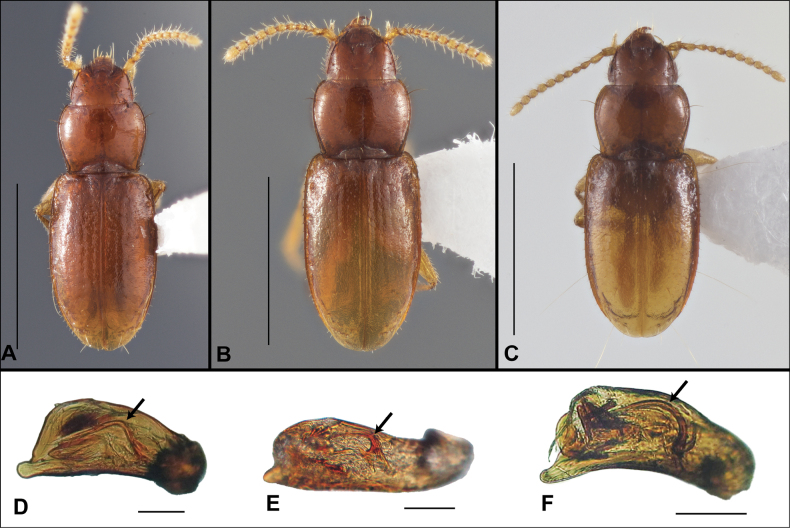
Dorsal habitus and median lobes of *Anillinus* species **A***Anillinuscastaneus* sp. nov. Holotype **B***Anillinus* sp. “South Carolina, Chestnut Ridge” **C***Anillinuscornelli*, abdomen removed for DNA extraction. **D***A.castaneus* sp. nov. Holotype, dorsal aspect **E***A.* sp. “South Carolina, Chestnut Ridge”, dorsal aspect **F***A.cornelli*, dorsal aspect. Black arrows point to proximal curve of flagellum. Scale bars: 1 mm (**A–C**); 0.1 mm (**D–F**).

Figs 29 , 30D–F , 32C, F

Anillinus cornelli Sokolov & Carlton, 2004: 209.

###### Material examined.

***Holotype male* (NCSU)**: USA. North Carolina: Gaston Co., Crowder’s Mt. State Park, Pine Log litter, J.F. Cornell leg., 23 June 1982. Specimen intact; aedeagus in microvial pinned beneath specimen, both parameres missing. (Sokolov et al. [2004] list USNM as the type depository).

###### Other material

**(*n* = 2, CUAC). USA** • **North Carolina** • 1 ♂, 1 ♀; **Gaston Co.**; Crowder’s Mountain State Park, near Linwood Access; 35.2417, -81.2717; 29 Apr. 2023; C.W. Harden leg.; under embedded rock; CWH-514 and CWH-515, CUAC000182316 and CUAC000182317.

GenBank accession numbers for topotype specimens: OR853205, OR839243, OR839720, OR837937, OR838267, OR853204, OR839242, OR839721.

###### Literature records.

The species has been reported from Kings Mountain State Park in South Carolina, without more specific locality information (Sokolov et al. 2004).

###### Notes on the type.

The median lobe of the aedeagus is damaged: the basal lobes appear to be partially torn off, and the organ is laterally flattened and distorted, as if it were previously crushed beneath a cover slip. The illustration of the median lobe in Sokolov et al. (2004) reflects this condition, and is not an accurate depiction of its shape and structure.

###### Diagnosis.

Members of *A.cornelli* are relatively large (ABL = 1.75–1.81 mm) and broad (PW/EW = 0.83–0.85, EW/ABL = 0.36–0.38) (Fig. 32C). As is typical of the *valentinei* group, microsculpture is effaced from most of the dorsal surfaces of the forebody, three large supraorbital setae are present and both the first and second protarsomeres of males have thick white adhesive setae ventrally. The metafemora of males are slightly swollen, larger than in females, but without teeth or coarse microsculpture patches. The male genitalia are distinctive, and are redescribed based on a recently collected specimen below.

###### Redescription of male genitalia.

Ring sclerite average sized for genus (RL/ABL = 0.27), oval and asymmetrically narrowed anteriorly, with anterior margin deflected ventrally. Median lobe (Fig. 30F) strongly asymmetrical, with apex abruptly and conspicuously curved to right side. In right lateral aspect, appearing obtusely angulate proximately, nearly straight medially and abruptly curved ventrally at apex. In right dorsolateral aspect, slightly curved and enlarged distally. Left side with small excavation proximally and long carina extending along nearly entire length. Internal sac with long, well-sclerotized flagellum that in dorsolateral aspect is strongly curved proximately (Fig. 32F), with long basal extension, becoming filamentous distally, where it coils and extends beyond ostium on right side. Three large, blunt spines are present on right side of internal sac near ostium; a fourth spine appears to be present ventral to the others, just before the apex of the median lobe, but this is an artifact caused by the thick, curved sclerotized wall of the aedeagus seen through its curve in right lateral aspect. Behind the three spines, against the ostial opening, a sclerite with three blunt spines fused at the base is present, appearing comb-like. Distal to this comb-like structure is a smaller semicircular ostial sclerite. Right paramere short, bearing four short apical setae (Fig. 30E). Left paramere conchoidal, with four preapical pores on ventral margin, the apical one bearing a long seta (Fig. 30D).

###### Description of female genitalia.

Spermatheca long, bisinuate, gradually enlarged distally. Spermathecal duct long and heavily coiled. Bursa with lightly sclerotized folds.

###### Distribution.

Endemic to Kings Mountain, a monadnock that spans the North Carolina-South Carolina border. (Fig. 29).

###### Sympatry.

At Crowders Mountain State Park, *A.cornelli* co-occurs with *A.simplex* sp. nov. and a species belonging to the *elongatus* group, possibly conspecific with *A.montrex*. Both *A.simplex* and *A.montrex* also occur at Kings Mountain, along with an undescribed species of *Serranillus*.

###### Natural history.

The type material was reportedly collected by litter extraction. On 29 April 2023, CWH collected a pair of *A.cornelli* from the underside of a large embedded rock on a gently sloping wooded hillside. Several large samples of sifted litter and soil taken from the same locality and another locality within Crowders Mountain State Park on the same date failed to produce specimens of *A.cornelli*. Two days of intensive hand collecting and litter extraction at Kings Mountain State Park also failed to produce specimens of *A.cornelli*.

###### Notes.

We have not seen material of this species from South Carolina. No paratypes were found in collections, including those from Kings Mountain State Park reported by Sokolov et al. (2004) to be deposited in the NCSU collection (Bob Blinn pers. comm., March 2022).

##### ﻿﻿ Anillinus castaneus sp. nov.

Taxon classificationAnimaliaColeopteraCarabidae

DFBBD752-2F2E-5B97-9AC2-FE02A8F5D5C2

https://zoobank.org/4CFF8F11-E1CE-422A-B68E-F3EA28B831EA

Figs 21C , 24B , 29 , 30A–C , 32A, D

###### Type material.

***Holotype male* (USNM)**: point mounted, with genitalia in Euparal on microslide pinned beneath specimen. Original label: “USA: S. Carolina: Greenville Co. Chestnut Ridge Heritage Pres. N 35.1471, W -82.2841. 8 April 2018 (373) Sift/Berl CWD5 M. Ferro” “CLEMSON-ENT CUAC000080962” “HOLOTYPE *Anillinuscastaneus* Harden & Caterino [orange cardstock]”

***Paratypes*** (*n* = 9, CUAC). **USA** • **South Carolina** • **Greenville Co.**; Chestnut Ridge Heritage Preserve; • 2 ♀; same data as holotype; CUAC000080963 and CUAC000080964; • 1 ♀; **Greenville Co.**; Chestnut Ridge Heritage Preserve; 35.1506, -82.2779; 8 Apr. 2018; M. Caterino & L. Vasquez leg.; sifted litter; CUAC000108120; • 2 ♂; **Greenville Co.**; Chestnut Ridge Heritage Preserve; 35.1523, -82.2814; 5 Jun. 2015; S. Myers leg.; Hardwood litter; CUAC000170064 and CUAC000170065, SSM098 and SSM099 [these two specimens do not have molecular voucher labels, but have been extracted and bear identical locality data to that entered for these voucher numbers by S. Myers]; • 1 ♀; **Greenville Co.**; Chestnut Ridge Heritage Preserve; 35.1501, -82.2820; 5 Jun. 2015; S. Myers leg.; Hardwood litter; CUAC000170066, SSM-101; • 1 ♀; **Greenville Co.**; Chestnut Ridge Heritage Preserve; 35.14970, -82.28207; 20 Oct. 2021; C.W. Harden leg.; On underside of large rock beside rivulet; CUAC000170067, CWH-415; • 1 ♀; **Greenville Co.**; Chestnut Ridge Heritage Preserve; 35.1507, -82.2821; 15 Mar. 2022; C.W. Harden leg.; Berlese, deep duff/soil; CUAC000170068;• 1 ♀; **Greenville Co.**; Chestnut Ridge Heritage Preserve; 35.1406, -82.2790; 5 Jun. 2015; S. Myers leg.; Secondary litter; CUAC000025521.

GenBank accession numbers for paratypes: OR839224, OR853178, OR839223, OR839749, OR837941, OR838100, OR838278, OR853179, OR839821, OR838112, OR838296.

###### Diagnosis.

A moderately large typical member of the *valentinei* group, externally similar to *A.murrayae* and *A.cornelli* (Fig. 32A). The male genitalia are distinctive, particularly the tripartite apex of the median lobe formed by the ventral margin, dorsal margin, and ostial plate (Fig. 30C), which is unique among *Anillinus* species east of the Appalachians.

###### Description.

***Habitus***ABL = 1.81–1.85 mm. ***Integument*** Dorsal microsculpture effaced from most of forebody, present only medially on vertex and weakly impressed on frons and extreme margins of pronotum. ***Head***HW/PW = 0.73–0.74. Frontoclypeal horn well-developed. Three pairs of supraorbital setae present, posterior outer pair smaller than other two. ***Pronotum*** Form variable, either convex and smoothly polished, or subdepressed and with microsculpture along margins. Relatively short (PL/ABL = 0.22–0.23) and broad (PW/EW = 0.82–0.85), sides evenly convergent behind middle, moderately constricted basally (PbW/PW = 0.74–0.77). ***Elytra*** Slightly ovoid, convex, broad (EW/ABL = 0.35–0.36), with large umbilicate punctures. ***Legs*** Male protarsi with protarsomeres 1 and 2 expanded and dentate on inner margin, both bearing adhesive setae ventrally. Male profemora unmodified. Male mesotrochanters unmodified. Male metafemora slightly swollen, with patch of coarse papillate microsculpture medially on posterior face. ***Abdominal ventrites*** Unmodified in either sex. ***Male genitalia*** Ring sclerite large (RL/ABL = 0.35), oval, strongly constricted anteriorly where it forms a curved shelf projecting ventrally. Median lobe (Fig. 30C) strongly asymmetrical, twisted dorsally from plane of basal lobes and curved to the right side. In right lateral aspect, appearing strongly curved, with the ventral margin somewhat angular and sinuate before apex, with several short setae present in proximal bend. In right dorsolateral view, appearing slightly curved and slightly enlarged distally, dorsal margin well sclerotized and forming a sharp beak apically, ventral margin narrowly expanded, apex small and produced, buttonlike. Left side of median lobe at base with large semicircular excavation that occupies the entire basal section before bend, associated carina distal to excavation short, ending at ostium that is large, occupying most of the left face of the median lobe. Internal sac with complex armature: the flagellum is long, well sclerotized, with long basal projections, obtusely bent medially or evenly curved depending on angle at which it is viewed, apex ending at ostium; a group of large, dark sharp spines is present dorsally at ostium; a large, spade-like ostial plate is present on left ventrolateral face of apex; in dorsal or ventral views, the apex appears tripartite, with the dorsal and ventral margins meeting and the pointed ostial plate projecting between them. Shape of flagellum and other internal sclerites appearing as an indecipherable dark mass in right lateral aspect. Right paramere short and broad, with four apical setae (Fig. 30B). Left paramere conchoid, with fore preapical pores on ventral face, apical two bearing short setae (Fig. 30A). ***Female genitalia*** Spermatheca long, abruptly enlarged distally, stem bent at a slightly acute angle proximally or evenly curved (Fig. 21C). Spermathecal duct not apparent in the two specimens examined. Bursa copulatrix with conspicuous sclerotized folds.

###### Distribution.

Known only from a small area of Chestnut Ridge Heritage Preserve in Greenville Co., SC (Fig. 29).

###### Sympatry.

Members of this species have been collected with *A.* sp. “South Carolina, Chestnut Ridge” and *S.dunavani*.

###### Natural history.

Most specimens have been collected through Berlese extraction of sifted leaf litter and dead wood. Two specimens were collected from the undersides of rocks.

###### Species status justification.

The male genitalia are unique within the genus, particularly the complex tripartite apex of the median lobe. DNA sequence data indicate the species is most closely related to *A.simplex*, *A.cornelli* and the undescribed species *A.* sp. “South Carolina, Chestnut Ridge”, which all differ from *A.castaneus* in external structure and male genitalic characters. *Anillinuscastaneus* and “South Carolina, Chestnut Ridge” occur in syntopy, providing strong evidence that the two are reproductively isolated.

###### Derivation of species name.

A male adjective, from the Latin for Chestnut, in reference to the color of the mature specimens and the name of the type locality, which itself is presumably named for the American Chestnut tree, once an abundant component of Appalachian forests.

###### ﻿﻿ Anillinus sp. nov.

Taxon classificationAnimaliaColeopteraCarabidae

690D3787-1F77-5ED3-9D26-EB7CDDC49259

Figs 24C , 29 , 32B, E

####### Material examined.

**USA** • **South Carolina** • 1 ♂; **Greenville Co.**; Chestnut Ridge Heritage Preserve; 35.15071, -82.28211; 20 Oct. 2021; C.W. Harden leg.; on underside of embedded rock, alluvial forest near Little Pacolet River; CWH-401, CUAC000170069.

GenBank: OR853206, OR839244, OR839666, OR837917, OR838074, OR838251.

####### Diagnosis.

The single specimen, a male (Fig. 32B), is large (ABL = 1.98 mm) and unusual in having the dorsal microsculpture of the forebody strongly developed in the pattern of the *loweae* group (present on entire surface except for paramedian patches on vertex) while being an otherwise typical member of the *valentinei* group, whose members typically have dorsal microsculpture largely absent. The median lobe is typical of the *valentinei* group, with a long, well-sclerotized flagellum and several well-sclerotized spines lining the internal sac. The proximal curve of the flagellum (Fig. 32E) is shorter than in *A.castaneus* (Fig. 32D).

####### Notes.

The data from morphology and DNA sequences both support recognition of this individual as a species distinct from any other in the genus, but we feel more specimens are needed to allow an adequate description. A return trip in March 2022 to search for more specimens was unsuccessful, but the site is readily accessible and the habitat is protected, so it is likely that more individuals can be obtained in the future.

##### ﻿﻿ Anillinus simplex sp. nov.

Taxon classificationAnimaliaColeopteraCarabidae

C68D5F83-74E8-538E-83D7-671886A3EFD7

https://zoobank.org/CA863666-9208-48EC-8AB0-B2E006B1F0E9

**Figure 33.**
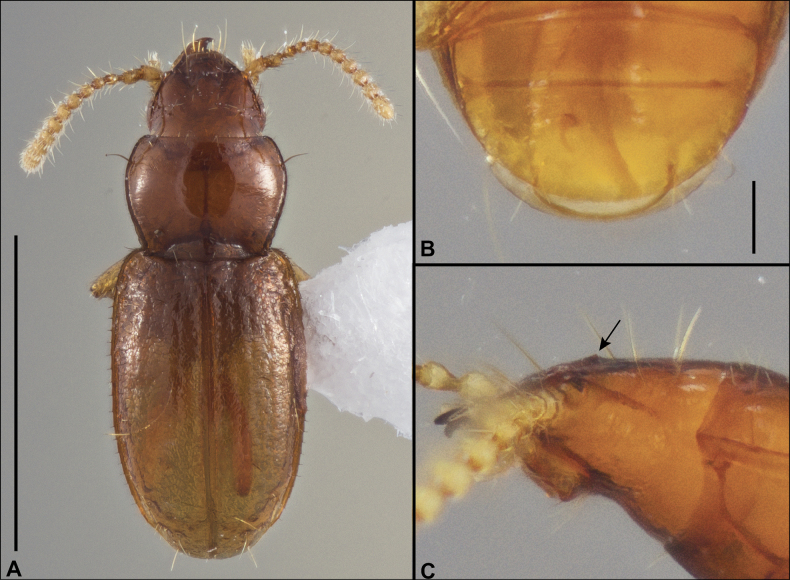
*Anillinussimplex* sp. nov. **A** dorsal habitus, abdomen removed for DNA extraction **B** last abdominal ventrite, ventral aspect **C** head, left lateral aspect (black arrow = frontoclypeal horn). Scale bars: 1 mm (**A**); 0.1 mm (**B**).

Figs 21E , 24E , 29 , 30G–I , 33A–C

###### Type material.

***Holotype male***: (**USNM**), point mounted, with abdominal ventrites glued to point and genitalia in Euparal on microslide pinned beneath specimen. Original label: “USA: SC, York Co. Kings Mountain S.P. 35.1307, -81.3649. 26.September.2020. C.W. Harden. Under rock near stream.” “[QR Code] CLEMSON-ENT CUAC000185896” “Harden DNA Voucher CWH-247 Anill. ‘kingsmtnsp1’ M Ext. 30/September/2020 [green-bordered label].” “HOLOTYPE *Anillinussimplex* Harden & Caterino [orange cardstock]”

GenBank: OR853342, OR839331, OR839570, OR837888, OR838032, OR838222.

***Paratypes*** (*n* = 46, ADGc, CMNH, CNC, CUAC, FSCA, LSAM, NCSU, NHMUK, OSAC, USNM, VMNH). **USA** • **South Carolina** • **York Co.** • Kings Mountain State Park; • 1 ♂; same data as holotype; CUAC000170024, CWH-248; CUAC; • 1 ♀; same data as holotype; CUAC000170025, CWH-249; CUAC; • 8 ♂, 2 ♀; same data as holotype; CUAC000170026 to CUAC000170035; CNC, CUAC; • 3 ♂; same data as holotype; ADGc; • 12 ♂, 12 ♀; 35.13198, -81.36608; 26 Sep. 2020; C.W. Harden leg.; Berlese, deep litter and topsoil, oak, maple, sourwood, pine; CUAC000170036 to CUAC000170059; CMNH, CNC, FSCA, LSAM, OSAC, USNM, VMNH; • 1 ♀; 35.13062, -81.36439; 26 Sep. 2020; C.W. Harden leg.; Berlese, litter near stream, mesic hardwoods; CUAC00017060; VMNH; • 2 ♂, 1 ♀; 35.13018, -81.36205; 23 Dec. 2021; C.W. Harden leg.; under embedded rock; CUAC000167147 to CUAC000167149; NCSU; • 1 ♂, 2 ♀; 35.1301, -81.3637; 23 Dec. 2021; C.W. Harden leg.; Berlese, soil near seepage; CUAC000170061 to CUAC000170063; NHMUK.

GenBank accession numbers for paratypes: OR839571, OR853339, OR839328, OR839572, OR837889, OR838033, OR838223.

###### Other material

**(*n* = 30, CUAC, CWHc, NCSU). USA** • **North Carolina** • 3 ♂, 5 ♀; **Gaston Co.**; Crowders Mountain State Park, near Linwood parking; 35.2417, -81.2717; 29 Apr. 2023; C.W. Harden leg.; Berlese, sifted duff/litter; NCSU; • 2 ♂; same data as previous; under embedded rock; CUAC000182312 and CUAC000182313, CWH-510 and CWH-511; CUAC; • 1 ♂; **Gaston Co.**; Crowders Mountain State Park; Ridgeline Trail at Road 1104; 35.1977, -81.3152; 29 Apr. 2023; C.W. Harden leg.; under embedded rock; CUAC000182314, CWH-512; CUAC; • 3 ♂; **Gaston Co.**; Crowders Mountain State Park; Ridgeline Trail at Road 1104; 35.1977, -81.3152; 29 Apr. 2023; C.W. Harden leg.; under embedded rock; CWHc; • 1 ♀; **Gaston Co.**; Crowders Mountain State Park, near Pinnacle, open piney ridge; 35.2011, -81.3148; 29 Apr. 2023; C.W. Harden leg.; under rock; CWHc; • 1 ♂; **Gaston Co.**; Crowders Mountain State Park, near Pinnacle, recently washed gully; 35.1993, -81.3152; 29 Apr. 2023; C.W. Harden leg.; under rock; CWHc; • 3 ♂; **Gaston Co.**; Crowders Mountain State Park, near Pinnacle, recently washed gully; 35.2031, -81.3144; 29 Apr. 2023; C.W. Harden leg.; under rock; CWHc; • **South Carolina** • **York Co.** • 7 ♂, 3 ♀; Kings Mountain State Park; 35.13198, -81.36608; 26 Sep. 2020; C.W. Harden leg.; Berlese, deep litter and topsoil, oak, maple, sourwood, pine; CWHc; • 1 ♀; Bethany; 35.116, -81.305; 9 Feb. 1998; J.C. Ciegler leg.; in leaf litter; CUAC.

###### Diagnosis.

Males of *A.simplex* are the only members of the *valentinei* group known to lack ventral adhesive setae on protarsomere 2, but this character is difficult to confirm without strong magnification. From most anillines occurring in South Carolina, members of *A.simplex* can be recognized by their small size (ABL < 1.65 mm) and effaced microsculpture on the sides of the vertex. The median lobe of *A.simplex* is distinctive, with a short, thick well-sclerotized curved flagellum in the internal sac and the absence of other well-sclerotized structures (Fig. 30I). Some individuals of *A.murrayae* from southern Pickens Co. overlap in size with members of *A.simplex* but are easily distinguished by the different male and female genitalia (Figs 21D, 30J–N).

###### Description.

***Habitus*** Body small (ABL = 1.45–1.64 mm), moderately convex, slightly ovoid, robust (EW/ABL = 0.35–0.38) (Fig. 33A). ***Integument*** Dorsal microsculpture of forebody variable. In specimens from the type locality, microsculpture is effaced from most of the pronotum, present only along extreme outer margins, and weakly impressed there, and absent from large portions on both sides of the vertex posteriorly. In specimens from Crowders Mountain, the pronotum is entirely covered in strong, easily visible isodiametric microsculpture, and the smooth patches on the vertex are smaller. ***Head***HW/PW = 0.71–0.77. Three pairs of supraorbital setae present, outer posterior pair shorter than other two. Frontoclypeal horn well-developed (Fig. 33C). ***Pronotum*** Relatively short (PL/ABL = 0.22–0.24) and broad (PW/EW = 0.80–0.85), sides evenly convergent or slightly sinuate before slightly narrowed posterior angles (PbW/PW = 0.73–0.78). ***Elytra*** Slightly ovoid, widest approximately middle, length variable (EL/ABL = 0.51–0.57). Umbilicate punctures relatively large and conspicuous. Striae weakly impressed, difficult to trace. ***Legs*** Profemora of males unmodified; protarsomere 1 of males expanded and with inner margin spinose, protarsomere 2 unmodified and apparently lacking ventral adhesive setae in most specimens (a single adhesive seta observed in one male). Mesofemora of males unmodified. Metafemora of males with patch of coarse papillate microsculpture medially on posterior face. ***Abdominal ventrites*** Unmodified in either sex (Fig. 33B). ***Male genitalia*** Ring sclerite (Fig. 24E) moderately small (RL/ABL = 0.28) and asymmetrical, strongly narrowed anteriorly. Median lobe (Fig. 30I) evenly curved, slightly twisted dorsally from level of basal lobes, gradually expanded in width from base to apex. Ventral margin slightly expanded, asetose. Apex small and evenly rounded. Left side of base with semicircular excavation visible in left posterolateral aspect, with carina extending distally from the excavation to approximately middle. Internal sac with well-sclerotized flagellum short and stout, evenly curved, with one short basal extension. Ventral surface of internal sac with field of weakly sclerotized teeth. Sides of internal sac near ostium appearing grooved with curved parallel lines. Ostial plate on left side, small and weakly sclerotized. Right paramere narrow, with four long apical setae (Fig. 30H). Left paramere conchoidal, with four ventral subapical pores, the apical two bearing short setae (Fig. 30G). ***Female genitalia*** Spermatheca long, gradually and slightly enlarged distally, stem slightly sinuate before basal curve, which is rotated perpendicular from rest of stem (Fig. 21E). Spermathecal duct short and not coiled.

###### Distribution.

Known only from Kings Mountain, a short linear monadnock spanning from York County, South Carolina to Gaston County, North Carolina (Fig. 29).

###### Sympatry.

This species has been collected with *A.montrex* and *A.cornelli* underneath embedded rocks. *Serranillusmonadnock* sp. nov. also occurs at Kings Mountain State Park.

###### Natural history.

Specimens have been collected in February, April, September, and December, from underneath embedded rocks on sandy clay rich soil near an ephemeral stream and through Berlese extraction of sifted litter and soil.

###### Species status justification.

The male and female genitalic characters are unique within the genus, and the DNA sequence data indicate *A.simplex* is most closely related to *A.cornelli*, *A.castaneus*, and “South Carolina, Chestnut Ridge”, all of which differ markedly in male genitalic characters, especially by possessing several sclerotized spines in the internal sac.

###### Derivation of species name.

A noun in apposition, from the Latin, meaning “simple”, in reference to the structure of the median lobe of the aedeagus, which lacks the complex sclerotized structures in the internal sac that are present in the other South Carolina species in the *valentinei* group, and the lack of modifications to male protarsomere 2.

##### ﻿﻿‘*elongatus* group’

The five described species of the *elongatus* group were revised by Harden and Caterino (2024) and are discussed and illustrated in more detail in that paper.

###### ﻿﻿ Anillinus arenicollis

Taxon classificationAnimaliaColeopteraCarabidae

Harden & Caterino, 2024

286AE8E1-E94C-5D8C-816F-B709D0E17399

**Figure 34.**
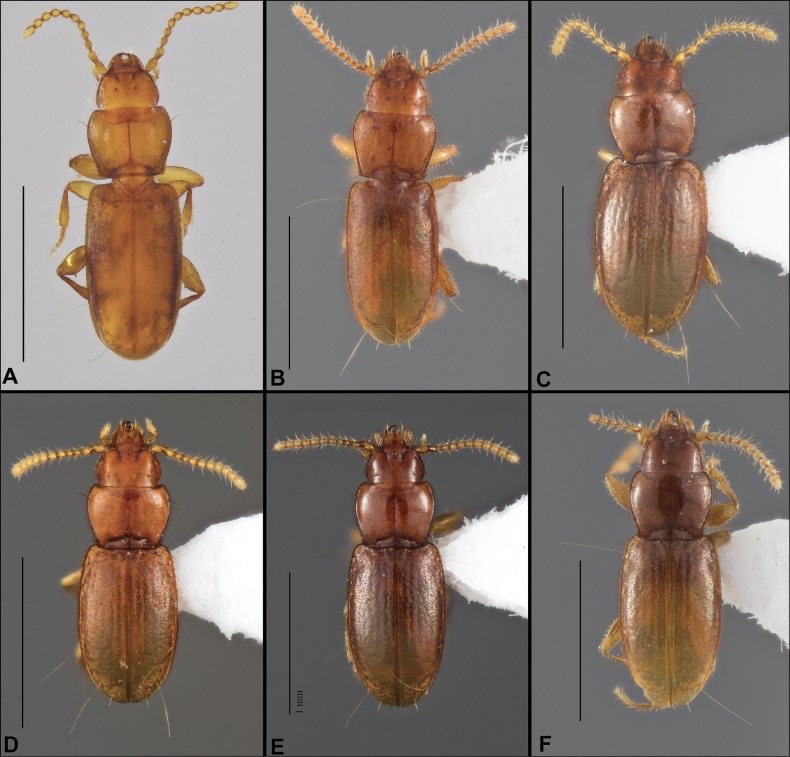
Dorsal habitus of *Anillinus* species in the *elongatus* group and *sinuaticollis* group **A***Anillinusmontrex***B***Anillinusarenicollis***C***Anillinuschoestoea* sp. nov. **D***Anillinusseneca* sp. nov. **E***Anillinusmica* sp. nov. **F***Anillinusmicamicus* sp. nov. Abdomens removed in **B–F** for DNA extraction. Scale bars: 1 mm.

Figs 21M , 24K , 34B

Anillinus arenicollis Harden & Caterino, 2024: 18.

####### GenBank.

OR853123, OR839200, OR838072, OR839197, OR839198, OR853122, OR839199, OR839746, OR837940, OR838098, OR838277.

####### Diagnosis.

Robust, dorsoventrally flattened, and parallel-sided (Fig. 34B). Dorsal surfaces of head and pronotum entirely covered in microsculpture. Males with both protarsomeres 1 and 2 expanded and dentate on inner margin with ventral adhesive setae. Male metafemora swollen with prominent tooth on posterior margin, median lobe of male aedeagus narrow and long with blocky apex that is deflexed ventrally. Female spermatheca (Fig. 21M) long, stem ribbed, not coiled proximally. Spermathecal duct long and coiled.

####### Distribution.

Known from a small area within the boundaries of the Carolina Sandhills National Wildlife Refuge in Chesterfield Co., SC.

####### Sympatry.

Members of this species have not been collected with other species of anillines.

####### Natural history.

The first specimen collected was in a sample of sifted litter collected in February. All other known specimens were collected in buried pipe traps set in sandy soil. Further litter sampling at the only known locality failed to produce further specimens, and members of this species are presumably endogean in habit.

###### ﻿﻿ Anillinus montrex

Taxon classificationAnimaliaColeopteraCarabidae

Harden & Caterino, 2024

26CBA407-8900-5DD7-BEC9-1DD96238EE9E

Figs 2H , 10C, I , 21L , 24L , 34A

Anillinus montrex Harden & Caterino, 2024: 25.

####### GenBank.

OR853113, OR853294, OR839300, OR839565, OR837884, OR838029, OR838218, OR838127, OR839566, OR839299, OR839567, OR837885, OR838030, OR838219.

####### Diagnosis.

Members of this species are strongly flattened dorsoventrally, narrow, and parallel sided (Fig. 34A). Males have the first and second protarsomeres expanded and spinose on inner margin with ventral adhesive setae; the male second protarsomeres of this species are more enlarged than any other known *Anillinus* species (Fig. 10C, I). The male genitalia are also distinctive, with a small apex that is abruptly bisected by the membranous dorsal margin. Female spermatheca (Fig. 21L) with stem coiled proximally, abruptly enlarged distally.

####### Distribution.

Known for certain only from a single hillside above a small stream in Kings Mountain State Park, York Co., SC.

####### Sympatry.

Members of this species have been collected under rocks with *A.simplex* sp. nov.

####### Natural history.

Members of this species are endogean in habit, occurring under deeply embedded rocks at cold times of year. Specimens have been collected in December.

##### ﻿﻿‘*sinuaticollis* group’

###### ﻿﻿ Anillinus choestoea sp. nov.

Taxon classificationAnimaliaColeopteraCarabidae

9996DBAB-EFF1-5E18-A2C9-882245C6D389

https://zoobank.org/E62B04B0-C153-4513-B053-69648A96E3BA

Figs 10E , 11B, D , 12C , 21J , 24I , 34C , 35A–C , 36

####### Type material.

***Holotype male* (USNM)**: point mounted, with abdominal ventrites glued to point and genitalia in Euparal on microslide pinned beneath specimen. Original label: “USA: SOUTH CAROLINA, Oconee Co. Choestoea Park. 34.54616, -83.10479. 21.December.2020. CW Harden. Under small rocks, pine/oak hill. Soft, moist sandy soil.” “[QR code] CLEMSON-ENT CUAC000163546” “Harden DNA Voucher CWH-335 A. ‘choestoea’ M Ext. 1/May/2021 [green-bordered cardstock]” “HOLOTYPE *Anillinuschoestoea* Harden & Caterino [orange cardstock]”

GenBank: OR839239, OR839627, OR838052.

***Paratypes*** (*n* = 4, CUAC). **USA** • **South Carolina** • 2 ♀; same data as holotype; CUAC000163544 and CUAC000163545, CWH-336 and CWH-337; • 1 ♂; **Oconee Co.**; Choestoea Park; 34.54318, -83.09894; 21 Dec. 2020; C.W. Harden leg.; Under rock in *Camponotus* nest; CUAC000163543, CWH-310; • 1 ♀; **Oconee Co.**; Choestoea Park; 34.5477, -83.1052; 19 Feb. 2022; C.W. Harden leg.; Under rock; CUAC000163547.

GenBank accession numbers for paratypes: OR853202, OR839240, OR839608, OR837897, OR838043, OR838232, OR839628, OR839629.

####### Diagnosis.

**Figure 35.**
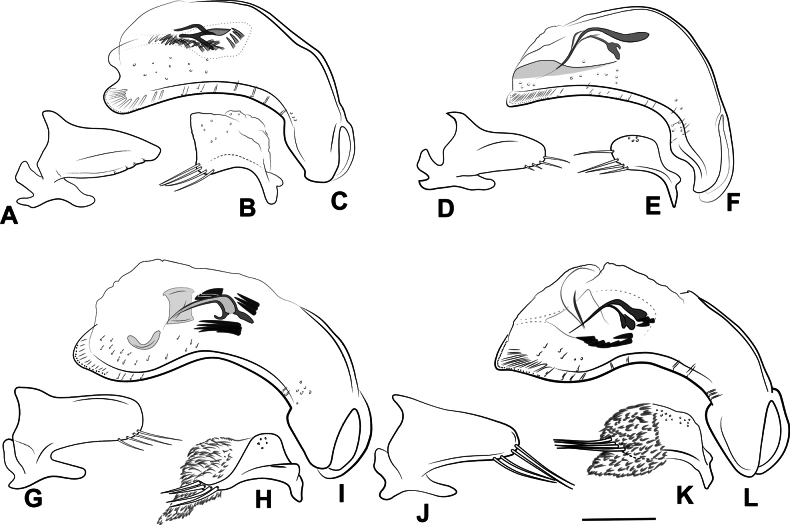
Male genitalia of *Anillinus* species in the *sinuaticollis* group **A–C***Anillinuschoestoea* sp. nov. **D–F***Anillinusseneca* sp. nov. **G–I***Anillinusmica* sp. nov. **J–L***Anillinusmicamicus* sp. nov. Median lobe of aedeagus in right dorsolateral (**C, I, L**) or right lateral (**F**) aspect. Left parameres (**A, D, G, J**) and right parameres (**B, E, H, K**) in left and right lateral aspects, respectively. Scale bar: 0.1 mm.

Compared to other members of the *sinuaticollis* group, the habitus of this species is broader (EW/ABL 0.37 or 0.38) and more convex (Fig. 34C). The microsculpture on the pronotum is more extensive, present anteriorly and extending onto the disc in some specimens. The aedeagus is also unique (Fig. 35C), most notably the dorsally expanded apex of the median lobe and the short, dorsally rotated flagellum of the internal sac.

####### Description.

***Habitus*** (Fig. 34C) ABL = 1.61–1.81 mm, males (1.81 mm) larger than females (1.61–1.65 mm), slightly convex and ovoid (EW/ABL = 0.37–0.38). ***Integument*** Irregular isodiametric microsculpture present anteriorly on pronotum, becoming effaced posteriorly and indistinguishable from surface rugosity, which is strong; microsculpture present across entire dorsal surface of head. Dorsal microsculpture is stronger in males than in females. ***Head***HW/PW = 0.74–0.78. Antennomeres IV–X moniliform. Labrum shallowly emarginate anteriorly. Frontoclypeal horn present, well developed. Three pairs of supraorbital setae present, outer posterior pair shorter than other two. Mentum with median pair of setae posterior to bead of mentum tooth, which is small and subtriangular. ***Pronotum*** Strongly constricted basally in females (Pbw/PW = 0.73–0.74), less so in males (Pbw/PW = 0.78); short in both sexes (PL/ABL = 0.22–0.24). Moderately broad (average PW/EW = 0.81). Sides straight or slightly sinuate before obtuse hind angles; 2–4 basal serrulations. ***Elytra*** Moderately to markedly ovoid, more so in females than in males; moderately convex; relatively long (EL/ABL = 0.55–0.57); humeri not sloped; inner two striae well impressed, traces of two or three additional striae visible. ***Legs*** Protarsi of males with protarsomere 1 expanded and spinose on inner margin, with adhesive setae ventrally, protarsomere 2 unmodified and without adhesive setae (Fig. 10E). Metafemora of males (Fig. 12C) not strongly swollen, without prominent tubercle or tooth on posterior margin. ***Abdominal ventrites*** Unmodified in either sex. ***Male genitalia*** Aedeagus relatively small (RL/ABL = 0.24). Median lobe (Fig. 35C) not twisted, evenly curved; dorsal margin sclerotized for ~1/2 its length; ventral margin without setae; apex large and broadly rounded, produced dorsally into lightly sclerotized lobe subequal to apex in size and shape; internal sac with flagellum rotated dorsally so that it is viewed through base in right lateral view and appears as a complex sclerotized structure; in dorsal view, flagellum is short and evenly curved, broadly “open” laterally, not closed at apex, with elongate basal extension; rows of lightly sclerotized teeth present along left side of internal sac beside flagellum and ostium; texture of internal sac slightly scaly at ostial opening. Right paramere (Fig. 35B) lightly sclerotized and quadrate, apical margin blunt and enlarged, with four moderately long setae basally. Left paramere (Fig. 35A) subtriangular, with four pores along lower margin near apex, without setae. ***Female genitalia*** Spermatheca long, gradually enlarged distally, stem coiled proximally (Fig. 21J); duct damaged in all specimens examined.

####### Distribution.

**Figure 36.**
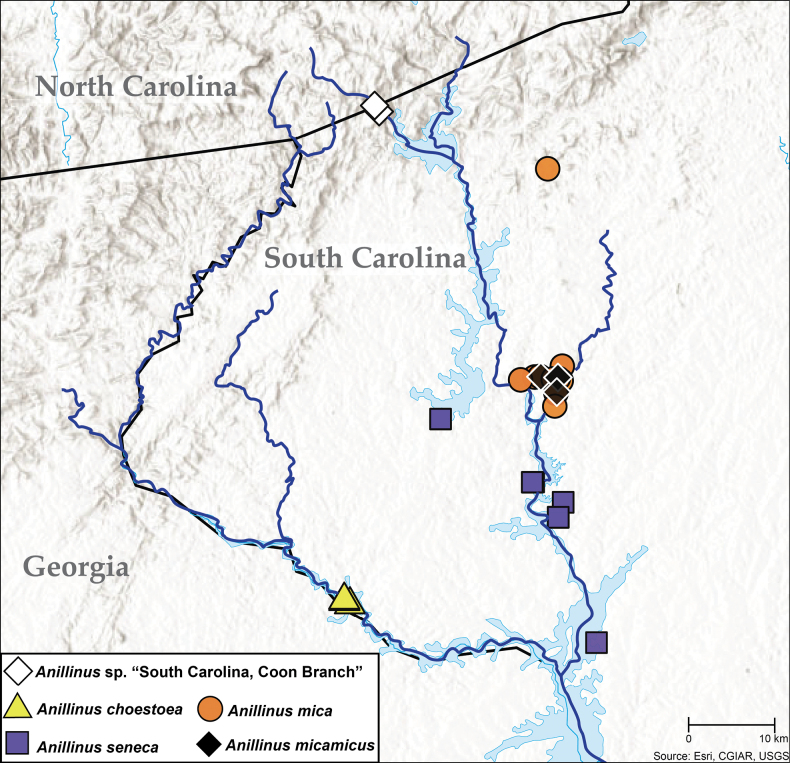
Distribution map of *sinuaticollis*-group species of *Anillinus* in South Carolina. Data are from Harden (2024). Blue lines show modern and pre-inundation courses of the Tugaloo River and Seneca River and their major tributaries. The confluence of the two rivers becomes the Savannah River.

Known from a small area of Choestoea Park in Oconee Co., SC, located along the former course of the Tugaloo River, currently inundated by Lake Hartwell (Fig. 36).

####### Sympatry.

This species has not been collected in association with other anilline species.

####### Species status justification.

The genitalic morphology of males and females of this species is unique within the genus. DNA sequence data indicate that *A.choestoea* is most closely related to geographically distant members of the *sinuaticollis* group, all of which differ from *A.choestoea* in external structure and male genitalic characters.

####### Natural history.

Known from endogean microhabitats. One specimen was collected in the galleries of *Camponotus* ants under a large rock. The remaining specimens were collected underneath smaller rocks embedded in sandy clay rich soil without evidence of *Camponotus* galleries. It is unlikely that the species is closely associated with ant hosts. Specimens were collected in December and February.

####### Derivation of species name.

From the type locality, Choestoea Park, to be treated as a noun in apposition.

####### Notes.

*Anillinuschoestoea* belongs to a subclade that is otherwise comprised of western species. All share a similar flagellum shape that is short and rotated dorsally so that in lateral aspect it is viewed through the base.

###### ﻿﻿ Anillinus mica sp. nov.

Taxon classificationAnimaliaColeopteraCarabidae

5D6DCB48-F598-51D8-B65A-04943E091B28

https://zoobank.org/10A909F3-814E-4576-9F93-14B8EB0173C3

Figs 10B , 12A , 21I , 24G , 25A , 34E , 35G–I , 36

####### Type material.

***Holotype male* (USNM)**: point mounted, with abdominal ventrites glued to point and genitalia in Euparal on microslide pinned beneath specimen. Original labels: “USA: SC, Pickens Co. Clemson Experimental Forest Waldrop Stone Falls. 34.7393, -82.8205. 8.x.2021. CW Harden. Under rock on steep slope near falls.” “[QR code] CLEMSON-ENT CUAC000163558” “Harden DNA Voucher CWH-403 *Anillinus* ‘caterino’ M Ext. 19-December-2021 [green-bordered cardstock]” “HOLOTYPE *Anillinusmica* Harden & Caterino [orange cardstock]”

GenBank: OR853287, OR839293, OR838076.

***Paratypes*** (*n* = 18, CUAC, USNM). **USA** • **South Carolina** • 2 ♂; **Pickens Co.**; Keowee WMA, near Todd Creek Falls; 34.74986, -82.81467; 6 Sep. 2020; C.W. Harden leg.; Under embedded rock on dry soil, mixed woods; CUAC000163551 and CUAC000163552, CWH-230 and CWH-231; • 1 ♂; **Pickens Co.**; Central, ~3 mi N Clemson; 34.7251, -82.8248; 2 Oct. 2020; C.W. Harden leg.; Buried pipe trap, sandy clay mica-rich soil; CUAC000134435; USNM; • 1 ♂; same locality as previous; 14 Aug. to 24 Oct. 2021; C.W. Harden leg.; Buried pipe trap, clay soil, trap 07; CUAC000170072; USNM; • 1 ♂; same locality as previous; 24 Oct. 2021 to 10 Apr. 2022; C.W. Harden leg.; Buried pipe trap, mica-rich sand/clay. Trap 05; CUAC000170073; • 1 ♂, 1 ♀; **Pickens Co.**; 5 mi N Clemson; 34.7252, -82.8245; 8 Nov. 2016; M & P Caterino leg.; floated from soil; CUAC000163553 and CUAC000163554, MSC-2458 and MSC-2466; • 1 ♂, 1 ♀; **Pickens Co.**; Clemson Experimental Forest, Issaqueena Lake Rd., West side; 34.74136, -82.86541; 12 Nov. 2020; C.W. Harden leg.; Underside of rock, clay soil; CUAC000163555 and CUAC000163556, CWH-333 and CWH-334; • 1 ♂; **Pickens Co.**; Clemson Experimental Forest, Waldrop Stone area; 34.73603, -82.81725; 12 Nov 2020; C.W. Harden leg.; Under large rock at lake edge; CUAC000163557, CWH-328; • 3 ♂, 3 ♀; **Pickens Co.**; Clemson Experimental Forest, Waldrop Stone Creek; 34.7395, -82.8270; 11 Sep. 2021; C.W. Harden leg.; Under embedded rock on stream bank; CUAC000163559 to CUAC000163564, CWH-409 to CWH-414; • 1 ♀; **Pickens Co.**; Clemson Experimental Forest; 34.7431, -82.8481; 14 Oct. 2019; C.W. Harden leg.; Soilwash-flotation berlese, mesic oak-pine woods, dark sandy soil near stream; CWH-057, CUAC000168224; • 1 ♀; **Pickens Co.**; Clemson Experimental Forest; 34.74265, -82.84167; 23 Nov. 2019; C.W. Harden leg.; On underside of large embedded rock during rain, oak-hickory woods, clay soil; CUAC000168225, CWH-060.

GenBank accession numbers for paratypes: OR839739, OR839745, OR839626, OR853280, OR839410, OR853281, OR839413, OR853283, OR838130, OR853110, OR853285, OR839292, OR839553, OR837876, OR838021, OR838209, OR838124, OR853286, OR839554, OR837877, OR838022, OR838210, OR853288, OR839294, OR838079, OR853289, OR839288, OR839289, OR839290.

####### Other material

**(*n* = 5, CWHc, CUAC). USA** • **South Carolina** • 1 ♂; **Pickens Co.**; Nine Times Preserve; 34.946, -82.806; 10 Nov. 2019; C.W. Harden leg.; On underside of embedded rock, steep rocky ditch, oak-hickory woods; CUAC000168226, CWH-039; CUAC • 2 ♂; **Pickens Co.**; 3 miles N Clemson; 34.7252, -82.8247; 4 July to 19 December 2022; C.W. Harden leg; buried pipe trap, trap 07; CWHc; • 1 ♀; **Pickens Co.**; Clemson Experimental Forest.; 34.7431, -82.8481; 14 Oct. 2019; C.W. Harden leg.; Soilwash-flotation berlese, mesic oak-pine woods, dark sandy soil near stream; CWH-056, CUAC000163565; CUAC; • 1 ♂; **Pickens Co.**; Clemson Experimental Forest, Pike Road, south of inlet; 34.7116, -82.828; 1 Jun 2023; C.W. Harden and J.R. LaBonte; under rock; CWHc.

####### Diagnosis.

Among members of the *sinuaticollis* group, males are recognized by the modified hind femora (Fig. 12A) bowed anteriorly and triangularly produced posteriorly, with a blunt tuberculate projection in the distal 1/3. The male genitalia (Fig. 35G–I) are similar to those of *A.micamicus*, but differ in the more rounded shape of the apex of the median lobe and the presence of two well-sclerotized strips along the ostium of the internal sac.

####### Description.

***Habitus*** (Fig. 34E) ABL 1.60–1.89 mm, avg. 1.82 mm, *n* = 7), males larger than females (male mean ABL = 1.85 *n* = 5; female mean ABL = 1.74, *n* = 2). Body moderately depressed dorsoventrally and parallel-sided, relatively elongate (EW/ABL = 0.35–0.37). ***Integument*** Irregular isodiametric microsculpture weakly impressed at anterior angles of pronotum, absent from disk, which is slightly rugose; microsculpture distinct on dorsal surface of head except for sides at base of vertex, where it is stretched and weak or entirely absent. ***Head***HW/PW = 0.74–0.80, wider in females than in males. Antennomeres IV–X moniliform, slightly clavate. Labrum slightly emarginate. Frontoclypeal horn present, well developed. Three pairs of supraorbital setae present. ***Pronotum*** Variable in length (PL/ABL = 0.23–0.29), broader in males (PW/EW = 0.82) than females (0.78–0.79). Moderately constricted basally (Pbw/PW = 0.75–0.80). Sides slightly sinuate before obtuse hind angles; 3 or 4 basal serrulations present. ***Elytra*** Moderately depressed and parallel sided, not markedly elongate (average EL/ABL = 0.55); humeri not sloped; 3 or 4 weakly impressed striae present; without prominent subapical plica. ***Legs*** Profemora of males not modified; protarsi of males with first tarsomere enlarged and spinose on inner margin (Fig. 10B), with adhesive setae ventrally, second tarsomere unmodified and without adhesive setae. Mesotrochanters of males unmodified. Metafemora of males modified: anterior margin arcuately swollen, posterior margin arcuate proximally towards broad, triangular tuberculate projection in distal 1/2; with several long setae along posterior margin (Fig. 12A). Female legs unmodified. ***Abdominal ventrites*** Unmodified in either sex. ***Male genitalia*** Ring sclerite relatively large (RL/ABL = 0.30), subtriangular and slightly asymmetrical in anterior 1/2. Median lobe (Fig. 35I) arcuate, not strongly twisted from plane of basal lobes; dorsal margin lightly sclerotized for ~ 1/2 of its length; ventral margin without setae, deeply arcuate medially, with shallow sinuation just before apex, which is broadly rounded. Internal sac with flagellum not rotated dorsally, relatively small, and abruptly curved past enlarged basal area; sides lightly sclerotized, “open” laterally; three groups of long, dark spines present near flagellum on left side of internal sac, variable in number; two sclerotized straps present at ostium, one on left side appearing as a broad, vertical strip and the other ventrally, appearing as a U-shape just beyond apex of flagellum in repose. Right paramere (Fig. 35H) partially membranous and enclosed in a feathery sheath, with four setae on apical margin. Left paramere (Fig. 35G) slightly quadrate, with apical margin relatively broad; four long setae present ventrally near apex. The male from the Nine Times Preserve has the dorsal margin of the median lobe nearly straight proximally, giving the organ a more triangular shape overall; the shape of the flagellum and additional sclerites are identical to those in males of other populations. ***Female genitalia*.** Bursa copulatrix with sclerotized folds. Spermatheca long, gradually enlarged distally, forming a loose coil proximally (Fig. 21I). Spermathecal duct short and slightly curved, not coiled.

####### Distribution.

Known only from Pickens Co., SC, from Nine Times Forest south to the historic course of the Twelve Mile River north of Clemson (Fig. 36).

####### Sympatry.

Collected under rocks in association with *A.murrayae* and *Anillinusmicamicus* sp. nov. The ranges of *Serranillusdunavani* and *Anillinus* sp. “South Carolina, Waldrop Stone” overlap with this species, but have so far not been collected in association.

####### Natural history.

Members of this species are endogean in habit. Specimens examined were collected from beneath rocks, buried pipe traps and soil washing. Hand collected specimens were found in May, September, October, and November. Teneral specimens were hand collected in September, indicating that immature stages occur in the Spring and Summer.

####### Species status justification.

The combination of external characters is unique within the genus, and the male genitalia are distinct from those of any other described species. The closely related and morphologically similar species *A.micamicus* sp. nov. shows consistent morphological differences in the characters of the male hind legs and genitalia, and sampled individuals of the two species are reciprocally monophyletic in our molecular phylogeny despite occurring together in syntopy under the same rocks. This provides strong support for the hypothesis that the two species are reproductively isolated.

####### Derivation of species name.

A noun in apposition, named for the mineral mica, which is conspicuous in the soils at most localities where this species has been collected.

###### ﻿﻿ Anillinus micamicus sp. nov.

Taxon classificationAnimaliaColeopteraCarabidae

9E68D9D2-42FA-5185-AED0-7243C1198316

https://zoobank.org/8230753F-5ECB-4A81-A70B-254DAA91B2FE

Figs 12B , 21H , 24H , 25A , 34F , 35J–L , 36

####### Type material.

***Holotype male* (USNM)**: point mounted, with abdominal ventrites glued to point and genitalia in Euparal on microslide pinned beneath specimen. Original labels: “USA: SC, Pickens Co. Clemson Experimental Forest Waldrop Stone Falls. 34.7393, -82.8205. 8.x.2021. CW Harden. Under rock on steep slope near falls.” “[QR code] CLEMSON-ENT CUAC000168229” “Harden DNA Voucher CWH-402 *Anillinus* “wildcat” M Ext. 19-December-2021 [green-bordered cardstock]” “HOLOTYPE *Anillinusmicamicus* Harden & Caterino [orange cardstock]”

GenBank: OR853291, OR839296, OR838075.

***Paratypes*** (*n* = 4; CUAC). **USA** • **South Carolina** • **Pickens Co.** • 1 ♂, 1 ♀; same data as Holotype; CUAC000168228 and CUAC000168230, CWH-404 and CWH-405; • 1 ♂; Clemson Experimental Forest; 34.74265, -82.84167; 23 Nov. 2019; CW Harden leg.; On underside of large embedded rock during rain, oak-hickory woods, clay soil; CUAC000168227, CWH-061; • 1 ♂; 3 mi N Clemson; 34.7252, -82.8247; 24 Oct. 2021 to 10 Apr. 2022; CW Harden leg.; Buried pipe trap. Mica-rich sand/clay. Trap 07; CUAC000170085.

GenBank accession numbers for paratypes: OR853293, OR839298, OR839414, OR837797, OR837958, OR838148, OR853292, OR839297, OR838077, OR853290, OR839295, OR838078, OR838252.

####### Diagnosis.

Closely similar to *A.mica*, differing in: metafemora of males not strongly modified (Fig. 12B); the shape of the median lobe (Fig. 35L), especially the sinuate ventral margin near the apex and the armature of the internal sac, which lacks the two distinct saddle-like sclerites present in *A.mica*. The single known female of *A.micamicus* has a spermatheca with a larger proximal coil and slightly shorter stem beyond the coil, and lacks sclerotized folds in the bursa.

####### Description.

***Habitus***ABL = 1.77–1.93 mm, average = 1.87 ± 0.07 mm. Moderately flattened dorsoventrally, body relatively narrow (average EW/ABL = 0.35) (Fig. 34F). ***Integument*** Isodiametric microsculpture present on most of dorsal surface of head, stretched and weakly impressed on center of vertex and sides at base in some specimens; absent from disc of pronotum, but present along all outer margins. ***Head***HW/PW = 0.75–0.80; antennomeres IV–X moniliform; frontoclypeal horn small, inconspicuous in lateral view; labrum shallowly emarginate; three pairs of supraorbital setae present. ***Pronotum***PL/ABL = 0.24, PW/EW = 0.81–0.84; Moderately constricted posteriorly (Pbw/PW = 0.76–0.80); sides slightly sinuate before the slightly obtuse hind angles; 3 or 4 basal serrulations present. ***Elytra*** Dorsoventrally flattened, parallel-sided; relatively long (EL/ABL = 0.56–0.57); humeri not sloped; with traces of 3–5 weakly impressed striae; without prominent subapical plica. ***Legs*** Profemora of males unmodified; protarsi of males with first protarsomere moderately expanded and spinose in inner margin, with ventral adhesive setae; second protarsomere of males not expanded and without ventral setae; metafemora of males not swollen, posterior surface weakly tuberculate medially, without prominent projection (Fig. 12B). ***Abdominal ventrites*** Unmodified in either sex. ***Male genitalia*** Ring sclerite large (RL/ABL = 0.29), subtriangular and slightly asymmetrical in anterior 1/2. Median lobe (Fig. 35L) of aedeagus arcuate, enlarged distally, slightly twisted from plane of basal lobes; dorsal margin weakly sclerotized for ~ 1/2 its length; ventral margin asetose, bisinuate before apex, which is abruptly narrowed to a slightly obtuse point. Internal sac with flagellum small and curved, not rotated dorsally, “open” laterally; two groups of small, elongate spines present on left side of sac in repose, in right lateral view appearing as a long U-shaped row below flagellum and a separate small, dark shape behind base of flagellum; left side of ostium with a lightly sclerotized fold, situated above apex of flagellum in right lateral view. Right paramere (Fig. 35K) partially membranous and enclosed in a feathery membranous sheath, with four setae apically. Left paramere (Fig. 35J) subtriangular, with four long subequal setae on ventral margin near apex. ***Female genitalia*** Spermatheca long, gradually enlarged distally, stem coiled proximally (Fig. 21H). Spermathecal duct present, short, slightly curved, without coils. Bursa without sclerotized folds.

####### Distribution.

Known from three nearby localities in southern Pickens Co., SC (Fig. 36).

####### Sympatry.

All of the hand collected specimens were found with *A.mica* under the same rocks. At Waldrop Stone Falls (Fig. 25A), *A.murrayae* was also collected with both species.

####### Natural history.

Specimens have been collected beneath embedded rocks in clay-rich soils and in buried pipe traps. They are presumably endogean in habit. Hand collected specimens were found in October and November.

####### Species status justification.

The combined male genitalic and secondary sexual modifications are unique within the genus, and the DNA sequence data indicate reproductive isolation from other *sinuaticollis*-group species. See justification under *A.mica* above.

####### Derivation of species name.

A noun in apposition created by combining *mica* and *amicus*, meaning friend or companion in Latin, in reference to the repeated cooccurrence of this species and the closely related *A.mica*.

####### Notes.

The following female specimens belong to either *A.mica* or *A.micamicus* (*n* = 5, CUAC): **USA** • **South Carolina** • **Pickens Co.**; • 4 ♀; Central, 3 mi N Clemson; 34.7252, -82.8247; 26 Apr. to 12 Jul. 2020; C.W. Harden & M.S. Caterino leg.; Buried pipe trap baited w/ cheese. Mica-rich clay soil, beech, oak; CUAC00017074 to CUAC000170077; • 1 ♀; same locality as previous; 11 Apr. to 14 Aug. 2021; C.W. Harden leg.; Buried pipe trap. Mica-rich sand/clay. Trap-01; CUAC000170078.

###### ﻿﻿ Anillinus seneca sp. nov.

Taxon classificationAnimaliaColeopteraCarabidae

96A666A3-D522-51C1-92D0-91BA6E8B15AA

https://zoobank.org/3ECB8AD7-7F13-46A2-8500-F72D6A9C2B32

Figs 12D , 21K , 24J , 34D , 35D–F , 36

####### Type material.

***Holotype male* (USNM)**: point mounted, with abdominal ventrites glued to point and genitalia in Euparal on microslide pinned beneath specimen. Original labels: “USA: SOUTH CAROLINA, Oconee Co. Lake Hartwell, Martins Creek Landing, SW of Clemson. 34.6389, -82.8656. 11-January-2020. C.W. Harden. Under embedded rocks in oak-hickory woods near lake.” “[QR Code] CLEMSON-ENT CUAC000168244” “Harden DNA Voucher CWH-121 Anill. sp n ‘martincrk’ Ext. 13/May/2020 [green-bordered cardstock]” “HOLOTYPE *Anillinusseneca* Harden & Caterino [orange cardstock]”

GenBank: OR853323, OR839466.

***Paratypes*** (*n* = 27; CMNH, CUAC, LSAM, USNM, VMNH). **USA** • **South Carolina** • 2 ♂, 2 ♀; same data as holotype; CUAC000168247, CUAC000168248, CUAC000168250, CUAC000168242; CWH-118, CWH-120, CWH-122, CWH-124; CUAC;• 1 ♂, 2 ♀; same locality as holotype; 4 Jan. 2020; C.W. Harden & L.M. Thompson leg.; soilwash flotation Berlese; CWH-115 to CWH-117, CUAC000168239 to CUAC000169241; CUAC; • 2 ♂, 5 ♀; **Oconee Co**.; Martin Creek Landing; 34.6388, -82.8655; 15 May 2022; C.W. Harden and L.M. Thompson leg.; soilwashing; CMNH, USNM; • 3 ♂, 2 ♀; **Oconee Co.**; Martin Creek Landing; 34.6390, -82.8638; 22 Oct. 2021; C.W. Harden leg.; under rock; LSAM, VMNH; • 4 ♂, 4 ♀; **Oconee Co.**; South Cove Park; 34.71165, -82.96594; 30 Jan. 2020; C.W. Harden & L.M. Thompson leg.; Berlese, sifted soil/wood, pine stump near fishing pier; CWH-338 to CWH-345, CUAC000168255 to CUAC000168262; CUAC.

GenBank accession numbers for paratypes: OR839463, OR839465, OR839467, OR853324, OR839469, OR839460, OR839461, OR839462, OR853327, OR839630, OR838053, OR853328, OR839631, OR839632, OR839633, OR853326, OR839621, OR838050.

####### Other material

**(*n* = 40 CUAC, CWHc). USA** • **South Carolina** • 2 ♂; **Anderson Co.**; Clemson Experimental Forest, Big Oaks area near Lake Hartwell; 34.60262, -82.83858; 21 May 2020; C.W. Harden; Under embedded rock in clay soil; CWH-161 and CWH-162, CUAC000168245 and CUAC000168251; CUAC; • 1 ♂, 1 ♀; **Anderson Co.**; Clemson Experimental Forest, Big Oaks area; 34.60225, -82.83893; 21 May 2020; C.W. Harden & L.M. Thompson; Berlese, deep soil by large stump near lake, after flood; CWH-163 and CWH-164, CUAC000168252 and CUAC000168253; CUAC; • 1 ♂; **Anderson Co.**; River Forks Recreation Area; 34.47497, -82.80914; 19 Mar. 2021; C.W. Harden & L.M. Thompson; Berlese, sifted pine stump soil, near lake; CWH-327, CUAC000168254; CUAC; • 2 ♂, 1 ♀; **Anderson Co.**; Clemson Experimental Forest, Big Oaks Area near Lake Hartwell; 34.60225, -82.83893; 21 May 2020; C.W. Harden and L.M. Thompson leg.; deep soil Berlese; CWHc; • 1 ♂, 2 ♀; **Anderson Co.**; Clemson Experimental Forest, Big Oaks; 34.61559, -82.83061; 25 Mar 2020; C.W. Harden leg.; deep soil and litter Berlese; CWHc; • 2 ♂; **Oconee Co.**; Lake Hartwell, Martin Creek Landing, SW of Clemson; 34.6389, -82.8656; 4 Jan. 2020; C.W. Harden & L.M. Thompson leg.; soilwash flotation Berlese; CUAC000168237 and CUAC000168238; CWH-070 and CWH-114; CUAC; • 1 ♂, 2 ♀; same data as previous; CWHc; • 2 ♂; **Oconee Co.**; Lake Hartwell, Martin Creek Landing, SW of Clemson; 34.6389, -82.8656; 4 Jan. 2020; C.W. Harden leg.; On underside of deeply embedded rocks; CUAC000168243 and CUAC000168233; CWH-062 and CWH-063; CUAC; • 3 ♂; **Oconee Co.**; Lake Hartwell, Martin Creek Landing, SW of Clemson; 34.6389, -82.8656; 31 Dec. 2019; C.W. Harden & L.M. Thompson leg.; On underside of deeply embedded rocks; CUAC000168234 to CUAC000168236; CWH-064 to CWH-066; CUAC; • 1 ♂, 1 ♀; **Oconee Co.**; Lake Hartwell, Martin Creek Landing, SW of Clemson; 34.6389, -82.8656; 11 Jan. 2020; C.W. Harden leg.; Under embedded rocks; CUAC000168246 and CUAC000168249; CWH-119 and CWH-123; CUAC; • 1 larva; **Oconee Co.**; Martin Creek Landing; 34.6388, -82.8655; 15 May 2022; C.W. Harden and L.M. Thompson leg.; soilwashing; CWHc; • 2 ♂, 6 ♀; **Oconee Co.**; Martin Creek Landing; 34.6388, -82.8644; 22 Oct 2021; C.W. Harden leg.; under rock; CWHc; • 1 ♂, 2 ♀; **Oconee Co.**; Martin Creek Landing; 34.6390, -82.8638; 22 Oct 2021; C.W. Harden leg.; under rock; CWHc; • 1 ♀; **Oconee Co.**; Martin Creek Landing; 34.6391, -82.8636; 22 Oct 2021; C.W. Harden leg.; under rock; CWHc; • 1 ♀; **Oconee Co.**; Martin Creek Landing; 34.6393, -82.8633; 22 Oct 2021; C.W. Harden leg.; under rock; CWHc; • 2 ♀; **Oconee Co.**; Lake Hartwell, Martin Creek Landing, southwest of Clemson; 34.6389, -82.8656; 4 Jan 2020; C.W. Harden and L.M. Thompson leg.; CWHc; • 1 ♂; **Oconee Co.**; Martin Creek Landing, southwest of Clemson; 34.6389, -82.8656; 13 Feb. 2023; C.W. Harden leg.; under rock; CWHc.

####### Diagnosis.

Males with metafemora not heavily modified, with a median tuberculate area on posterior margin (Fig. 12D), sometimes with a small blunt tooth. Male genitalia distinctive (Fig. 35D–F): median lobe of aedeagus with straight and narrow ventral margin, internal sac without groups of spines, flagellum relatively large and evenly curved.

####### Description.

***Habitus*** Widely variable in body size (ABL = 1.46–1.78 mm, average = 1.63 ± 0.08) and shape, with smaller specimens being more convex and ovoid (Fig. 34D) and larger specimens being flatter and more parallel sided (similar to Fig. 34E). Males and females similarly variable in size (ABL = 1.51–1.78 mm and 1.46–1.78 mm, respectively). Not markedly narrow (average EW/ABL = 0.36). ***Integument*** Dorsal surface of head fully microsculptured; microsculpture coverage of pronotum varying: usually lacking from disc and present across entire anterior margin and along sides, sometimes absent anteriorly except for anterior angles; sometimes distinct across entire anterior 1/4, including part of disc. ***Head***HW/PW = 0.74–79, antennomeres IV–X moniliform; frontoclypeal horn small, but distinct in lateral view; three pairs of supraorbital setae present, outer posterior pair smaller than other two. ***Pronotum*** Size and shape variable: PL/ABL = 0.22–0.24, PW/EW = 0.78–0.87, PbW/PW = 0.73–0.79. Sides either convergent or distinctly sinuate before posterior angles, which are either obtuse or nearly right. ***Elytra*** Variable in shape, either parallel sided and flat or slightly ovoid and convex. EL/ABL = 0.52–0.56. ***Legs*** Profemora of males unmodified. First protarsomere of males dilated and spinose on inner margin, bearing adhesive setae ventrally; second protarsomere of males unmodified. Mesotrochanters of males unmodified. Metafemora of males variable, either slightly enlarged medially with patch of coarse microsculpture (Fig. 12D) or swollen and bearing a blunt tooth on posterior margin. Females without leg modifications. ***Abdominal ventrites*** Unmodified in either sex. ***Male genitalia*** Aedeagus small (RL/ABL = 0.23). Median lobe (Fig. 35F) slightly rotated dorsally from plane of basal lobes, also curved towards left side in apical 1/2; slightly narrowed apically; ventral margin evenly curved, not expanded, without setae; dorsal margin sclerotized for ~ 2/3 its length; apex small, rounded. Internal sac covered in small scales, without spines; flagellum relatively long, not rotated dorsally, sinuate, abruptly narrowed apically, lightly sclerotized, and “open” laterally. Right paramere (Fig. 35E) small, broadly rounded, with four apical setae. Left paramere (Fig. 35D) subtriangular, ventral margin with four apical setae of variable length. ***Female genitalia*** Spermatheca long, abruptly expanded distally, stem with loose proximal coil, nearly straight before enlarged apex which is strongly curved (Fig. 21K). Spermathecal duct short, not coiled.

####### Distribution.

Endemic to South Carolina, known from Oconee and Anderson Counties on both sides of the former Seneca River (currently Lake Hartwell) from Seneca south to River Forks recreation area (Fig. 36).

####### Sympatry.

At the type locality, *S.dunavani* and a large *Anillinus* species belonging to the *valentinei* group (*Anillinus* sp. “South Carolina, Waldrop Stone”) also occur. The other South Carolina members of the *sinuaticollis* group are apparently allopatric with respect to *A.seneca*.

####### Natural history.

Members of this species are endogean, inhabiting mineral soil layers in sandy clay rich soils. They have been collected beneath embedded rocks and through Berlese extraction of soil and washed soil. In samples from the type locality, Laboulbeniales fungi were observed on the dorsal surface of pronotum and elytra of several females and on the abdominal apex in a male. Specimens were collected in January, February, March, May, October, and December.

####### Species status justification.

The male genitalia are unique within the genus. DNA sequence data indicate that *A.seneca* is sister to the pair of *A.mica* and *A.micamicus*, both of which differ markedly in male genitalic characters.

####### Derivation of species name.

A noun in apposition, named for the former Seneca River. The known localities for this species are all near the former course of this river, which was lost due to the construction of Lake Hartwell in the 1950s.

####### Notes.

DNA sequences of individuals from the opposite side of the historic course of the Seneca River in Anderson Co. are divergent from Oconee Co. individuals. There are no differences in genitalic morphology, and the divergence is interpreted as recent intraspecific variation. Still, those Anderson Co. individuals are not made part of the type series, in case future study concludes they are specifically distinct.

###### ﻿﻿ Anillinus sp. “South Carolina, Coon Branch”

Taxon classificationAnimaliaColeopteraCarabidae

A48C9D52-6FF8-5561-A921-18DE87F29682

Figs 21G , 25B , 36

####### Material examined

**(*n* = 3). USA** • **South Carolina** • **Oconee Co.**; Coon Branch Natural Area; • 1 ♂, 1 ♀; 35.020, -83.000; 21 Jun. 2018; B. Owens and C. Carlton leg.; soil flotation; CUAC000168231 and CUAC000168232, CWH-102 and CWH-178; CUAC • 1 ♂; 35.0256, -83.0050; 2 Oct. 2021; C.W. Harden leg.; on soil under large rock.; CWHc.

GenBank: OR853183, OR839228, OR839449, OR837811, OR837968, OR838156, OR839507, OR837843, OR837990, OR838176.

####### Diagnosis.

The male genitalia are similar to those of *A.mica* and *A.micamicus*, but differ in several characters: the shape of the median lobe is more elongate, with the ventral margin less curved and abruptly straightened at apex, which is obtusely angulate; the internal sac has a flagellum that is similar to *A.micamicus*, and has a single group of approximately 12 elongate, well-sclerotized spines on the left side; there is a sclerotized saddle like structure near the ostium, as in *A.mica*, but it is situated dorsal to the level of the flagellum; a pair of parallel rows of small sclerotized teeth run along the left side of the ostium, appearing as a pair of dark curved lines below the saddle like structure. The right paramere is encased in a membranous sheath as in *A.mica* and *A.micamicus*. The left paramere is shaped similar to that of *A.micamicus*, but the inner two setae on the apical margin are much shorter than the outer two, which are elongate but not as much as in *A.mica* and *A.micamicus*. The spermatheca (Fig. 21G) has a stem that is coiled evenly in a spiral; it is evenly enlarged distally. The duct has a single sharp bend just behind the junction with the spermatheca, and is otherwise straight and relatively wide, without coils. The bursa has a few lightly sclerotized regions.

####### Notes.

The data from morphology and DNA sequences indicate that these individuals might represent a species distinct from *A.micamicus*. However, more sampling in the intervening area between Coon Branch and the localities of *A.micamicus* will be necessary to test this hypothesis.

##### ﻿﻿‘*langdoni* group’

###### ﻿﻿ Anillinus cf. nantahala

Taxon classificationAnimaliaColeopteraCarabidae

Dajoz, 2005

01FD5A4E-1C1B-5382-A896-0D968D427555

**Figure 37.**
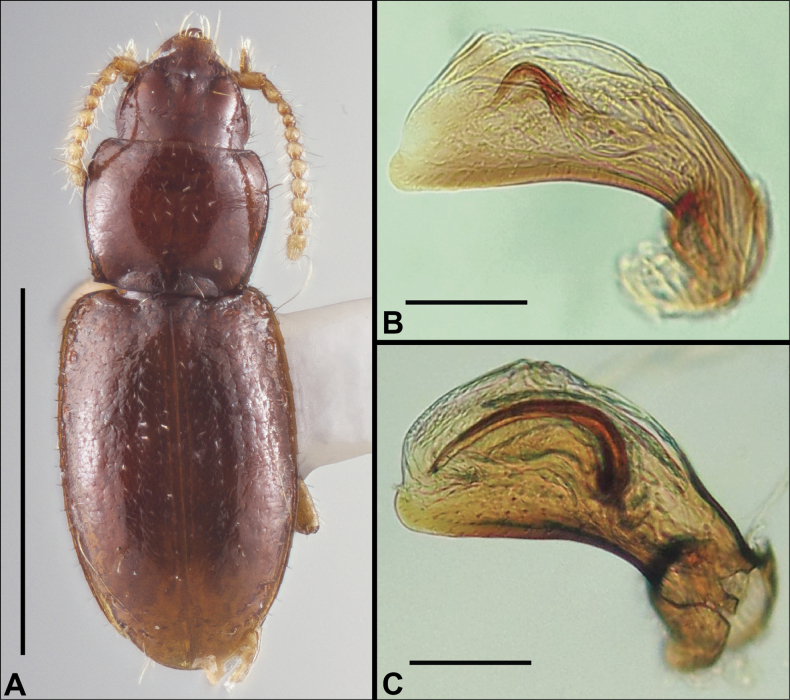
*Anillinus* species in the *langdoni* group **A**Anillinuscf.nantahala, dorsal habitus **B** median lobe, right dorsolateral aspect of *A.nantahala***C** median lobe right dorsolateral aspect of *Anillinus* sp. “Georgia, Brasstown Bald sp. 1”. Scale bars: 1 mm (**A**); 0.1 mm (**B, C**).

**Figure 38.**
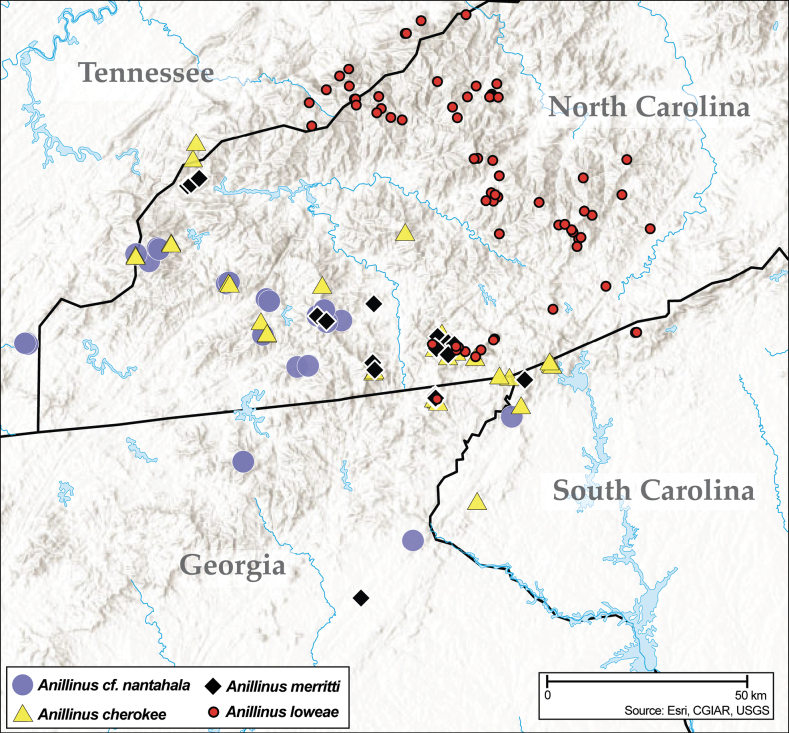
Distribution map of *langdoni*-group and *loweae*-group species that occur in South Carolina. Data are from Harden (2024).

Figs 21O , 37A,B , 38

Anillinus nantahala Dajoz: 2005: 210.

####### Notes on type material.

Dajoz (2005) designated a holotype and an unspecified number of paratypes, all deposited in his personal collection. Upon Dajoz’s death in 2019, his collection was deposited in the Muséum National d’Histoire Naturelle in Paris (Kippenhan 2023). On 11 December 2023, CWH submitted a request for a digital loan of the holotype of *A.nantahala* (request #181113), but as of March 2024 this has not been processed. We have not studied the type material of this species, and our interpretation of the name is explained in the notes section below.

####### Material examined

**(*n* = 148). USA** • **Georgia** • 1 ♂; **Habersham Co.**; Big Panther Creek Trail; 34.68, -83.40; 12 Sep. 1999; W. Reeves leg.; CWHc; • 3 ♂, 1 ♀; **Towns Co.**; Chattahoochee National Forest, Little Bald Mountain, north of Brasstown Bald; 34.8829, -83.8094; 2 Jul. 2020; M. Caterino leg.; CWH-186, CWH-188, CWH-194, CWH-195, CUAC000182284 to CUAC000182287; CUAC; • **North Carolina** • 3 ♂, 2 unsexed; **Cherokee Co.**; Hickory Branch trail; 35.2165, -83.7047; 26 Jul. 2015; S. Myers leg.; SSM59, SSM62 to SSM66, CUAC000182289, CUAC000182290, CUAC000185551, CUAC000182293, CUAC000182294; CUAC; • 32 ♂, 16 ♀; **Clay Co.**; Nantahala National Forest, Tusquitee Bald; 35.1425, -83.7260; 6 Jul. 2021; • 1 ♂; **Clay Co.**; Nantahala National Forest, Shooting Creek Bald trail; 35.0674, -83.6452; 11 May 2020; C.W. Harden and M.S. Caterino leg.; CWH-179, CUAC000182280; • 1 ♀; **Clay Co.**; Nantahala National Forest, Riley Knob, off Highway 64 ca. 8 km northeast of Shooting Creek; 35.0678, -83.6193; 11 May 2020; C.W. Harden and M.S. Caterino leg.; CWH-181, CUAC000182279; • 1 ♂; **Graham Co.**; Nantahala National Forest, Huckleberry Knob; 35.3216, -83.9929; 4 May 2020; M. Caterino and F. Etzler leg.; CWH-283, CUAC000182281; • 1 ♂; **Graham Co.**; Joyce Kilmer Memorial Forest; 35.3467, -83.9688; 20 Jul. 2015; S. Myers leg.; SSM22, CUAC00182288; • 1 ♀; **Graham Co.**; Joyce Kilmer Memorial Forest; 35.3448, -83.9649; 24 Jun. 2015; S. Myers and M. Caterino leg.; SSM37, CUAC000182292; • 2 unsexed; **Graham Co.**; Joyce Kilmer Memorial Forest; 35.3426, -83.9660; 24 Jun. 2015; S. Myers and M. Caterino; SSM38 and SSM61, CUAC000185877 and CUAC000182291; • 1 ♂, 1 ♀; **Graham Co.**; Nantahala National Forest, Teyahalee Bald; 35.2559, -83.8043; 12 Apr. 2022; • 1 ♂, 2 ♀; **Graham Co.**; Nantahala National Forest, Teyahalee Bald; 35.2585, -83.7959; 19 Jul. 2019; • 5 ♂, 3 ♀; **Graham Co.**; Nantahala National Forest, Teyahalee Bald; 35.2598, -83.7970; 12 Apr. 2022; • 1 ♂; **Graham Co.**; Nantahala National Forest, Cherohala Skyway, Stratton Ridge; 35.3382, -84.0249; 4 May 2020; C.W. Harden leg.; • 1 ♀; **Macon Co.**; Nantahala National Forest, off Wayah Road ca. 10 km east of Rte 64; 35.1554, -83.5581; 20 Oct. 2019; C.W. Harden leg.; CWH-054, CUAC000182274; • 3 ♂, 2 ♀; **Macon Co.**; Nantahala National Forest, Wayah Bald Road; 35.1700, -83.5811; 18 Apr. 2020; C.W. Harden leg.; CWH-139 to CWH-142, CWH-167, CUAC000182275 to CUAC000182278, CUAC000185892; • 4 ♂; same data as previous; • 1 ♂; **Macon Co.**; Eight miles west of Franklin; 35.1550, -83.5220; 19 Mar. 1976; Q.D. Wheeler leg.; OSUC441957; OSUC; • 5 ♂, 9 ♀; **Macon Co.**; Wayah Bald; 35.1790, -83.5620; 29 Jun. 2013; T. Lawton leg.; TLc; • **South Carolina** • 1 ♂; **Oconee Co.**; Sumter N.F., near Chattooga River; 34.9170, -83.1166; 5 Sep. 2015; M. and K. Caterino leg.; CWH-494, CUAC000110284; • **Tennessee** • 1 ♂; **Polk Co.**; Cherokee National Forest, Miller Cove; 35.1766, -84.3241; 28 May 2020; C.W. Harden leg.; CWH-349, CUAC000182282; • 1 ♀; **Polk Co.**; Cherokee National Forest, Miller Cove; 35.1758, -84.3246; 28 May 2020; C.W. Harden leg.; CWH-350, CUAC000182283; • 8 ♂, 5 ♀; **Polk Co.**; Cherokee National Forest, John Muir trail, Highway 68 ca. 3 km north of Farner; 35.1771, -84.3298; 23 May 2021; C.W. Harden and K. Ivanov leg.; • 12 ♂, 16 ♀; **Polk Co.**; Cherokee National Forest, John Muir trail, Highway 68 ca. 3 km north of Farner; 35.1771, -84.3298; 7 Nov. 2022; C.W. Harden leg.

GenBank: GU556025, GU556075, MK112078, MK118201, OR830242, OR837795, OR837826, OR837827, OR837844, OR837846, OR837849, OR837956, OR837980, OR837981, OR837991, OR837993, OR837995, OR838110, OR838120, OR838146, OR838167, OR838168, OR838177, OR838179, OR838181, OR838183, OR838292, OR838293, OR839324–OR839327, OR839407, OR839483– OR839486, OR839506, OR839508, OR839510, OR839514, OR839516, OR839522, OR839523, OR839604, OR839637, OR839748, OR839812, OR839813, OR853329–OR853338.

####### Literature records.

This species has previously been reported only from the type locality, Wayah Bald, Macon Co., North Carolina (Dajoz 2005).

####### Diagnosis.

Externally typical of the *langdoni* group (Fig. 37A), with fully developed dorsal microsculpture on the head and pronotum and a moderately convex habitus. The male genitalia (Fig. 37B) are most similar to *A.pusillus*, with the ventral margin narrow and the flagellum short, but unlike *A.pusillus*, the flagellum in *A.nantahala* is sinuate at its apex. Spermatheca with short basal bend, gradually enlarged apically (Fig. 21O). Spermathecal duct long and not coiled. The single individual of this species known from South Carolina has an ABL of 1.73 mm.

####### Distribution.

Relatively widespread in the southern Appalachians from the flank of the Unicoi Mountains in Tennessee to South Carolina, where it is known only from Oconee Co. near the Chattooga River (Fig. 38).

####### Sympatry.

Other species of anillines known from the Chattooga River gorge include *Serranillusdunavani*, *A.cherokee*, *A.merritti*, and *A.murrayae*.

####### Natural history.

The single South Carolina specimen was collected in a sample of sifted litter, as were most of the other specimens examined.

####### Notes.

The description and illustrations of *A.nantahala* are terse, and it is impossible to confidently determine the identity of the species that the name refers to. Four species of *Anillinus* have been collected at the type locality (Wayah Bald, Macon Co., NC), and so the name could apply to any of these. However, the description did specify that members of *A.nantahala* have the dorsal microsculpture on the forebody fully developed, and the species we have chosen to apply the name to is the most abundant and readily collected species at Wayah Bald, and therefore most likely to be encountered by a traveling collector with limited time (as Dajoz was).

###### ﻿﻿ Anillinus sp. "Georgia, Brasstown Bald sp. 1"

Taxon classificationAnimaliaColeopteraCarabidae

AA5B4C53-2777-5D1E-B046-33B62A0CC99E

Fig. 37C

####### Material examined

(*n* = 5). **USA** • **Georgia** • 2 ♂; **Towns Co.**; Chattahoochee National Forest, Little Bald Mountain north of Brasstown Bald; 34.8829, -83.8094; 2 Jul. 2020; M.S. Caterino leg.; CWH-192 and CWH-193, CUAC000182295 and CUAC0001822296; • 2 ♂; **Towns Co.**; Chattahoochee National Forest, Brasstown Bald; 34.8766, -83.8109; 2 Jul. 2020; M.S. Caterino leg.; CWH-196 and CWH-197; CUAC000182297 and CUAC000182298; • **South Carolina** • 1 ♂; **Pickens Co.**; Sassafras Mtn; 21 July 1967; S. Peck and A. Fiske leg.; B-1377A; CMNH.

GenBank: OR837853– OR837855, OR837999, OR838000, OR838186– OR838188, OR839277, OR839520, OR839521, OR839524, OR839525, OR853248, OR853249.

####### Diagnosis.

The only specimen of this species seen from South Carolina is slide mounted and distorted beneath a cover slip. The genitalia are identical to specimens from the vicinity of Brasstown Bald in northern Georgia (Fig. 37C). Individuals from Georgia are externally typical members of the *langdoni* group, measuring 1.63–1.77 mm. The median lobe is simple, with the ventral margin moderately expanded and the apex not deflected, simply rounded. The flagellum is enormous, well-sclerotized and strongly curved, without an apical sinuation; a lightly sclerotized basal piece is apparent ventral to the flagellum.

####### Notes.

Considering the disjunct locality and the absence of this species in any other samples taken from Sassafras Mountain, or anywhere other than the vicinity of Brasstown Bald, the South Carolina record is doubtful.

##### ﻿﻿‘*loweae* group’

###### ﻿﻿ Anillinus cherokee

Taxon classificationAnimaliaColeopteraCarabidae

Sokolov & Carlton, 2008

666FAEAB-8D0C-5DD7-811B-EEAA7AA86343

**Figure 39.**
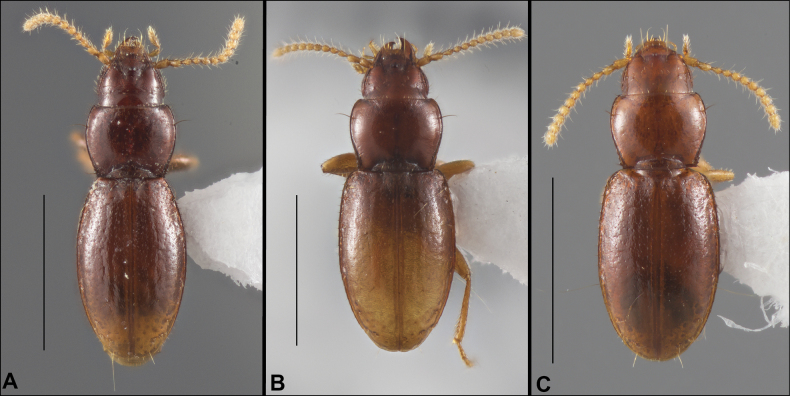
Dorsal habitus of female *Anillinus* in the *loweae* group **A***Anillinusmerritti*, sloped-humeri form (Georgia, Rabun Bald) **B***Anillinusloweae* (Georgia, Rabun Cliffs) **C***Anillinuscherokee* (South Carolina, Coon Branch). Scale bars: 1 mm.

Figs 21P , 24O , 25B , 38 , 39C

Anillinus cherokee Sokolov & Carlton, 2008: 40.

####### Material examined.

***Holotype male*** (**USNM**), point mounted, not dissected, labeled: “USA: NC: Graham Co. Nantahala NF, Joyce Kilmer Memorial, 83°56'03" W 35°21'20" N leaf/log Berlese C. Carlton 05 Apr 2004” “♂” “HOLOTYPE *Anillinuscherokee* sp. n. Sokolov and Carlton des. 2008”

####### Other material

**(*n* = 323). USA** • **Georgia** (**new state record**) • 1 ♂; **Rabun Co.**; Chattahoochee National Forest, Rabun Bald trail; 34.9724, -83.3020; 29 Sep. 2019; C.W. Harden leg.; CWH-026, CUAC000169308; • 1 ♀; **Rabun Co.**; Chattahoochee National Forest, Rabun Bald trail; 34.9716, -83.3013; 29 Sep. 2019; C.W. Harden leg.; CWH-028, CUAC000169307; • 1 ♀; **Rabun Co.**; Chattahoochee National Forest, 0.6 km south of Rabun Bald trailhead; 34.9748, -83.3059; 26 Oct. 2019; C.W. Harden leg.; CWH-096, CUAC000169306; • 4 ♂, 1 ♀; **Rabun Co.**; Rabun Bald, rotten wood debris; 34.967, -83.299; 30 May 1964; H.R. Steeves and J.D. Patrick, Jr. leg.; CMNH; • 1 ♀; **Rabun Co.**; Rabun Bald, rotten wood debris; 34.967, -83.299; 9 Jul. 2014; T. Lawton leg.; TLc; • **North Carolina** • 1 ♂; **Clay Co.**; Nantahala National Forest, Chunky Gal trail; 35.1467, -83.7146; 1 Sep. 2020; P. Wooden and F. Etzler leg.; CWH-506, CUAC000182308; • 2 ♂; **Clay Co.**; Nantahala National Forest, Chunky Gal trail; 35.1471, -83.7144; 6 Jul. 2021; M.S. Caterino leg.; • 1 ♂, 1 ♀; **Graham Co.**; Nantahala National Forest, Teyahalee Bald; 35.2585, -83.7959; 19 Jul. 2019; • 1 ♂; **Graham Co.**; Nantahala National Forest, Teyahalee Bald; 35.2598, -83.7970; 12 Apr. 2022; • 2 ♂; **Graham Co.**; Nantahala National Forest, Stratton Ridge, Cherohala Skyway; 35.3382, -84.0249; 27 Sep. 2020; C.W. Harden leg.; • 1 ♂; **Graham Co.**; Nantahala National Forest, below Stratton Ridge parking; 35.3390, -84.0250; 28 May 2020; C.W. Harden leg.; CWH-191, CUAC000169309; • 1 ♂, 2 ♀; **Graham Co.**; Joyce Kilmer Memorial Forest, 25 mi southwest of Tapoco; 35.357, -83.933; 24 Aug. 1974; J.L. Bengston; CMNH; • 1 ♂; **Graham Co.**; Cherokee National Forest, Wright Creek Trail [*sic*]; 19 Oct. 2007; I. Sokolov leg.; NCSU_ENT00293740; • 1 ♀; **Macon Co.**; Ellicott Rock; 35.0029, -83.1094; 29 Jun. 2015; S. Myers leg.; SSM249, CUAC000170595; • 1 ♂, 1 ♀; **Macon Co.**; Ellicott Rock; 35.0075, -83.1358; 18 Jul. 2015; S. Myers leg.; SSM97 and SSM248, CUAC000169311 and CUAC000170594; • 5 ♂, 7 ♀; **Macon Co.**; Coweeta Hydrological Lab, ca. 13 mi west of Highlands; 35.045, -83.451; 8 Aug. 1965; H.R. Steeves leg.; CMNH; • 8 ♂, 30 ♀; **Macon Co.**; Coweeta Hydrological Lab, ca. 13 mi west of Highlands; 35.045, -83.451; 22 May 1965; H.R. Steeves leg.; • 47 ♂, 63 ♀; **Macon Co.**; Coweeta Hydrological Lab, ca. 13 mi west of Highlands; 35.045, -83.451; 23 May 1965; H.R. Steeves leg.; • 11 ♂; **Macon Co.**; Coweeta Hydrological Lab, ca. 13 mi west of Highlands; 35.045, -83.451; no date; H.R. Steeves leg.; • 2 ♂, 1 ♀; **Macon Co.**; Coweeta Hydrological Lab; 35.045, -85.451; 8 Jun. 1973; W. Suter leg.; • 1 ♂, 7 ♀; **Macon Co.**; Highlands vicinity; 35.05, -83.19; 7 Jul. 1981; J. Pakaluk leg.; • 1 ♂; **Macon Co.**; Turtle Pond Creek, ca. 4 mi west northwest of Highlands; 35.06, -83.26; 8 Aug. 1970; T.C. Barr leg.; • 1 ♂, 1 ♀; **Macon Co.**; Dry Fall, Cullasaja River; 35.067, -83.238; 27 Jun. 1949; J.M. Valentine leg.; • 1 ♂; **Macon Co.**; Jones Gap; 35.0785, -83.2923; 22 Jul. 2015; S. Myers leg.; SSM173, CUAC000169310; • 26 ♂, 31 ♀; **Macon Co.**; 4 mi northwest of Highlands near Buckhorn Gap; 35.0850, -83.2600; 22 Aug. 1982; J. Pakaluk leg.; • 3 ♂, 4 ♀, 2 unsexed; **Macon Co.**; 0.6 mi northeast of Goldmine, California Ridge; 35.110, -83.2710; 14 May 1971; T.C. Barr leg.; • 8 ♂, 2 ♀; **Macon Co.**; 0.2 mi southeast of Old Road Gap; 35.173, -83.726; 15 May 1971; T.C. Barr leg.; • 2 ♂; **Macon Co.**; Nantahala National Forest, Copper Ridge Bald; 35.2348, -83.5596; 15 Sep. 2020; F. Etzler leg.; CWH-504 and CWH-505, CUAC000182306 and CUAC000182307; • 1 ♂; **Macon Co.**; Nantahala National Forest, Cowee Bald; 35.3269, -83.3350; 15 Sep. 2020; F. Etzler leg.; CWH-507, CUAC000182309; • **South Carolina** • 1 ♀; **Oconee Co.**; Sumter National Forest, Doran Creek, off Spy Rock Road; 34.7512, -83.2244; 30 Mar. 2021; C.W. Harden and L.M. Thompson leg.; CWH-357, CUAC000169305; • 3 ♂, 1 ♀; **Oconee Co.**; 7 mi south of NC state line on Highway 107; 34.942, -83.089; 29 May 1983; D.S. Chandler leg.; LSAM0295327 to LSAM0295330; • 8 ♀; **Oconee Co.**; Coon Branch Natural Area; 35.017, -82.997; 18 Oct. 2020; C.W. Harden leg.; • 8 ♂; **Oconee Co.**; Coon Branch Natural Area; 35.0200, -83.0000; 21 Jun. 2018; B. Owens and C. Carlton leg.; CWH-103 to CWH-110, CUAC000169292 to CUAC000169299; • 10 ♀; **Oconee Co.**; Coon Branch Natural Area; 35.0200, -83.0000; 21 Jun. 2018; B. Owens and C. Carlton leg.; CUAC000169300 to CUAC000169316; • 1 ♂, 1 ♀; **Oconee Co.**; Coon Branch Natural Area; 35.0256, -83.0050; 2 Oct. 2021; C.W. Harden leg.

####### Literature records.

**USA** • **Tennessee** • **Blount Co.**; Great Smoky Mountains National Park, upper Gregory Ridge Trail; 35.5268, -83.8530; 12 Apr. 2006; A.K. Tishechkin leg.; • **Blount Co.**; Great Smoky Mountains National Park, upper Gregory Ridge Trail; 35.5583, -83.8416; 28 Jul. 2004; A.K. Tishechkin leg.

####### Diagnosis.

The male genitalia are unique: the median lobe is strongly curved and twisted dorsally, lacking a prominent dorsal projection apically; the flagellum is short and rotated dorsally so that in right dorsolateral view it is seen through the base. The female spermatheca is S-shaped (Fig. 21P), with the basal bend shallower than that in *A.loweae* and *A.merritti*. The spermathecal duct is short and not coiled. In SC, females are smaller than those of other *loweae*-group species (Fig. 39C).

####### Distribution.

Western Smokies (Blount Co., TN) south to Graham Co, NC and east to Rabun Co, GA and Oconee Co, SC (Fig. 38).

####### Sympatry.

In SC, specimens of *A.cherokee* have been collected with *A.murrayae*, *A.* sp. “South Carolina, Coon Branch”, and *S.dunavani* at Coon Branch, Oconee Co. At Rabun Bald, GA, *A.cherokee* co-occurs with the closely-related species *A.merritti*.

####### Natural history.

Specimens examined were collected from sifted litter, sifted woody debris, underneath rocks, soil washing, and buried pipe traps. Ferro et al. (2012) found *A.cherokee* to be significantly associated with primary forest.

####### Notes.

Jeannel’s illustration of the aedeagus of *Anillinusdohrni* (Ehlers) (1963a, fig. 64) is identical to the appearance of that of *A.cherokee* in left lateral aspect. The specimen figured was collected in Clayton, GA, within the known range of *A.cherokee*. The specimen Jeannel had before him was most likely a member of *A.cherokee*. The identity of *A.dohrni* remains unknown; the female type (Academy of Natural Sciences, Philadelphia) is labeled “Florida” without further information. Images of the type sent to us by J. Weintraub show the specimen to be in good condition, with an ABL of 1.60 mm and a moderately convex habitus similar to females of several species groups of *Anillinus*.

###### ﻿﻿ Anillinus loweae

Taxon classificationAnimaliaColeopteraCarabidae

Sokolov & Carlton, 2004

50966AC5-0396-5B0C-A9AE-10B2E54B63DB

Figs 2O , 21Q , 24N , 38 , 39B

Anillinus dunavani Jeannel 1963: 76; Barr 1995: 245.
Anillinus loweae Sokolov & Carlton, 2004: 218.

####### Material examined.

***Holotype male*** (**USNM**), point mounted, not dissected, labeled “USA: NC: Haywood Co., GSMNP, Cataloochee Divide Trail near Purchase, UTM311819 E 3940339 N C. Carlton 17 July 2002” “HOLOTYPE *Anillinusloweae* sp. n. Sokolov and Carlton des 2003”

####### Other material

**(*n* = 244). USA** • **Georgia** (**new state record**) • 2 ♂, 1 ♀; **Rabun Co.**; Chattahoochee National Forest, Rabun Cliffs; 34.9707, -83.3008; 25 Nov. 2019; M.S. Caterino leg.; CWH-280 to CWH-282, CUAC000168354 to CUAC000168356; • **North Carolina** • 1 ♀; **Canton Co.**; Art Loeb Trail; 35.3957, -82.8690; 15 Jul. 2015; S. Myers leg.; MSC-2462, CUAC000185782; • 1 ♂; **Haywood Co.**; Pisgah National Forest, Mount Hardy summit; 35.3036, -82.9274; 8 Sep. 2020; C.W. Harden leg.; • 1 ♂, 1 ♀; **Haywood Co.**; Pisgah National Forest, Black Balsam Knob; 35.327, -82.874; 20 Oct. 2020; M.S. Caterino leg.; • 14 ♂, 15 ♀; **Haywood Co.**; Mount Pisgah; 35.4250, -82.7529; 10 Aug. 2021; M. Caterino and A. Haberski leg.; • 1 ♂; same data as previous; CWH-477, CUAC000066797; • 6 ♂, 9 ♀; **Haywood Co.**; Great Smoky Mountains National Park, Cataloochee Divide; 35.5859, -83.0815; 8 Jun. 2020; B. Camper leg.; • 6 ♂, 9 ♀; **Haywood Co.**; Great Smoky Mountains National Park, Cataloochee Divide; 35.5865, -83.0811; 8 Jun. 2020; B. Camper leg.; • 1 ♂; **Haywood Co.**; Great Smoky Mountains National Park, Cataloochee area, Rough Fork Trail; 35.610, -83.117; 29 Jul. 2002; C. Carlton leg.; NCSU_ENT00293718; • 1 ♂; **Haywood/Jackson Cos.**; Waterrock Knob; 35.46, -83.13; 30 May 2001; R. Davidson leg.; •2 ♂, 3 ♀; **Jackson Co.**; Whiteside Mountain, near Highlands; 35.083, -83.138; 25 May 2014; T. Lawton leg.; TLc; • 2 ♂; **Jackson Co.**; Toxaway Mountain; 35.132, -82.982; 5 Aug. 2020; • 13 ♂, 10 ♀; same data as previous; 13 Oct. 2020; • 1 ♂; **Jackson Co.**; Balsam Mountain Preserve; 35.3008, -83.0971; 20 Jul. 2016; M. Caterino and L. Vasquez leg.; CUAC000055510; • 1 ♂; **Jackson Co.**; Blue Ridge Parkway, Rough Butt Overlook; 35.3039, -82.9429; 27 Jun. 2018; K.E. Schnepp leg.; KESc; • 1 ♂; **Jackson Co.**; Blue Ridge Parkway, Cowee Mountain Overlook; 35.3556, -82.9888; 27 Jun. 2018; K.E. Schnepp leg.; KESc; • 2 ♂; **Jackson Co.**; Balsam Mountain Preserve; 35.3681, -83.1036; 17 Jun. 2015; S. Myers leg.; SSM 236 and SSM237, CUAC000185547 and CUAC185548; • 1 ♀; **Jackson Co.**; Balsam Mountain Preserve; 35.3703, -83.1216; 17 Jun. 2015; S. Myers leg.; SSM412, CUAC000185549; • 2 ♂; **Jackson Co.**; Balsam Mountain Preserve; 35.3772, -83.0921; 17 Jun. 2015; S. Myers leg.; SSM229 and SSM231, CUAC000185545 and CUAC000185546; • 1 ♀; **Jackson Co.**; Balsam Mountain Preserve; 35.3808, -83.0971; 17 Jun. 2015; S. Myers leg.; SSM413, CUAC000185550; • 2 ♂, 1 ♀; **Jackson Co.**; Balsam Mountain Preserve; 35.3869, -83.1507; 16 Jun. 2015; S. Myers leg.; CWH-135 to CWH-137, CUAC000168348 to CUAC000168350; • 3 ♂, 4 ♀; **Jackson Co.**; Balsam; 35.42, -83.08; 17 Jul. [no year]; OSUC442495 to OSUC442501; OSUC; • 3 ♂; **Jackson Co.**; Tennessee Mt [*sic*]; 18 May 1972; J. Hunter leg; NCSU_ENT00293720 to NCSU_ENT00293722; • 1 ♀; **Macon Co.**; Highlands; 35.05, -83.19; 24 Jul. 1962; R.C. and A. Graves leg.; NCSU; • 3 ♂, 3 ♀; **Macon Co.**; 1 mi northwest of Highlands; 35.061, -83.217; 24 Aug. 1981; J. Pakaluk leg.; NCSU; • 1 ♀; **Macon Co.**; 1.5 mi northwest of Highlands; 35.063, -83.174; 2 Jul. 1983; J. Pakaluk leg.; NCSU; • 6 ♂, 4 ♀; **Macon Co.**; 2.5 mi northwest of Highlands; 35.072, -83.230; 30 Jun. 1983; NCSU; • 1 ♂, 1 ♀; **Macon Co.**; Nantahala National Forest, trail to Cliffside Lake near Highway 28; 35.0745, -83.2390; 2 Jul. 2020; C.W. Harden and L.M. Thompson leg.; CWH-210 and CWH-211, CUAC000168351 and CUAC000168352; • 4 ♀; **Macon Co.**; Jones Gap; 35.0841, -83.29786; 28 Jul. 2015; S. Myers leg.; SSM163 to SSM166, CUAC000185542 to CUAC000185544 and CUAC000185903; • 1 ♂, 1 ♀; **Swain Co.**; Great Smoky Mountains National Park, Double Springs Gap; 35.5646, -83.5447; 3 Jun. 2020; S. Bewick leg.; CUAC; • 3 ♂; **Swain Co.**; Great Smoky Mountains National Park, Double Springs Gap; 35.5649, -83.5447; 3 Jun. 2020; B. Camper leg.; CUAC; • 1 ♂; **Swain Co.**; Great Smoky Mountains National Park, Newfound Gap; 35.6103, -83.4285; 29 Sep. 2020; C.W. Harden; CUAC; • 10 ♀; **Transylvania Co.**; Sassafras Mountain; 35.0656, -82.7776; 11 Jun. 2020; CUAC; • 8 ♂, 19 ♀; **Transylvania Co.**; Sassafras Mountain; 35.0657, -82.7757; 20 Oct. 2020; F. Etzler and P. Wooden leg.; CUAC; • 1 ♂; **Transylvania Co.**; Sassafras Mountain; 35.0658, -82.7763; 20 Oct. 2020; F. Etzler and P. Wooden leg.; CWH-371, CUAC000168357; • 2 ♂, 11 ♀; same data as previous; • 1 ♂, 3 ♀; **Transylvania Co.**; Balsam Grove; 35.2530, -82.9032; 11 Sep. 2019; M.S. Caterino leg.; CUAC; • 1 ♀; **Transylvania Co.**; Courthouse Falls Trail; 35.2716, -82.8964; 23 Jul. 2015; S. Myers leg.; SSM263, CUAC000170596; • 1 ♂, 1 ♀; **Transylvania Co.**; Courthouse Falls Trail; 35.2747, -82.8902; 23 Jul. 2015; S. Myers leg.; SSM264 and SSM265, CUAC000170597 and CUAC000170598; • 1 ♂; **Transylvania Co.**; Pisgah National Forest, Sycamore Flats; 35.2760, -82.7120; 15 Jun. 1965; J.F. Cornell leg.; ex slime mold; NCSU; • 1 ♂; **Transylvania Co.**; Blue Ridge Parkway; 35.2871, -82.9080; 29 May 2015; S. Myers leg.; MSC-2456, CUAC000185779; • 1 ♀; **Transylvania Co.**; Pisgah National Forest, Highway 215 1 mi south of Blue Ridge Parkway; 35.2910, -82.9133; 8 May 2018; M.S. Caterino, R. Kucuk, L. Cushman leg.; • 1 ♂; **Transylvania Co.**; Near Brevard, Pisgah National Forest, Pink Bed Area, Forest Road 1206, in deer dung; 4 Aug. 2009; J.F. and T.A.D. Cornell leg.; NCSU; • 9 ♂, 2 ♀; • same data as previous, but ex flood debris; • **Tennessee** • 1 ♂; **Cocke Co.**; Great Smoky Mountains National Park, Albright Grove; 35.7340, -83.2807; 6 Aug. 2006; J.F. Cornell and S. Ranger leg.; NCSU; • 3 ♂, 1 ♀; **Sevier Co.**; Great Smoky Mountains National Park, Newfound Gap; 35.6110, -83.4250; 17 Jul. 2003; S. O’Keefe leg.; NCSU; • 1 ♂; **Sevier Co.**; Great Smoky Mountains National Park, Newfound Gap; 35.6110, -83.4250; 16 Jul. 2003; J.S. Ashe leg.; NCSU; • 1 ♂; **Sevier Co.**; Great Smoky Mountains National Park; 35.6125, -83.5425; 6 Jun. 2020; B. Camper leg.; CUAC; • 2 ♂, 1 ♀; **Sevier Co.**; Great Smoky Mountains National Park; 35.6130, -83.5427; B. Camper leg.; 6 Jun. 2020; B. Camper leg.; CUAC; • 3 ♂; **Sevier Co.**; Great Smoky Mountains National Park, Mount LeConte; 35.6382, -83.4387; 28 Sep. 2021; M. Caterino and E. Recuero leg.; MSC-9549, CUAC000160000, CUAC000173086 and CUAC000173087; CUAC & GRSM; • 2 ♂, 2 ♀; **Sevier Co.**; Great Smoky Mountains National Park, Trillium Gap; 35.6734, -83.4338; 7 Jun. 2020; B. Camper leg.; CUAC; • 1 ♀; **Sevier Co.**; Great Smoky Mountains National Park, Trillium Gap; 35.6738, -83.4336; 7 Jun. 2020; S. Bewick Leg.; CUAC; • 1 ♀; **Sevier Co.**; Great Smoky Mountains National Park, Albright Grove; 35.7340, -83.2807; 4 Jun. 2020; B. Camper leg.; CUAC.

**Literature records. USA** • **North Carolina** • **Haywood Co.**; Blue Ridge Parkway, Woodfin Cascade; 35.4526, -83.0634; 28 May 1986; A. Smetana leg.; CNC; • **Haywood Co.**; Great Smoky Mountains National Park, Purchase Knob; 35.5828, -83.0625; 20 Jul. 2002; C.E. Carlton leg.; GRSM; • **Haywood Co.**; Great Smoky Mountains National Park, McKee Branch Trail; 35.5850, -83.0833; 14 Jul. 2002; C.E. Carlton leg.; LSAM; • **Haywood Co.**; Great Smoky Mountains National Park, Rough Fork Trail upper; 35.5893, -83.1415; 29 Jul. 2002; C.E. Carlton; LSAM; • **Haywood Co.**; Great Smoky Mountains National Park, Cataloochee Divide Trail north; 35.6113, -83.0630; 23 Jul. 2002; C.E. Carlton leg.; GRSM; • **Haywood Co.**; Great Smoky Mountains National Park, Chestnut Branch Trail; 35.760, -83.123; 1 Aug. 2001; A.K. Tishechkin leg.; LSAM; • **Jackson Co.**; Whiteside Mountain near Highlands; 35.0797, -83.1412; 21 May 1986; A. Smetana leg.; CNC; • **Jackson Co.**; Blue Ridge Parkway, Waterrock Overlook, Mile 452; 35.4607, -83.1406; 1 Nov. 1967; J.M. and B.A. Campbell leg.; CNC; • **Macon Co.**; Highway 64 near Dry Falls; 35.0673, -83.2385; 16 May 1986; A. Smetana leg.; CNC; • **Swain Co.**; Great Smoky Mountains National Park, Flat Creek Trail; 35.5502, -83.1725; 31 Jul. 2001; A.K. Tishechkin leg.; LSAM; • **Swain Co.**; Great Smoky Mountains National Park, Collins Picnic area, Quiet Walk; 35.5656, -83.3408; 20 Jul. 2002; C.E. Carlton leg.; LSAM; • **Swain Co.**; Great Smoky Mountains National Park, Heintooga overlook; 35.5727, -83,1807; 29 Jun. 1994; J.F. Cornell leg.; LSAM; • **Swain Co.**; Great Smoky Mountains National Park, Kanati Fork Trail; 35.5765, -83.3745; 20 Jul. 2002; C.E. Carlton leg.; LSAM; • **Swain Co.**; Great Smoky Mountains National Park, Quiet Walk, across from Kanati Fork Trail; 35.5859, -83.3628; 20 Jul. 2002; C.E. Carlton leg.; LSAM; • **Swain Co.**; Great Smoky Mountains National Park, Deep Creek trail upper; 35.5977, -83.4245; 22 Jul. 2002; C.E. Carlton leg.; LSAM; • **Swain Co.**; Great Smoky Mountains National Park, Kephart Prong trail; 35.6100, -83.3655; 20 Jul. 2003; A.K. Tishechkin; LSAM; • **Swain Co.**; Great Smoky Mountains National Park, Beech Gap trail; 35.6275, -83.2116; 20 Oct. 2001; C.E. Carlton and A. Cline leg.; LSAM; • **Swain Co.**; Great Smoky Mountains National Park, Smokemont Campground; 35.5573, -83.3116; 10 Jun. 1982; Y. Bousquet leg.; CNC; • **Transylvania Co.**; Pisgah National Forest, along Forest Road 215; 35.166, -82.840; 4 Mar. 1997; C.E. Carlton leg.; LSAM; • **South Carolina** • **Pickens Co.**; Sassafras Mountain; • **Tennessee** • **Cocke Co.**; Great Smoky Mountains National Park, Albright Grove Trail; 35.7361, -83.2791; 19 Oct. 2001; C.E. Carlton, A. Cline, A. Tishechkin leg.; LSAM; • **Cocke Co.**; Great Smoky Mountains National Park, Gabes Mountain Trail at Hen Wallow Falls; 35.7586, -83.2381; 19 Jul. 2002; C.E. Carlton leg.; LSAM; • **Sevier Co.**; Great Smoky Mountains National Park; Chimneys Picnic Area nature trail; 35.6350, -83.4958; 30 Jun. 2001; C.E. Carlton, A. Tishechkin, V. Moseley leg.; LSAM; • **Sevier Co.**; Great Smoky Mountains National Park, Roaring Fork area, Rainbow Falls Trail; 35.6614, -83.4608; 1 Aug. 2002; C.E. Carlton leg.; LSAM.

####### Diagnosis.

The male genitalia are unique within the *loweae* group in having a group of dark, sclerotized spines in the endophallus. The apex of the median lobe has a more prominent dorsal projection. The flagellum is slightly rotated dorsally, and is slightly sinuate, with the distal 1/2 nearly straight. Females (Fig. 39B) are similar in habitus to males, though smaller. The spermatheca is S-shaped (Fig. 21Q), with a deep basal bend, similar that of *A.merritti*. The spermathecal duct is short and not coiled.

####### Distribution.

The species has a relatively wide range that includes the Great Balsams, Plott Balsams, Eastern Smokies, and portions of the Southern Blue Ridge escarpment as far west as Rabun Bald in Georgia. Sassafras Mountain is the only known South Carolina occurrence (Fig. 38).

####### Sympatry.

In South Carolina, specimens have been collected in association with *Serranillusdunavani*. Elsewhere, the species has also been collected with *A.murrayae*, *A.langdoni*, and *A.* sp. “North Carolina, Balsam Mountain.”

####### Natural history.

Specimens examined were collected from sifted litter, sifted flood debris, deer dung, slime mold (*Stemonitis* sp.), and underneath rocks.

####### Notes.

Jeannel (1963a) and Barr (1995) illustrated this species as *Anillinusdunavani* Jeannel, but the holotype of that species was found to be a *Serranillus*. See Sokolov et al (2004) for discussion. We have not seen specimens collected in South Carolina.

###### ﻿﻿ Anillinus merritti

Taxon classificationAnimaliaColeopteraCarabidae

Sokolov & Carlton, 2010

5EA61554-F802-538E-BE51-C3D991C6505F

Figs 21R , 24P , 38 , 39A

Anillinus merritti Sokolov & Carlton, 2010: 9.

####### Material examined.

Holotype male (**USNM**), point mounted and dissected with genitalia in dried-out glycerin cup pinned beneath specimen.

####### Other material

**(*n* = 77). USA** • **Georgia** (**new state record**) • 1 ♂, 1 ♀; **Habersham Co.**; 34.5726, -83.5477; Jun. 1946; J.M. Valentine leg.; CMNH; • 2 ♂, 4 ♀; **Rabun Co.**; Chattahoochee National Forest, ca. 1 km south of Rabun bald trailhead; 34.9708, -83.3032; 2 Jul. 2020; C.W. Harden leg.; buried pipe trap; CWHc; • 1 ♀; **Rabun Co.**; Chattahoochee National Forest, ca. 1 km south of Rabun bald trailhead; 34.9709, -83.3031; 2 Jul. 2020; C.W. Harden leg.; buried pipe trap; CWHc; • 1 ♂, 4 ♀; **Rabun Co.**; Chattahoochee National Forest, ca. 1 km south of Rabun bald trailhead; 34.9711, -83.3032; 2 Jul. 2020; C.W. Harden leg.; buried pipe trap; CWHc; • 1 ♂; **Rabun Co.**; Chattahoochee National Forest, ca. 1 km south of Rabun bald trailhead; 34.9712, -83.3030; 2 Jul. 2020; C.W. Harden leg.; CWHc; • 1 ♀; **Rabun Co.**; Chattahoochee National Forest, Rabun Bald trail; 34.9724, -83.3020; 29 Sep. 2019; C.W. Harden leg.; under rock; CWH-027, CUAC000169281; • 1 ♂, 4 ♀; **Rabun Co.**; Chattahoochee National Forest, 0.6 km south of Rabun Bald trailhead, east of Sky Valley; 34.9736, -83.3085; 26 Oct. 2019; C.W. Harden leg.; under rock; CWH-041, CWH-043 to CWH-046, CUAC000168372, CUAC000169274 to CUAC000169277; • 2 ♂, 3 ♀; **Rabun Co.**; Chattahoochee National Forest, 0.6 km south of Rabun Bald trailhead, east of Sky Valley; 34.9748, -83.3059; 26 Oct. 2019; C.W. Harden leg.; under rock; CWH-040, CWH-042, CWH-047, CWH-048, CWH-095, CUAC000168371, CUAC000168373, CUAC000169278 to CUAC000169280; • **Rabun Co.**; Beegum Gap; 34.9786, -83.3032; 11 Aug. 1970; T.C. Barr leg.; CMNH; • **North Carolina** • 1 ♂; **Macon Co.**; Coweeta Hydrological Lab, ca. 13 mi west of Highlands; 35.0450, -83.4510; 22 May 1965; H.R. Steeves leg.; CMNH; • 3 ♂, 4 ♀; **Macon Co.**; Turtle Pond Creek, ca 4 mi west-northwest of Highlands; 35.06, -83.26; 8 Aug. 1970; T.C. Barr leg.; CMNH; • 1 ♂, 1 ♀; **Macon Co.**; Jones Gap; 35.0752, -83.2883; 16 Jul. 2015; S. Myers leg.; SSM245 and SSM246, CUAC000170592 and CUAC000170593; • 1 ♀; **Macon Co.**; Jones Gap; 35.0785, -83.2923; 22 Jul. 2015; S. Myers leg.; SSM174, CUAC000169286; • 1 ♀; **Macon Co.**; Nantahala National Forest, Cliffside Vista trail; 35.0795, -83.2416; 2 Jul. 2020; C.W. Harden and L.M. Thompson leg.; under rock; CWH-203, CUAC000169284; • 1 ♀; **Macon Co.**; 4 mi northwest of Highlands; 35.0840, -83.2570; 19 Mar. 1976; Q.D. Wheeler leg.; litter extraction; OSUC; • 1 ♂, 1 ♀; **Macon Co.**; 0.6 mi northeast of Goldmine, California Ridge; 35.10, -83.28; 14 May 1971; T.C. Barr leg.; CMNH; • 1 ♀; **Macon Co.**; Nantahala National Forest, off Wayah Road ca. 10 km from Route 64; 35.1554, -83.5584; 3 Aug. 2020; C.W. Harden leg.; buried pipe trap; CWHc; • 1 ♀; **Macon Co.**; Nantahala National Forest, off Wayah Road ca. 10 km from Route 64; 35.1556, -83.5583; 20 Oct. 2019; C.W. Harden leg.; under rock; CWH-055; CUAC000169291; • 3 ♂, 14 ♀; **Macon Co.**; Nantahala National Forest, off Wayah Road ca. 10 km from Route 64; 35.1557, -83.5583; 3 Aug. 2020; buried pipe trap or under rock; C.W. Harden leg.; CWHc; • 2 ♂, 9 ♀; **Macon Co.**; Nantahala National Forest, off Wayah Road ca. 10 km from Route 64; 35.1557, -83.5583; 4 Jun. 2021; C.W. Harden leg.; buried pipe trap; • 1 ♂, 1 ♀; **Macon Co.**; Nantahala National Forest, Forest Service road 1.25 mi south of Wayah Bald; 35.1700, -83.5810; 18 Apr. 2020; C.W. Harden leg.; under rock; CWH-165 and CWH-166, CUAC000169282 and CUAC000169283; • 1 ♀; **Macon Co.**; Nantahala National Forest, Bartram Trail, Wallace Branch, end of Ray Cove Road; 35.1809, -83.4336; 3 Aug. 2020; C.W. Harden leg.; under rock; CWH-208, CUAC000169285; **Swain Co.**; Twentymile Trail near Twentymile Creek; 35.4800, -83.8450; 19 Oct. 2007; I.M. Sokolov leg.; litter sifting; NCSU_ENT00293734; NCSU; • **South Carolina** (**new state record**) • 1 ♀; **Oconee Co.**; Indian Camp Creek; 34.9899, -83.0724; 4 May 2015; S. Myers leg.; litter extraction; SSM414, CUAC000169287.

####### Literature records.

**USA** • **North Carolina** • **Macon Co.**; Coweeta Hydrobiological Station, Shope Fork; 35.0597, -83.4532; 29 May 1983; D.S. Chandler leg.; litter extraction; LSAM.

####### Diagnosis.

The male genitalia are diagnostic: the median lobe is straighter than in other *loweae*-group species, and the apex is more elongate. The flagellum is slightly rotated dorsally; in right lateral view it is similar to that of *A.loweae*, but shorter; in dorsal view it is evenly curved. Female spermatheca (Fig. 21R) is similar in form to that of *A.loweae*, S-shaped with deep basal bend, and a short uncoiled spermathecal duct.

Females of this species have two distinct phenotypes: west of the Little Tennessee River, most females resemble those of *A.cherokee* (cf. Fig. 39C), whereas east of the Little Tennessee River, females have greatly narrowed humeri without angles, giving the body a striking hourglass shape (Fig. 39A). The two forms seem to intergrade in the vicinity of Wayah Bald in Macon Co., NC, and the DNA sequence data associate both forms with typical males of *A.merritti*.

####### Distribution.

The range of this species is similar to that of *A.cherokee*, but it has not yet been found in Tennessee (Fig. 38).

####### Sympatry.

At the single known SC locality, this species was collected with *A.murrayae* and *S.dunavani*.

####### Natural history.

This species inhabits deeper strata than the other described members of the *loweae* group, and should be considered endogean in habit. Evidence for this comes from seven years of extensive litter sampling within the range of *A.merritti* producing only four specimens, while a single year of endogean collecting (turning rocks during rain and using buried pitfall traps) yielded more than 50 specimens. The morphology of the sloped-humeri females is also considered to be associated with increased mobility through deeper soil strata (Sokolov 2013).

####### Note.

The single specimen known from South Carolina is a female.

##### ﻿﻿Species group incertae sedis

###### ﻿﻿ Anillinus sp. “South Carolina, Wateree”

Taxon classificationAnimaliaColeopteraCarabidae

177EBC4C-535B-513E-9560-91FD65458303

Fig. 40A–C

####### Material examined.

**USA** • **South Carolina** • 2 ♀; **Kershaw Co.**; English Swamp, Wateree Floodland Memorial Forest; 34.0911, -80.6578; 27 Feb. 2010; J.F. Cornell, S. Cornell, and B. Gregory leg.; litter ex *Pinus* stumps; NCSU.

####### Diagnosis.

**Figure 40.**
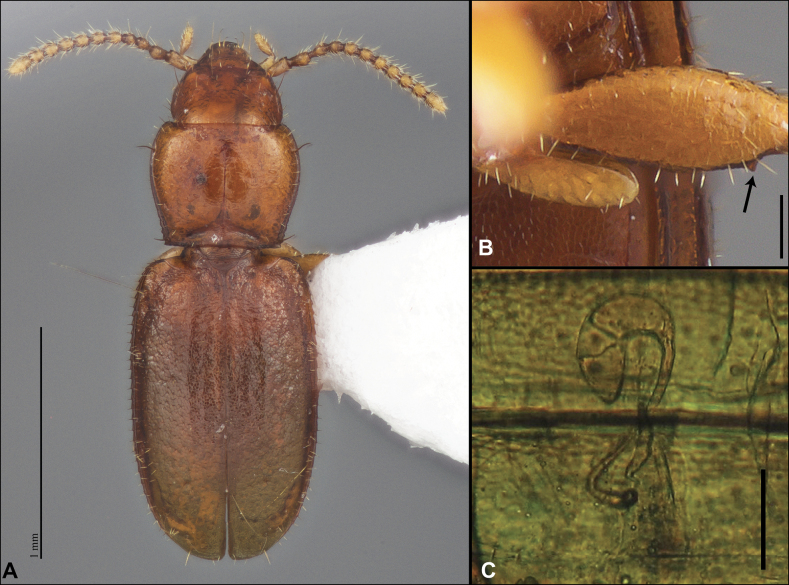
*Anillinus* sp. “South Carolina, Wateree.” **A** dorsal habitus **B** left metafemora, ventral aspect (black arrow = metafemoral spine) **C** spermatheca within cleared abdomen. Scale bars: 1 mm (**A**) and 0.1 mm (**B, C**).

The two female specimens of this species are unique among known eastern *Anillinus* females in having a sharp tooth on the posterior margin of the metafemur in the distal 1/3 (Fig. 40B). The specimens are large, ABL = 2.25 mm, dorsoventrally flattened and parallel-sided (Fig. 40A). Dorsal microsculpture is fully developed on the head and pronotum. The spermatheca is long, 2-shaped in ventral aspect (Fig. 40C), stem narrow with an acute bend basally, abruptly enlarged at curved apex. Spermathecal duct long and not coiled. The gonocoxites are longer and narrower than in most *Anillinus* species.

####### Notes.

The proper systematic placement of this species is unclear. Externally, the species is similar to members of the *elongatus* group, but all species in that group have a long and heavily coiled spermathecal duct, and modified metafemora are unknown in females. Several large samples of sifted wood and soil taken from large pine stumps at the known locality in April 2021 failed to produce specimens.

## ﻿﻿Discussion

### ﻿﻿Phylogenetics and systematics of eastern Nearctic Anillini

The Nearctic Anillini, and the Appalachian species in particular, have long been recognized as distinct from other anillines, beginning with Jeannel’s monograph (Jeannel 1963a, 1963b) in which *Anillinus* is placed in its own phyletic series based on the relatively small right paramere. Most genera of anillines have not yet been sampled for molecular phylogenetics, but several studies have consistently recovered an Anillini topology in which the New Zealand endemic genus *Nesamblyops* Jeannel is sister to all other anillines, and the Nearctic taxa are sister to all anillines sampled except for *Nesamblyops* (Maddison and Ober 2011; Andújar et al. 2016; Maddison et al. 2019; LaBonte and Maddison 2023), as in our 6-gene phylogeny (Suppl. material 1: fig. S10). Sokolov (2023) formally erected the subtribe Nesamblyopina for the species of *Nesamblyops*, and pointed out that the Nearctic anillines share with the species of *Nesamblyops* an asetose posterior pronotal margin, an apparently plesiomorphic state within Trechitae. In other anillines that have been studied, the posterior pronotal margin is setose.

The newly discovered larvae of *Anillinus* and *Serranillus* add further support for the exclusion of Nearctic Anillini from the large clade of other Anillina, Scotodipnina, and Typhlocharina (Andújar et al. 2016; Maddison et al. 2019; Sokolov 2023). Larvae of three anilline genera have been previously described, *Zapotecanillus* Sokolov from Mexico (as *Geocharidius* Jeannel) (Grebennikov 2002), *Typhlocharis* Dieck, and *Microcharidius* Coiffait from Spain (Arndt et al. 1999; Andújar et al. 2010; Pérez-González et al. 2018). Grebennikov (2002) listed ten possible synapomorphies of the two known anilline larvae, five of which were found to strongly support monophyly of Anillini (Grebennikov and Maddison 2005): presence of only two pores on antennomere 1, reduction or absence of antennomere 2, antennal fossa separated from pleurostoma by a strip of cuticle, mandible with two large terebral teeth, and mandible with greatly reduced retinaculum. None of these character states are found in larvae of *Anillinus* and *Serranillus*; in both genera, antennomere 1 has three pores, antennomere 2 is typical in length for Trechitae, the antennal fossa is not separated from the pleurostoma, the mandible lacks large terebral teeth, and the retinaculum is well-developed (Fig. 14).

Another character, the ocular tubercles in adults of *Serranillus*, has not been previously noted, although it has bearing on the matter. The position of the tubercles on the head in *Serranillus* is the same as the ommatidia in *Nesamblyops* (cf. Sokolov 2023: fig. 1a–c); the tubercles could represent 'scars' left from the fusion of integument at the site of former ommatidia. Similar ocular tubercles are found in other eyeless beetles, including several clivinine carabid species (Barr 1967; Bousquet and Skelley 2012; Huang et al. 2021) and most pselaphine rove beetles of the tribe Amauropini (Carlton 2008; Hlaváč et al. 2021). We have noted ocular tubercles in a small number of *Anillinus* species, but none are as prominent as in *Serranillus* except in *A.indianae* and in an undescribed species from Washington Co., Arkansas. The character requires more study, but it could represent another plesiomorphy of Nearctic Anillini.

Likely morphological apomorphies of *Serranillus* are the greatly reduced right paramere that lacks pores or setae, the large internal rolled sclerite on the left side of the median lobe, and the modified last male abdominal ventrite. The presence of a retinacular tooth on the left mandible is rare within Anillini, and is another possible apomorphy, but mandibular teeth are not described for most anillines, and not all *Serranillus* species have been checked. There are at least ten additional known species of *Serranillus*, some of which are quite divergent in male genitalic morphology, which were not sampled by us. Including these species in future molecular phylogenetic studies will help clarify the higher classification of the genus.

**Figure 41.**
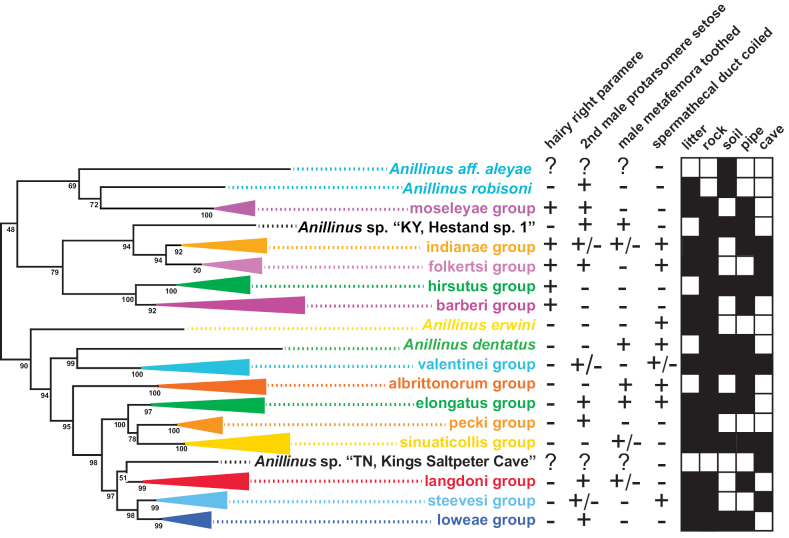
6-gene maximum likelihood tree of *Anillinus*, with select character states and microhabitats shown. Numbers below nodes are SBS values. ‘+’ denotes presence of character and ‘-’ denotes absence; these do not correspond to presumed derived and plesiomorphic states in all characters. Black squares indicate records of collections from litter (hand sifting, Berlese or Winkler extraction), rock (hand collecting under rocks), soil (Berlese extraction of washed or unwashed soil), pipe (buried pipe trap), and cave (hand collected in cave).

While morphological support for monophyly of *Anillinus* is lacking, each of the species groups identified by us is morphologically diagnosable. Furthermore, we have identified characters that show promise to be phylogenetically informative, given their consistency within clades in the 6-gene phylogeny (Fig. 41). Among male genitalic characters that vary across species of *Anillinus*, for example, is setation of the right paramere. Some species have numerous setae forming a dense brush on the apex of the paramere, whereas in other species only a few stout setae are present, typically four in number (Sokolov 2011, 2012, 2020). The presence of more than four setae on the right paramere is a unique state within Anillini; in all other anilline genera, the right paramere has four setae or fewer, with most species having only two (e.g., Jeannel 1963a; Giachino and Vailati 2011; Sokolov 2013, 2023; Giachino 2015; Pérez-González and Zaballos 2019; Giachino et al. 2021). Thus, a right paramere with more than four apical setae is likely a derived condition within the tribe. However, two of the sampled species recovered in the “hairy clade” have only four setae on the right paramere: *Anillinusrobisoni* has a stout, semicircular right paramere with four apical setae, and *Anillinus* sp. “Kentucky, Hestand sp. 1” has a small, narrow right paramere with numerous apical pores but only four apical setae. The additional pores present on the right paramere of “Kentucky, Hestand sp. 1” suggest that the reduced number of setae is a secondary loss in that species. We also note that within the “quadrisetose clade” one of the two males of *A.jancae* (the holotype) has six apical setae on the right paramere, while the other has four. Most known *Anillinus* species with more than four setae on their right parameres were not sampled for our phylogeny, so further studies will be necessary to test the reality of the “hairy clade” and the number of times a hairy right paramere has evolved.

One valuable character that has been overlooked in previous work is the number of male protarsomeres that are dilated and bear ventral adhesive setae; all *Serranillus* have the first and second protarsomeres modified, while the number varies within *Anillinus*. The typical number of dilated protarsomeres in Trechitae is two, although examples of reduction are found throughout the supertribe. The number of modified male protarsomeres has been considered a phylogenetically useful character in other trechite genera, such as *Paratachys* (Lindroth 1966), *Trechus* (Barr 1979), and the closely similar pair of anilline genera, *Binaghites* Jeannel and *Scotodipnus* Schaum (Magrini 2008). If two modified male protarsomeres is considered the plesiomorphic state, given its occurrence in most trechites, then reduction to one has likely evolved multiple times within *Anillinus*. Of the sixteen distinct lineages of *Anillinus* in eastern North America, the number of modified male protarsomeres varies in only three, the *indianae*, *steevesi*, and *valentinei* groups, in which only a single species is an exception (Fig. 41). The number of modified male protarsomeres has proven to be one of the most useful diagnostic characters for classifying newly discovered *Anillinus* species. For example, the species *Anillinus* sp. “North Carolina, Orange Co. sp. 2” shares some characters with *A.jancae*, including an almost identical profemoral spine (Fig. 27B), a unique character within the genus. However, unlike *A.jancae*, the single known male of “North Carolina, Orange Co. sp. 2” has a modified second protarsomere, and our initial prediction that it was related to the *elongatus* group was supported by its placement in the molecular phylogeny.

A more common male secondary sexual leg modification is the presence of a spine or tooth on the posterior face of the metafemur. Such toothed metafemora are known in seven of the eastern *Anillinus* lineages (Fig. 41). In all instances, species with toothed metafemora in males are primarily endogean in habit. The most likely function of these toothed male femora is to allow the male to securely grip the female during courtship, mating, and/or post-mating. We have observed mating pairs of *Anillinus* on two occasions on the undersides of embedded rocks, and the beetles were coupled in typical carabid fashion, with the male on top of the female, moving as a single unit when disturbed by exposure. If such in-copula pairs move throughout the interstices of deep soil habitats, males with a stronger grip would be less likely to be dislodged. Not all endogean *Anillinus* species have modified male legs, however. The *folkertsi* group and *valentinei* group are notable in being largely endogean in habit and having no species with modified male metafemora. Other endogean groups have male metafemora that are not toothed but are greatly swollen (some *barberi*-group species) or densely setose (most *hirsutus*-group species). Modified female metafemora are known only in *Anillinusalleni*, in which the males have enormous metafemoral spines and females have the posterior margin angularly produced apically (Sokolov et al. 2017) and in *Anillinus* sp. “South Carolina, Wateree”, in which females have a small, sharp tooth on the posterior face near the apex; males of “South Carolina, Wateree” are unknown.

A female character useful for diagnosing groups of *Anillinus* is the presence or absence of many coils in the spermathecal duct. Although the coiled shape is doubtfully homologous across all species, it is consistent within all species groups studied except the *valentinei* group (Fig. 41). Most species studied in this group have a long, coiled spermathecal duct, but *A.murrayae* and *A.castaneus* seem to lack a duct entirely, and *A.simplex* has a short, simple duct. The length of the spermathecal duct has been found to correspond to the length of the flagellum of the aedeagus in some groups of carabids (Schuler 1971; Liebherr 2008). We have not attempted to measure these structures in *Anillinus*, but note that the apparent lack of a spermathecal duct in *A.murrayae*, a species in which the flagellum is quite long, is a clear exception to such a pattern.

Patterns of dorsal microsculpture on the head and pronotum are variable within *Anillinus* and have been previously used to group species (Sokolov et al. 2004; Sokolov 2012). While consistent within most lineages, intraspecific variation has been documented (Sokolov and Carlton 2010; Harden and Caterino 2024), including for *A.simplex* described in this paper. In each species group, we have observed exceptions to the typical microsculpture pattern. Dorsal microsculpture, while frequently valuable for species recognition and diagnosis of species groups, is too variable to be a reliable indicator of relationships.

Patterns of observed microhabitat use have been used to classify species of *Anillinus* (Sokolov et al. 2004). Microhabitat use is consistent in many species and in some species groups, such as the largely endogean *elongatus* group and largely litter-dwelling *langdoni* group. However, the distinction between use of litter and soil habitats is not always a clear, and presumed microhabitat associations are not always consistent with collecting data. For example, the *moseleyae* group has been considered endogean, despite all of the previously reported specimens being collected from leaf litter (Sokolov et al. 2004; Sokolov 2011). In Fig. 41, we demonstrate the consistency (and lack thereof) between collecting method and phylogenetic placement of members of the species groups in our 6-gene tree. One pattern that is repeated throughout the tree is a sister relationship between a phylogenetically and geographically isolated endogean lineage and a more diverse and widespread lineage: the micro-range endemics *A.* sp. “Kentucky, Hestand sp. 1” and *A.dentatus* are sister to the *indianae* group+*folkertsi* group clade and the *valentinei* group, respectively; the *albrittonorum* group is sister to the ‘ESP+LSL’ clade, and the Piedmont endemic *elongatus* group is sister to the montane *pecki* group and widespread *sinuaticollis* group.

### ﻿﻿Biogeography of eastern Nearctic Anillini and the unique South Carolina assemblage

The anilline fauna of South Carolina was previously considered to be among the least diverse of the states from which anillines have been reported. Tallies of the state’s fauna in Bousquet (2012) and Sokolov (2021) overlooked the South Carolina record of *A.cherokee* in Sokolov and Carlton (2010), and listed only three *Anillinus* and one *Serranillus* species from South Carolina. Our work has increased the number of described anilline species in South Carolina nearly five-fold, with three described *Serranillus* and 17 described *Anillinus* now known, making South Carolina one of the most diverse states along with North Carolina and Tennessee, which previously had the highest number (19) of *Anillinus* species known among eastern states. We report nine endemic species from South Carolina (53%) as well as two unique lineages not found elsewhere in the Appalachian region, the *dentatus* group and the *albrittonorum* group. The diverse assemblage of anillines present in South Carolina doubtlessly reflects the unique combination of ecoregions and dispersal barriers present in the state.

The *Serranillus* species found in South Carolina belong to all three of the main clades in our 6-gene phylogeny. *Serranillusdunavani* is the most commonly collected anilline in South Carolina, and also the most widespread (Fig. 16). The range of the species is concentrated along the Blue Ridge escarpment, a region of high relief where many narrow streams cascade through rich cove forests, and drier oak-pine forests dominate the exposed south-facing slopes; *S.dunavani* has been collected in large numbers from both habitat extremes. Its unusually large geographic range is likely a reflection of this broad ecological tolerance. This tolerance likely also explains the occurrence of *S.dunavani* on both sides of the French Broad River basin (FBR). The FBR is an important biogeographic barrier for many groups of flightless animals, as distinctly different montane faunas and/or genotypes exist of opposite sides (Barr 1979; Donabauer 2009; Hedin and Thomas 2010; Keith and Hedin 2012; Garrick et al. 2018; Hennen et al. 2022; Caterino and Recuero 2023). The range of *S.dunavani* skirts south of the headwaters of the FBR and extends on the northeast side to the Hickory Nut Gorge in western North Carolina. Three disjunct occurrences of *S.dunavani* are known in the outer Piedmont of South Carolina and the Uwharrie Mountains of North Carolina. Little dedicated collecting has been done in the intervening areas, so this disjunction could be an artifact, but it is another illustration of both the surprising dispersal capabilities of *S.dunavani* and its wide habitat tolerances.

Two species of *Serranillus*, *S.jeanneli* and *S.* sp. “South Carolina, Coon Branch” occur in South Carolina only in the extreme northwest corner, where both are known from the mesic north-facing slopes in the lower Whitewater River gorge. Given that *S.* sp. “South Carolina, Coon Branch” has been collected at only a single locality, its full range is unknown, but *S.jeanneli* is limited to higher elevations in a small area in the North Carolina-South Carolina-Georgia corner. Other flightless carabids endemic to the Southern Appalachians that are rare or absent elsewhere in South Carolina are found in the Whitewater River Gorge, including Scaphinotus (Maronetus) unistriatus Darlington, Scaphinotus (Steniridia) violaceus (LeConte), Trechus (Microtrechus) barberi (Jeannel), and Pterostichus (Monoferonia) carolinus Darlington (CUAC data).

The last *Serranillus* occurring in South Carolina, *S.monadnock*, is known from two disjunct monadnocks, Kings Mountain in York Co. and Little Mountain in Newberry Co. The median lobe of the aedeagus, with a relatively broad apex, distinct flagellum, and lack of large spines, is similar in form to that of *S.septentrionis* and its undescribed sister species, as well as several undescribed species known from Georgia and Alabama. Without DNA sequence data the affinities of *S.monadnock* are uncertain, but it could be part of the clade that includes *S.septentrionis*, *S.* sp. “South Carolina, Coon Branch” and the other undescribed species with similar genitalia. Both collections of *S.monadnock* were made at colder times of year, and the apparent restriction of the species to isolated monadnocks suggests it is a cold-adapted lineage that has been extirpated elsewhere in South Carolina.

**Figure 42.**
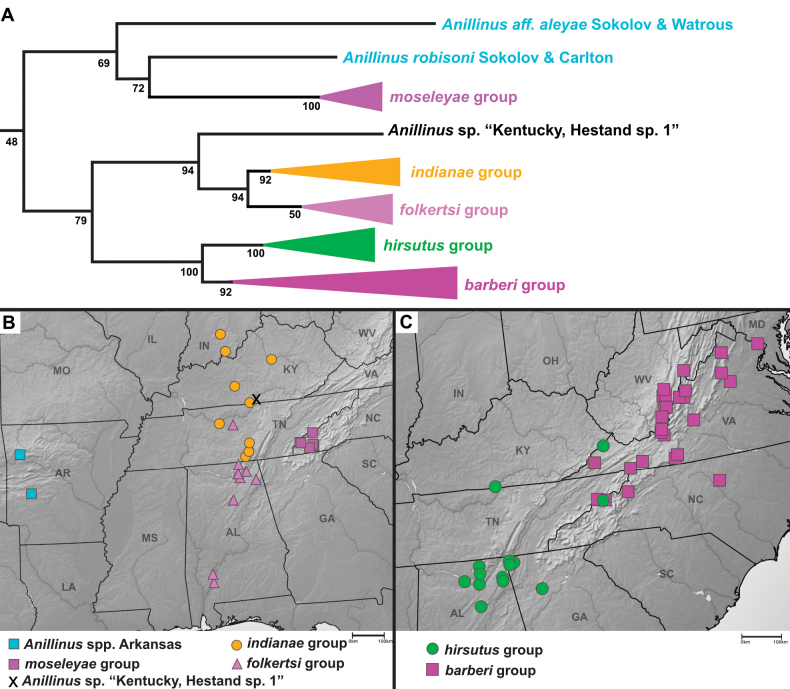
Topology and distribution of “hairy clade” species groups of *Anillinus***A** collapsed maximum likelihood tree of “hairy clade” of *Anillinus*, from 6-gene concatenated core matrix, SBS values shown below nodes **B** distribution map of Arkansas *Anillinus* specimens sampled, *Anillinus* sp. “Kentucky, Hestand sp. 1”, and all occurrences of the *moseleyae* group, *indianae* group and *folkertsi* groups **C** distribution map of all occurrences of the *hirsutus* group and *barberi* group. Data for distribution maps come from Harden (2024).

In *Anillinus*, the absence of any “hairy clade” species in South Carolina is notable, because representatives of the clade are present in every other Appalachian state in which anillines occur (Fig. 42). In terms of airline distance, the only “hairy clade” group found close enough to South Carolina to be expected to occur is the *moseleyae* group; the southernmost known occurrence of the group is at Coweeta Hydrological Lab (CMNH data), less than 30 km airline distance from the northwestern corner of South Carolina (Fig. 42B). The *moseleyae*-group species are all strictly high-elevation endemics, with no known occurrences below 1370 m. Such elevations do not occur in South Carolina, and it is unlikely that species of the group are present in the state. The factors that limit *moseleyae*-group species to higher elevations are unknown, but they seem to include more than simple microclimatic requirements, since no specimens have been found at lower elevations in endogean habitats in mesic North-facing slopes adjacent to mountains on which they occur. Two *moseleyae*-group species have surprisingly large geographic ranges, considering the apparent elevation restriction: *A.unicoi* occurs on both sides of the Little Tennessee River in the Unicoi and Great Smoky Mountains, where it has been collected on Thunderhead Mountain approximately 0.8 km west of the type locality of *A.carltoni* (NCSU data); an undescribed species has been collected in the Snowbird and Nantahala Mountains.

Other “hairy clade” groups are also unlikely to be found in South Carolina. The *indianae* group+*folkertsi* group clade is found only West of the Appalachian Mountains. Members of the *folkertsi* group are the least strictly endogean of eastern “hairy clade” species, having been collected several times in series from sifted litter, so ecological factors are unlikely to limit their occurrence in South Carolina. Rather, the distribution of the group suggests that the Alabama River drainage is a barrier to eastward dispersal (Fig. 42B). The *indianae* group is known only west of the Tennessee River, and these species seem to be ecologically restricted to cooler subterranean habitats; in Kentucky and Indiana, specimens have been collected from deep soil and MSS, while at lower and hotter elevations in southern Tennessee the group is known only from caves. A similar pattern is shown in the *hirsutus* group, which ranges from the Cumberland Mountains on the Virginia—Kentucky border and the Interior Plateau of southern Kentucky south to northern Alabama and Georgia. A notably disjunct site for the group is Big Bald, in the Bald Mountains, a short chain northeast of the FBR on the North Carolina-Tennessee border. In northern hardwood forests on the northwestern-facing slope below the treeless summit of Big Bald, at approximately 1660 m elevation, two *hirsutus*-group species co-occur under rather small rocks; one individual was even collected in a sample of sifted litter. This is in contrast to all other known localities for the group, where specimens are rarely encountered without laborious deep soil extraction methods or – as with several species in Alabama and Georgia, including *A.hirsutus* itself – known only from caves. The ecological requirements of the *hirsutus* group have apparently prevented their dispersal into South Carolina. The absence of the group from suitable habitats in the mountains southwest of the FBR (Fig. 42C) suggests that ancestors of the group never occurred in these mountains, and dispersed to Alabama and Georgia from more northern areas. The sister group to the *hirsutus* group is the *barberi* group, which ranges from Big Bald (where an undescribed species co-occurs syntopically with the two *hirsutus*-group species) north to Plummers Island in the Potomac River west of Washington D.C (Fig. 42C). Most records of the *barberi* group are from higher elevations in the Ridge and Valley and Blue Ridge ecoregions, but an undescribed species has been collected in Duke Forest near Chapel Hill, North Carolina, in an endogean Piedmont habitat similar to those that can be found in South Carolina. An undescribed *elongatus*-group species that is sister to *A.montrex* is found at the same Duke Forest site, so a biogeographic connection between the Duke Forest and South Carolina is known to exist. Further trapping in deep soils in the Piedmont of northeastern South Carolina could lead to discovery of the *barberi* group in the state, but this 200-km range extension for a predominately northern/high elevation clade would be surprising. One last biogeographical pattern we have noted in the “hairy clade” is that with the exception of *A.robisoni*, all *Anillinus* species known from West of the Mississippi river have right parameres with more than four setae (Sokolov and Watrous 2008; Sokolov et al. 2014, 2017; Sokolov 2022). We found the right paramere to have more than four setae in the western species *Anillinuslescheni* Sokolov & Carlton, *Anillinusmagazinensis* Sokolov & Carlton, *Anillinusstephani* Sokolov & Carlton, and *Anillinustishechkini* Sokolov & Carlton, which were all described without mention of the parameres (Sokolov et al. 2004). Inclusion of more western *Anillinus* species in future molecular phylogenetic studies will be key to uncovering the broader biogeographic history of the genus.

**Figure 43.**
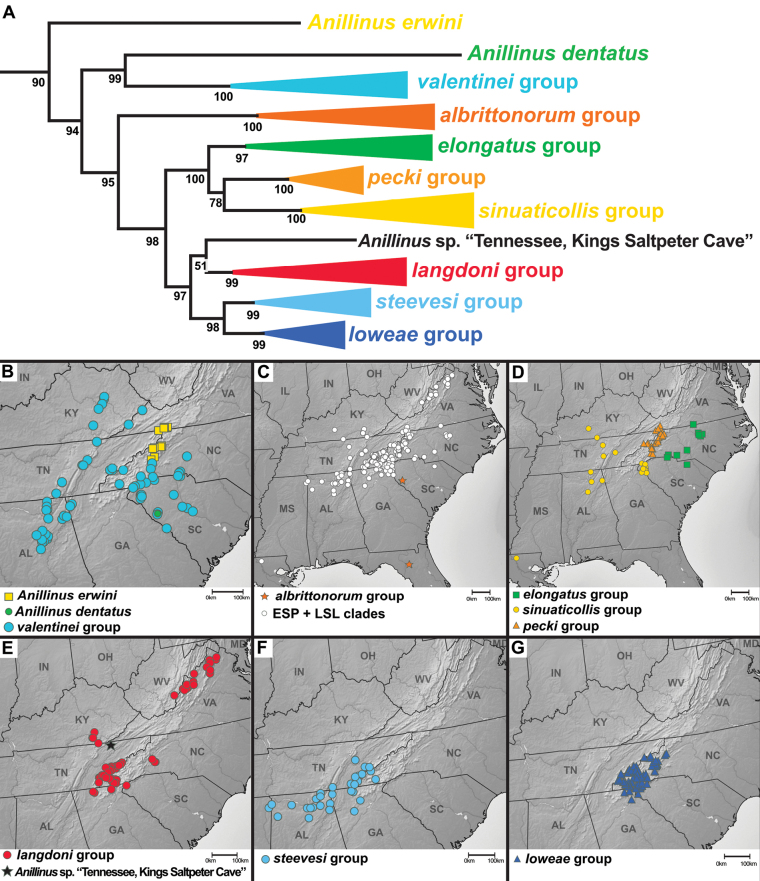
Topology and distribution maps of “quadrisetose clade” species groups of *Anillinus***A** collapsed maximum likelihood tree of “quadrisetose clade” *Anillinus* species, from 6-gene concatenated core matrix, SBS values shown below nodes **B** distribution map of all known occurrences of *Anillinuserwini* and the *valentinei* group **C** distribution map of all known occurrences of the “ESP+LSL” clade and *albrittonorum* group **D** distribution map of all known occurrences of the “ESP clade” **E** distribution map of all known occurrences of the *langdoni* group and *Anillinus* sp. “Tennessee, Kings Saltpeter Cave” **F** distribution map of all known occurrences of the *steevesi* group **G** distribution map of all known occurrences of the *loweae* group. Data for distribution maps come from Harden (2024).

All but three lineages of the “quadrisetose clade” of *Anillinus* are present in South Carolina (Fig. 43). The exceptions are the isolated species *A.erwini*, the *pecki* group, and the *steevesi* group. *Anillinuserwini* is endemic to higher elevations in the mountains northeast of the FBR; the FBR apparently has prevented this species from dispersing to the southern mountains (Fig. 43B). As discussed above, the phylogenetic placement of *A.erwini* is enigmatic, which limits hypothesizing about its biogeographic history. The *pecki* group is also endemic to montane habitats northeast of the French Broad River, with the southernmost limit being the Hickory Nut Gorge in western North Carolina. The known occurrences of the *pecki* group in the Hickory Nut Gorge are all from the south side of the gorge, where steep Northeast-facing slopes create a cool, mesic habitat despite the relatively low elevation. Ecological factors limiting the *pecki* group to colder microhabitats probably have prevented the southward dispersal of the group into South Carolina. The likely sister lineage of the *pecki* group, the *sinuaticollis* group, occurs in northwestern South Carolina, and the common ancestor of the two groups might have historically had a distribution similar to that of *S.dunavani*, with subsequent extinction during warm, dry periods along most of the southern Blue Ridge escarpment. The third quadrisetose *Anillinus* lineage lacking in South Carolina, the *steevesi* group is distributed mostly west of the southern Appalachians, with no known occurrences east of the Little Tennessee River (Fig. 43F). Members of the *steevesi* group are found mostly at lower elevations, and the absence of the group of South Carolina is probably due to physical barriers such as the Little Tennessee River or the high mountains that flank the northwestern corner of the state.

The dominant clade of *Anillinus* found at lower elevations in South Carolina is the *valentinei* group (Fig. 43B). This group is the most species-rich lineage in the genus, and also the most widespread, ranging from the Cumberland Mountains in eastern Kentucky south to northern Alabama and across the Blue Ridge escarpment to western North Carolina. Except for *A.murrayae*, members of the *valentinei* group are largely restricted to lower elevations, and are absent from most of the southern Appalachian Mountains in the strict sense. Three clades are recovered in our 6-gene phylogeny, and all are represented in South Carolina: a clade consisting of *A.chandleri* and two closely related undescribed species, a clade consisting of all sampled individuals of *A.murrayae*, and a widespread clade containing all remaining *valentinei*-group species sampled. The *chandleri* subclade is strongly supported as sister to the remaining *valentinei* group, and is endemic to South Carolina. *Anillinuschandleri* is known from scattered localities in the Piedmont between the Savannah and Broad Rivers, while the two undescribed species are known from Long Cane Creek in Abbeville Co. and a few localities in the Piedmont and Blue Ridge ecoregions in Pickens County, respectively (Fig. 29). Other than the single specimen of *A.* sp. “South Carolina, Waldrop Stone” collected at Chimneytop Gap in the Blue Ridge, all members of the *chandleri* subclade are restricted to lower elevations.

*Anillinusmurrayae* is the most widespread member of the *valentinei* group, with a range similar to that of *S.dunavani*, with which it frequently co-occurs (Fig. 29). *Anillinusmurrayae* seems to be less tolerant of hot, dry microhabitats than *S.dunavani*, and occurrences are limited to mesic habitats, usually at colder seasons. Like *S.dunavani*, *A.murrayae* spans the FBR along the Blue Ridge escarpment, and reaches a slightly more northern limit at the southern end of the Black Mountains in North Carolina. The large amount of intraspecific COI variation in *A.murrayae* (uncorrected p-distance 0.00–5.47% in COIbc, 0.00–4.61% in COIjp) suggests that more than one cryptic species may be involved. Morphological variation is also apparent (Fig. 31), but not always consistent with the molecular data. All 28S sequences sampled for *A.murrayae* are identical, and the uncorrected p-distances in CAD and Wg are less than 1.50%.

The remaining *valentinei*-group species sampled, from Virginia, Kentucky, Tennessee, Alabama, and South Carolina form the third clade in our 6-gene phylogeny. Four species in this clade occur in South Carolina, and themselves form a well-supported clade: *A.castaneus*, *A.cornelli*, *A.simplex*, and *A.* sp. “South Carolina, Chestnut Ridge”. *Anillinuscornelli* and *A.simplex* are endemic to Kings Mountain, an isolated monadnock that spans the South Carolina-North Carolina border, and *A.castaneus* and *A.* sp. “South Carolina, Chestnut Ridge” are endemic to a small area in the gorge of the upper South Pacolet River, separated from Kings Mountain by an airline distance of ~ 80 km. These Kings Mountain and South Pacolet species are widely disjunct from the remaining species in the clade, which are all known from the opposite side of the Appalachian Mountains. Dismissing a possible relationship between *A.chandleri* and the troglobitic *A.valentinei*Sokolov (2011) suggested, “As a rule *Anillinus* spp. from the same lineage are allopatric and occupy the same types of habitats”, the habitats in this case being leaf litter, soil, and caves. Our results disagree with this hypothesis. This is best exemplified in the widespread subclade of the *valentinei* group. Specimens in this clade were sampled from leaf litter, deep soil, and caves, and three cases of syntopy were documented: *A.simplex* and *A.cornelli* at Crowders Mountain, Gaston Co., NC; *A.castaneus* and *A.* sp. “South Carolina, Chestnut Ridge” at Chestnut Ridge Heritage Preserve, Greenville Co., SC; and *A.gimmeli* and *A.smokiensis* at Turkeypen Ridge, Blount Co, TN. Syntopy of two or more species belonging to the same lineage was also documented in other species groups: *A.merritti* and *A.* sp. “North Carolina, Wayah sp. 2” (Wayah Road, NC), *A.merritti* and *A.cherokee* (Rabun Bald, GA), *A.mica* and *A.micamicus* (Waldrop Stone Falls, SC), *A.castaneus* and *A.* sp. “South Carolina, Chestnut Ridge” (Chestnut Ridge Heritage Preserve, SC), A.cf.nantahala and *A.* sp. “Georgia, Brasstown Bald sp. 1” (Little Bald, GA), and *A.* sp. “Tennessee, Hiawassee sp. 1” and *A.* sp. “Tennessee, Hiawassee sp. 2” (John Muir Trail, TN).

The lack of consistency between microhabitat and the phylogeny is not surprising, considering the ecological similarities shared by deep litter, soil, and caves. Use of “cave” microhabitats is also difficult to distinguish from accidental occurrence of endogean or surface species. The only eastern *Anillinus* that has been repeatedly collected from any cave in large series is the species that lives near Clay, Alabama in the cave known as Crystal Caverns or McCluney Cave. The species was illustrated and interpreted as *Anillinusvalentinei* (Jeannel) by Sokolov (2012). In addition to *A.valentinei* (sensu Sokolov (2012)), three other *Anillinus* were considered troglobitic by Sokolov et al. (2014): *A.tombarri*, *A.longiceps*, and *A.smokiensis*. The type series of *A.smokiensis* was collected from leaf litter in a cave entrance, which is not a troglobitic habitat. We collected specimens of *A.smokiensis* from beneath rocks at two localities in deciduous forest in the Cades Cove area of Great Smoky Mountains National Park, and the species should not be considered troglobitic.

Two of the most biogeographically important anillines in South Carolina are *A.dentatus* and *A.jancae*. Both are phylogenetically isolated, possess a unique combination of male secondary sexual modifications, and are known only from the vicinity of Long Cane Creek in Abbeville Co. *Anillinusdentatus* is recovered as sister to the widespread *valentinei* group in our 6-gene phylogeny, but there is no obvious morphological support for this relationship. Nor are there other known *Anillinus* species that are likely relatives. Thus, *A.dentatus* represents a unique, relict lineage that has apparently been extirpated elsewhere (Fig. 43B). Males possess dentate mesotrochanters, a unique character in *Anillinus* but shared with males of the Oregon endemic *Medusapygaalsea* LaBonte (LaBonte and Maddison 2023). Most specimens of *A.dentatus* have been collected in cooler seasons in the months of January and March, but that is also when most collecting has taken place. Members of the species are endogean in habit, often being found beneath deeply embedded rocks in pure, red clay. The beetles move slowly when exposed, and their small distribution perhaps reflects both a strictly endogean lifestyle and greatly limited dispersal capabilities. The other notable Long Cane Creek species, *A.jancae*, is strongly supported as sister to the Florida endemic *A.albrittonorum* (Fig. 43C). Females of both species possess a unique form of spermatheca not found elsewhere in the genus, and males lack adhesive setae on the second protarsomere. Aside from these characters, there are no obvious morphological synapomorphies, but every gene sampled recovers the two species as a clade with strong support.

In the 6-gene phylogeny, the sister to the *albrittonorum* group is the large “ESP+LSL” clade, containing the bulk of quadrisetose Appalachian *Anillinus* species. A history of ancient hydrochory – transport from higher elevations by intense floods – is a tempting explanation for this pattern, but if that were the case one would expect *A.jancae* and *A.albrittonorum* to be polyphyletic within the “ESP+LSL” clade rather than each other’s closest relative. The *albrittonorum* group likely represents an even older relict lineage that was formerly more widespread across the southeastern United States. As in *A.dentatus*, the endogean habits and limited dispersal capabilities of *A.jancae* explain its very small range. In a case of remarkable convergence, male *A.jancae* possess an abdominal keel and dentate profemora, as in the Washington endemic *Medusapygachehalis* LaBonte (LaBonte and Maddison 2023). The selective pressures that have resulted in convergence between two distantly related but co-occurring *Anillinus* in the Piedmont of South Carolina and a pair of anillines in the Pacific Northwest are difficult to fathom, but it is noteworthy that all four of the species inhabit mineral soil layers and have greatly limited dispersal capabilities.

The “ESP clade”, consisting of the *elongatus*, *sinuaticollis*, and *pecki* groups, is represented in South Carolina by members of the *elongatus* and *sinuaticollis* groups. South Carolina is the only state known to have representatives of both groups, and both groups reach their southwestern and eastern-most limits, respectively, within South Carolina. The *elongatus* group contains endogean species endemic to a small area of the Piedmont ecoregion, ranging from southern Virginia to northeastern South Carolina (Fig. 43D). Most of the species are known from a single locality, and their endogean habits and slow, sluggish behavior when exposed are similar to those of *A.dentatus*; their limited ranges probably reflect poor dispersal capabilities. Sampling within the range of the group has not been dense enough to precisely identify the biogeographic barriers separating most species, but the closely related and geographically proximate *Anillinuselongatus* Jeannel and *Anillinuspittsylvanicus* occur on opposite sides of the divide between two watersheds, and the ranges of the other species suggest that passive movement by hydrochory has been an important mode of dispersal in the group (Harden and Caterino 2024), as has been suggested for other anillines (Ortuño and Gilgado 2011; Andújar et al. 2017).

The two South Carolina *elongatus*-group species are known from quite different habitats. *Anillinusmontrex* is endemic to Kings Mountain, where it has been found under rocks in a small, mesic stream hollow, whereas *A.arenicollis* lives in deep sand in longleaf pine (*Pinuspalustris* Mill.) savannah. The two species also belong to two different subclades within the *elongatus* group, with *A.montrex* sister to an undescribed species from Orange Co., North Carolina and *A.arenicollis* in a clade with the remaining species. Both clades co-occur at the Orange Co. site, where *A.elongatus* is relatively common and the undescribed species has been collected only once, from a pipe trap set in deep rocky soil. The non-sister relationship between the two Orange Co. species indicates that allopatric speciation with subsequent dispersal has occurred, rather than sympatric speciation.

*Anillinusmontrex* and its undescribed Orange Co. sister species show more pronounced morphological adaptations to endogean existence than other *elongatus*-group species, with a flatter and more parallel sided body. These two species also have larger metafemoral spines, with the Orange Co. species having two spines, a unique state in the genus. As seen also in *A.dentatus* and *A.albrittonorum*, accumulation of male secondary sexual modifications is a common pattern in endogean *Anillinus* in the southeastern United States. Endogean habit and male secondary leg modifications are found also in the *sinuaticollis* group, which is known in South Carolina only from the upper Savannah River drainage (Fig. 36). Three closely related species occur along the tributaries and historic course of the Seneca River, with *A.mica* and *A.micamicus* found north of the confluence of the Keowee and Twelvemile Rivers, and *A.seneca* found west and south of this point. *Anillinusmica* and *A.micamicus* are syntopic at all three sites from which *A.micamicus* is known. The sister to *A.micamicus* is *A.* sp. “South Carolina, Coon Branch” which is morphologically similar to *A.micamicus* and might be one end of a grade of variation. *Anillinus* sp. “South Carolina, Coon Branch” occurs in the Whitewater River Gorge, at a higher elevation than any other *sinuaticollis* group occurrences. As in the two clades of the *elongatus* group, the syntopy of *A.mica* and *A.micamicus* is the result of dispersal; the two species may have evolved allopatrically in isolated stream gorges in the Blue Ridge Escarpment and subsequently been brought together by hydrochory.

The fourth *sinuaticollis*-group species known from South Carolina, *A.choestoea*, is phylogenetically distant from the clade of *A.mica*, *A.micamicus*, and *A.seneca*, and in the 6-gene phylogeny *A.choestoea* is in a clade with species from Alabama, Tennessee, and Kentucky. Hydrochory also provides a compelling explanation for the occurrence of this western lineage in South Carolina. The Tugaloo River, along which *A.choestoea* occurs, captured the Tallulah and Chattooga Rivers during the Pleistocene (Voss et al. 1993), rerouting their waters to the Atlantic Ocean via the Savannah River watershed. Prior to this, both the Tallulah and Chattooga Rivers entered the ancestral Chattahoochee River, which eventually flowed to the Gulf of Mexico. The ancestral Chattahoochee River may have been a barrier separating the eastern and western *sinuaticollis* group lineages, and capture by the Tugaloo River could have transported *A.choestoea* (or its ancestor) into the Savannah River drainage. As Fig. 35 shows, most of the land along the Tugaloo River drainage remains to be explored for members of the *sinuaticollis* group, and the clade is entirely unknown from Georgia. Discovery of *sinuaticollis* group specimens along the Chattooga River and Chattahoochee Rivers would allow testing of this biogeographical hypothesis.

The remaining *Anillinus* species found in South Carolina belong to the *langdoni* group and *loweae* group, which make up part of the well-supported “LSL clade”. This large clade includes the *Anillinus* species most commonly collected in litter samples in the Southern Appalachians. The *langdoni* group has been found in four disjunct regions (Fig. 43E): northwestern Virginia and adjacent West Virginia (*A.virginiae*), the South Mountains in western North Carolina, the Cumberland Plateau in southeastern Kentucky, and the Southern Appalachians southwest of the FBR. The gap between *A.virginiae* and the Southern Appalachian *langdoni*-group species does not correspond to any obvious biogeographic barriers, but a somewhat similar gap is seen in the distribution of *Serranillus* (Fig. 1A). The sister lineage to the Southern Appalachian species is not *A.virginiae* but a surprising clade that spans the Tennessee River Valley, consisting of the Kentucky endemic *A.balli* and an undescribed species from northern Georgia. The single *langdoni*-group species known from South Carolina, A.cf.nantahala, belongs to the Southern Appalachian lineage. Anillinuscf.nantahala is the most widespread species in the clade, ranging from the western flank of the Unicoi Mountains in eastern Tennessee to northwestern South Carolina and eastern Georgia (Fig. 38). The South Carolina specimen was collected from a litter sample taken from a mesic stream hollow near the Chattooga River gorge.

The *loweae* group has a smaller range, limited to the Southern Appalachians (Fig. 43G). Collectively, the range of the *loweae* group skirts the FBR above its headwaters, as in *S.dunavani* and *A.murrayae*, with *A.fortis* occurring in several mountain ranges to the northeast, including elevations above 1650 m on Big Bald in the Bald Mountains. The two most commonly collected species of the *loweae* group, *A.loweae* and *A.cherokee*, can be readily collected from rather shallow leaf litter where they occur. Specimens of *A.loweae* have been captured in traditional pitfall traps in Great Smoky Mountains National Park (National Ecological Observatory Network data), which attests to their active movement above-ground, as does their extensive geographic range and wide elevational distribution (Sokolov and Carlton 2010). In South Carolina, *A.loweae* is known only from Sassafras Mountain, the highest mountain in the state, while *A.cherokee* is known from several localities further west. The third described species, *A.merritti*, is primarily endogean in habit and is only rarely collected in leaf litter. Its range is similar to that of *A.cherokee* (Fig. 38).

Females of *A.merritti* are unusually variable in external structure; in some populations east of the Little Tennessee River, the humeri are strongly sloped and constricted, giving the body an hourglass-shaped appearance (Fig. 39A). Such a body form, otherwise known in *Anillinus* only in females of some *moseleyae*-group species, has been hypothesized to be an adaptation to endogean existence, allowing greater flexibility to move through tighter interstices (Sokolov 2013). Only females of these *A.merritti* populations have the hourglass-shaped body, so either females have adapted to reach deeper strata, possibly for oviposition, or the shape is involved in sexual recognition. The area occupied by hourglass-shaped *A.merritti* is also where the greatest local diversity of *loweae*-group species is found; at Rabun Bald, all three described species occur. Competition and/or detrimental interbreeding between these closely related species could have also been selective factors. Like several other more montane anillines, *A.merritti* is known in South Carolina only from the extreme northwest corner of the state.

Habitats used by members of the *langdoni* and *loweae* groups may explain aspects of their distributions. For example, members of the *langdoni* group are typically collected in leaf litter, and seem to be strongly associated with primary-growth forest (Ferro et al. 2012). Strong association with such habitats would limit their occurrence in South Carolina to the few relatively undisturbed steep hollows at higher elevations in the northwest corner of the state.

### ﻿﻿Conservation of Appalachian Anillini

While all anillines are short range (or micro-range) endemics with apparently limited dispersal capabilities, there are currently none officially listed as of conservation concern, although it has been suggested that they deserve such ranking (Cornell 1979). Anillines are difficult to collect and therefore are rarely detected in non-specialist survey efforts, which can skew perceptions about rarity. For example, in the first symposium of endangered species of South Carolina (Brooks et al. 1979), *Anillinusdunavani* Jeannel (now placed in the genus *Serranillus*) was described as rare and “only known from type specimen”; in fact, *Serranillusdunavani* is the most abundant and commonly collected anilline in South Carolina. On the other hand, our study has shown that many more species of anillines exist in the eastern United States than are formally recognized, and most of them occur at lower elevations that receive less conservation attention than montane habitats (Timpe et al. 2009). It is important to note that almost all of the forested habitats in which anillines currently occur have been heavily impacted by humans throughout recent history by logging and agriculture. At Long Cane Creek in Abbeville Co., SC, for example, one of the most diverse and important anilline sites in the state, the woods are scarred by deep gullies formed by erosion from poor farming practices when the area would have been a barren cotton field (Fig. 25C). The persistence of anillines through such periods of habitat destruction and alteration is incredible, and offers some hope that at least the endogean species are resilient to these disturbances.

However, it is unlikely that anillines could survive “development” of a site, which necessarily requires heavy compaction of the soil to support man-made structures. The Piedmont ecoregion of the southeastern United States is rapidly undergoing such habitat destruction, and anilline species are likely being lost before being even being discovered. The damage that such losses could cause to our understanding of biodiversity are best illustrated by *A.dentatus* and *A.jancae*, the most phylogenetically important species of anillines in South Carolina, both known only from a small area in the vicinity of Long Cane Creek, Abbeville County. The discovery of such unusual anillines in the outer Piedmont of the southeastern United States reflects the fact that the soil and litter arthropod fauna in the region has been generally overlooked. In reference to millipedes, Richard Hoffman (1963) wrote that the species inhabiting this area “are as poorly known as those of any comparable area in the world.” Examples of phylogenetically and morphologically isolated taxa found in the vicinity of Long Cane Creek include the millipede *Parvulodesmusprolixogonus* (Shelley 1983) and the spider *Epiceraticelusmandyae* (Draney et al. 2019). Disjunct occurrences of Appalachian endemics are also known, including beetles such as the stayphylinid *Dasyceruscarolinensis* (Caterino and Harden 2024), otherwise known only from mountains northeast of the French Broad River, and the carabid *Pterostichusacutipes* Barr, otherwise known in South Carolina only on the Blue Ridge Escarpment (CUAC data).

These concerns are not restricted to small and cryptic speices. For example, unusual populations of the carabid subgenera Pterostichus (Gastrosticta) and Dicaelus (Paradicaelus) have been discovered in the outer Piedmont of South Carolina in the past two decades, and likely represent new species (CUAC and AMDc data). The latter are relatively large, more than 20 mm in length, with distinctive external and genitalic characters. That such conspicuous taxa can still be discovered in the eastern United States emphasizes the desperate need for baseline bioinventory work in the Piedmont of the southeastern United States. The situation for anillines must be even more desperate.

## ﻿﻿Conclusions

The Anillini of the Eastern United States comprise a unique component of the region’s rich biodiversity. Conservatively, 148 species of anillines are now known from east of the Mississippi River. The majority of these remain undescribed (Suppl. material 3). The systematic framework that we present here will facilitate description and classification of these species and others that will doubtlessly be discovered in the future. While our sampling has been relatively thorough, large swaths of the region are entirely unexplored for endogean anillines, and additional unique lineages such as those represented by *A.dentatus* and *A.jancae* may remain to be discovered. We hope that our contributions will influence more biologists to focus on this surprising and diverse tribe of beetles.

## Supplementary Material

XML Treatment for Anillini

XML Treatment for Serranillus

XML Treatment for Serranillus dunavani

XML Treatment for Serranillus jeanneli

XML Treatment for Serranillus monadnock

XML Treatment for Serranillus

XML Treatment for Anillinus

XML Treatment for Anillinus dentatus

XML Treatment for Anillinus jancae

XML Treatment for Anillinus chandleri

XML Treatment for Anillinus

XML Treatment for Anillinus monadnock

XML Treatment for Anillinus murrayae

XML Treatment for Anillinus cornelli

XML Treatment for Anillinus castaneus

XML Treatment for Anillinus simplex

XML Treatment for Anillinus arenicollis

XML Treatment for Anillinus montrex

XML Treatment for Anillinus choestoea

XML Treatment for Anillinus mica

XML Treatment for Anillinus micamicus

XML Treatment for Anillinus seneca

XML Treatment for Anillinus

XML Treatment for Anillinus cf. nantahala

XML Treatment for Anillinus

XML Treatment for Anillinus cherokee

XML Treatment for Anillinus loweae

XML Treatment for Anillinus merritti

XML Treatment for Anillinus

## ﻿﻿Additional information

### Author ORCIDs

Curt W. Harden https://orcid.org/0000-0002-5226-8725

Michael S. Caterino https://orcid.org/0000-0002-2597-5707

### Data availability

The data underpinning the analyses reported in this paper are deposited in the Dryad Data Repository at https://doi.org/10.5061/dryad.g79cnp5z3.

## Supplementary materials

﻿Supplementary material 1 Trees from maximum likelihood analyses of single gene and 6-gene matrices Data type pdf https://binary.pensoft.net/file/1110383This dataset is made available under the Open Database License (http://opendatacommons.org/licenses/odbl/1.0/). The Open Database License (ODbL) is a license agreement intended to allow users to freely share, modify, and use this Dataset while maintaining this same freedom for others, provided that the original source and author(s) are credited.Curt W. Harden, Michael S. Caterino

﻿Supplementary material 2 Taxa sampled for molecular phylogenetics and associated GenBank accession numbers Data type docx https://binary.pensoft.net/file/1110384This dataset is made available under the Open Database License (http://opendatacommons.org/licenses/odbl/1.0/). The Open Database License (ODbL) is a license agreement intended to allow users to freely share, modify, and use this Dataset while maintaining this same freedom for others, provided that the original source and author(s) are credited.Curt W. Harden, Michael S. Caterino

﻿Supplementary material 3 Systematic checklist of eastern Nearctic Anillini Data type docx https://binary.pensoft.net/file/1110385This dataset is made available under the Open Database License (http://opendatacommons.org/licenses/odbl/1.0/). The Open Database License (ODbL) is a license agreement intended to allow users to freely share, modify, and use this Dataset while maintaining this same freedom for others, provided that the original source and author(s) are credited.Curt W. Harden, Michael S. Caterino
