# Supplementary material for: ﻿Systematics and biogeography of Appalachian Anillini, and a taxonomic review of the species of South Carolina (Coleoptera, Carabidae, Trechinae, Anillini)
Source: Zookeys. 2024 Aug 8;1209:69–197. doi: 10.3897/zookeys.1209.125897 (PMC11336398; doi:10.3897/zookeys.1209.125897)
Supplement: ﻿Supplementary material 1 — Trees from maximum likelihood analyses of single gene and 6-gene matrices [file zookeys-1209-069_article-125897__-s001.pdf]

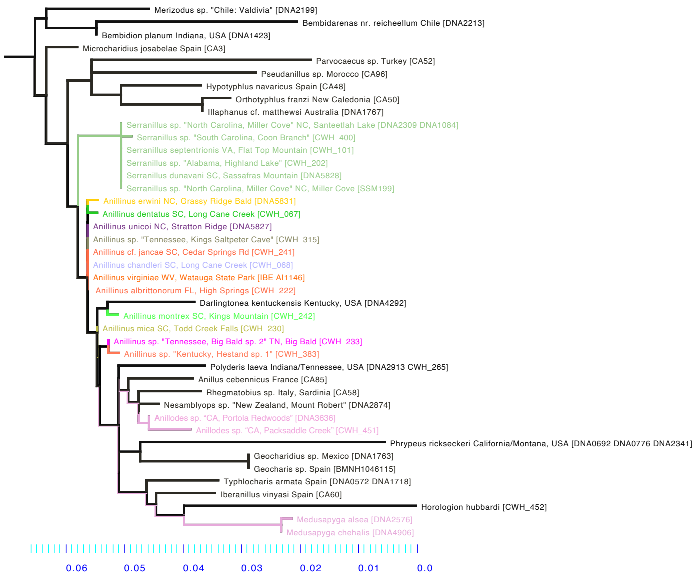

18S ML tree

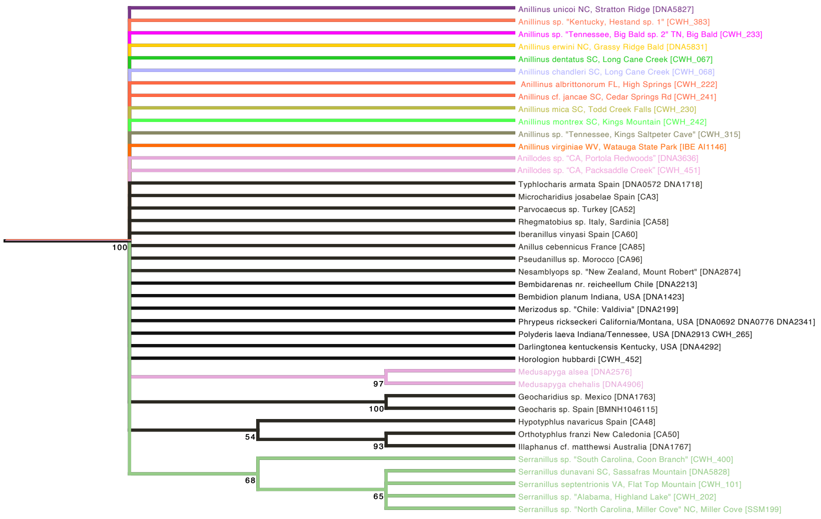

18S SBS majority rule consensus tree

Figure S1. Maximum likelihood trees of 18S.

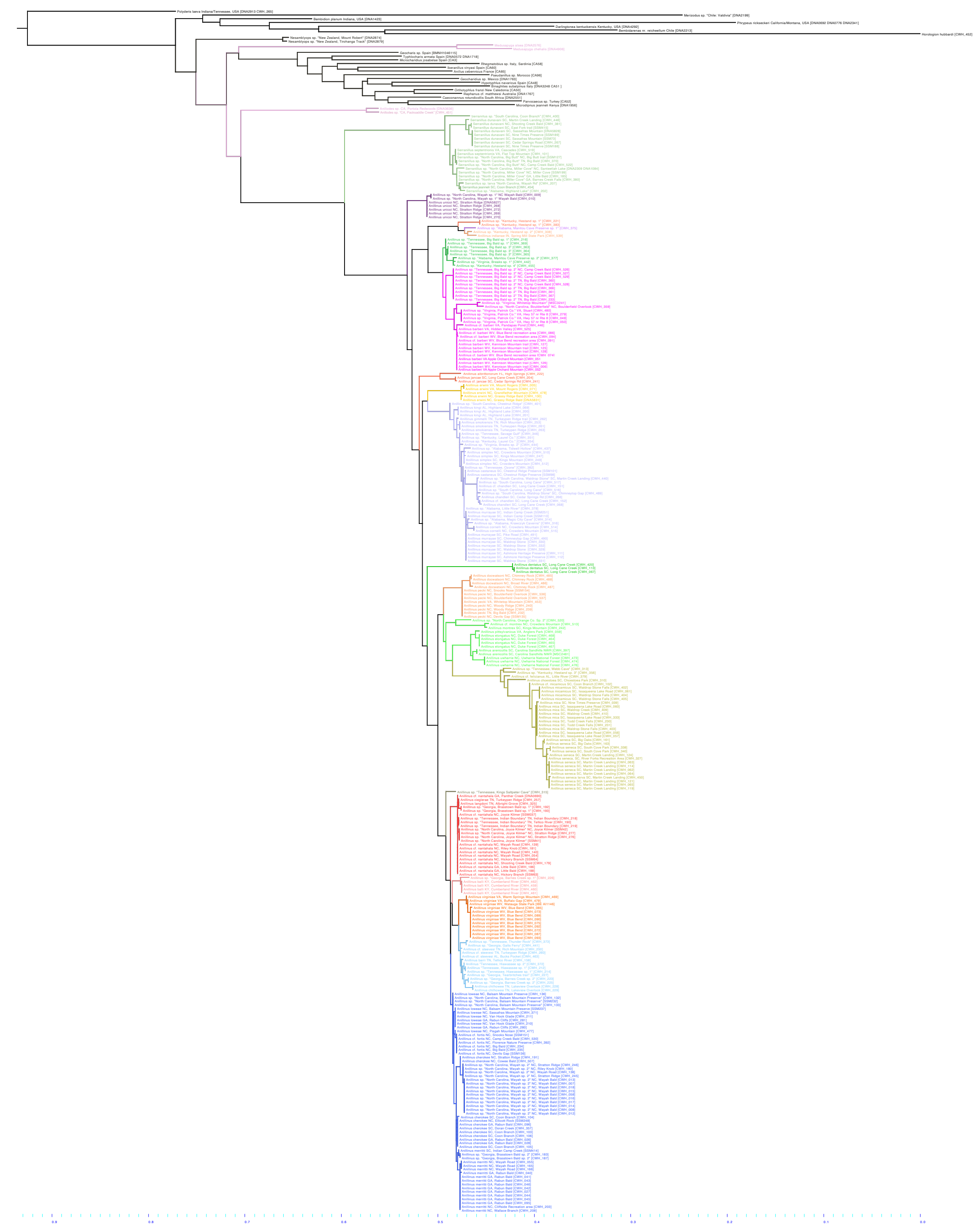

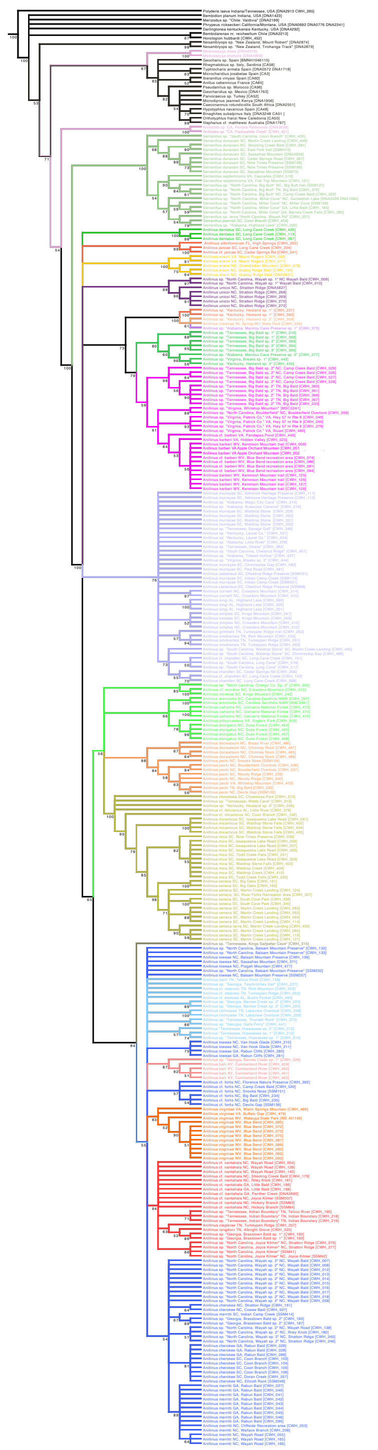

28S no GBLOCKS ML tree

28S no GBLOCKS
SBS majority rule consensus tree

Figure S2. Maximum likelihood trees of 28S no GBLOCKS.

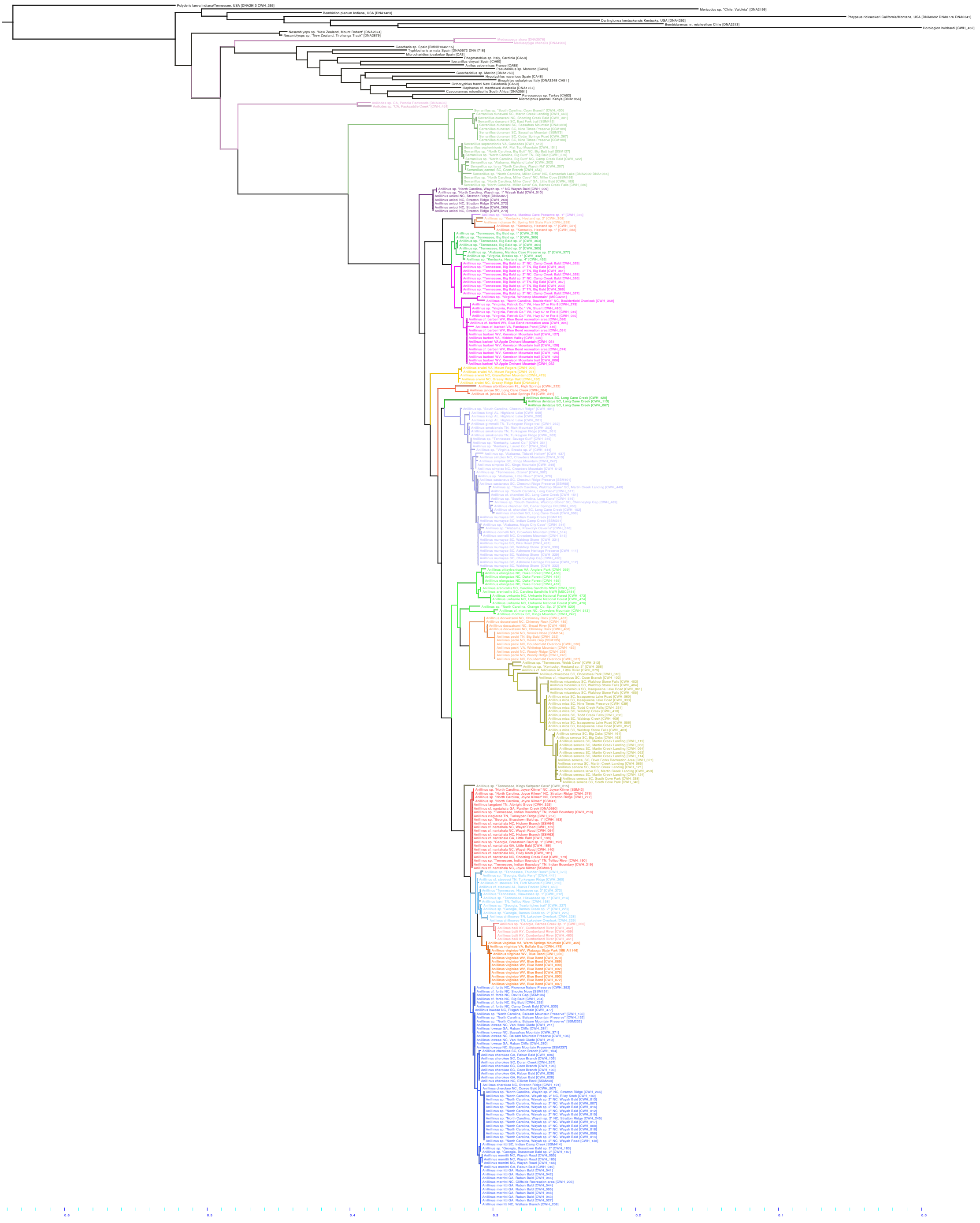

28S GBLOCKS ML tree

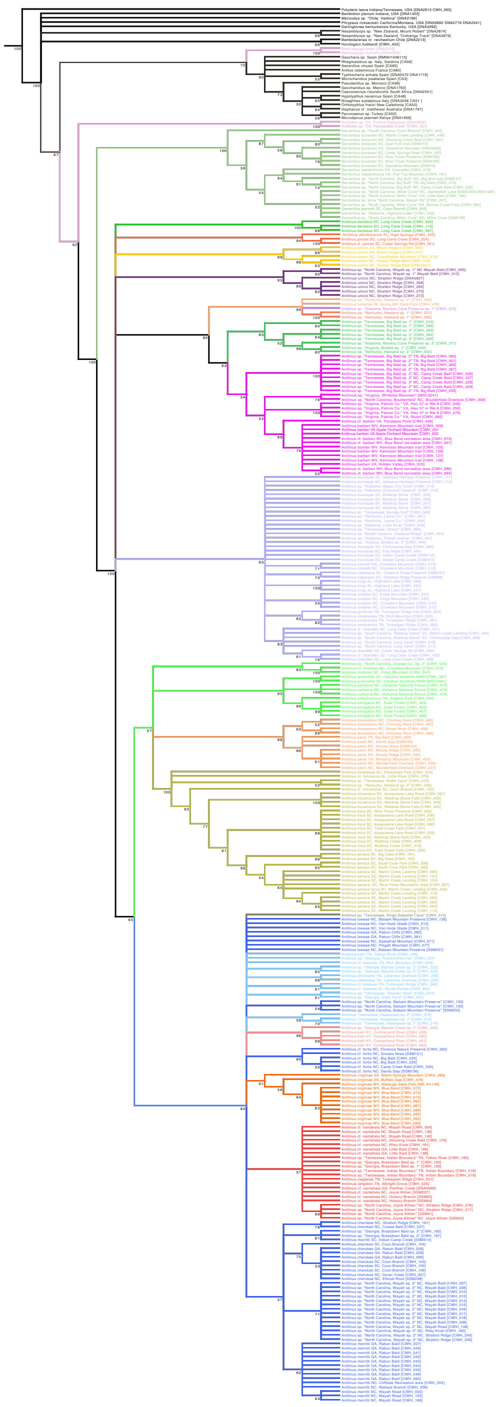

28S GBLOCKS
SBS majority rule consensus tree

Figure S3. Maximum likelihood trees of 28S with GBLOCKS.

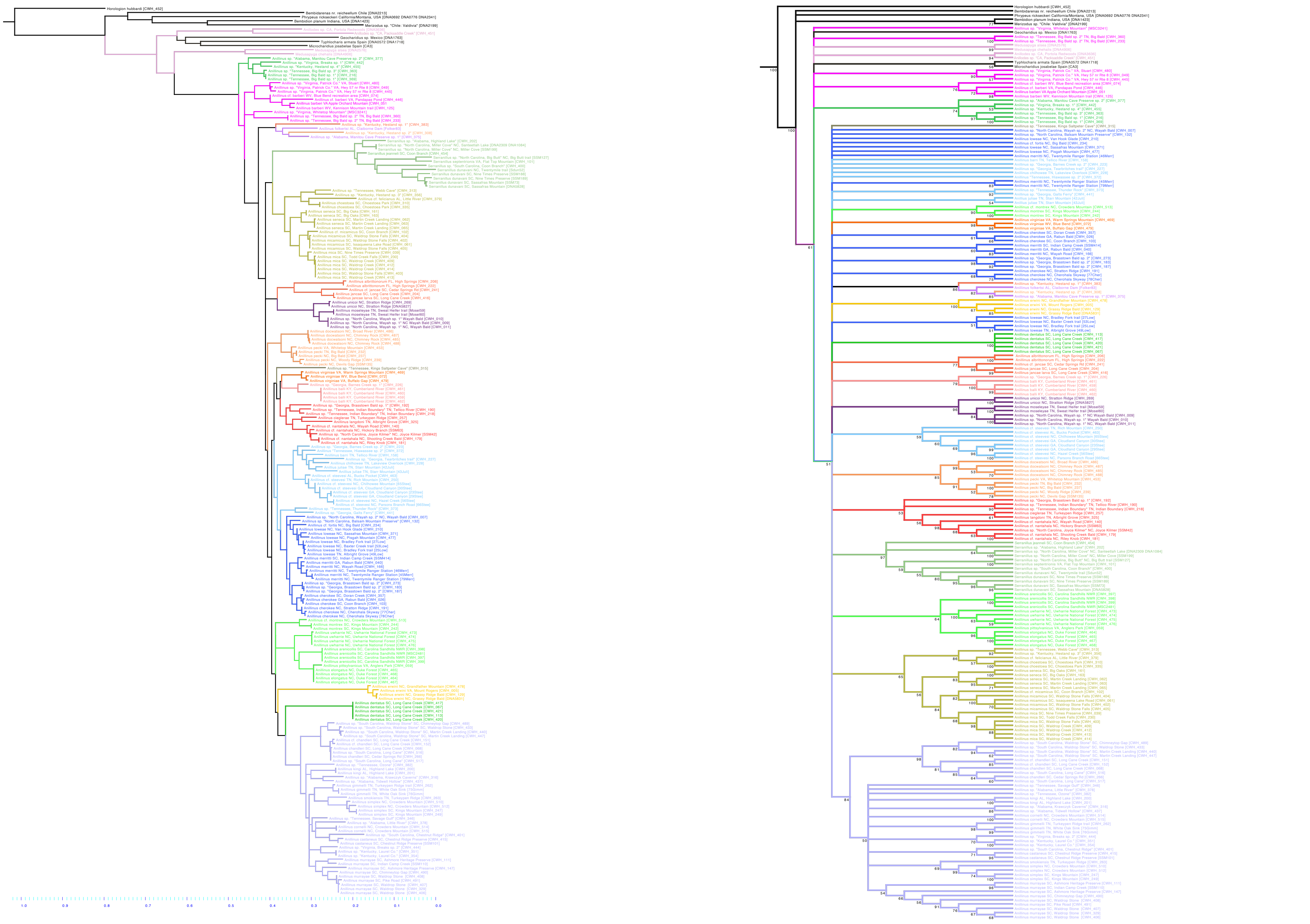

***COIbc* ML tree**

***COIbc* SBS majority rule consensus tree**

Figure S4. Maximum likelihood trees of *COIbc*.

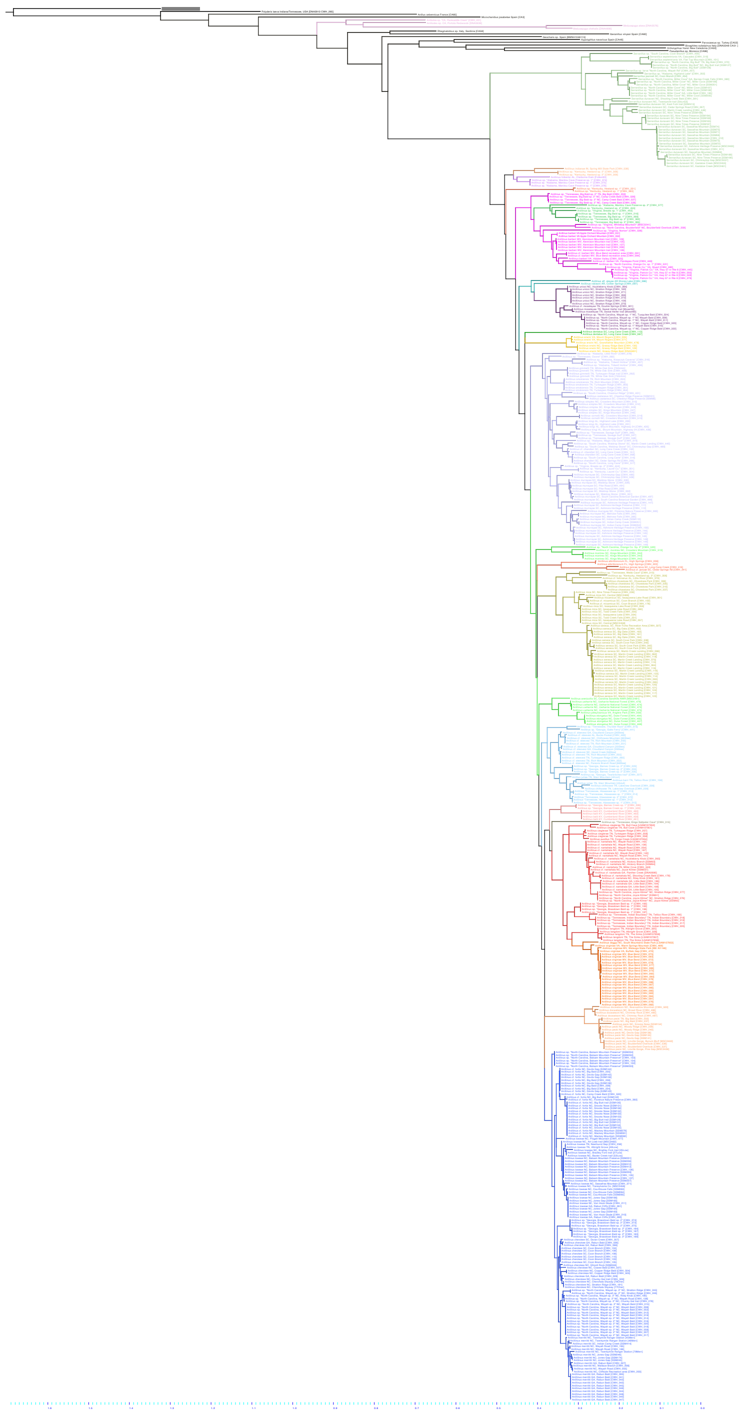

***COIjp* ML tree**

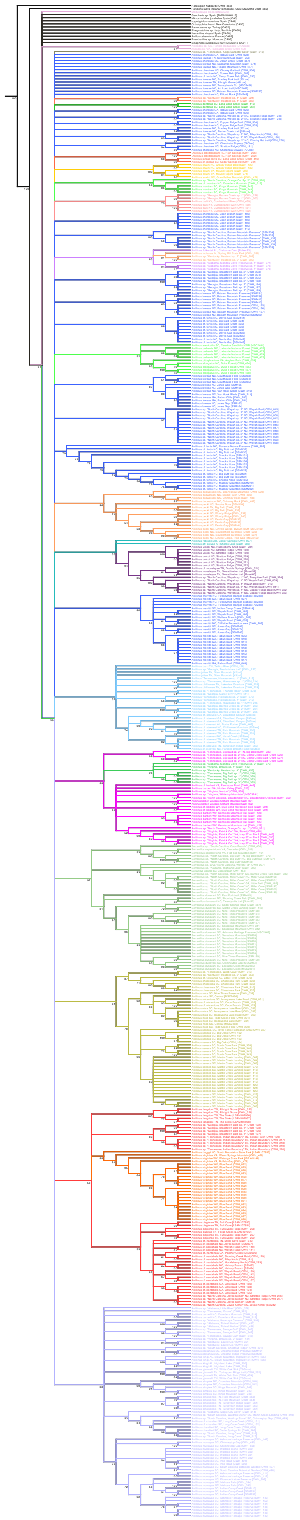

***COIjp* SBS majority rule consensus tree**

Figure S5. Maximum likelihood trees of *COIjp*.

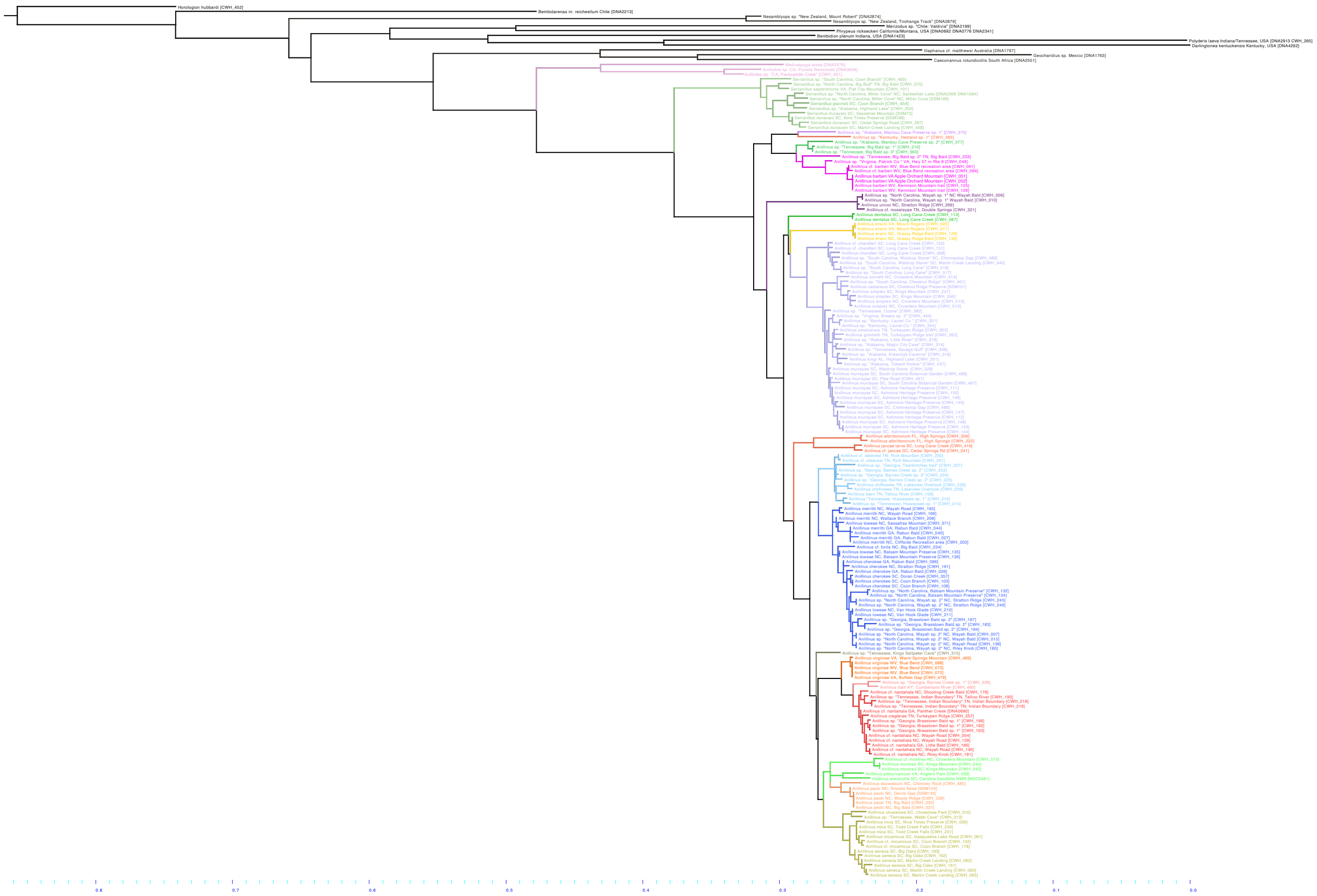

***CAD2* ML tree**

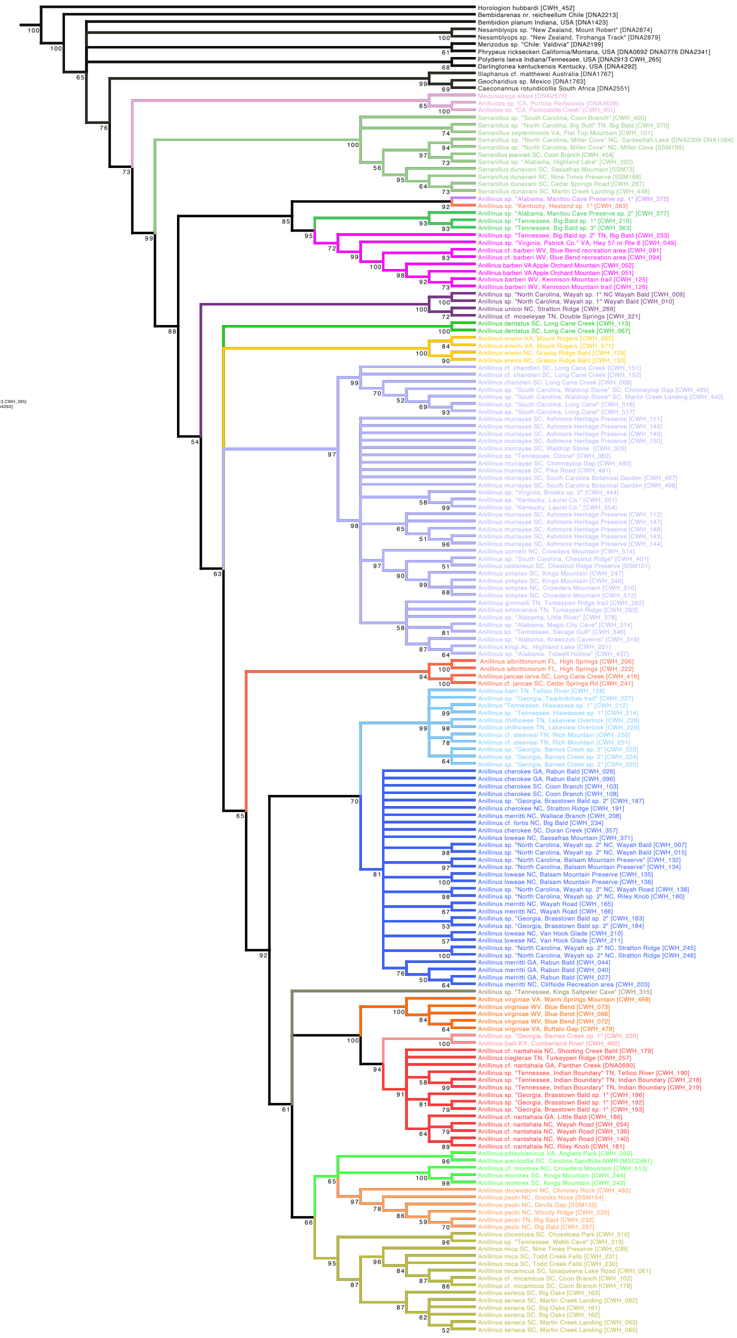

***CAD2* SBS majority rule consensus tree**

Figure S6. Maximum likelihood trees of *CAD2*.

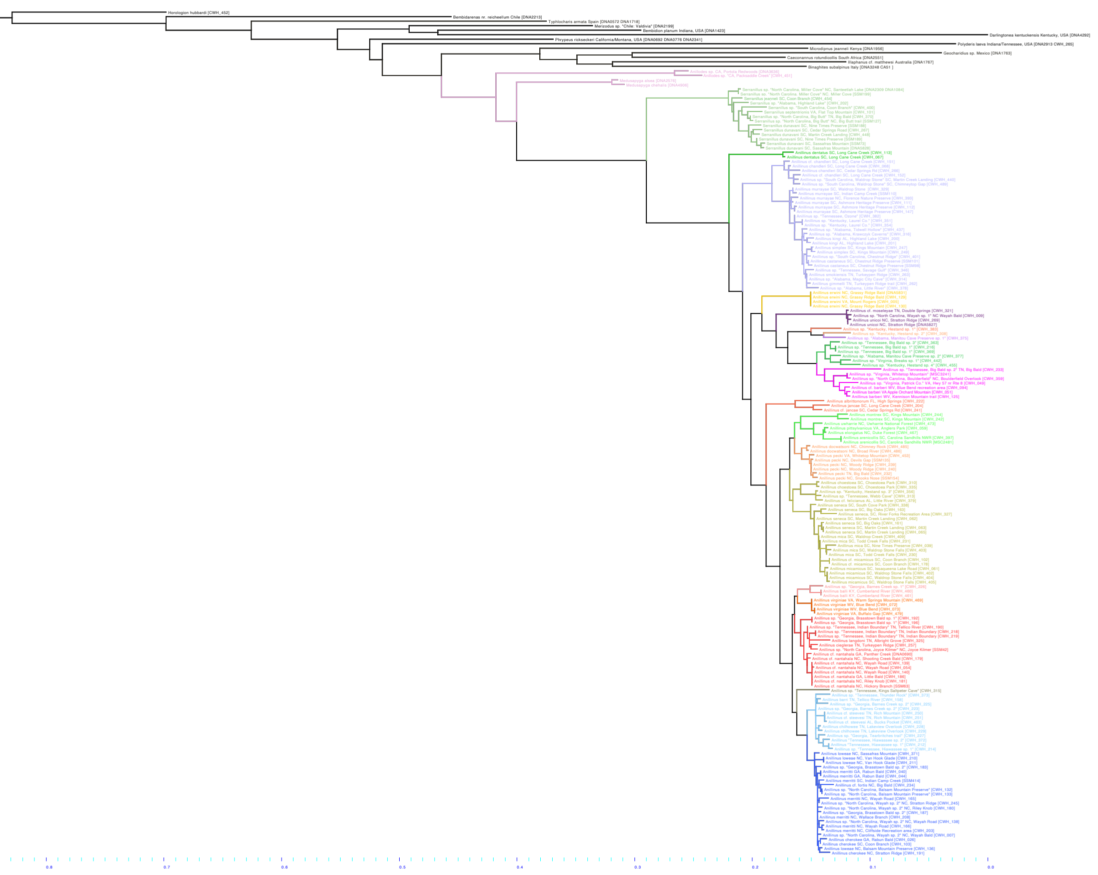

***CAD4* ML tree**

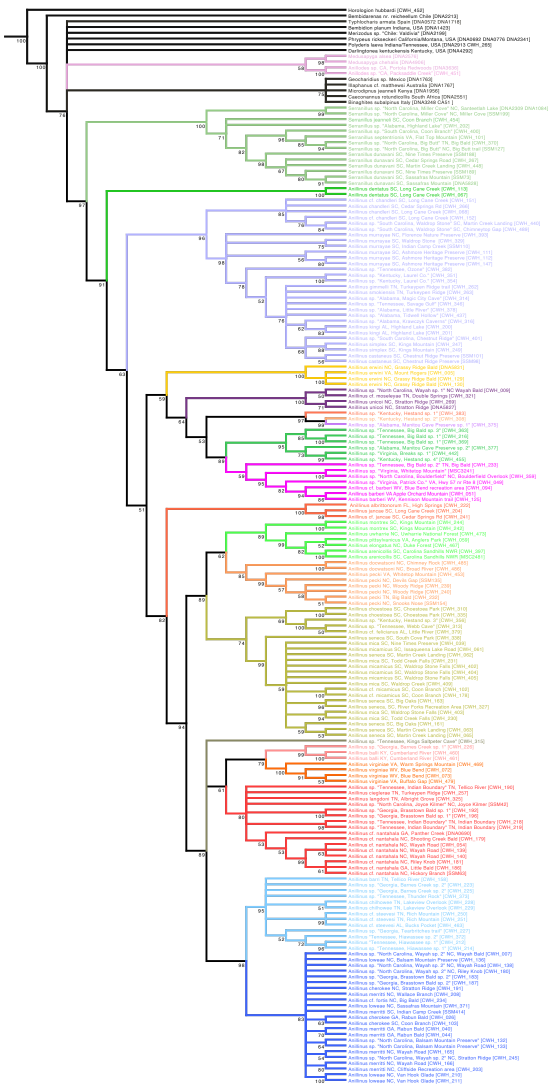

***CAD4* SBS majority rule consensus tree**

Figure S7. Maximum likelihood trees of *CAD4*.

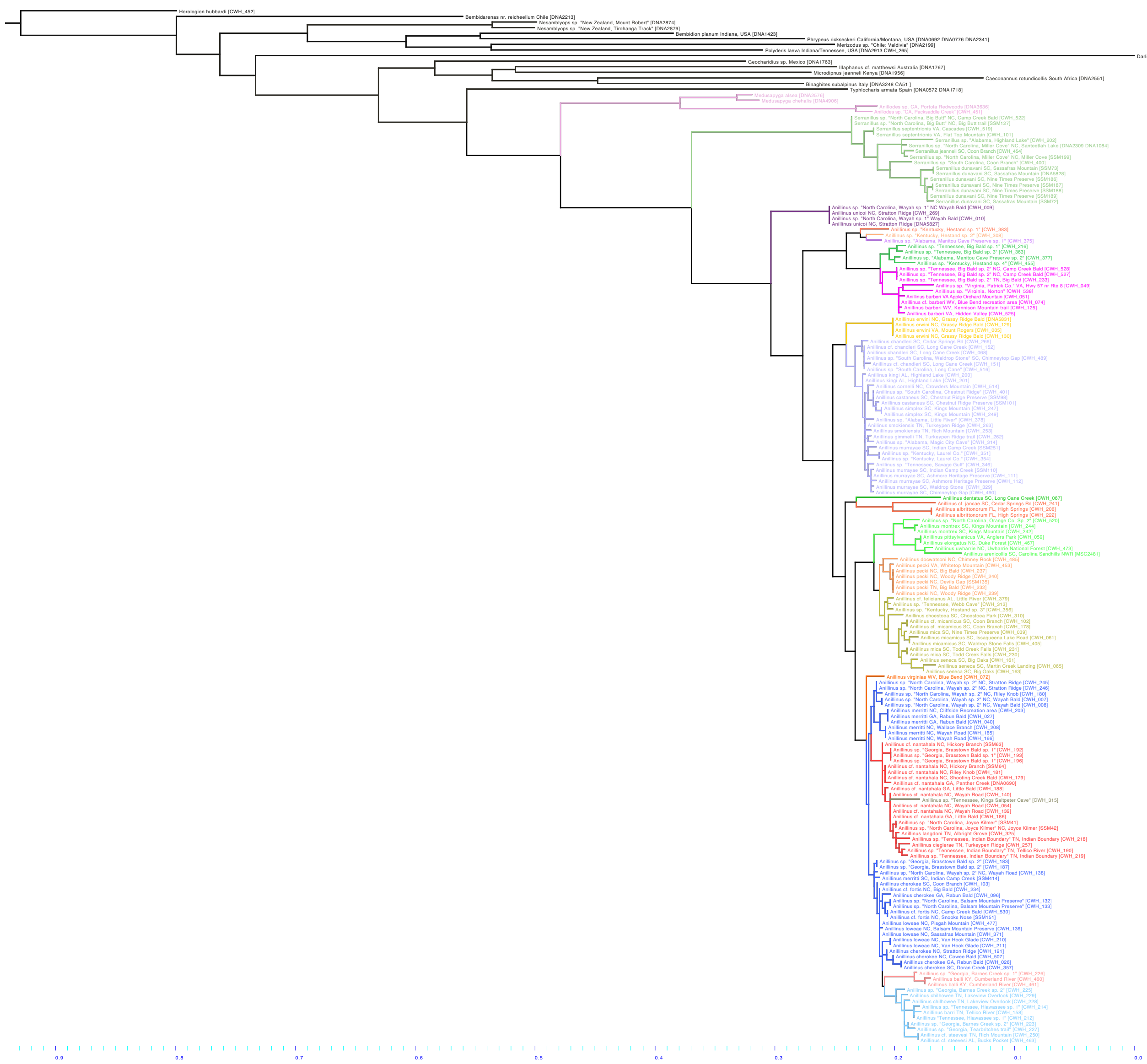

***Wg* ML tree**

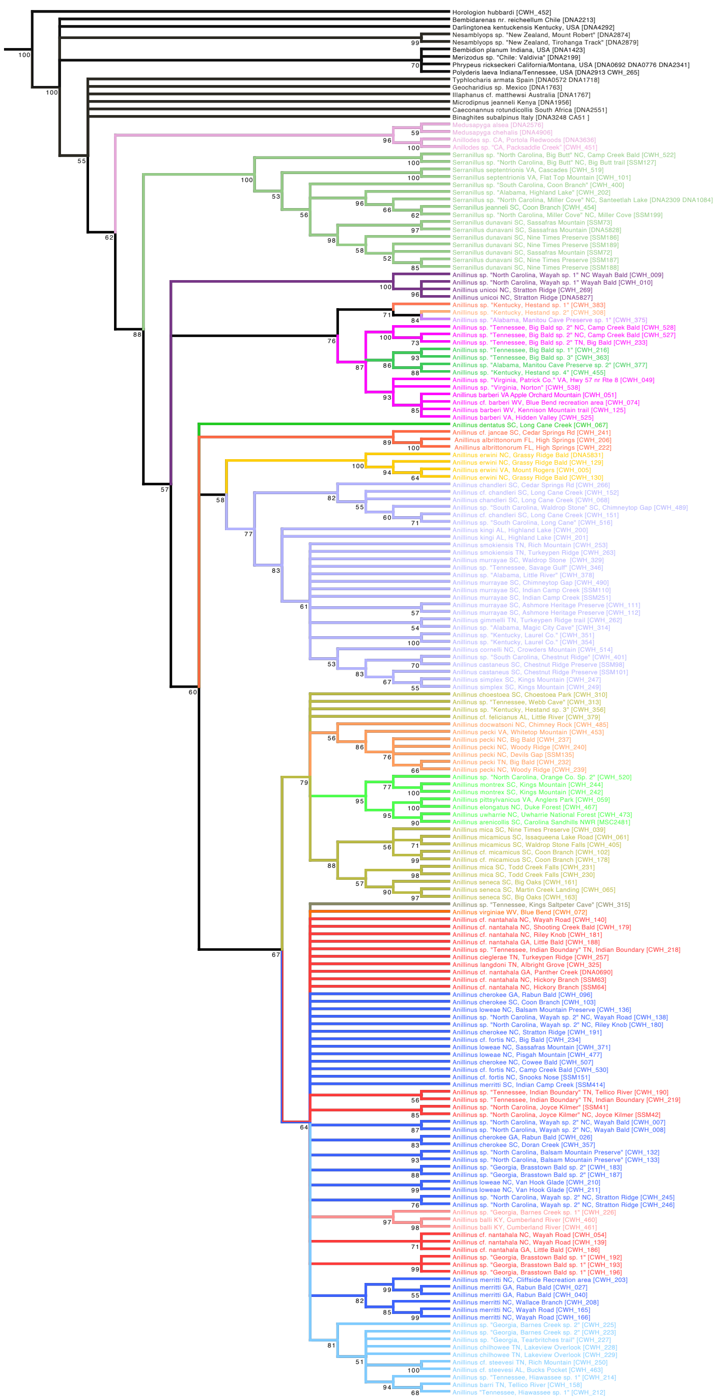

***Wg* SBS majority rule consensus tree**

Figure S8. Maximum likelihood trees of *Wg*.

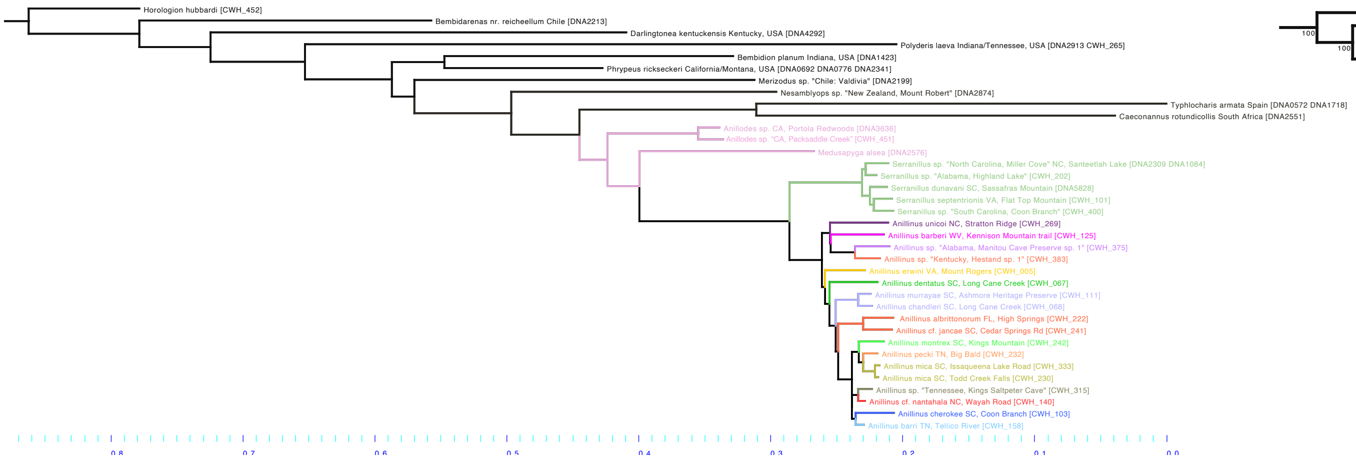

***MSP* ML tree**

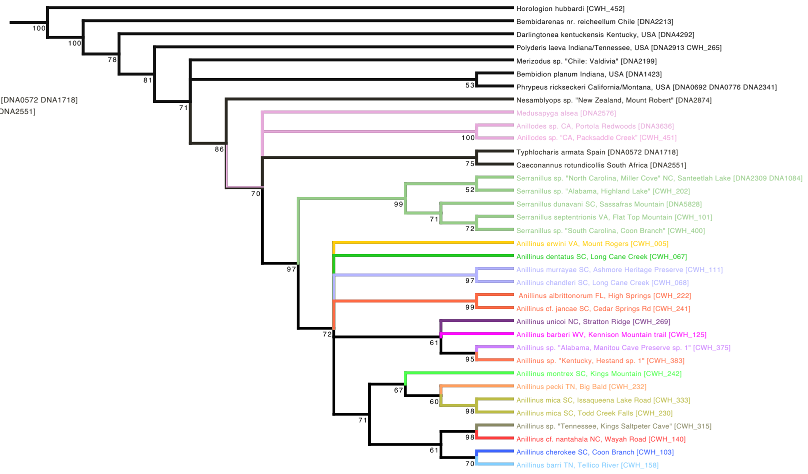

***MSP* SBS majority rule consensus tree**

Figure S9. Maximum likelihood trees of *MSP*.

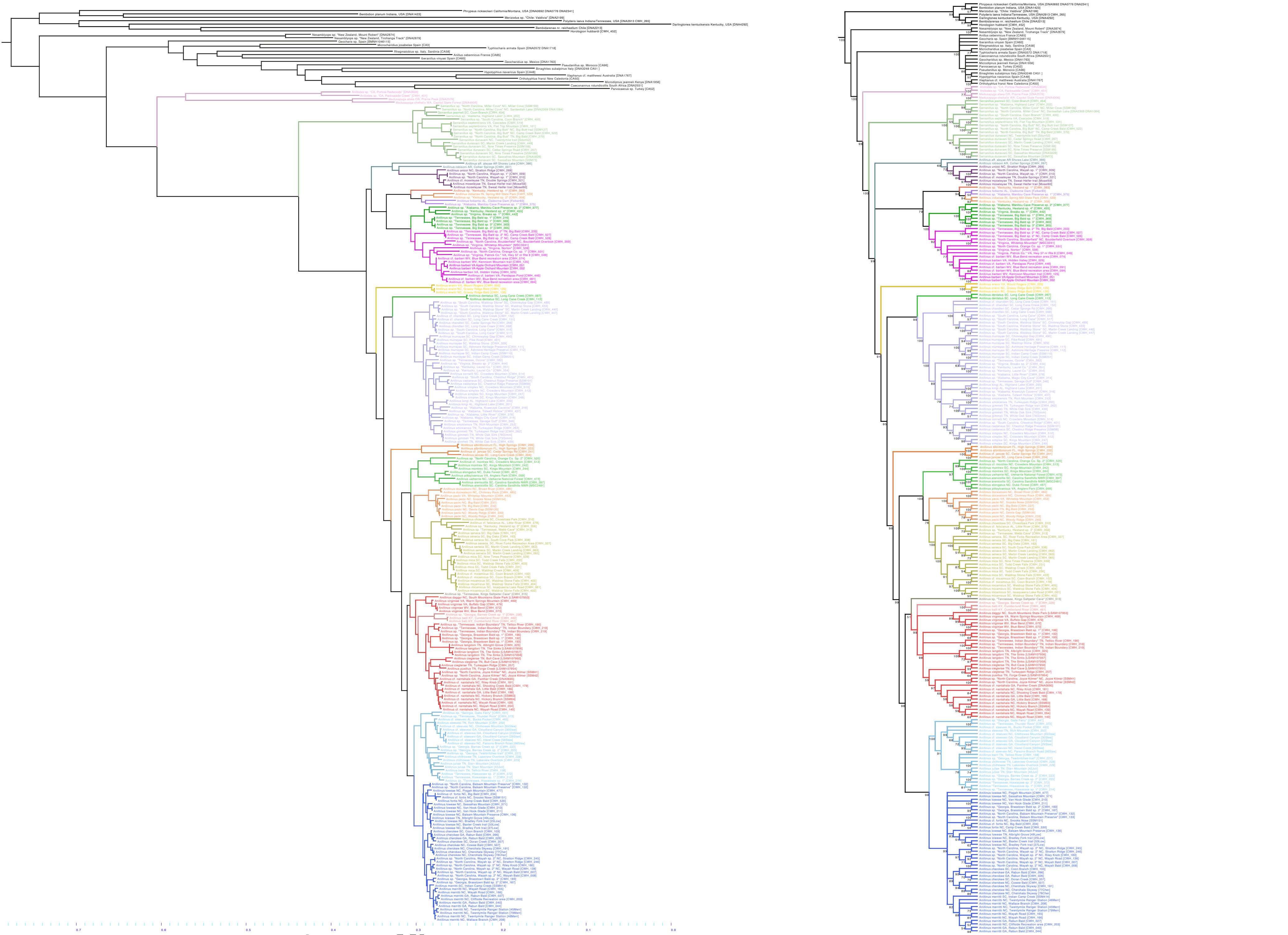

6-gene ML tree

6-gene SBS majority rule consensus tree

Figure S10. Maximum likelihood trees of the 6-gene core matrix.
