# Supplementary material for: ﻿Systematics and biogeography of Appalachian Anillini, and a taxonomic review of the species of South Carolina (Coleoptera, Carabidae, Trechinae, Anillini)
Source: Zookeys. 2024 Aug 8;1209:69–197. doi: 10.3897/zookeys.1209.125897 (PMC11336398; doi:10.3897/zookeys.1209.125897)
Supplement: ﻿Supplementary material 2 — Taxa sampled for molecular phylogenetics and associated GenBank accession numbers [file zookeys-1209-069_article-125897__-s002.docx]

|  | **Identifier Code** | **18S** | **28S** | ***COIbc*** | ***COIjp*** | ***CAD2*** | ***CAD4*** | ***wg*** | ***MSP*** |
| --- | --- | --- | --- | --- | --- | --- | --- | --- | --- |
| **Arkansas *Anillinus*** |  |  |  |  |  |  |  |  |  |
| *Anillinus* aff*. aleyae* Sokolov & Watrous AR, Shores Lake | CWH_386 | **—** | **—** | **—** | **OR839660** | **—** | **—** | **—** | **—** |
| *Anillinus robisoni* Sokolov & Carlton AR, Collier Springs | CWH_097 | **—** | **—** | **—** | **OR839447** | **—** | **—** | **—** | **—** |
| ***Anillinus* “*moseleyae*-group”** |  |  |  |  |  |  |  |  |  |
| *Anillinus* cf*. moseleyae* Sokolov & Carlton TN, Double Springs | CWH_321 | **—** | **—** | **—** | **OR839615** | **OR837902** | **OR838048** | **—** | **—** |
| *Anillinus moseleyae* Sokolov & Carlton TN, Sweat Heifer trail | Mosel59 | **—** | **—** | GU573760 | GU573760 | **—** | **—** | **—** | **—** |
| *Anillinus moseleyae* Sokolov & Carlton TN, Sweat Heifer trail | Mosel60 | **—** | **—** | GU573761 | GU573761 | **—** | **—** | **—** | **—** |
| *Anillinus* sp. "North Carolina, Wayah sp. 1" NC Wayah Bald | CWH_009 | **—** | **OR853383** | **OR839355** | **OR839378** | **OR837784** | **OR837949** | **OR838138** | **—** |
| *Anillinus* sp. "North Carolina, Wayah sp. 1" NC Wayah Bald | CWH_010 | **—** | **OR853384** | **OR839356** | **OR839379** | **OR837785** | **—** | **OR838139** | **—** |
| *Anillinus* sp. "North Carolina, Wayah sp. 1" NC, Copper Ridge | CWH_322 | **—** | **—** | **—** | **OR839616** | **—** | **—** | **—** | **—** |
| *Anillinus* sp. "North Carolina, Wayah sp. 1" NC, Copper Ridge | CWH_323 | **—** | **—** | **—** | **OR839617** | **—** | **—** | **—** | **—** |
| *Anillinus* sp. "North Carolina, Wayah sp. 1" NC, Tusquitee Bald | CWH_324 | **—** | **—** | **—** | **OR839618** | **—** | **—** | **—** | **—** |
| *Anillinus* sp. "North Carolina, Wayah sp. 1" NC, Wayah Bald | CWH_011 | **—** | **—** | **OR839354** | **OR839380** | **—** | **—** | **—** | **—** |
| *Anillinus unicoi* Sokolov NC, Huckleberry Knob | CWH_284 | **—** | **—** | **—** | **OR839605** | **—** | **—** | **—** | **—** |
| *Anillinus unicoi* Sokolov NC, Stratton Ridge | CWH_159 | **—** | **—** | **—** | **OR839498** | **—** | **—** | **—** | **—** |
| *Anillinus unicoi* Sokolov NC, Stratton Ridge | CWH_160 | **—** | **—** | **—** | **OR839499** | **—** | **—** | **—** | **—** |
| *Anillinus unicoi* Sokolov NC, Stratton Ridge | CWH_268 | **—** | **OR853366** | **—** | **—** | **—** | **—** | **—** | **—** |
| *Anillinus unicoi* Sokolov NC, Stratton Ridge | CWH_269 | **—** | **OR853367** | **OR839345** | **OR839591** | **OR837896** | **OR838041** | **OR838230** | **OR838128** |
| *Anillinus unicoi* Sokolov NC, Stratton Ridge | CWH_270 | **—** | **OR853365** | **—** | **OR839592** | **—** | **—** | **—** | **—** |
| *Anillinus unicoi* Sokolov NC, Stratton Ridge | CWH_271 | **—** | **—** | **—** | **OR839593** | **—** | **—** | **—** | **—** |
| *Anillinus unicoi* Sokolov NC, Stratton Ridge | CWH_272 | **—** | **OR853368** | **—** | **OR839594** | **—** | **—** | **—** | **—** |
| *Anillinus unicoi* Sokolov NC, Stratton Ridge | DNA5827 | ON557660 | ON557652 | ON554761 | **—** | **—** | ON721283 | ON721287 | **—** |
| ***Anillinus* “Hestand sp. 1”** |  |  |  |  |  |  |  |  |  |
| *Anillinus* sp. "Kentucky, Hestand sp. 1" | CWH_383 | **OR853115** | **OR853359** | **OR839339** | **OR839659** | **OR837915** | **OR838070** | **OR838249** | **OR838132** |
| *Anillinus* sp. "Kentucky, Hestand sp. 1" | CWH_221 | **—** | **OR853358** | **—** | **OR839544** | **—** | **—** | **—** | **—** |
| ***Anillinus* “*indianae*-group”** |  |  |  |  |  |  |  |  |  |
| *Anillinus indianae* Jeannel IN, Spring Mill State Park | CWH_539 | **—** | **OR853231** | **—** | **OR839735** | **—** | **—** | **—** | **—** |
| *Anillinus* sp. "Kentucky, Hestand sp. 2" | CWH_308 | **—** | **OR853360** | **OR839340** | **OR839606** | **—** | **OR838042** | **OR838231** | **—** |
| *Anillinus* sp. "Kentucky, Hestand sp. 2" | CWH_309 | **—** | **—** | **—** | **OR839607** | **—** | **—** | **—** | **—** |
| ***Anillinus* “*folkertsi*-group”** |  |  |  |  |  |  |  |  |  |
| *Anillinus folkertsi* Sokolov & Carlton AL, Claiborne Dam | Folker83 | — | — | GU573762 | GU573762 | — | — | — | — |
| *Anillinus* sp. "Alabama, Manitou Cave Preserve sp. 1" | CWH_375 | **—** | **OR853262** | **OR839282** | **OR839651** | **OR837911** | **OR838065** | **OR838245** | **OR838131** |
| *Anillinus* sp. "Alabama, Manitou Cave Preserve sp. 1" | CWH_374 | **—** | **—** | **—** | **OR839650** | **—** | **—** | **—** | **—** |
| *Anillinus* sp. "Alabama, Manitou Cave Preserve sp. 1" | CWH_376 | **—** | **—** | **—** | **OR839652** | **—** | **—** | **—** | **—** |
| ***Anillinus* “*barberi*-group”** |  |  |  |  |  |  |  |  |  |
| *Anillinus barberi* Jeannel VA, Apple Orchard Mountain | CWH_051 | **—** | **OR853131** | **OR839205** | **OR839404** | **OR837793** | **OR837955** | **OR838145** | **—** |
| *Anillinus barberi* Jeannel VA, Apple Orchard Mountain | CWH_052 | **—** | **OR853128** | **—** | **OR839405** | **OR837794** | **—** | **—** | **—** |
| *Anillinus barberi* Jeannel VA, Hidden Valley | CWH_525 | **—** | **OR853132** | **—** | **OR839726** | **—** | **—** | **OR838272** | **—** |
| *Anillinus barberi* Jeannel WV, Kennison Mountain Trail | CWH_006 | **—** | **OR853133** | **—** | **OR839375** | **—** | **—** | **—** | **—** |
| *Anillinus barberi* Jeannel WV, Kennison Mountain Trail | CWH_125 | **—** | **OR853134** | **OR839206** | **OR839470** | **OR837817** | **OR837973** | **OR838160** | **OR838119** |
| *Anillinus barberi* Jeannel WV, Kennison Mountain Trail | CWH_126 | **—** | **OR853135** | **—** | **OR839471** | **OR837818** | **—** | **—** | **—** |
| *Anillinus barberi* Jeannel WV, Kennison Mountain Trail | CWH_127 | **—** | **OR853129** | **—** | **OR839472** | **—** | **—** | **—** | **—** |
| *Anillinus barberi* Jeannel WV, Kennison Mountain Trail | CWH_128 | **—** | **OR853130** | **—** | **OR839473** | **—** | **—** | **—** | **—** |
| *Anillinus* cf*. barberi* Jeannel VA, Pandapas Pond | CWH_446 | **—** | **OR853180** | **OR839225** | **OR839678** | **—** | **—** | **—** | **—** |
| *Anillinus* cf*. barberi* Jeannel WV, Blue Bend Recreation Area | CWH_074 | **—** | **OR853138** | **OR839207** | **—** | **—** | **—** | **OR838153** | **—** |
| *Anillinus* cf*. barberi* Jeannel WV, Blue Bend Recreation Area | CWH_086 | **—** | **OR853136** | **—** | **—** | **—** | **—** | **—** | **—** |
| *Anillinus* cf*. barberi* Jeannel WV, Blue Bend Recreation Area | CWH_091 | **—** | **OR853137** | **—** | **OR839441** | **OR837807** | **—** | **—** | **—** |
| *Anillinus* cf*. barberi* Jeannel WV, Blue Bend Recreation Area | CWH_094 | **—** | **OR853139** | **—** | **OR839444** | **OR837808** | **OR837966** | **—** | **—** |
| *Anillinus* sp. "North Carolina, Boulder Field" | CWH_359 | **—** | **OR853177** | **—** | **OR839642** | **—** | **OR838058** | **—** | **—** |
| *Anillinus* sp. "North Carolina, Orange Co. sp. 1" | CWH_531 | **—** | **—** | **—** | **OR839731** | **—** | **—** | **—** | **—** |
| *Anillinus* sp. "Tennessee, Big Bald sp. 2" NC, Camp Creek Bald | CWH_526 | **—** | **OR853154** | **—** | **OR839727** | **—** | **—** | **—** | **—** |
| *Anillinus* sp. "Tennessee, Big Bald sp. 2" NC, Camp Creek Bald | CWH_527 | **—** | **OR853147** | **—** | **OR839728** | **—** | **—** | **OR838273** | **—** |
| *Anillinus* sp. "Tennessee, Big Bald sp. 2" NC, Camp Creek Bald | CWH_528 | **—** | **OR853155** | **—** | **OR839729** | **—** | **—** | **OR838274** | **—** |
| *Anillinus* sp. "Tennessee, Big Bald sp. 2" NC, Camp Creek Bald | CWH_529 | **—** | **OR853148** | **—** | **—** | **—** | **—** | **—** | **—** |
| *Anillinus* sp. "Tennessee, Big Bald sp. 2" TN, Big Bald | CWH_233 | **OR853111** | **OR853149** | **OR839214** | **OR839556** | **OR837879** | **OR838024** | **OR838212** | **—** |
| *Anillinus* sp. "Tennessee, Big Bald sp. 2" TN, Big Bald | CWH_360 | **—** | **OR853150** | **OR839215** | **—** | **—** | **—** | **—** | **—** |
| *Anillinus* sp. "Tennessee, Big Bald sp. 2" TN, Big Bald | CWH_361 | **—** | **OR853151** | **—** | **—** | **—** | **—** | **—** | **—** |
| *Anillinus* sp. "Tennessee, Big Bald sp. 2" TN, Big Bald | CWH_366 | **—** | **OR853152** | **—** | **—** | **—** | **—** | **—** | **—** |
| *Anillinus* sp. "Tennessee, Big Bald sp. 2" TN, Big Bald | CWH_367 | **—** | **OR853153** | **—** | **—** | **—** | **—** | **—** | **—** |
| *Anillinus* sp. "Virginia, Norton" | CWH_538 | **—** | **—** | **—** | **OR839734** | **—** | **—** | **OR838276** | **—** |
| *Anillinus* sp. "Virginia, Patrick Co." VA, Hwy 57 nr Route 8 | CWH_049 | **—** | **OR853143** | **OR839210** | **OR839402** | **OR837792** | **OR837954** | **OR838144** | **—** |
| *Anillinus* sp. "Virginia, Patrick Co." VA, Hwy 57 nr Route 8 | CWH_050 | **—** | **OR853141** | **—** | **OR839403** | **—** | **—** | **—** | **—** |
| *Anillinus* sp. "Virginia, Patrick Co." VA, Hwy 57 nr Route 8 | CWH_279 | **—** | **OR853142** | **—** | **OR839601** | **—** | **—** | **—** | **—** |
| *Anillinus* sp. "Virginia, Patrick Co." VA, Hwy 57 nr Route 8 | CWH_445 | **—** | **—** | **OR839209** | **OR839677** | **—** | **—** | **—** | **—** |
| *Anillinus* sp. "Virginia, Patrick Co." VA, Stuart | CWH_480 | **—** | **OR853144** | **OR839211** | **OR839700** | **—** | **—** | **—** | **—** |
| *Anillinus* sp. "Virginia, Whitetop Mountain" | MSC3241 | **—** | **OR853386** | **OR839358** | **OR839747** | **—** | **OR838099** | **—** | **—** |
| ***Anillinus* “*hirsutus*-group”** |  |  |  |  |  |  |  |  |  |
| *Anillinus* sp. "Alabama, Manitou Cave Preserve sp. 2" | CWH_377 | **—** | **OR853263** | **OR839283** | **OR839653** | **OR837912** | **OR838066** | **OR838246** | **—** |
| *Anillinus* sp. "Kentucky, Hestand sp. 4" | CWH_455 | **—** | **OR853362** | **OR839342** | **OR839682** | **—** | **OR838087** | **OR838256** | **—** |
| *Anillinus* sp. "Tennessee, Big Bald sp. 1" | CWH_216 | **—** | **OR853145** | **OR839212** | **OR839539** | **OR837865** | **OR838011** | **OR838199** | **—** |
| *Anillinus* sp. "Tennessee, Big Bald sp. 1" | CWH_369 | **—** | **OR853146** | **OR839213** | **OR839645** | **—** | **OR838060** | **—** | **—** |
| *Anillinus* sp. "Tennessee, Big Bald sp. 3" | CWH_363 | **—** | **OR853157** | **OR839216** | **OR839643** | **OR837908** | **OR838059** | **OR838243** | **—** |
| *Anillinus* sp. "Tennessee, Big Bald sp. 3" | CWH_364 | **—** | **OR853158** | **—** | **—** | **—** | **—** | **—** | **—** |
| *Anillinus* sp. "Tennessee, Big Bald sp. 3" | CWH_365 | **—** | **OR853156** | **—** | **OR839644** | **—** | **—** | **—** | **—** |
| *Anillinus* sp. "Virginia, Breaks sp. 1" | CWH_442 | **—** | **OR853175** | **OR839221** | **OR839675** | **—** | **OR838082** | **—** | **—** |
| ***Anillinus* “*erwini*-group”** |  |  |  |  |  |  |  |  |  |
| *Anillinus erwini* Sokolov & Carlton NC, Grandfather Mountain | CWH_478 | **—** | **OR853222** | **OR839259** | **OR839698** | **—** | **—** | **—** | **—** |
| *Anillinus erwini* Sokolov & Carlton NC, Grassy Ridge Bald | CWH_129 | **—** | **—** | **OR839260** | **OR839474** | **OR837819** | **OR837974** | **OR838161** | **—** |
| *Anillinus erwini* Sokolov & Carlton NC, Grassy Ridge Bald | CWH_130 | **—** | **OR853220** | **—** | **OR839475** | **OR837820** | **OR837975** | **OR838162** | **—** |
| *Anillinus erwini* Sokolov & Carlton NC, Grassy Ridge Bald | DNA5831 | ON557662 | ON557654 | ON554763 | ON554763 | **—** | ON72128 | ON721289 | **—** |
| *Anillinus erwini* Sokolov & Carlton VA, Mount Rogers | CWH_005 | **—** | **OR853223** | **OR839261** | **OR839374** | **OR837782** | **OR837947** | **OR838135** | **OR838113** |
| *Anillinus erwini* Sokolov & Carlton VA, Mount Rogers | CWH_071 | **—** | **OR853221** | **—** | **OR839423** | **OR837803** | **—** | **—** | **—** |
| ***Anillinus* “*dentatus*-group”** |  |  |  |  |  |  |  |  |  |
| *Anillinus dentatus* Harden & Caterino SC, Long Cane Creek | CWH_067 | **OR853105** | **OR853210** | **OR839250** | **OR839420** | **OR837801** | **OR837962** | **OR838150** | **OR838114** |
| *Anillinus dentatus* Harden & Caterino SC, Long Cane Creek | CWH_113 | **—** | **OR853209** | **OR839249** | **OR839458** | **OR837816** | **OR837972** | **—** | **—** |
| *Anillinus dentatus* Harden & Caterino SC, Long Cane Creek | CWH_417 | **—** | **—** | **OR839247** | **—** | **—** | **—** | **—** | **—** |
| *Anillinus dentatus* Harden & Caterino SC, Long Cane Creek **(HT)** | CWH_420 | **—** | **OR853208** | **OR839248** | **—** | **—** | **—** | **—** | **—** |
| *Anillinus dentatus* Harden & Caterino SC, Long Cane Creek | CWH_421 | **—** | **—** | **OR839246** | **—** | **—** | **—** | **—** | **—** |
| ***Anillinus* “*valentinei*-group”** |  |  |  |  |  |  |  |  |  |
| *Anillinus castaneus* Harden & Caterino SC, Chestnut Ridge Pres. | CWH_415 | **—** | **—** | **OR839224** | **—** | **—** | **—** | **—** | **—** |
| *Anillinus castaneus* Harden & Caterino SC, Chestnut Ridge Pres. | SSM101 | **—** | **OR853178** | **OR839223** | **OR839749** | **OR837941** | **OR838100** | **OR838278** | **—** |
| *Anillinus castaneus* Harden & Caterino SC, Chestnut Ridge Pres. | SSM98 | **—** | **OR853179** | **—** | **OR839821** | **—** | **OR838112** | **OR838296** | **—** |
| *Anillinus* cf*. chandleri* Sokolov SC, Long Cane Creek | CWH_151 | **—** | **OR853185** | **OR839230** | **OR839495** | **OR837835** | **OR837983** | **OR838169** | **—** |
| *Anillinus* cf*. chandleri* Sokolov SC, Long Cane Creek | CWH_152 | **—** | **OR853186** | **OR839231** | **OR839496** | **OR837836** | **OR837984** | **OR838170** | **—** |
| *Anillinus chandleri* Sokolov SC, Cedar Springs Rd | CWH_266 | **—** | **OR853187** | **OR839232** | **OR839589** | **—** | **OR838039** | **OR838229** | **—** |
| *Anillinus chandleri* Sokolov SC, Long Cane Creek | CWH_068 | **OR853106** | **OR853188** | **OR839233** | **OR839421** | **OR837802** | **OR837963** | **OR838151** | **OR838115** |
| *Anillinus cornelli* Sokolov & Carlton NC, Crowders Mountain | CWH_514 | **—** | **OR853205** | **OR839243** | **OR839720** | **OR837937** | **—** | **OR838267** | **—** |
| *Anillinus cornelli* Sokolov & Carlton NC, Crowders Mountain | CWH_515 | **—** | **OR853204** | **OR839242** | **OR839721** | **—** | **—** | **—** | **—** |
| *Anillinus gimmeli* Sokolov & Carlton TN, Turkeypen Ridge trail | CWH_262 | **—** | **OR853230** | **OR839263** | **OR839585** | **OR837893** | **OR838037** | **OR838227** | **—** |
| *Anillinus gimmeli* Sokolov & Carlton TN, White Oak Sink | CWH_439 | **—** | **—** | **—** | **OR839672** | **—** | **—** | **—** | **—** |
| *Anillinus gimmeli* Sokolov & Carlton TN, White Oak Sink | 75Gimm | — | — | FJ765218 | FJ765218 | — | — | — | — |
| *Anillinus gimmeli* Sokolov & Carlton TN, White Oak Sink | 76Gimm | — | — | FJ765219 | FJ765219 | — | — | — | — |
| *Anillinus kingi* Sokolov AL, Blount Mountain, Highway 24 | CWH_435 | **—** | **—** | **—** | **OR839668** | **—** | **—** | **—** | **—** |
| *Anillinus kingi* Sokolov AL, Blount Mountain, Highway 24 | CWH_436 | **—** | **—** | **—** | **OR839669** | **—** | **—** | **—** | **—** |
| *Anillinus kingi* Sokolov AL, Highland Lake | CWH_069 | **—** | **OR853235** | **—** | **—** | **—** | **—** | **—** | **—** |
| *Anillinus kingi* Sokolov AL, Highland Lake | CWH_200 | **—** | **OR853236** | **OR839268** | **OR839526** | **—** | **OR838001** | **OR838189** | **—** |
| *Anillinus kingi* Sokolov AL, Highland Lake | CWH_201 | **—** | **OR853234** | **OR839267** | **OR839527** | **OR837856** | **OR838002** | **OR838190** | **—** |
| *Anillinus murrayae* Sokolov & Carlton NC, Florence Nat. Pres. | CWH_393 | **—** | **—** | **—** | **OR839662** | **—** | **OR838071** | **—** | **—** |
| *Anillinus murrayae* Sokolov & Carlton NC, Melrose Falls | CWH_394 | **—** | **—** | **—** | **OR839663** | **—** | **—** | **—** | **—** |
| *Anillinus murrayae* Sokolov & Carlton NC, Melrose Falls | CWH_395 | **—** | **—** | **—** | **OR839664** | **—** | **—** | **—** | **—** |
| *Anillinus murrayae* Sokolov & Carlton SC, Ashmore Her. Pres | CWH_111 | **—** | **OR853300** | **OR839304** | **OR839456** | **OR837814** | **OR837970** | **OR838158** | **OR838118** |
| *Anillinus murrayae* Sokolov & Carlton SC, Ashmore Her. Pres | CWH_112 | **—** | **OR853295** | **—** | **OR839457** | **OR837815** | **OR837971** | **OR838159** | **—** |
| *Anillinus murrayae* Sokolov & Carlton SC, Ashmore Her. Pres | CWH_143 | **—** | **—** | **—** | **OR839487** | **OR837828** | **—** | **—** | **—** |
| *Anillinus murrayae* Sokolov & Carlton SC, Ashmore Her. Pres | CWH_144 | **—** | **—** | **—** | **OR839488** | **OR837829** | **—** | **—** | **—** |
| *Anillinus murrayae* Sokolov & Carlton SC, Ashmore Her. Pres | CWH_145 | **—** | **—** | **—** | **OR839489** | **OR837830** | **—** | **—** | **—** |
| *Anillinus murrayae* Sokolov & Carlton SC, Ashmore Her. Pres | CWH_146 | **—** | **—** | **—** | **OR839490** | **—** | **—** | **—** | **—** |
| *Anillinus murrayae* Sokolov & Carlton SC, Ashmore Her. Pres | CWH_147 | **—** | **—** | **OR839305** | **OR839491** | **OR837831** | **OR837982** | **—** | **—** |
| *Anillinus murrayae* Sokolov & Carlton SC, Ashmore Her. Pres | CWH_148 | **—** | **—** | **—** | **OR839492** | **OR837832** | **—** | **—** | **—** |
| *Anillinus murrayae* Sokolov & Carlton SC, Ashmore Her. Pres | CWH_149 | **—** | **—** | **—** | **OR839493** | **OR837833** | **—** | **—** | **—** |
| *Anillinus murrayae* Sokolov & Carlton SC, Ashmore Her. Pres | CWH_150 | **—** | **—** | **—** | **OR839494** | **OR837834** | **—** | **—** | **—** |
| *Anillinus murrayae* Sokolov & Carlton SC, Chimneytop Gap | CWH_490 | **—** | **OR853301** | **OR839306** | **OR839705** | **OR837930** | **—** | **OR838265** | **—** |
| *Anillinus murrayae* Sokolov & Carlton SC, Chimneytop Gap | CWH_508 | **—** | **—** | **—** | **OR839715** | **—** | **—** | **—** | **—** |
| *Anillinus murrayae* Sokolov & Carlton SC, Indian Camp Creek | SSM110 | **—** | **OR853296** | **OR839301** | **OR839750** | **—** | **OR838101** | **OR838279** | **—** |
| *Anillinus murrayae* Sokolov & Carlton SC, Indian Camp Creek | SSM251 | **—** | **OR853297** | **—** | **OR839798** | **—** | **—** | **OR838288** | **—** |
| *Anillinus murrayae* Sokolov & Carlton SC, Indian Camp Creek | SSM252 | **—** | **—** | **—** | **OR839799** | **—** | **—** | **—** | **—** |
| *Anillinus murrayae* Sokolov & Carlton SC, Pike Road | CWH_491 | **—** | **OR853302** | **OR839307** | **OR839706** | **OR837931** | **—** | **—** | **—** |
| *Anillinus murrayae* Sokolov & Carlton SC, Pike Road | CWH_509 | **—** | **—** | **—** | **OR839716** | **—** | **—** | **—** | **—** |
| *Anillinus murrayae* Sokolov & Carlton SC, State Botanical Gard. | CWH_497 | **—** | **—** | **—** | **OR839707** | **OR837932** | **—** | **—** | **—** |
| *Anillinus murrayae* Sokolov & Carlton SC, State Botanical Gard. | CWH_498 | **—** | **—** | **—** | **OR839708** | **OR837933** | **—** | **—** | **—** |
| *Anillinus murrayae* Sokolov & Carlton SC, Waldrop Stone | CWH_329 | **—** | **OR853303** | **OR839308** | **OR839622** | **OR837903** | **OR838051** | **OR838237** | **—** |
| *Anillinus murrayae* Sokolov & Carlton SC, Waldrop Stone | CWH_330 | **—** | **OR853304** | **—** | **OR839623** | **—** | **—** | **—** | **—** |
| *Anillinus murrayae* Sokolov & Carlton SC, Waldrop Stone | CWH_331 | **—** | **OR853298** | **—** | **OR839624** | **—** | **—** | **—** | **—** |
| *Anillinus murrayae* Sokolov & Carlton SC, Waldrop Stone | CWH_332 | **—** | **OR853299** | **—** | **OR839625** | **—** | **—** | **—** | **—** |
| *Anillinus murrayae* Sokolov & Carlton SC, Waldrop Stone | CWH_406 | **—** | **—** | **OR839302** | **—** | **—** | **—** | **—** | **—** |
| *Anillinus murrayae* Sokolov & Carlton SC, Waldrop Stone | CWH_407 | **—** | **—** | **OR839303** | **—** | **—** | **—** | **—** | **—** |
| *Anillinus murrayae* Sokolov & Carlton SC, Waldrop Stone | CWH_408 | **—** | **—** | **OR839309** | **—** | **—** | **—** | **—** | **—** |
| *Anillinus simplex* Harden & Caterino NC, Crowders Mountain | CWH_510 | **—** | **OR853340** | **OR839329** | **OR839717** | **OR837934** | **—** | **—** | **—** |
| *Anillinus simplex* Harden & Caterino NC, Crowders Mountain | CWH_512 | **—** | **OR853341** | **OR839330** | **OR839718** | **OR837935** | **—** | **—** | **—** |
| *Anillinus simplex* Harden & Caterino SC, Kings Mountain **(HT)** | CWH_247 | **—** | **OR853342** | **OR839331** | **OR839570** | **OR837888** | **OR838032** | **OR838222** | **—** |
| *Anillinus simplex* Harden & Caterino SC, Kings Mountain | CWH_248 | **—** | **—** | **—** | **OR839571** | **—** | **—** | **—** | **—** |
| *Anillinus simplex* Harden & Caterino SC, Kings Mountain | CWH_249 | **—** | **OR853339** | **OR839328** | **OR839572** | **OR837889** | **OR838033** | **OR838223** | **—** |
| *Anillinus smokiensis* Sokolov TN, Rich Mountain | CWH_253 | **—** | **OR853347** | **—** | **OR839576** | **—** | **—** | **OR838225** | **—** |
| *Anillinus smokiensis* Sokolov TN, Rich Mountain | CWH_254 | **—** | **—** | **—** | **OR839577** | **—** | **—** | **—** | **—** |
| *Anillinus smokiensis* Sokolov TN, Turkeypen Ridge | CWH_261 | **—** | **OR853346** | **—** | **OR839584** | **—** | **—** | **—** | **—** |
| *Anillinus smokiensis* Sokolov TN, Turkeypen Ridge | CWH_263 | **—** | **OR853348** | **OR839333** | **OR839586** | **OR837894** | **OR838038** | **OR838228** | **—** |
| *Anillinus smokiensis* Sokolov TN, Turkeypen Ridge | CWH_264 | **—** | **—** | **—** | **OR839587** | **—** | **—** | **—** | **—** |
| *Anillinus* sp. "Alabama, Krawczyk Caverns" | CWH_316 | **—** | **OR853238** | **OR839270** | **OR839614** | **OR837901** | **OR838047** | **—** | **—** |
| *Anillinus* sp. "Alabama, Little River" | CWH_378 | **—** | **OR853244** | **OR839274** | **OR839654** | **OR837913** | **OR838067** | **OR838247** | **—** |
| *Anillinus* sp. "Alabama, Magic City Cave" | CWH_314 | **—** | **OR853261** | **—** | **OR839612** | **OR837899** | **OR838045** | **OR838234** | **—** |
| *Anillinus* sp. "Alabama, Tidwell Hollow" | CWH_437 | **—** | **OR853363** | **OR839343** | **OR839670** | **OR837919** | **OR838080** | **—** | **—** |
| *Anillinus* sp. "Alabama, Tidwell Hollow" | CWH_438 | **—** | **—** | **—** | **OR839671** | **—** | **—** | **—** | **—** |
| *Anillinus* sp. "Kentucky, Laurel Co." | CWH_351 | **—** | **OR853357** | **OR839338** | **OR839638** | **OR837905** | **OR838055** | **OR838239** | **—** |
| *Anillinus* sp. "Kentucky, Laurel Co." | CWH_354 | **—** | **OR853356** | **OR839337** | **OR839639** | **OR837906** | **OR838056** | **OR838240** | **—** |
| *Anillinus* sp. “South Carolina, Chestnut Ridge” | CWH_401 | **—** | **OR853206** | **OR839244** | **OR839666** | **OR837917** | **OR838074** | **OR838251** | **—** |
| *Anillinus* sp. "South Carolina, Long Cane" | CWH_516 | **—** | **OR853246** | **OR839276** | **OR839722** | **OR837938** | **—** | **OR838268** | **—** |
| *Anillinus* sp. "South Carolina, Long Cane" | CWH_517 | **—** | **OR853245** | **OR839275** | **OR839723** | **OR837939** | **—** | **—** | **—** |
| *Anillinus* sp. "South Carolina, Waldrop Stone" SC, Chimneytop | CWH_489 | **—** | **OR853159** | **OR839217** | **OR839704** | **OR837929** | **OR838097** | **OR838264** | **—** |
| *Anillinus* sp. "South Carolina, Waldrop Stone" SC, Martin Cr. | CWH_440 | **—** | **OR853160** | **OR839218** | **OR839673** | **OR837920** | **OR838081** | **—** | **—** |
| *Anillinus* sp. "South Carolina, Waldrop Stone" SC, Martin Cr. | CWH_447 | **—** | **—** | **OR839219** | **—** | **—** | **—** | **—** | **—** |
| *Anillinus* sp. "South Carolina, Waldrop Stone" SC, Waldrop | CWH_433 | **—** | **—** | **OR839353** | **—** | **—** | **—** | **—** | **—** |
| *Anillinus* sp. "Tennessee, Ozone" | CWH_382 | **—** | **OR853243** | **OR839273** | **OR839658** | **OR837914** | **OR838069** | **—** | **—** |
| *Anillinus* sp. "Tennessee, Savage Gulf" | CWH_346 | **—** | **OR853315** | **OR839318** | **OR839634** | **OR837904** | **OR838054** | **OR838238** | **—** |
| *Anillinus* sp. "Tennessee, Savage Gulf" | CWH_347 | **—** | **—** | **—** | **OR839635** | **—** | **—** | **—** | **—** |
| *Anillinus* sp. "Tennessee, Savage Gulf" | CWH_348 | **—** | **—** | **—** | **OR839636** | **—** | **—** | **—** | **—** |
| *Anillinus* sp. "Virginia, Breaks sp. 2" | CWH_444 | **—** | **OR853176** | **OR839222** | **OR839676** | **OR837921** | **—** | **—** | **—** |
| ***Anillinus* “*albrittonorum*-group”** |  |  |  |  |  |  |  |  |  |
| *Anillinus albrittonorum* Sokolov & Schnepp FL, High Springs | CWH_206 | **—** | **—** | **OR839194** | **OR839530** | **OR837859** | **—** | **OR838193** | **—** |
| *Anillinus albrittonorum* Sokolov & Schnepp FL, High Springs | CWH_222 | **OR853109** | **OR853120** | **OR839195** | **OR839545** | **OR837868** | **OR838014** | **OR838202** | **OR838123** |
| *Anillinus* cf. *jancae* Harden & Caterino SC, Cedar Springs Rd | CWH_241 | **OR853112** | **OR853182** | **OR839227** | **OR839564** | **OR837883** | **OR838028** | **OR838217** | **OR838126** |
| *Anillinus jancae* Harden & Caterino larva SC, Long Cane Creek | CWH_416 | **—** | **—** | **OR839265** | **OR839667** | **OR837918** | **—** | **—** | **—** |
| *Anillinus jancae* Harden & Caterino SC, Long Cane Creek | CWH_204 | **—** | **OR853233** | **OR839266** | **—** | **—** | **OR838005** | **—** | **—** |
| ***Anillinus* “*pecki*-group”** |  |  |  |  |  |  |  |  |  |
| *Anillinus docwatsoni* Sokolov & Carlton NC, Bearwallow Mtn. | CWH_500 | **—** | **—** | **—** | **OR839709** | **—** | **—** | **—** | **—** |
| *Anillinus docwatsoni* Sokolov & Carlton NC, Broad River | CWH_486 | **—** | **OR853213** | **OR839253** | **OR839702** | **—** | **OR838096** | **—** | **—** |
| *Anillinus docwatsoni* Sokolov & Carlton NC, Chimney Rock | CWH_485 | **—** | **OR853214** | **OR839254** | **OR839701** | **OR837928** | **OR838095** | **OR838263** | **—** |
| *Anillinus docwatsoni* Sokolov & Carlton NC, Chimney Rock | CWH_487 | **—** | **OR853211** | **OR839251** | **OR839703** | **—** | **—** | **—** | **—** |
| *Anillinus docwatsoni* Sokolov & Carlton NC, Chimney Rock | CWH_488 | **—** | **OR853212** | **OR839252** | **—** | **—** | **—** | **—** | **—** |
| *Anillinus pecki* Giachino NC, Big Bald | CWH_237 | **—** | **—** | **OR839313** | **OR839560** | **OR837881** | **—** | **OR838214** | **—** |
| *Anillinus pecki* Giachino NC, Boulderfield Overlook | CWH_536 | **—** | **OR853309** | **—** | **OR839732** | **—** | **—** | **—** | **—** |
| *Anillinus pecki* Giachino NC, Boulderfield Overlook | CWH_537 | **—** | **OR853307** | **—** | **OR839733** | **—** | **—** | **—** | **—** |
| *Anillinus pecki* Giachino NC, Devils Gap | SSM135 | **—** | **OR853310** | **OR839314** | **OR839758** | **OR837942** | **OR838103** | **OR838281** | **—** |
| *Anillinus pecki* Giachino NC, Devils Gap | SSM138 | **—** | **—** | **—** | **OR839760** | **—** | **—** | **—** | **—** |
| *Anillinus pecki* Giachino NC, Devils Gap | SSM141 | **—** | **—** | **—** | **OR839762** | **—** | **—** | **—** | **—** |
| *Anillinus pecki* Giachino NC, Linville Gorge, Bynum Bluff | MSC2460 | **—** | **—** | **—** | **OR839741** | **—** | **—** | **—** | **—** |
| *Anillinus pecki* Giachino NC, Linville Gorge, Pine Gap | MSC2459 | **—** | **—** | **—** | **OR839740** | **—** | **—** | **—** | **—** |
| *Anillinus pecki* Giachino NC, Snooks Nose | SSM154 | **—** | **OR853308** | **—** | **OR839768** | **OR837943** | **OR838104** | **—** | **—** |
| *Anillinus pecki* Giachino NC, Woody Ridge | CWH_239 | **—** | **OR853311** | **OR839315** | **OR839562** | **OR837882** | **OR838026** | **OR838215** | **—** |
| *Anillinus pecki* Giachino NC, Woody Ridge | CWH_240 | **—** | **OR853312** | **—** | **OR839563** | **—** | **OR838027** | **OR838216** | **—** |
| *Anillinus pecki* Giachino TN, Big Bald | CWH_232 | **—** | **OR853313** | **OR839316** | **OR839555** | **OR837878** | **OR838023** | **OR838211** | **OR838125** |
| *Anillinus pecki* Giachino VA, Whitetop Mountain | CWH_453 | **—** | **OR853314** | **OR839317** | **—** | **—** | **OR838085** | **OR838254** | **—** |
| ***Anillinus* “*sinuaticollis*-group”** |  |  |  |  |  |  |  |  |  |
| *Anillinus* cf. *felicianus* Sokolov AL, Little River | CWH_379 | **—** | **OR853181** | **OR839226** | **OR839655** | **—** | **OR838068** | **OR838248** | **—** |
| *Anillinus* sp. “South Carolina, Coon Branch” SC, Coon Branch | CWH_102 | **—** | **OR853183** | **OR839228** | **OR839449** | **OR837811** | **OR837968** | **OR838156** | **—** |
| *Anillinus* sp. “South Carolina, Coon Branch” SC, Coon Branch | CWH_178 | **—** | **—** | **—** | **OR839507** | **OR837843** | **OR837990** | **OR838176** | **—** |
| *Anillinus choestoea* Harden & Caterino SC, Choestoea Park | CWH_310 | **—** | **OR853202** | **OR839240** | **OR839608** | **OR837897** | **OR838043** | **OR838232** | **—** |
| *Anillinus choestoea* Harden & Caterino SC, Choestoea Park **(HT**) | CWH_335 | **—** | **—** | **OR839239** | **OR839627** | **—** | **OR838052** | **—** | **—** |
| *Anillinus choestoea* Harden & Caterino SC, Choestoea Park | CWH_336 | **—** | **—** | **—** | **OR839628** | **—** | **—** | **—** | **—** |
| *Anillinus choestoea* Harden & Caterino SC, Choestoea Park | CWH_337 | **—** | **—** | **—** | **OR839629** | **—** | **—** | **—** | **—** |
| *Anillinus mica* Harden & Caterino SC, Central | MSC2458 | **—** | **—** | **—** | **OR839739** | **—** | **—** | **—** | **—** |
| *Anillinus mica* Harden & Caterino SC, Central | MSC2466 | **—** | **—** | **—** | **OR839745** | **—** | **—** | **—** | **—** |
| *Anillinus mica* Harden & Caterino SC, Issaqueena Lake | CWH_334 | **—** | **—** | **—** | **OR839626** | **—** | **—** | **—** | **—** |
| *Anillinus mica* Harden & Caterino SC, Issaqueena Lake Road | CWH_056 | **—** | **OR853282** | **—** | **OR839409** | **—** | **—** | **—** | **—** |
| *Anillinus mica* Harden & Caterino SC, Issaqueena Lake Road | CWH_057 | **—** | **OR853280** | **—** | **OR839410** | **—** | **—** | **—** | **—** |
| *Anillinus mica* Harden & Caterino SC, Issaqueena Lake Road | CWH_060 | **—** | **OR853281** | **—** | **OR839413** | **—** | **—** | **—** | **—** |
| *Anillinus mica* Harden & Caterino SC, Issaqueena Lake Road | CWH_333 | **—** | **OR853283** | **—** | **—** | **—** | **—** | **—** | **OR838130** |
| *Anillinus mica* Harden & Caterino SC, Nine Times Preserve | CWH_039 | **—** | **OR853284** | **OR839291** | **OR839392** | **OR837789** | **OR837951** | **OR838142** | **—** |
| *Anillinus mica* Harden & Caterino SC, Todd Creek Falls | CWH_230 | **OR853110** | **OR853285** | **OR839292** | **OR839553** | **OR837876** | **OR838021** | **OR838209** | **OR838124** |
| *Anillinus mica* Harden & Caterino SC, Todd Creek Falls | CWH_231 | **—** | **OR853286** | **—** | **OR839554** | **OR837877** | **OR838022** | **OR838210** | **—** |
| *Anillinus mica* Harden & Caterino SC, Waldrop Creek | CWH_409 | **—** | **OR853288** | **OR839294** | **—** | **—** | **OR838079** | **—** | **—** |
| *Anillinus mica* Harden & Caterino SC, Waldrop Creek | CWH_410 | **—** | **OR853289** | **—** | **—** | **—** | **—** | **—** | **—** |
| *Anillinus mica* Harden & Caterino SC, Waldrop Creek | CWH_412 | **—** | **—** | **OR839288** | **—** | **—** | **—** | **—** | **—** |
| *Anillinus mica* Harden & Caterino SC, Waldrop Creek | CWH_413 | **—** | **—** | **OR839289** | **—** | **—** | **—** | **—** | **—** |
| *Anillinus mica* Harden & Caterino SC, Waldrop Creek | CWH_414 | **—** | **—** | **OR839290** | **—** | **—** | **—** | **—** | **—** |
| *Anillinus mica* Harden & Caterino SC, Waldrop Stone Falls **(HT)** | CWH_403 | **—** | **OR853287** | **OR839293** | **—** | **—** | **OR838076** | **—** | **—** |
| *Anillinus micamicus* Harden & Caterino SC, Issaqueena Lake Rd. | CWH_061 | **—** | **OR853293** | **OR839298** | **OR839414** | **OR837797** | **OR837958** | **OR838148** | **—** |
| *Anillinus micamicus* Harden & Caterino SC, Waldrop Stone Falls **(HT**) | CWH_402 | **—** | **OR853291** | **OR839296** | **—** | **—** | **OR838075** | **—** | **—** |
| *Anillinus micamicus* Harden & Caterino SC, Waldrop Stone Falls | CWH_404 | **—** | **OR853292** | **OR839297** | **—** | **—** | **OR838077** | **—** | **—** |
| *Anillinus micamicus* Harden & Caterino SC, Waldrop Stone Falls | CWH_405 | **—** | **OR853290** | **OR839295** | **—** | **—** | **OR838078** | **OR838252** | **—** |
| *Anillinus seneca* Harden & Caterino larva SC, Martin Creek | CWH_450 | **—** | **OR853242** | **—** | **—** | **—** | **—** | **—** | **—** |
| *Anillinus seneca* Harden & Caterino SC, Big Oaks | CWH_161 | **—** | **OR853317** | **OR839320** | **OR839500** | **OR837838** | **OR837986** | **OR838172** | **—** |
| *Anillinus seneca* Harden & Caterino SC, Big Oaks | CWH_162 | **—** | **—** | **—** | **OR839501** | **OR837839** | **—** | **—** | **—** |
| *Anillinus seneca* Harden & Caterino SC, Big Oaks | CWH_163 | **—** | **OR853316** | **OR839319** | **OR839502** | **OR837840** | **OR837987** | **OR838173** | **—** |
| *Anillinus seneca* Harden & Caterino SC, Big Oaks | CWH_164 | **—** | **—** | **—** | **OR839503** | **—** | **—** | **—** | **—** |
| *Anillinus seneca* Harden & Caterino SC, Martin Creek Landing | CWH_062 | **—** | **OR853318** | **OR839321** | **OR839415** | **OR837798** | **OR837959** | **—** | **—** |
| *Anillinus seneca* Harden & Caterino SC, Martin Creek Landing | CWH_063 | **—** | **OR853319** | **OR839322** | **OR839416** | **OR837799** | **OR837960** | **—** | **—** |
| *Anillinus seneca* Harden & Caterino SC, Martin Creek Landing | CWH_064 | **—** | **OR853320** | **—** | **OR839417** | **—** | **—** | **—** | **—** |
| *Anillinus seneca* Harden & Caterino SC, Martin Creek Landing | CWH_065 | **—** | **OR853321** | **OR839323** | **OR839418** | **OR837800** | **OR837961** | **OR838149** | **—** |
| *Anillinus seneca* Harden & Caterino SC, Martin Creek Landing | CWH_066 | **—** | **—** | **—** | **OR839419** | **—** | **—** | **—** | **—** |
| *Anillinus seneca* Harden & Caterino SC, Martin Creek Landing | CWH_070 | **—** | **—** | **—** | **OR839422** | **—** | **—** | **—** | **—** |
| *Anillinus seneca* Harden & Caterino SC, Martin Creek Landing | CWH_114 | **—** | **OR853325** | **—** | **OR839459** | **—** | **—** | **—** | **—** |
| *Anillinus seneca* Harden & Caterino SC, Martin Creek Landing | CWH_115 | **—** | **—** | **—** | **OR839460** | **—** | **—** | **—** | **—** |
| *Anillinus seneca* Harden & Caterino SC, Martin Creek Landing | CWH_116 | **—** | **—** | **—** | **OR839461** | **—** | **—** | **—** | **—** |
| *Anillinus seneca* Harden & Caterino SC, Martin Creek Landing | CWH_117 | **—** | **—** | **—** | **OR839462** | **—** | **—** | **—** | **—** |
| *Anillinus seneca* Harden & Caterino SC, Martin Creek Landing | CWH_118 | **—** | **—** | **—** | **OR839463** | **—** | **—** | **—** | **—** |
| *Anillinus seneca* Harden & Caterino SC, Martin Creek Landing | CWH_119 | **—** | **OR853322** | **—** | **OR839464** | **—** | **—** | **—** | **—** |
| *Anillinus seneca* Harden & Caterino SC, Martin Creek Landing | CWH_120 | **—** | **—** | **—** | **OR839465** | **—** | **—** | **—** | **—** |
| *Anillinus seneca* Harden & Caterino SC, Martin Creek Landing **(HT)** | CWH_121 | **—** | **OR853323** | **—** | **OR839466** | **—** | **—** | **—** | **—** |
| *Anillinus seneca* Harden & Caterino SC, Martin Creek Landing | CWH_122 | **—** | **—** | **—** | **OR839467** | **—** | **—** | **—** | **—** |
| *Anillinus seneca* Harden & Caterino SC, Martin Creek Landing | CWH_123 | **—** | **—** | **—** | **OR839468** | **—** | **—** | **—** | **—** |
| *Anillinus seneca* Harden & Caterino SC, Martin Creek Landing | CWH_124 | **—** | **OR853324** | **—** | **OR839469** | **—** | **—** | **—** | **—** |
| *Anillinus seneca* Harden & Caterino SC, South Cove Park | CWH_338 | **—** | **OR853327** | **—** | **OR839630** | **—** | **OR838053** | **—** | **—** |
| *Anillinus seneca* Harden & Caterino SC, South Cove Park | CWH_340 | **—** | **OR853328** | **—** | **OR839631** | **—** | **—** | **—** | **—** |
| *Anillinus seneca* Harden & Caterino SC, South Cove Park | CWH_342 | **—** | **—** | **—** | **OR839632** | **—** | **—** | **—** | **—** |
| *Anillinus seneca* Harden & Caterino SC, South Cove Park | CWH_343 | **—** | **—** | **—** | **OR839633** | **—** | **—** | **—** | **—** |
| *Anillinus seneca* Harden & Caterino SC, River Forks Rec. Area | CWH_327 | **—** | **OR853326** | **—** | **OR839621** | **—** | **OR838050** | **—** | **—** |
| *Anillinus* sp. "Kentucky, Hestand sp. 3" | CWH_356 | **—** | **OR853361** | **OR839341** | **OR839640** | **—** | **OR838057** | **OR838241** | **—** |
| *Anillinus* sp. "Tennessee, Webb Cave" | CWH_313 | **—** | **OR853385** | **OR839357** | **OR839611** | **OR837898** | **OR838044** | **OR838233** | **—** |
| ***Anillinus* “*elongatus*-group”** |  |  |  |  |  |  |  |  |  |
| *Anillinus arenicollis* Harden & Caterino SC, Sandhills NWR **(HT)** | CWH_397 | **—** | **OR853123** | **OR839200** | **—** | **—** | **OR838072** | **—** | **—** |
| *Anillinus arenicollis* Harden & Caterino SC, Sandhills NWR | CWH_398 | **—** | **—** | **OR839197** | **—** | **—** | **—** | **—** | **—** |
| *Anillinus arenicollis* Harden & Caterino SC, Sandhills NWR | CWH_399 | **—** | **—** | **OR839198** | **—** | **—** | **—** | **—** | **—** |
| *Anillinus arenicollis* Harden & Caterino SC, Sandhills NWR | MSC2481 | **—** | **OR853122** | **OR839199** | **OR839746** | **OR837940** | **OR838098** | **OR838277** | **—** |
| *Anillinus* cf*. montrex* Harden & Caterino NC, Crowders Mtn. | CWH_513 | **—** | **OR853184** | **OR839229** | **OR839719** | **OR837936** | **—** | **—** | **—** |
| *Anillinus elongatus* Jeannel NC, Duke Forest | CWH_464 | **—** | **OR853218** | **OR839257** | **OR839688** | **—** | **—** | **—** | **—** |
| *Anillinus elongatus* Jeannel NC, Duke Forest | CWH_465 | **—** | **OR853219** | **OR839258** | **OR839689** | **—** | **—** | **—** | **—** |
| *Anillinus elongatus* Jeannel NC, Duke Forest | CWH_467 | **—** | **OR853216** | **OR839255** | **OR839690** | **—** | **OR838091** | **OR838260** | **—** |
| *Anillinus elongatus* Jeannel NC, Duke Forest | CWH_468 | **—** | **OR853217** | **OR839256** | **OR839691** | **—** | **—** | **—** | **—** |
| *Anillinus montrex* Harden & Caterino SC, Kings Mountain **(HT)** | CWH_242 | **OR853113** | **OR853294** | **OR839300** | **OR839565** | **OR837884** | **OR838029** | **OR838218** | **OR838127** |
| *Anillinus montrex* Harden & Caterino SC, Kings Mountain | CWH_243 | **—** | **—** | **—** | **OR839566** | **—** | **—** | **—** | **—** |
| *Anillinus montrex* Harden & Caterino SC, Kings Mountain | CWH_244 | **—** | **—** | **OR839299** | **OR839567** | **OR837885** | **OR838030** | **OR838219** | **—** |
| *Anillinus pittsylvanicus* Harden & Caterino VA, Anglers Park | CWH_059 | **—** | **OR853207** | **OR839245** | **OR839412** | **OR837796** | **OR837957** | **OR838147** | **—** |
| *Anillinus* sp. "North Carolina, Orange Co. Sp. 2" | CWH_520 | **—** | **OR853215** | **—** | **OR839725** | **—** | **—** | **OR838270** | **—** |
| *Anillinus uwharrie* Harden & Caterino NC, Uwharrie Nat. Forest **(HT)** | CWH_473 | **—** | **OR853370** | **OR839348** | **OR839693** | **—** | **OR838093** | **OR838261** | **—** |
| *Anillinus uwharrie* Harden & Caterino NC, Uwharrie Nat. Forest | CWH_474 | **—** | **OR853371** | **OR839349** | **OR839694** | **—** | **—** | **—** | **—** |
| *Anillinus uwharrie* Harden & Caterino NC, Uwharrie Nat. Forest | CWH_475 | **—** | **—** | **OR839346** | **OR839695** | **—** | **—** | **—** | **—** |
| *Anillinus uwharrie* Harden & Caterino NC, Uwharrie Nat. Forest | CWH_476 | **—** | **OR853369** | **OR839347** | **OR839696** | **—** | **—** | **—** | **—** |
| ***Anillinus* “Kings Saltpeter Cave”** |  |  |  |  |  |  |  |  |  |
| *Anillinus* sp. "Tennessee, Kings Saltpeter Cave" | CWH_315 | **OR853114** | **OR853237** | **OR839269** | **OR839613** | **OR837900** | **OR838046** | **OR838235** | **OR838129** |
| ***Anillinus* “*langdoni*-group”** |  |  |  |  |  |  |  |  |  |
| *Anillinus balli* Sokolov & Carlton KY, Cumberland River | CWH_459 | **—** | **OR853126** | **OR839203** | **OR839683** | **—** | **—** | **—** | **—** |
| *Anillinus balli* Sokolov & Carlton KY, Cumberland River | CWH_460 | **—** | **OR853127** | **OR839204** | **OR839684** | **OR837925** | **OR838088** | **OR838257** | **—** |
| *Anillinus balli* K Sokolov & Carlton Y, Cumberland River | CWH_461 | **—** | **OR853124** | **OR839201** | **OR839685** | **—** | **OR838089** | **OR838258** | **—** |
| *Anillinus balli* Sokolov & Carlton KY, Cumberland River | CWH_462 | **—** | **OR853125** | **OR839202** | **OR839686** | **—** | **—** | **—** | **—** |
| *Anillinus* cf. *nantahala* Dajoz GA, Little Bald | CWH_186 | **—** | **OR853334** | **—** | **OR839514** | **OR837849** | **OR837995** | **OR838181** | **—** |
| *Anillinus* cf. *nantahala* Dajoz GA, Little Bald | CWH_188 | **—** | **OR853329** | **—** | **OR839516** | **—** | **—** | **OR838183** | **—** |
| *Anillinus* cf. *nantahala* Dajoz GA, Little Bald | CWH_194 | **—** | **—** | **—** | **OR839522** | **—** | **—** | **—** | **—** |
| *Anillinus* cf. *nantahala* Dajoz GA, Little Bald | CWH_195 | **—** | **—** | **—** | **OR839523** | **—** | **—** | **—** | **—** |
| *Anillinus* cf. *nantahala* Dajoz GA, Panther Creek | DNA0690 | — | GU556075 | — | **OR830242** | MK118201 | MK112078 | GU556025 | — |
| *Anillinus* cf. *nantahala* Dajoz NC, Hickory Branch | SSM63 | **—** | **OR853335** | **OR839325** | **OR839812** | **—** | **OR838110** | **OR838292** | **—** |
| *Anillinus* cf. *nantahala* Dajoz NC, Hickory Branch | SSM64 | **—** | **OR853336** | **—** | **OR839813** | **—** | **—** | **OR838293** | **—** |
| *Anillinus* cf. *nantahala* Dajoz NC, Huckleberry Knob | CWH_283 | **—** | **—** | **—** | **OR839604** | **—** | **—** | **—** | **—** |
| *Anillinus* cf. *nantahala* Dajoz NC, Joyce Kilmer | SSM037 | **—** | **OR853330** | **—** | **OR839748** | **—** | **—** | **—** | **—** |
| *Anillinus* cf. *nantahala* Dajoz NC, Riley Knob | CWH_181 | **—** | **OR853331** | **OR839324** | **OR839510** | **OR837846** | **OR837993** | **OR838179** | **—** |
| *Anillinus* cf. *nantahala* Dajoz NC, Shooting Creek Bald | CWH_179 | **—** | **OR853337** | **OR839326** | **OR839508** | **OR837844** | **OR837991** | **OR838177** | **—** |
| *Anillinus* cf. *nantahala* Dajoz NC, Wayah Road | CWH_054 | **—** | **OR853333** | **—** | **OR839407** | **OR837795** | **OR837956** | **OR838146** | **—** |
| *Anillinus* cf. *nantahala* Dajoz NC, Wayah Road | CWH_139 | **—** | **OR853332** | **—** | **OR839483** | **OR837826** | **OR837980** | **OR838167** | **—** |
| *Anillinus* cf. *nantahala* Dajoz NC, Wayah Road | CWH_140 | **—** | **OR853338** | **OR839327** | **OR839484** | **OR837827** | **OR837981** | **OR838168** | **OR838120** |
| *Anillinus* cf. *nantahala* Dajoz NC, Wayah Road | CWH_141 | **—** | **—** | **—** | **OR839485** | **—** | **—** | **—** | **—** |
| *Anillinus* cf. *nantahala* Dajoz NC, Wayah Road | CWH_142 | **—** | **—** | **—** | **OR839486** | **—** | **—** | **—** | **—** |
| *Anillinus* cf. *nantahala* Dajoz NC, Wayah Road | CWH_167 | **—** | **—** | **—** | **OR839506** | **—** | **—** | **—** | **—** |
| *Anillinus* cf. *nantahala* Dajoz TN, Miller Cove | CWH_349 | **—** | **—** | **—** | **OR839637** | **—** | **—** | **—** | **—** |
| *Anillinus cieglerae* Sokolov & Carlton TN, Bull Cave | LSAM107950 | — | — | — | EF460865 | — | — | — | — |
| *Anillinus cieglerae* Sokolov & Carlton TN, Bull Cave | LSAM107951 | — | — | — | EF460866 | — | — | — | — |
| *Anillinus cieglerae* Sokolov & Carlton TN, Turkeypen Ridge | CWH_257 | **—** | **OR853203** | **OR839241** | **OR839580** | **OR837892** | **OR838036** | **OR838226** | **—** |
| *Anillinus cieglerae* Sokolov & Carlton TN, Turkeypen Ridge | CWH_258 | **—** | **—** | **—** | **OR839581** | **—** | **—** | **—** | **—** |
| *Anillinus cieglerae* Sokolov & Carlton TN, Turkeypen Ridge | CWH_259 | **—** | **—** | **—** | **OR839582** | **—** | **—** | **—** | **—** |
| *Anillinus daggyi* Sokolov & Carlton NC, South Mountains | LSAM107953 | — | — | — | EF460868 | — | — | — | — |
| *Anillinus langdoni* Sokolov & Carlton TN, Albright Grove | CWH_325 | **—** | **OR853239** | **OR839271** | **OR839619** | **—** | **OR838049** | **OR838236** | **—** |
| *Anillinus langdoni* Sokolov & Carlton TN, Albright Grove | CWH_326 | **—** | **—** | **—** | **OR839620** | **—** | **—** | **—** | **—** |
| *Anillinus langdoni* Sokolov & Carlton TN, The Sinks | LSAM107956 | — | — | — | EF460872 | — | — | — | — |
| *Anillinus langdoni* Sokolov & Carlton TN, The Sinks | LSAM107957 | — | — | — | EF460871 | — | — | — | — |
| *Anillinus langdoni* Sokolov & Carlton TN, The Sinks | LSAM107958 | — | — | — | EF460873 | — | — | — | — |
| *Anillinus pusillus* Sokolov & Carlton TN, Forge Creek | LSAM107954 | — | — | — | EF460869 | — | — | — | — |
| *Anillinus virginiae* Jeannel VA, Buffalo Gap | CWH_479 | **—** | **OR853378** | **OR839350** | **OR839699** | **OR837927** | **OR838094** | **—** | **—** |
| *Anillinus virginiae* Jeannel VA, Warm Springs Mountain | CWH_469 | **—** | **OR853379** | **OR839351** | **OR839692** | **OR837926** | **OR838092** | **—** | **—** |
| *Anillinus virginiae* Jeannel WV, Blue Bend | CWH_075 | **—** | **OR853382** | **—** | **OR839426** | **—** | **—** | **—** | **—** |
| *Anillinus virginiae* Jeannel WV, Blue Bend | CWH_076 | **—** | **—** | **—** | **OR839427** | **—** | **—** | **—** | **—** |
| *Anillinus virginiae* Jeannel WV, Blue Bend | CWH_077 | **—** | **—** | **—** | **OR839428** | **—** | **—** | **—** | **—** |
| *Anillinus virginiae* Jeannel WV, Blue Bend | CWH_078 | **—** | **—** | **—** | **OR839429** | **—** | **—** | **—** | **—** |
| *Anillinus virginiae* Jeannel WV, Blue Bend | CWH_079 | **—** | **—** | **—** | **OR839430** | **—** | **—** | **—** | **—** |
| *Anillinus virginiae* Jeannel WV, Blue Bend | CWH_080 | **—** | **—** | **—** | **OR839431** | **—** | **—** | **—** | **—** |
| *Anillinus virginiae* Jeannel WV, Blue Bend | CWH_081 | **—** | **—** | **—** | **OR839432** | **—** | **—** | **—** | **—** |
| *Anillinus virginiae* Jeannel WV, Blue Bend | CWH_082 | **—** | **—** | **—** | **OR839433** | **—** | **—** | **—** | **—** |
| *Anillinus virginiae* Jeannel WV, Blue Bend | CWH_083 | **—** | **—** | **—** | **OR839434** | **—** | **—** | **—** | **—** |
| *Anillinus virginiae* Jeannel WV, Blue Bend | CWH_084 | **—** | **—** | **—** | **OR839435** | **—** | **—** | **—** | **—** |
| *Anillinus virginiae* Jeannel WV, Blue Bend | CWH_085 | **—** | **OR853372** | **—** | **OR839436** | **—** | **—** | **—** | **—** |
| *Anillinus virginiae* Jeannel WV, Blue Bend | CWH_087 | **—** | **OR853373** | **—** | **OR839437** | **—** | **—** | **—** | **—** |
| *Anillinus virginiae* Jeannel WV, Blue Bend | CWH_088 | **—** | **—** | **—** | **OR839438** | **OR837806** | **—** | **—** | **—** |
| *Anillinus virginiae* Jeannel WV, Blue Bend | CWH_089 | **—** | **OR853374** | **—** | **OR839439** | **—** | **—** | **—** | **—** |
| *Anillinus virginiae* Jeannel WV, Blue Bend | CWH_090 | **—** | **OR853375** | **—** | **OR839440** | **—** | **—** | **—** | **—** |
| *Anillinus virginiae* Jeannel WV, Blue Bend | CWH_092 | **—** | **OR853376** | **—** | **OR839442** | **—** | **—** | **—** | **—** |
| *Anillinus virginiae* Jeannel WV, Blue Bend | CWH_093 | **—** | **OR853377** | **—** | **OR839443** | **—** | **—** | **—** | **—** |
| *Anillinus virginiae* Jeannel WV, Blue Bend | CWH_072 | **—** | **OR853380** | **OR839352** | **OR839424** | **OR837804** | **OR837964** | **OR838152** | **—** |
| *Anillinus virginiae* Jeannel WV, Blue Bend | CWH_073 | **—** | **OR853381** | **—** | **OR839425** | **OR837805** | **OR837965** | **—** | **—** |
| *Anillinus virginiae* Jeannel WV, Watoga State Park | IBE AI1146 | KU978703 | KU978752 | **—** | KU976327 | **—** | **—** | **—** | **—** |
| *Anillinus* sp. "Georgia, Barnes Creek sp. 1" | CWH_226 | **—** | **OR853364** | **OR839344** | **OR839549** | **OR837872** | **OR838017** | **OR838205** | **—** |
| *Anillinus* sp. "Georgia, Barnes Creek sp. 1" | CWH_503 | **—** | **—** | **—** | **OR839710** | **—** | **—** | **—** | **—** |
| *Anillinus* sp. "Georgia, Brasstown Bald sp. 1" | CWH_192 | **—** | **OR853248** | **OR839277** | **OR839520** | **OR837853** | **OR837999** | **OR838186** | **—** |
| *Anillinus* sp. "Georgia, Brasstown Bald sp. 1" | CWH_193 | **—** | **OR853249** | **—** | **OR839521** | **OR837854** | **—** | **OR838187** | **—** |
| *Anillinus* sp. "Georgia, Brasstown Bald sp. 1" | CWH_196 | **—** | **—** | **—** | **OR839524** | **OR837855** | **OR838000** | **OR838188** | **—** |
| *Anillinus* sp. "Georgia, Brasstown Bald sp. 1" | CWH_197 | **—** | **—** | **—** | **OR839525** | **—** | **—** | **—** | **—** |
| *Anillinus* sp. "North Carolina, Joyce Kilmer" | SSM41 | **—** | **OR853247** | **—** | **OR839806** | **—** | **—** | **OR838289** | **—** |
| *Anillinus* sp. "North Carolina, Joyce Kilmer" NC, Joyce Kilmer | SSM42 | **—** | **OR853250** | **OR839278** | **OR839811** | **—** | **OR838109** | **OR838291** | **—** |
| *Anillinus* sp. "North Carolina, Joyce Kilmer" NC, Stratton Ridge | CWH_276 | **—** | **OR853251** | **—** | **OR839598** | **—** | **—** | **—** | **—** |
| *Anillinus* sp. "North Carolina, Joyce Kilmer" NC, Stratton Ridge | CWH_277 | **—** | **OR853252** | **—** | **OR839599** | **—** | **—** | **—** | **—** |
| *Anillinus* sp. "Tennessee, Indian Boundary" TN, Indian Bound. | CWH_217 | **—** | **—** | **—** | **OR839540** | **—** | **—** | **—** | **—** |
| *Anillinus* sp. "Tennessee, Indian Boundary" TN, Indian Bound. | CWH_218 | **—** | **OR853241** | **OR839272** | **OR839541** | **OR837866** | **OR838012** | **OR838200** | **—** |
| *Anillinus* sp. "Tennessee, Indian Boundary" TN, Indian Bound. | CWH_219 | **—** | **OR853240** | **—** | **OR839542** | **OR837867** | **OR838013** | **OR838201** | **—** |
| *Anillinus* sp. "Tennessee, Indian Boundary" TN, Indian Bound. | CWH_220 | **—** | **—** | **—** | **OR839543** | **—** | **—** | **—** | **—** |
| *Anillinus* sp. "Tennessee, Indian Boundary" TN, Tellico River | CWH_190 | **—** | **OR853264** | **OR839284** | **OR839518** | **OR837851** | **OR837997** | **OR838184** | **—** |
| ***Anillinus* “*loweae*-group”** |  |  |  |  |  |  |  |  |  |
| *Anillinus* cf*. fortis* (Horn) NC, Big Bald | CWH_234 | **—** | **OR853227** | **OR839262** | **OR839557** | **OR837880** | **OR838025** | **OR838213** | **—** |
| *Anillinus* cf*. fortis* (Horn) NC, Big Bald | CWH_235 | **—** | **OR853228** | **—** | **OR839558** | **—** | **—** | **—** | **—** |
| *Anillinus* cf*. fortis* (Horn) NC, Big Bald | CWH_236 | **—** | **—** | **—** | **OR839559** | **—** | **—** | **—** | **—** |
| *Anillinus* cf*. fortis* (Horn) NC, Big Bald | CWH_238 | **—** | **—** | **—** | **OR839561** | **—** | **—** | **—** | **—** |
| *Anillinus* cf*. fortis* (Horn) NC, Big Butt trail | SSM129 | **—** | **—** | **—** | **OR839753** | **—** | **—** | **—** | **—** |
| *Anillinus* cf*. fortis* (Horn) NC, Big Butt trail | SSM130 | **—** | **—** | **—** | **OR839754** | **—** | **—** | **—** | **—** |
| *Anillinus* cf*. fortis* (Horn) NC, Big Butt trail | SSM131 | **—** | **—** | **—** | **OR839755** | **—** | **—** | **—** | **—** |
| *Anillinus* cf*. fortis* (Horn) NC, Big Butt trail | SSM132 | **—** | **—** | **—** | **OR839756** | **—** | **—** | **—** | **—** |
| *Anillinus* cf*. fortis* (Horn) NC, Big Butt trail | SSM134 | **—** | **—** | **—** | **OR839757** | **—** | **—** | **—** | **—** |
| *Anillinus* cf*. fortis* (Horn) NC, Devils Gap | SSM136 | **—** | **OR853225** | **—** | **OR839759** | **—** | **—** | **—** | **—** |
| *Anillinus* cf*. fortis* (Horn) NC, Devils Gap | SSM139 | **—** | **—** | **—** | **OR839761** | **—** | **—** | **—** | **—** |
| *Anillinus* cf*. fortis* (Horn) NC, Devils Gap | SSM142 | **—** | **—** | **—** | **OR839763** | **—** | **—** | **—** | **—** |
| *Anillinus* cf*. fortis* (Horn) NC, Devils Gap | SSM143 | **—** | **—** | **—** | **OR839764** | **—** | **—** | **—** | **—** |
| *Anillinus* cf*. fortis* (Horn) NC, Devils Gap | SSM144 | **—** | **—** | **—** | **OR839765** | **—** | **—** | **—** | **—** |
| *Anillinus* cf*. fortis* (Horn) NC, Florence Nature Preserve | CWH_392 | **—** | **OR853226** | **—** | **OR839661** | **—** | **—** | **—** | **—** |
| *Anillinus* cf*. fortis* (Horn) NC, Camp Creek Bald | CWH_530 | **—** | **OR853224** | **—** | **OR839730** | **—** | **—** | **OR838275** | **—** |
| *Anillinus* cf*. fortis* (Horn) NC, Mackey Mountain | SSM279 | **—** | **—** | **—** | **OR839803** | **—** | **—** | **—** | **—** |
| *Anillinus* cf*. fortis* (Horn) NC, Mackey Mountain | SSM281 | **—** | **—** | **—** | **OR839804** | **—** | **—** | **—** | **—** |
| *Anillinus* cf*. fortis* (Horn) NC, Mackey Mountain | SSM282 | **—** | **—** | **—** | **OR839805** | **—** | **—** | **—** | **—** |
| *Anillinus* cf*. fortis* (Horn) NC, Snooks Nose | SSM150 | **—** | **—** | **—** | **OR839766** | **—** | **—** | **—** | **—** |
| *Anillinus* cf*. fortis* (Horn) NC, Snooks Nose | SSM151 | **—** | **OR853229** | **—** | **OR839767** | **—** | **—** | **OR838282** | **—** |
| *Anillinus* cf*. fortis* (Horn) NC, Snooks Nose | SSM152 | **—** | **—** | **—** | **OR839822** | **—** | **—** | **—** | **—** |
| *Anillinus* cf*. fortis* (Horn) NC, Snooks Nose | SSM153 | **—** | **—** | **—** | **OR839823** | **—** | **—** | **—** | **—** |
| *Anillinus* cf*. fortis* (Horn) NC, Snooks Nose | SSM155 | **—** | **—** | **—** | **OR839769** | **—** | **—** | **—** | **—** |
| *Anillinus* cf*. fortis* (Horn) NC, Snooks Nose | SSM156 | **—** | **—** | **—** | **OR839770** | **—** | **—** | **—** | **—** |
| *Anillinus cherokee* Sokolov & Carlton GA, Rabun Bald | CWH_026 | **—** | **OR853193** | **OR839235** | **OR839389** | **OR837787** | **OR837950** | **OR838140** | **—** |
| *Anillinus cherokee* Sokolov & Carlton GA, Rabun Bald | CWH_028 | **—** | **OR853189** | **—** | **OR839391** | **—** | **—** | **—** | **—** |
| *Anillinus cherokee* Sokolov & Carlton GA, Rabun Bald | CWH_096 | **—** | **OR853190** | **—** | **OR839446** | **OR837809** | **—** | **OR838154** | **—** |
| *Anillinus cherokee* Sokolov & Carlton NC, Cherohala Skyway | 77Cher | — | — | FJ765223 | FJ765223 | — | — | — | — |
| *Anillinus cherokee* Sokolov & Carlton NC, Cherohala Skyway | 78Cher | — | — | FJ765224 | FJ765224 | — | — | — | — |
| *Anillinus cherokee* Sokolov & Carlton NC, Chunky Gal trail | CWH_506 | **—** | **—** | **—** | **OR839713** | **—** | **—** | **—** | **—** |
| *Anillinus cherokee* Sokolov & Carlton NC, Copper Ridge Bald | CWH_504 | **—** | **—** | **—** | **OR839711** | **—** | **—** | **—** | **—** |
| *Anillinus cherokee* Sokolov & Carlton NC, Copper Ridge Bald | CWH_505 | **—** | **—** | **—** | **OR839712** | **—** | **—** | **—** | **—** |
| *Anillinus cherokee* Sokolov & Carlton NC, Cowee Bald | CWH_507 | **—** | **OR853195** | **—** | **OR839714** | **—** | **—** | **OR838266** | **—** |
| *Anillinus cherokee* Sokolov & Carlton NC, Ellicott Rock | SSM248 | **—** | **OR853191** | **—** | **OR839797** | **—** | **—** | **—** | **—** |
| *Anillinus cherokee* Sokolov & Carlton NC, Stratton Ridge | CWH_191 | **—** | **OR853194** | **OR839236** | **OR839519** | **OR837852** | **OR837998** | **OR838185** | **—** |
| *Anillinus cherokee* Sokolov & Carlton SC, Coon Branch | CWH_103 | **—** | **OR853196** | **OR839237** | **OR839450** | **OR837812** | **OR837969** | **OR838157** | **OR838117** |
| *Anillinus cherokee* Sokolov & Carlton SC, Coon Branch | CWH_104 | **—** | **OR853197** | **—** | **OR839451** | **—** | **—** | **—** | **—** |
| *Anillinus cherokee* Sokolov & Carlton SC, Coon Branch | CWH_105 | **—** | **OR853198** | **—** | **OR839452** | **—** | **—** | **—** | **—** |
| *Anillinus cherokee* Sokolov & Carlton SC, Coon Branch | CWH_106 | **—** | **OR853199** | **—** | **—** | **—** | **—** | **—** | **—** |
| *Anillinus cherokee* Sokolov & Carlton SC, Coon Branch | CWH_108 | **—** | **—** | **—** | **OR839453** | **OR837813** | **—** | **—** | **—** |
| *Anillinus cherokee* Sokolov & Carlton SC, Coon Branch | CWH_109 | **—** | **—** | **—** | **OR839454** | **—** | **—** | **—** | **—** |
| *Anillinus cherokee* Sokolov & Carlton SC, Coon Branch | CWH_110 | **—** | **—** | **—** | **OR839455** | **—** | **—** | **—** | **—** |
| *Anillinus cherokee* Sokolov & Carlton SC, Doran Creek | CWH_357 | **—** | **OR853192** | **OR839234** | **OR839641** | **OR837907** | **—** | **OR838242** | **—** |
| *Anillinus loweae* Sokolov & Carlton GA, Rabun Cliffs | CWH_280 | **—** | **OR853254** | **—** | **OR839602** | **—** | **—** | **—** | **—** |
| *Anillinus loweae* Sokolov & Carlton GA, Rabun Cliffs | CWH_281 | **—** | **OR853255** | **—** | **OR839603** | **—** | **—** | **—** | **—** |
| *Anillinus loweae* Sokolov & Carlton NC, Art Loeb trail | MSC2462 | **—** | **—** | **—** | **OR839743** | **—** | **—** | **—** | **—** |
| *Anillinus loweae* Sokolov & Carlton NC, Balsam Mountain Pres. | CWH_135 | **—** | **—** | **—** | **OR839479** | **OR837823** | **—** | **—** | **—** |
| *Anillinus loweae* Sokolov & Carlton NC, Balsam Mountain Pres. | CWH_136 | **—** | **OR853256** | **—** | **OR839480** | **OR837824** | **OR837978** | **OR838165** | **—** |
| *Anillinus loweae* Sokolov & Carlton NC, Balsam Mountain Pres. | CWH_137 | **—** | **—** | **—** | **OR839481** | **—** | **—** | **—** | **—** |
| *Anillinus loweae* Sokolov & Carlton NC, Balsam Mountain Pres. | SSM229 | **—** | **—** | **—** | **OR839788** | **—** | **—** | **—** | **—** |
| *Anillinus loweae* Sokolov & Carlton NC, Balsam Mountain Pres. | SSM231 | **—** | **—** | **—** | **OR839789** | **—** | **—** | **—** | **—** |
| *Anillinus loweae* Sokolov & Carlton NC, Balsam Mountain Pres. | SSM236 | **—** | **—** | **—** | **OR839793** | **—** | **—** | **—** | **—** |
| *Anillinus loweae* Sokolov & Carlton NC, Balsam Mountain Pres. | SSM237 | **—** | **OR853257** | **—** | **OR839794** | **—** | **—** | **—** | **—** |
| *Anillinus loweae* Sokolov & Carlton NC, Balsam Mountain Pres. | SSM412 | **—** | **—** | **—** | **OR839807** | **—** | **—** | **—** | **—** |
| *Anillinus loweae* Sokolov & Carlton NC, Balsam Mountain Pres. | SSM413 | **—** | **—** | **—** | **OR839808** | **—** | **—** | **—** | **—** |
| *Anillinus loweae* Sokolov & Carlton NC, Baxter Creek trail | 53Low | — | — | FJ765228 | FJ765228 | — | — | — | — |
| *Anillinus loweae* Sokolov & Carlton NC, Bradley Fork trail | 25Low | — | — | FJ765225 | FJ765225 | — | — | — | — |
| *Anillinus loweae* Sokolov & Carlton NC, Bradley Fork trail | 27Low | — | — | FJ765226 | FJ765226 | — | — | — | — |
| *Anillinus loweae* Sokolov & Carlton NC, Courthouse Falls | SSM263 | **—** | **—** | **—** | **OR839800** | **—** | **—** | **—** | **—** |
| *Anillinus loweae* Sokolov & Carlton NC, Courthouse Falls | SSM264 | **—** | **—** | **—** | **OR839801** | **—** | **—** | **—** | **—** |
| *Anillinus loweae* Sokolov & Carlton NC, Courthouse Falls | SSM265 | **—** | **—** | **—** | **OR839802** | **—** | **—** | **—** | **—** |
| *Anillinus loweae* Sokolov & Carlton NC, Jones Gap | SSM163 | **—** | **—** | **—** | **OR839771** | **—** | **—** | **—** | **—** |
| *Anillinus loweae* Sokolov & Carlton NC, Jones Gap | SSM164 | **—** | **—** | **—** | **OR839772** | **—** | **—** | **—** | **—** |
| *Anillinus loweae* Sokolov & Carlton NC, Jones Gap | SSM165 | **—** | **—** | **—** | **OR839773** | **—** | **—** | **—** | **—** |
| *Anillinus loweae* Sokolov & Carlton NC, Jones Gap | SSM166 | **—** | **—** | **—** | **OR839774** | **—** | **—** | **—** | **—** |
| *Anillinus loweae* Sokolov & Carlton NC, Pisgah Mountain | CWH_477 | **—** | **OR853258** | **OR839279** | **OR839697** | **—** | **—** | **OR838262** | **—** |
| *Anillinus loweae* Sokolov & Carlton NC, Sassafras Mountain | CWH_371 | **—** | **OR853260** | **OR839281** | **OR839647** | **OR837910** | **OR838062** | **OR838244** | **—** |
| *Anillinus loweae* Sokolov & Carlton NC, Transylvania Co. | MSC2456 | **—** | **—** | **—** | **OR839737** | **—** | **—** | **—** | **—** |
| *Anillinus loweae* Sokolov & Carlton NC, Van Hook Glade | CWH_210 | **—** | **OR853259** | **OR839280** | **OR839533** | **OR837861** | **OR838007** | **OR838195** | **—** |
| *Anillinus loweae* Sokolov & Carlton NC, Van Hook Glade | CWH_211 | **—** | **OR853253** | **—** | **OR839534** | **OR837862** | **OR838008** | **OR838196** | **—** |
| *Anillinus loweae* Sokolov & Carlton TN, Albright Grove | 49Low | — | — | FJ765227 | FJ765227 | — | — | — | — |
| *Anillinus loweae* Sokolov & Carlton TN, Newfound Gap | CWH_256 | **—** | **—** | **—** | **OR839579** | **—** | **—** | **—** | **—** |
| *Anillinus merritti* Sokolov & Carlton GA, Rabun Bald | CWH_027 | **—** | **OR853271** | **—** | **OR839390** | **OR837788** | **—** | **OR838141** | **—** |
| *Anillinus merritti* Sokolov & Carlton GA, Rabun Bald | CWH_040 | **—** | **OR853266** | **OR839285** | **OR839393** | **OR837790** | **OR837952** | **OR838143** | **—** |
| *Anillinus merritti* Sokolov & Carlton GA, Rabun Bald | CWH_041 | **—** | **OR853267** | **—** | **OR839394** | **—** | **—** | **—** | **—** |
| *Anillinus merritti* Sokolov & Carlton GA, Rabun Bald | CWH_042 | **—** | **OR853268** | **—** | **OR839395** | **—** | **—** | **—** | **—** |
| *Anillinus merritti* Sokolov & Carlton GA, Rabun Bald | CWH_043 | **—** | **OR853272** | **—** | **OR839396** | **—** | **—** | **—** | **—** |
| *Anillinus merritti* Sokolov & Carlton GA, Rabun Bald | CWH_044 | **—** | **OR853273** | **—** | **OR839397** | **OR837791** | **OR837953** | **—** | **—** |
| *Anillinus merritti* Sokolov & Carlton GA, Rabun Bald | CWH_045 | **—** | **OR853274** | **—** | **OR839398** | **—** | **—** | **—** | **—** |
| *Anillinus merritti* Sokolov & Carlton GA, Rabun Bald | CWH_046 | **—** | **OR853275** | **—** | **OR839399** | **—** | **—** | **—** | **—** |
| *Anillinus merritti* Sokolov & Carlton GA, Rabun Bald | CWH_047 | **—** | **—** | **—** | **OR839400** | **—** | **—** | **—** | **—** |
| *Anillinus merritti* Sokolov & Carlton GA, Rabun Bald | CWH_048 | **—** | **—** | **—** | **OR839401** | **—** | **—** | **—** | **—** |
| *Anillinus merritti* Sokolov & Carlton GA, Rabun Bald | CWH_095 | **—** | **OR853276** | **—** | **OR839445** | **—** | **—** | **—** | **—** |
| *Anillinus merritti* Sokolov & Carlton NC, Cliffside Rec. Area | CWH_203 | **—** | **OR853277** | **—** | **OR839529** | **OR837858** | **OR838004** | **OR838192** | **—** |
| *Anillinus merritti* Sokolov & Carlton NC, Jones Gap | SSM174 | **—** | **—** | **—** | **OR839775** | **—** | **—** | **—** | **—** |
| *Anillinus merritti* Sokolov & Carlton NC, Jones Gap | SSM245 | **—** | **—** | **—** | **OR839795** | **—** | **—** | **—** | **—** |
| *Anillinus merritti* Sokolov & Carlton NC, Jones Gap | SSM246 | **—** | **—** | **—** | **OR839796** | **—** | **—** | **—** | **—** |
| *Anillinus merritti* Sokolov & Carlton NC, Twentymile Station | 45Merr | — | — | FJ765220 | FJ765220 | — | — | — | — |
| *Anillinus merritti* Sokolov & Carlton NC, Twentymile Station | 46Merr | — | — | FJ765221 | FJ765221 | — | — | — | — |
| *Anillinus merritti* Sokolov & Carlton NC, Twentymile Station | 79Merr | — | — | FJ765222 | FJ765222 | — | — | — | — |
| *Anillinus merritti* Sokolov & Carlton NC, Wallace Branch | CWH_208 | **—** | **OR853265** | **—** | **OR839532** | **OR837860** | **OR838006** | **OR838194** | **—** |
| *Anillinus merritti* Sokolov & Carlton NC, Wayah Road | CWH_055 | **—** | **OR853270** | **—** | **OR839408** | **—** | **—** | **—** | **—** |
| *Anillinus merritti* Sokolov & Carlton NC, Wayah Road | CWH_165 | **—** | **OR853278** | **—** | **OR839504** | **OR837841** | **OR837988** | **OR838174** | **—** |
| *Anillinus merritti* Sokolov & Carlton NC, Wayah Road | CWH_166 | **—** | **OR853269** | **OR839286** | **OR839505** | **OR837842** | **OR837989** | **OR838175** | **—** |
| *Anillinus merritti* Sokolov & Carlton SC, Indian Camp Creek | SSM414 | **—** | **OR853279** | **OR839287** | **OR839809** | **—** | **OR838108** | **OR838290** | **—** |
| *Anillinus* sp. "Georgia, Brasstown Bald sp. 2" | CWH_183 | **—** | **OR853306** | **OR839312** | **OR839511** | **OR837847** | **OR837994** | **OR838180** | **—** |
| *Anillinus* sp. "Georgia, Brasstown Bald sp. 2" | CWH_184 | **—** | **—** | **—** | **OR839512** | **OR837848** | **—** | **—** | **—** |
| *Anillinus* sp. "Georgia, Brasstown Bald sp. 2" | CWH_187 | **—** | **OR853305** | **OR839310** | **OR839515** | **OR837850** | **OR837996** | **OR838182** | **—** |
| *Anillinus* sp. "Georgia, Brasstown Bald sp. 2" | CWH_189 | **—** | **—** | **—** | **OR839517** | **—** | **—** | **—** | **—** |
| *Anillinus* sp. "Georgia, Brasstown Bald sp. 2" | CWH_273 | **—** | **—** | **OR839311** | **OR839595** | **—** | **—** | **—** | **—** |
| *Anillinus* sp. "Georgia, Brasstown Bald sp. 2" | CWH_274 | **—** | **—** | **—** | **OR839596** | **—** | **—** | **—** | **—** |
| *Anillinus* sp. "Georgia, Brasstown Bald sp. 2" | CWH_275 | **—** | **—** | **—** | **OR839597** | **—** | **—** | **—** | **—** |
| *Anillinus* sp. "North Carolina, Balsam Mountain Preserve" | CWH_132 | **—** | **OR853343** | **OR839332** | **OR839476** | **OR837821** | **OR837976** | **OR838163** | **—** |
| *Anillinus* sp. "North Carolina, Balsam Mountain Preserve" | CWH_133 | **—** | **OR853344** | **—** | **OR839477** | **—** | **OR837977** | **OR838164** | **—** |
| *Anillinus* sp. "North Carolina, Balsam Mountain Preserve" | CWH_134 | **—** | **—** | **—** | **OR839478** | **OR837822** | **—** | **—** | **—** |
| *Anillinus* sp. "North Carolina, Balsam Mountain Preserve" | SSM232 | **—** | **OR853345** | **—** | **OR839790** | **—** | **—** | **—** | **—** |
| *Anillinus* sp. "North Carolina, Balsam Mountain Preserve" | SSM233 | **—** | **—** | **—** | **OR839791** | **—** | **—** | **—** | **—** |
| *Anillinus* sp. "North Carolina, Balsam Mountain Preserve" | SSM234 | **—** | **—** | **—** | **OR839792** | **—** | **—** | **—** | **—** |
| *Anillinus* sp. "North Carolina, Wayah sp. 2" NC, Chunky Gal Tr. | CWH_278 | **—** | **—** | **—** | **OR839600** | **—** | **—** | **—** | **—** |
| *Anillinus* sp. "North Carolina, Wayah sp. 2" NC, Riley Knob | CWH_180 | **—** | **OR853169** | **—** | **OR839509** | **OR837845** | **OR837992** | **OR838178** | **—** |
| *Anillinus* sp. "North Carolina, Wayah sp. 2" NC, Stratton Ridge | CWH_245 | **—** | **OR853170** | **—** | **OR839568** | **OR837886** | **OR838031** | **OR838220** | **—** |
| *Anillinus* sp. "North Carolina, Wayah sp. 2" NC, Stratton Ridge | CWH_246 | **—** | **OR853162** | **—** | **OR839569** | **OR837887** | **—** | **OR838221** | **—** |
| *Anillinus* sp. "North Carolina, Wayah sp. 2" NC, Wayah Bald | CWH_007 | **—** | **OR853172** | **OR839220** | **OR839376** | **OR837783** | **OR837948** | **OR838136** | **—** |
| *Anillinus* sp. "North Carolina, Wayah sp. 2" NC, Wayah Bald | CWH_008 | **—** | **OR853171** | **—** | **OR839377** | **—** | **—** | **OR838137** | **—** |
| *Anillinus* sp. "North Carolina, Wayah sp. 2" NC, Wayah Bald | CWH_012 | **—** | **OR853163** | **—** | **OR839381** | **—** | **—** | **—** | **—** |
| *Anillinus* sp. "North Carolina, Wayah sp. 2" NC, Wayah Bald | CWH_013 | **—** | **OR853164** | **—** | **—** | **—** | **—** | **—** | **—** |
| *Anillinus* sp. "North Carolina, Wayah sp. 2" NC, Wayah Bald | CWH_014 | **—** | **OR853165** | **—** | **OR839382** | **—** | **—** | **—** | **—** |
| *Anillinus* sp. "North Carolina, Wayah sp. 2" NC, Wayah Bald | CWH_015 | **—** | **OR853173** | **—** | **OR839383** | **OR837786** | **—** | **—** | **—** |
| *Anillinus* sp. "North Carolina, Wayah sp. 2" NC, Wayah Bald | CWH_016 | **—** | **OR853166** | **—** | **OR839384** | **—** | **—** | **—** | **—** |
| *Anillinus* sp. "North Carolina, Wayah sp. 2" NC, Wayah Bald | CWH_017 | **—** | **OR853167** | **—** | **OR839385** | **—** | **—** | **—** | **—** |
| *Anillinus* sp. "North Carolina, Wayah sp. 2" NC, Wayah Bald | CWH_018 | **—** | **OR853168** | **—** | **OR839386** | **—** | **—** | **—** | **—** |
| *Anillinus* sp. "North Carolina, Wayah sp. 2" NC, Wayah Bald | CWH_019 | **—** | **—** | **—** | **OR839387** | **—** | **—** | **—** | **—** |
| *Anillinus* sp. "North Carolina, Wayah sp. 2" NC, Wayah Bald | CWH_020 | **—** | **—** | **—** | **OR839388** | **—** | **—** | **—** | **—** |
| *Anillinus* sp. "North Carolina, Wayah sp. 2" NC, Wayah Bald | CWH_053 | **—** | **—** | **—** | **OR839406** | **—** | **—** | **—** | **—** |
| *Anillinus* sp. "North Carolina, Wayah sp. 2" NC, Wayah Bald | CWH_058 | **—** | **OR853174** | **—** | **OR839411** | **—** | **—** | **—** | **—** |
| *Anillinus* sp. "North Carolina, Wayah sp. 2" NC, Wayah Road | CWH_138 | **—** | **OR853161** | **—** | **OR839482** | **OR837825** | **OR837979** | **OR838166** | **—** |
| ***Anillinus* “*steevesi*-group”** |  |  |  |  |  |  |  |  |  |
| *Anillinus* sp. "Tennessee, Hiawassee sp. 1" | CWH_212 | **—** | **OR853353** | **—** | **OR839535** | **OR837863** | **OR838009** | **OR838197** | **—** |
| *Anillinus* sp. "Tennessee, Hiawassee sp. 1" | CWH_213 | **—** | **—** | **—** | **OR839536** | **—** | **—** | **—** | **—** |
| *Anillinus* sp. "Tennessee, Hiawassee sp. 1" | CWH_214 | **—** | **OR853349** | **—** | **OR839537** | **OR837864** | **OR838010** | **OR838198** | **—** |
| *Anillinus* sp. "Tennessee, Hiawassee sp. 1" | CWH_215 | **—** | **—** | **—** | **OR839538** | **—** | **—** | **—** | **—** |
| *Anillinus* sp. "Tennessee, Hiawassee sp. 2" | CWH_372 | **—** | **OR853119** | **OR839193** | **OR839648** | **—** | **OR838063** | **—** | **—** |
| *Anillinus barri* Sokolov & Carlton TN, Tellico River | CWH_158 | **—** | **OR853140** | **OR839208** | **OR839497** | **OR837837** | **OR837985** | **OR838171** | **OR838121** |
| *Anillinus* cf*. steevesi* Barr AL, Bucks Pocket | CWH_463 | **—** | **OR853354** | **OR839336** | **OR839687** | **—** | **OR838090** | **OR838259** | **—** |
| *Anillinus* cf*. steevesi* Barr GA, Cloudland Canyon | 23Stee | — | — | FJ765210 | FJ765210 | — | — | — | — |
| *Anillinus* cf*. steevesi* Barr GA, Cloudland Canyon | 29Stee | — | — | FJ765211 | FJ765211 | — | — | — | — |
| *Anillinus* cf*. steevesi* Barr GA, Cloudland Canyon | 30Stee | — | — | FJ765212 | FJ765212 | — | — | — | — |
| *Anillinus* cf*. steevesi* Barr NC, Chilhowee Mountain | 65Stee | — | — | FJ765214 | FJ765214 | — | — | — | — |
| *Anillinus* cf*. steevesi* Barr NC, Hazel Creek | 56Stee | — | — | FJ765213 | FJ765213 | — | — | — | — |
| *Anillinus* cf*. steevesi* Barr NC, Parsons Branch Road | 66Stee | — | — | FJ765215 | FJ765215 | — | — | — | — |
| *Anillinus* cf*. steevesi* Barr TN, Rich Mountain | CWH_250 | **—** | **OR853388** | **OR839360** | **OR839573** | **OR837890** | **OR838034** | **OR838224** | **—** |
| *Anillinus* cf*. steevesi* Barr TN, Rich Mountain | CWH_251 | **—** | **—** | **—** | **OR839574** | **OR837891** | **OR838035** | **—** | **—** |
| *Anillinus* cf*. steevesi* Barr TN, Rich Mountain | CWH_252 | **—** | **—** | **—** | **OR839575** | **—** | **—** | **—** | **—** |
| *Anillinus* cf*. steevesi* Barr TN, Rich Mountain | CWH_255 | **—** | **—** | **—** | **OR839578** | **—** | **—** | **—** | **—** |
| *Anillinus* cf*. steevesi* Barr TN, Turkeypen Ridge | CWH_260 | **—** | **OR853355** | **—** | **OR839583** | **—** | **—** | **—** | **—** |
| *Anillinus chilhowee* Sokolov TN, Lakeview Overlook | CWH_228 | **—** | **OR853201** | **OR839238** | **OR839551** | **OR837874** | **OR838019** | **OR838207** | **—** |
| *Anillinus chilhowee* Sokolov TN, Lakeview Overlook | CWH_229 | **—** | **OR853200** | **—** | **OR839552** | **OR837875** | **OR838020** | **OR838208** | **—** |
| *Anillinus sp.* "Georgia, Barnes Creek sp. 2" | CWH_223 | **—** | **OR853350** | **OR839334** | **OR839546** | **OR837869** | **OR838015** | **OR838203** | **—** |
| *Anillinus sp.* "Georgia, Barnes Creek sp. 2" | CWH_224 | **—** | **—** | **—** | **OR839547** | **OR837870** | **—** | **—** | **—** |
| *Anillinus sp.* "Georgia, Barnes Creek sp. 2" | CWH_225 | **—** | **OR853351** | **—** | **OR839548** | **OR837871** | **OR838016** | **OR838204** | **—** |
| *Anillinus sp.* "Georgia, Galts Ferry" | CWH_441 | **—** | **OR853121** | **OR839196** | **OR839674** | **—** | **—** | **—** | **—** |
| *Anillinus sp.* "Georgia, Tearbritches Trail" | CWH_227 | **—** | **OR853352** | **OR839335** | **OR839550** | **OR837873** | **OR838018** | **OR838206** | **—** |
| *Anillinus* sp. "Tennessee, Thunder Rock" | CWH_373 | **—** | **OR853232** | **OR839264** | **OR839649** | **—** | **OR838064** | **—** | **—** |
| *Anillinus juliae* Sokolov & Carlton TN, Starr Mountain | 42Juli | — | — | FJ765216 | FJ765216 | — | — | — | — |
| *Anillinus juliae* Sokolov & Carlton TN, Starr Mountain | 43Juli | — | — | FJ765217 | FJ765217 | — | — | — | — |
| ***Serranillus*** |  |  |  |  |  |  |  |  |  |
| *Serranillus* sp. “North Carolina, Miller Cove” NC, Santeetlah Lake | DNA2309/DNA1084 | GU556145 | GU556116 | JN171149 | — | MK118284 | JN170958 | JN171550 | MK121287 |
| *Serranillus* sp. “North Carolina, Miller Cove” GA, Barnes Crk Falls | CWH_380 | **—** | **OR853390** | **—** | **OR839656** | **—** | **—** | **—** | **—** |
| *Serranillus* sp. “North Carolina, Miller Cove” GA, Little Bald | CWH_185 | **—** | **OR853391** | **—** | **OR839513** | **—** | **—** | **—** | **—** |
| *Serranillus* sp. “North Carolina, Miller Cove” NC, Miller Cove | SSM197 | **—** | **—** | **—** | **OR839783** | **—** | **—** | **—** | **—** |
| *Serranillus* sp. “North Carolina, Miller Cove” NC, Miller Cove | SSM198 | **—** | **—** | **—** | **OR839784** | **—** | **—** | **—** | **—** |
| *Serranillus* sp. “North Carolina, Miller Cove” NC, Miller Cove | SSM199 | **OR853118** | **OR853392** | **OR839361** | **OR839785** | **OR837945** | **OR838107** | **OR838287** | **—** |
| *Serranillus* sp. “North Carolina, Miller Cove” NC, Miller Cove | SSM200 | **—** | **—** | **—** | **OR839786** | **—** | **—** | **—** | **—** |
| *Serranillus* sp. “North Carolina, Miller Cove” NC, Miller Cove | SSM201 | **—** | **—** | **—** | **OR839787** | **—** | **—** | **—** | **—** |
| *Serranillus jeanneli* Barr SC, Coon Branch | CWH_454 | **—** | **OR853398** | **OR839364** | **OR839681** | **OR837924** | **OR838086** | **OR838255** | **—** |
| *Serranillus dunavani* (Jeannel) NC, Shooting Creek Bald | CWH_381 | **—** | **OR853389** | **—** | **OR839657** | **—** | **—** | **—** | **—** |
| *Serranillus dunavani* (Jeannel) NC, Twentymile trail | Sdun52 | — | — | FJ765229 | FJ765229 | — | — | — | — |
| *Serranillus dunavani* (Jeannel) SC, Ashmore Heritage Preserve | MSC2463 | **—** | **—** | **—** | **OR839744** | **—** | **—** | **—** | **—** |
| *Serranillus dunavani* (Jeannel) SC, Cedar Springs Road | CWH_267 | **—** | **OR853393** | **—** | **OR839590** | **OR837895** | **OR838040** | **—** | **—** |
| *Serranillus dunavani* (Jeannel) SC, Chimneytop Gap | MSC2457 | **—** | **—** | **—** | **OR839738** | **—** | **—** | **—** | **—** |
| *Serranillus dunavani* (Jeannel) SC, East Fork trail | SSM415 | **—** | **OR853397** | **—** | **OR839810** | **—** | **—** | **—** | **—** |
| *Serranillus dunavani* (Jeannel) SC, Eastatoe Creek | MSC2455 | **—** | **—** | **—** | **OR839736** | **—** | **—** | **—** | **—** |
| *Serranillus dunavani* (Jeannel) SC, Eastatoe Creek | MSC2461 | **—** | **—** | **—** | **OR839742** | **—** | **—** | **—** | **—** |
| *Serranillus dunavani* (Jeannel) SC, Martin Creek Landing | CWH_448 | **—** | **OR853394** | **—** | **OR839679** | **OR837922** | **OR838083** | **—** | **—** |
| *Serranillus dunavani* (Jeannel) SC, Nine Times Preserve | SSM184 | **—** | **—** | **—** | **OR839776** | **—** | **—** | **—** | **—** |
| *Serranillus dunavani* (Jeannel) SC, Nine Times Preserve | SSM185 | **—** | **—** | **—** | **OR839777** | **—** | **—** | **—** | **—** |
| *Serranillus dunavani* (Jeannel) SC, Nine Times Preserve | SSM186 | **—** | **—** | **—** | **OR839778** | **—** | **—** | **OR838283** | **—** |
| *Serranillus dunavani* (Jeannel) SC, Nine Times Preserve | SSM187 | **—** | **—** | **—** | **OR839779** | **—** | **—** | **OR838284** | **—** |
| *Serranillus dunavani* (Jeannel) SC, Nine Times Preserve | SSM188 | **—** | **OR853407** | **OR839369** | **OR839780** | **OR837944** | **OR838105** | **OR838285** | **—** |
| *Serranillus dunavani* (Jeannel) SC, Nine Times Preserve | SSM189 | **—** | **OR853395** | **OR839362** | **OR839781** | **—** | **OR838106** | **OR838286** | **—** |
| *Serranillus dunavani* (Jeannel) SC, Nine Times Preserve | SSM190 | **—** | **—** | **—** | **OR839782** | **—** | **—** | **—** | **—** |
| *Serranillus dunavani* (Jeannel) SC, Sassafras Mountain larva | CWH_311 | **—** | **—** | **—** | **OR839609** | **—** | **—** | **—** | **—** |
| *Serranillus dunavani* (Jeannel) SC, Sassafras Mountain | CWH_312 | **—** | **—** | **—** | **OR839610** | **—** | **—** | **—** | **—** |
| *Serranillus dunavani* (Jeannel) SC, Sassafras Mountain | DNA5828 | ON557661 | ON557653 | ON554762 | — | — | ON721284 | ON721288 | ON721290 |
| *Serranillus dunavani* (Jeannel) SC, Sassafras Mountain | SSM68 | **—** | **—** | **—** | **OR839814** | **—** | **—** | **—** | **—** |
| *Serranillus dunavani* (Jeannel) SC, Sassafras Mountain | SSM69 | **—** | **—** | **—** | **OR839815** | **—** | **—** | **—** | **—** |
| *Serranillus dunavani* (Jeannel) SC, Sassafras Mountain | SSM70 | **—** | **—** | **—** | **OR839816** | **—** | **—** | **—** | **—** |
| *Serranillus dunavani* (Jeannel) SC, Sassafras Mountain | SSM71 | **—** | **—** | **—** | **OR839817** | **—** | **—** | **—** | **—** |
| *Serranillus dunavani* (Jeannel) SC, Sassafras Mountain | SSM72 | **—** | **—** | **—** | **OR839818** | **—** | **—** | **OR838294** | **—** |
| *Serranillus dunavani* (Jeannel) SC, Sassafras Mountain | SSM73 | **—** | **OR853396** | **OR839363** | **OR839819** | **OR837946** | **OR838111** | **OR838295** | **—** |
| *Serranillus dunavani* (Jeannel) SC, Sassafras Mountain | SSM74 | **—** | **—** | **—** | **OR839820** | **—** | **—** | **—** | **—** |
| *Serranillus septentrionis* Sokolov & Carlton VA, Cascades | CWH_519 | **—** | **OR853401** | **—** | **OR839724** | **—** | **—** | **OR838269** | **—** |
| *Serranillus septentrionis* Sokolov & Carlton VA, Flat Top Mtn. | CWH_101 | **OR853107** | **OR853400** | **OR839365** | **OR839448** | **OR837810** | **OR837967** | **OR838155** | **OR838116** |
| *Serranillus* sp. "Alabama, Highland Lake" | CWH_202 | **OR853108** | **OR853402** | **OR839366** | **OR839528** | **OR837857** | **OR838003** | **OR838191** | **OR838122** |
| *Serranillus* sp. "North Carolina, Big Butt" NC, Big Butt trail | SSM127 | **—** | **OR853405** | **OR839368** | **OR839751** | **—** | **OR838102** | **OR838280** | **—** |
| *Serranillus* sp. "North Carolina, Big Butt" NC, Big Butt trail | SSM128 | **—** | **—** | **—** | **OR839752** | **—** | **—** | **—** | **—** |
| *Serranillus* sp. "North Carolina, Big Butt" NC, Camp Creek Bald | CWH_522 | **—** | **OR853404** | **—** | **—** | **—** | **—** | **OR838271** | **—** |
| *Serranillus* sp. "North Carolina, Big Butt" TN, Big Bald | CWH_370 | **—** | **OR853406** | **—** | **OR839646** | **OR837909** | **OR838061** | **—** | **—** |
| *Serranillus* sp. "South Carolina, Coon Branch" | CWH_400 | **OR853116** | **OR853403** | **OR839367** | **OR839665** | **OR837916** | **OR838073** | **OR838250** | **OR838133** |
| *Serranillus* sp. larva | CWH_207 | **—** | **OR853399** | **—** | **OR839531** | **—** | **—** | **—** | **—** |
| **Western Nearctic Anillini** |  |  |  |  |  |  |  |  |  |
| *Anillodes* sp. “CA, Portola Redwoods” | DNA3636 | ON557658 | MK104107 | ON554758 | ON554758 | MK118368 | MK112258 | MK118704 | MK121345 |
| *Anillodes* sp. “CA, Packsaddle Creek” | CWH_451 | **OR853117** | **OR853387** | **OR839359** | **OR839680** | **OR837923** | **OR838084** | **OR838253** | **OR838134** |
| *Medusapyga alsea* LaBonte OR, Prairie Peak | DNA2576 | MK103916 | MK103988 | ON554759 | ON554759 | MK118299 | MK112149 | MK118588 | MK121300 |
| *Medusapyga chehalis* LaBonte WA, Capitol State Forest | DNA4906 | ON557659 | ON557651 | ON554760 | ON554760 | — | ON721282 | ON721286 | — |
| **World Anillini outgroups** |  |  |  |  |  |  |  |  |  |
| *Anillus cebennicus* Balazuc & Bruneau de Miré France | CA85 | KU978698 | KU978746 | — | KU976321 | — | — | — | — |
| *Binaghites subalpinus* (Baudi) Italy | DNA3248/CA51 | — | MK104079 | — | KU976303 | — | MK112234 | MK118676 | — |
| *Caeconannus rotundicollis* (Jeannel) South Africa | DNA2551 | — | MK103986 | — | — | MK118297 | MK112147 | MK118586 | MK121299 |
| *Geocharidius* sp. Mexico | DNA1763 | JN170251 | JN170466 | JN171139 | — | MK118244 | JN170946 | JN171543 | — |
| *Geocharis* sp. Spain | BMNH1046115 | KU978666 | KU978712 | — | KU976287 | — | — | — | — |
| *Hypotyphlus navaricus* (Coiffait) Spain | CA48 | KU978680 | KU978726 | — | KU976300 | — | — | — | — |
| *Iberanillus vinyasi* Espanol Spain | CA60 | KU978687 | KU978733 | — | KU976308 | — | — | — | — |
| *Illaphanus* cf*. matthewsi* Giachino Australia | DNA1767 | MK103905 | MK103945 | — | — | MK118246 | MK112104 | MK118546 | — |
| *Microcharidius josabelae* (Ortuño & Gilgado) Spain | CA3 | KU978675 | KU978721 | MF539602 | KU976295 | — | — | — | — |
| *Microdipnus jeanneli* (Alluaud) Kenya | DNA1956 | — | MK103958 | — | — | — | MK112118 | MK118559 | — |
| *Nesamblyops* sp. "New Zealand, Mount Robert" | DNA2874 | MK103921 | MK104018 | — | — | MK118325 | — | MK118615 | MK121315 |
| *Nesamblyops* sp. "New Zealand, Tirohanga Track" | DNA2879 | — | MK104023 | — | — | MK118328 | — | MK118620 | — |
| *Orthotyphlus franzi* (Zaballos & Mateu) New Caledonia | CA50 | KU978682 | KU978728 | — | KU976302 | — | — | — | — |
| *Parvocaecus* sp. Turkey | CA52 | KU978684 | KU978730 | — | KU976304 | — | — | — | — |
| *Pseudanillus* sp. Morocco | CA96 | KU978702 | KU978751 | — | KU976326 | — | — | — | — |
| *Rhegmatobius* sp. Italy, Sardinia | CA58 | KU978686 | KU978732 | — | KU976306 | — | — | — | — |
| *Typhlocharis armata* Coiffait Spain | DNA0572/DNA1718 | GU556152 | GU556130 | JN171157 | — | — | JN170970 | JN171559 | MK121251 |
| **Trechitae outgroups** |  |  |  |  |  |  |  |  |  |
| *Bembidarenas nr. reicheellum* (Csiki) Chile | DNA2213 | JN170140 | JN170274 | JN170980 | — | MK118277 | JN170740 | JN171345 | MK121283 |
| *Bembidion planum* (Haldeman) Indiana, USA | DNA1423 | JN170216 | JF800048 | JF800067 | — | MK118230 | JN170879 | JN171478 | MK121240 |
| *Darlingtonea kentuckensis* Valentine Kentucky, USA | DNA4292 | MK103932 | MK104111 | — | — | MK118372 | MK112262 | MK118708 | MK121349 |
| *Horologion hubbardi* Harden & Davidson Virginia, USA | CWH_452 | OR505843 | OR505933 | OR500886 | OR500886 | OR503053 | OR503061 | OR503098 | OR503063 |
| *Merizodus* sp. "Chile: Valdivia" | DNA2199 | JN170255 | JN170471 | JN171144 | — | MK118275 | JN170952 | JN171548 | MK121281 |
| *Phrypeus rickseckeri* Hayward California/Montana, USA | DNA0692/DNA0776/DNA2341 | JN170258 | GU556113 | JN171147 | — | MK118288 | JN170956 | GU556056 | MK121291 |
| *Polyderis laeva* (Say) Indiana/Tennessee, USA | DNA2913/CWH_265 | MK103923 | MK104043 | — | OR839588 | MK118340 | MK112200 | MK118640 | MK121323 |
