# Supplementary material for: ﻿Systematics and biogeography of Appalachian Anillini, and a taxonomic review of the species of South Carolina (Coleoptera, Carabidae, Trechinae, Anillini)
Source: Zookeys. 2024 Aug 8;1209:69–197. doi: 10.3897/zookeys.1209.125897 (PMC11336398; doi:10.3897/zookeys.1209.125897)
Supplement: ﻿Supplementary material 3 — Systematic checklist of eastern Nearctic Anillini [file zookeys-1209-069_article-125897__-s003.docx]

| ***Anillinus* affabilis group**  *Anillinus relictus* Sokolov 2021 **AL**  *Anillinus* sp. “Alabama, Aladdin Cave sp. 2” **AL** | |
| --- | --- |
| ***Anillinus* davidsoni group**  *Anillinus davidsoni* Sokolov 2012 **AL**  *Anillinus* sp. “Alabama, Morgan Co.” **AL**  ***Anillinus* tombarri group**  *Anillinus tombarri* Sokolov 2012 **AL**  ***Anillinus* moseleyae group** | |
| *Anillinus carltoni* Sokolov, 2011 **TN** | |
| *Anillinus moseleyae* Sokolov & Carlton, 2004 **NC, TN** | |
| *Anillinus unicoi* Sokolov, 2011 **NC** | |
| *Anillinus* sp. "North Carolina, Wayah sp. 1" **NC** | |
| ***Anillinus* “Kentucky, Hestand sp. 1”** | |
| *Anillinus* sp. "Kentucky, Hestand sp. 1" **KY** | |
| ***Anillinus* indianae group** | |
| *Anillinus indianae* Jeannel, 1963 **IN** | |
| *Anillinus longiceps* Jeannel, 1963 **TN** | |
| *Anillinus* sp. "Kentucky, Hestand sp. 2" **KY** | |
| *Anillinus* sp. "Kentucky, Jessamine" **KY** | |
| ***Anillinus* folkertsi group** | |
| *Anillinus folkertsi* Sokolov & Carlton, 2004 **AL** | |
| *Anillinus folkertsioides* Sokolov, 2020 **AL** | |
| *Anillinus* sp. "Alabama, Manitou Cave Preserve sp. 1" **AL** | |
| *Anillinus sp.* “Tennessee, Cedar Glades” **TN** | |
| ***Anillinus* barberi group** | |
| *Anillinus barberi* Jeannel, 1963 **DC, MD, VA, WV** | |
| *Anillinus* sp. "North Carolina, Boulder Field" **NC** | |
| *Anillinus* sp. "North Carolina, Orange Co. sp. 1" **NC** | |
| *Anillinus* sp. "Tennessee, Big Bald sp. 2" **NC, TN** | |
| *Anillinus* sp. "Virginia, Norton" **VA** | |
| *Anillinus* sp. "Virginia, Patrick Co." **VA** | |
| *Anillinus* sp. “Virginia, Somerset” **VA** | |
| *Anillinus* sp. "Virginia, Whitetop Mountain" **VA** | |
| ***Anillinus* hirsutus group** | |
| *Anillinus clinei* Sokolov, 2020 **AL** | |
| *Anillinus hildebrandti* Sokolov, 2020 **AL** | |
| *Anillinus hirsutus* Sokolov, 2020 **AL**  *Anillinus* sp. “Alabama, Aladdin Cave sp. 1” **AL**  *Anillinus* sp. “Alabama, Cave Mountain Cave” **AL** | |
| *Anillinus* sp. "Alabama, Manitou Cave Preserve sp. 2" **AL** | |
| *Anillinus* sp. “Georgia, Galts Ferry sp. 2” **GA** | |
| *Anillinus sp.* “Georgia, Horseshoe Cave” **GA** | |
| *Anillinus sp.* “Georgia, Johnson Crook” **GA** | |
| *Anillinus sp.* “Georgia, Morrison Cave” **GA** | |
| *Anillinus* sp. "Kentucky, Hestand sp. 4" **KY** | |
| *Anillinus* sp. "Tennessee, Big Bald sp. 1" **TN** | |
| *Anillinus* sp. "Tennessee, Big Bald sp. 3" **TN** | |
| *Anillinus* sp. "Virginia, Breaks sp. 1" **VA**  ***Anillinus* cavicola group**  *Anillinus cavicola* Sokolov, 2012 **AL**  *Anillinus* sp. “Alabama, Cornelison Cave” **AL** | |
| ***Anillinus* erwini group** | |
| *Anillinus erwini* Sokolov & Carlton, 2004 **NC, TN, VA** | |
| ***Anillinus* dentatus group** | |
| *Anillinus dentatus* Harden & Caterino, 2024 **SC** | |
| ***Anillinus* valentinei group** | |
| *Anillinus castaneus* Harden & Caterino, 2024 **SC** | |
| *Anillinus chandleri* Sokolov, 2011 **SC** | |
| *Anillinus cornelli* Sokolov & Carlton, 2004 **NC, SC** | |
| *Anillinus gimmeli* Sokolov & Carlton, 2010 **TN**  *Anillinus humicolus* Sokolov, 2020 **AL** | |
| *Anillinus kingi* Sokolov, 2012 **AL** | |
| *Anillinus murrayae* Sokolov & Carlton, 2004 **NC, SC** | |
| *Anillinus simplex* Harden & Caterino, 2024 **NC, SC** | |
| *Anillinus smokiensis* Sokolov, 2011 **TN**  *Anillinus valentinei* (Jeannel) **AL**  *Anillinus* sp. “Alabama, Horseshoe Cave sp. 1” (=”obsese”) **AL** | |
| *Anillinus* sp. "Alabama, Krawczyk Caverns" **AL** | |
| *Anillinus* sp. "Alabama, Little River" **AL**  *Anillinus* sp. “Alabama, MacFarland Blowing Cave” **AL** | |
| *Anillinus* sp. "Alabama, Magic City Cave" **AL**  *Anillinus* sp. “Alabama, Marshall Co.” **AL**  *Anillinus* sp. “Alabama, McCluney Cave” **AL**  *Anillinus* sp. “Alabama, Princeton” **AL** | |
| *Anillinus* sp. "Alabama, Tidwell Hollow" **AL**  *Anillinus* sp. “Alabama, Warnock Cave” **AL**  *Anillinus* sp. “Georgia, Hickman Gulf Cave” **GA**  *Anillinus* sp. “Georgia, McLemore” **GA** | |
| *Anillinus* sp. “Kentucky, Big Black Mountain” **KY** | |
| *Anillinus* sp. "Kentucky, Laurel Co." **KY**  *Anillinus* sp. “Kentucky, Raven Rock” **KY**  *Anillinus* sp. “North Carolina, Iredell Co.” **NC**  *Anillinus* sp. “South Carolina, Chestnut Ridge” **SC** | |
| *Anillinus* sp. "South Carolina, Long Cane" **SC** | |
| *Anillinus* sp. "South Carolina, Waldrop Stone" **SC**  *Anillinus* sp. “Tennessee, Jim Creek Nat. Pres.” **TN**  *Anillinus* sp. “Tennessee, Lost Cove” **TN** | |
| *Anillinus* sp. "Tennessee, Ozone" **TN** | |
| *Anillinus* sp. "Tennessee, Savage Gulf" **TN** | |
| *Anillinus* sp. "Virginia, Breaks sp. 2" **VA** | |
| ***Anillinus* albrittonorum group** | |
| *Anillinus albrittonorum* Sokolov & Schnepp, 2021 **FL** | |
| *Anillinus jancae* Harden & Caterino, 2024 **SC** | |
| ***Anillinus* pecki group** | |
| *Anillinus docwatsoni* Sokolov & Carlton, 2004 **NC** | |
| *Anillinus pecki* Giachino, 2011 **NC, TN, VA** | |
| ***Anillinus* sinuaticollis group** | |
| *Anillinus felicianus* Sokolov, 2021 **?AL, LA** | |
| *Anillinus choestoea* Harden & Caterino, 2024 **SC** | |
| *Anillinus mica* Harden & Caterino, 2024 **SC** | |
| *Anillinus micamicus* Harden & Caterino, 2024 **SC** | |
| *Anillinus seneca* Harden & Caterino, 2024 **SC**  *Anillinus sinuaticollis* Jeannel, 1963 **TN** | |
| *Anillinus* sp. "Kentucky, Hestand sp. 3" **KY**  *Anillinus* sp. “South Carolina, Coon Branch” **SC** | |
| *Anillinus* sp. "Tennessee, Webb Cave" **TN** | |
| ***Anillinus* elongatus group** | |
| *Anillinus arenicollis* Harden & Caterino, 2024 **SC** | |
| *Anillinus elongatus* Jeannel, 1963 **NC** | |
| *Anillinus montrex* Harden & Caterino, 2024 **?NC, SC** | |
| *Anillinus pittsylvanicus* Harden & Caterino, 2024 **VA** | |
| *Anillinus uwharrie* Harden & Caterino, 2024 **NC** | |
| *Anillinus* sp. “North Carolina, Mint Hill” **NC** | |
| *Anillinus* sp. "North Carolina, Orange Co. Sp. 2" **NC** | |
| ***Anillinus* “Tennessee, Kings Saltpeter Cave”** | |
| *Anillinus* sp. "Tennessee, Kings Saltpeter Cave" **TN** | |
| ***Anillinus* langdoni group** | |
| *Anillinus balli* Sokolov & Carlton, 2004 **KY** | |
| *Anillinus cieglerae* Sokolov & Carlton, 2007 **TN** | |
| *Anillinus daggyi* Sokolov & Carlton, 2004 **NC** | |
| *Anillinus langdoni* Sokolov & Carlton, 2004 **TN** | |
| *Anillinus nantahala* Dajoz, 2005 **NC, GA, SC, TN** | |
| *Anillinus pusillus* Sokolov & Carlton, 2007 **NC** | |
| *Anillinus virginiae* Jeannel, 1963 **VA, WV** | |
| *Anillinus* sp. "Georgia, Barnes Creek sp. 1" **GA** |  |
| *Anillinus* sp. "Georgia, Brasstown Bald sp. 1" **GA, ?SC** | |
| *Anillinus* sp. "North Carolina, Joyce Kilmer" **NC** | |
| *Anillinus* sp. "Tennessee, Indian Boundary” **TN** | |
| ***Anillinus* loweae group** | |
| *Anillinus cherokee* Sokolov & Carlton, 2008 **NC, GA, SC, TN** | |
| *Anillinus fortis* (Horn, 1869) **NC** | |
| *Anillinus loweae* Sokolov & Carlton, 2004 **NC, GA, SC, TN** | |
| *Anillinus merritti* Sokolov & Carlton, 2010 **NC, GA, SC** | |
| *Anillinus* sp. "Georgia, Brasstown Bald sp. 2" **GA** | |
| *Anillinus* sp. "North Carolina, Balsam Mountain Preserve" **NC** | |
| *Anillinus* sp. "North Carolina, Wayah sp. 2” **NC** | |
| ***Anillinus* steevesi group** | |
| *Anillinus barri* Sokolov & Carlton, 2004 **TN** | |
| *Anillinus chilhowee* Sokolov, 2011 **TN** | |
| *Anillinus inexpectatus* Sokolov, 2014 **TN** | |
| *Anillinus juliae* Sokolov & Carlton, 2010 **TN** | |
| *Anillinus steevesi* Barr, 1995 **AL, MS, NC, TN, GA** | |
| *Anillinus* sp. "Georgia, Barnes Creek sp. 2" **GA** | |
| *Anillinus* sp. "Georgia, Galts Ferry sp.1" **GA** | |
| *Anillinus* sp. “Georgia, Mount Ogelthorpe” **GA**  *Anillinus* sp. “Georgia, Potatopatch Mountain” **GA** | |
| *Anillinus* sp. "Georgia, Tearbritches Trail" **GA** | |
| *Anillinus* sp. "Tennessee, Hiawassee sp. 1" **TN** | |
| *Anillinus* sp. "Tennessee, Hiawassee sp. 2" **TN** | |
| *Anillinus* sp. "Tennessee, Thunder Rock" **TN** | |
| ***Anillinus incertae sedis*** | |
| *Anillinus campbelli* Giachino, 2011 **NC** | |
| *Anillinus dohrni* (Ehlers, 1884) **FL** | |
| *Anillinus kovariki* Sokolov & Carlton, 2004 **FL**  *Anillinus turneri* Jeannel, 1963 **GA** | |
| *Anillinus* sp. “South Carolina, Wateree” **SC** | |
| ***Serranillus*** | |
| *Serranillus dunavani* (Jeannel, 1963) **NC, SC** | |
| *Serranillus jeanneli* Barr, 1995 **GA, NC, SC**  *Serranillus magnus* (Zaballos & Mateu, 1997) **GA**, **?AL** | |
| *Serranillus monadnock* Harden & Caterino, 2024 **SC**  *Serranillus septentrionis* Sokolov & Carlton, 2008 **VA**  *Serranillus* sp. “Alabama, Bangor Cave” **AL**  *Serranillus* sp. “Alabama, Bat Cave” **AL** | |
| *Serranillus* sp. "Alabama, Highland Lake" **AL**  *Serranillus* sp. “Georgia, Galt’s Landing” **GA** | |
| *Serranillus* sp. “Georgia, Rabun Bald sp. 1” **GA**  *Serranillus* sp. “Georgia, Rabun Bald sp. 3” **GA** | |
| *Serranillus* sp. "North Carolina, Big Butt" **NC, TN**  *Serranillus* sp. “North Carolina, Miller Cove” **NC**, **GA** | |
| *Serranillus* sp. “North Carolina, Riley Knob” **NC** | |
| *Serranillus* sp. "South Carolina, Coon Branch" **SC** | |
